# Allelic effects on uromodulin aggregates drive autosomal dominant tubulointerstitial kidney disease

**DOI:** 10.15252/emmm.202318242

**Published:** 2023-10-26

**Authors:** Guglielmo Schiano, Jennifer Lake, Marta Mariniello, Céline Schaeffer, Marianne Harvent, Luca Rampoldi, Eric Olinger, Olivier Devuyst

**Affiliations:** ^1^ Mechanisms of Inherited Kidney Disorders, Institute of Physiology University of Zurich Zurich Switzerland; ^2^ Molecular Genetics of Renal Disorders, Division of Genetics and Cell Biology IRCCS San Raffaele Scientific Institute Milan Italy; ^3^ Institut de Recherche Expérimentale et Clinique UCLouvain Brussels Belgium; ^4^ Translational and Clinical Research Institute Newcastle University Newcastle upon Tyne UK; ^5^ Center for Human Genetics Cliniques Universitaires Saint‐Luc, UCLouvain Brussels Belgium

**Keywords:** ADTKD‐*UMOD*, aggregates, gain‐of‐function, kidney fibrosis, unfolded protein response, Urogenital System

## Abstract

Missense mutations in the uromodulin (*UMOD*) gene cause autosomal dominant tubulointerstitial kidney disease (ADTKD), one of the most common monogenic kidney diseases. The unknown impact of the allelic and gene dosage effects and fate of mutant uromodulin leaves open the gap between postulated gain‐of‐function mutations, end‐organ damage and disease progression in ADTKD. Based on two prevalent missense *UMOD* mutations with divergent disease progression, we generated *Umod* ^*C171Y*^ and *Umod* ^*R186S*^ knock‐in mice that showed strong allelic and gene dosage effects on uromodulin aggregates and activation of ER stress and unfolded protein and immune responses, leading to variable kidney damage. Deletion of the wild‐type *Umod* allele in heterozygous *Umod* ^*R186S*^ mice increased the formation of uromodulin aggregates and ER stress. Studies in kidney tubular cells confirmed differences in uromodulin aggregates, with activation of mutation‐specific quality control and clearance mechanisms. Enhancement of autophagy by starvation and mTORC1 inhibition decreased uromodulin aggregates. These studies substantiate the role of toxic aggregates as driving progression of ADTKD‐*UMOD*, relevant for therapeutic strategies to improve clearance of mutant uromodulin.

## Graphical abstract

Representative missense mutations in the *UMOD* gene causing ADTKD differentially drive the formation of mutant uromodulin aggregates, impacting on kidney damage and disease progression. Enhancement of autophagy decreased uromodulin aggregates, relevant for therapeutic strategies in ADTKD.

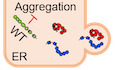

## Synopsis

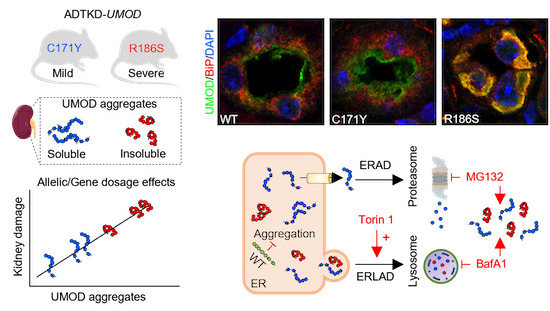

- Two Umod KI models show strong allelic and gene dosage effects on uromodulin aggregates and activation of inflammation and fibrosis, leading to variable kidney damage.
- The wild‐type Umod allele protects against the formation of uromodulin aggregates.
- Studies in kidney tubular cells support mutation‐specific effects on uromodulin aggregates and activation of quality control and clearance mechanisms.
- Enhancement of autophagy by mTORC1 inhibition decreased uromodulin aggregates.

**The paper explained**

**Problem**

Missense mutations in the *UMOD* gene that encodes the kidney‐specific protein uromodulin cause autosomal dominant tubulointerstitial kidney disease (ADTKD), one of the most common monogenic kidney diseases. Affected individuals develop chronic kidney disease (CKD) that progresses to kidney failure, with no specific treatment to alter the progression of the disease. The strong interfamilial variability in progression, the unknown consequences of mutant uromodulin accumulation, and a possible gene dosage effect leave open the mechanisms of end‐organ damage in ADTKD‐*UMOD*. A pressing need for ADTKD is to bridge the gap linking postulated gain‐of‐function mutations with clinically relevant end points.

**Results**

By combining human, mouse, and cellular studies, we show how representative missense mutations of uromodulin, associated with divergent disease severity, lead to distinct trafficking defects and propensity to form intracellular aggregates, with differential activation of ER stress and UPR, and variable end‐organ damage. The *Umod* ^C171Y/+^ mice showed a distinct protein folding chaperone response, preventing accumulation of premature uromodulin in the kidneys. Conversely, the *Umod* ^R186S/+^ mice presented an accumulation of premature uromodulin in the kidney, triggering the formation of intracellular aggregates and a pathogenic cascade. Studies in kidney cells confirmed differences in the formation of aggregates, with activation of distinct clearance mechanisms. Enhancement of autophagy by starvation and mTORC1 inhibition decreased uromodulin aggregates, suggesting a potential therapeutic strategy.

**Impact**

Overall, we demonstrate that the differential effect of specific missense *UMOD* mutations on kidney disease progression in mice and humans is due to their propensity to misfold and aggregate, overcoming the capacity to activate chaperone and folding systems. The fact that toxic uromodulin aggregates drive end‐organ disease implies that treatments should aim to decrease uromodulin aggregation and/or to promote folding capacity. As in other dominant, toxic gain‐of‐function disorders, the protective role of wild‐type uromodulin suggests the importance of allele‐specific targeting to decrease the levels of uromodulin. In addition to activators of autophagy, enhancement of specific degradative pathways may be required to promote the clearance of uromodulin mutants.

## Introduction

Autosomal dominant tubulointerstitial kidney disease (ADTKD) includes a set of rare kidney disorders characterized by tubular damage and interstitial fibrosis in the absence of glomerular lesions. Affected individuals develop chronic kidney disease (CKD) that ultimately progresses to kidney failure. There is currently no specific treatment to alter the progression of ADTKD (Devuyst *et al*, 2019). The most common gene causing ADTKD is *UMOD*, which is exclusively expressed in the kidney and encodes uromodulin, the most abundant protein excreted in the urine (Olinger *et al*, 2020). ADTKD‐*UMOD* (MIM #162000) is one of the most common monogenic kidney diseases, with a prevalence of ~2% in patients with kidney failure (Gast *et al*, 2018; Groopman *et al*, 2019).

Uromodulin is a glycosylphosphatidylinositol (GPI)‐anchored glycoprotein, produced mainly by the cells lining the thick ascending limb (TAL) of the loop of Henle (Devuyst *et al*, 2017). After the formation of 24 disulfide bridges and extensive glycosylation, uromodulin is trafficked to the apical plasma membrane, where it is cleaved and released into the tubular lumen to form high‐molecular‐weight polymers (Brunati *et al*, 2015). Uromodulin exerts important roles in tubular cells and lumen, including regulation of sodium transport (Trudu *et al*, 2013; Tokonami *et al*, 2018), inhibition of kidney stone formation (Mo *et al*, 2004a) and protection against urinary tract infections (Bates *et al*, 2004; Weiss *et al*, 2020).

More than 135 ADTKD‐*UMOD* mutations have been reported, essentially missense mutations that replace or introduce a cysteine residue (Devuyst *et al*, 2019; Olinger *et al*, 2020). Analyses of kidney biopsies from patients with ADTKD‐*UMOD* revealed accumulation of uromodulin in the endoplasmic reticulum (ER) of TAL cells, suggestive of ER storage disease (Dahan *et al*, 2003; Rampoldi *et al*, 2003; Vylet'al *et al*, 2006). Consistent with abnormal processing, a dramatic reduction in uromodulin levels in the urine was observed in individuals with ADTKD‐*UMOD* (Dahan *et al*, 2003; Bleyer & Kmoch, 2014). *In vitro* analyses confirmed that mutations in uromodulin cause defective ER to Golgi transport, with activation of the unfolded protein response (UPR) and a suggestive correlation between the severity of the trafficking defect of uromodulin mutants and the age of end‐stage kidney disease (ESKD) (Bernascone *et al*, 2006; Kidd *et al*, 2020).

Mouse models carrying *Umod* mutations have been generated using various methodologies (Kemter *et al*, 2009, 2013; Bernascone *et al*, 2010; Johnson *et al*, 2017; Piret *et al*, 2017). Although these mice recapitulate the key features of ADTKD‐*UMOD*, they are restricted by technical issues, lack of corresponding human mutations, and limited phenotype analyses. The potential role of gene dosage effects and of the wild‐type uromodulin, and the fate of mutant uromodulin are also unknown. Altogether, the limited information coming from these models and the unknown consequences of specific mutant uromodulin accumulation leave open the mechanisms of end‐organ damage and disease progression. These issues are emphasized by the strong interfamilial variability in the progression of ADTKD‐*UMOD* (Moskowitz *et al*, 2013; Olinger *et al*, 2020) and a possible gene‐dosage effect on uromodulin processing (Edwards *et al*, 2017). Wild‐type uromodulin may also play a role, analogous to other dominant, gain‐of‐toxic function disorders such as Huntington disease, where the lack of wild‐type huntingtin causes a more severe phenotype (Leavitt *et al*, 2001; Zhang *et al*, 2003).

To elucidate the gap between postulated gain‐of‐function mutations and clinically relevant endpoints, we selected two missense *UMOD* mutations representative of divergent disease progression in patients with ADTKD‐*UMOD*. We generated *Umod* knock‐in (KI) mice carrying these p.C170Y and p.R185S *UMOD* mutations and investigated the impact of allelic and gene dosage effects on the formation of intracellular aggregates, the role of wild‐type uromodulin and the downstream signaling pathways leading to CKD. Analyses in stably transduced kidney tubular cells revealed distinct ER accumulation of premature mutant uromodulin and activation of the ER stress response, with the formation of biochemically distinct aggregates and differential activation of clearing mechanisms. These studies decipher the pathways linking the formation of intracellular uromodulin aggregates, unfolded protein response and kidney damage in ADTKD, with relevance for therapeutic strategies and for toxic gain‐of‐function mechanisms in autosomal dominant diseases.

## Results

### Identification of *UMOD* mutations associated with divergent ADTKD progression

**Figure 1:**
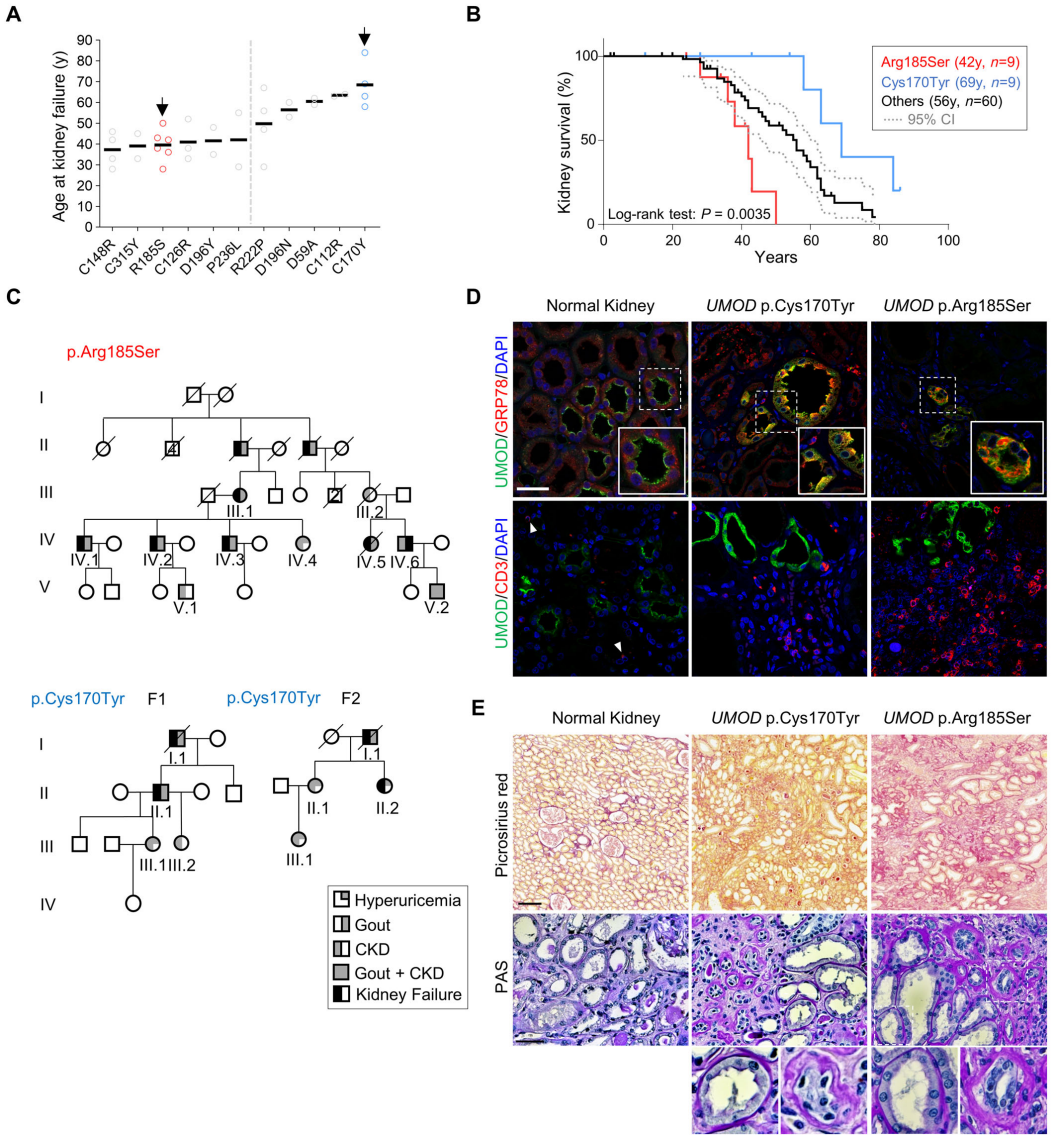
Mutations associated with divergent progression of ADTKD‐*UMOD* Age at onset of kidney failure for ADTKD‐*UMOD* patients with the indicated *UMOD* mutations. Only mutations with at least 2 individuals reaching kidney failure at documented age are represented here. Two clusters of *UMOD* mutations associated with earlier‐onset and later‐onset kidney failure with two cluster‐representative mutations (arrows, see Results text) are highlighted.Kaplan–Meier curve of kidney survival in patients with the *UMOD* p.Arg185Ser mutation (*n* = 9, median age at kidney failure: 42 years), the *UMOD* p.Cys170Tyr mutation (*n* = 9, median age at kidney failure: 69 years) and 60 ADTKD‐*UMOD* patients from the Belgo‐Swiss registry (see Material & Methods) with 24 different *UMOD* mutations (median age at kidney failure: 56 years). A log‐rank test was used for comparison of survival curves. Dotted lines indicate 95% confidence interval.Pedigrees of three multiplex families with ADTKD in which representative *UMOD* mutations p.Arg185Ser and p.Cys170Tyr have been identified. Females are represented by circles and males by squares, and phenotypes are denoted as indicated. Clinical features are detailed for each patient in Appendix Tables S1 and S2.Representative confocal analysis of uromodulin (UMOD, green), GRP78/BiP or CD3 (red) of kidney nephrectomy samples from ADTKD‐*UMOD* patients (p.Cys170Tyr – F1, II.1; p.Arg185Ser – IV.5). Arrowheads indicate CD3^+^ cells. Nuclei were counterstained with DAPI (blue). Scale bar: 25 μm.Picrosirius Red and periodic acid‐Schiff (PAS) stainings of kidney nephrectomy samples from ADTKD‐*UMOD* patients (p.Cys170Tyr – F1, II.1; p.Arg185Ser – IV.5). Squares indicate images shown at higher magnification. Scale bars: 100 μm for Picrosirius red; 50 μm for PAS. Source data are available online for this figure.

We characterized missense mutations associated with divergent severity of ADTKD‐*UMOD* as a first step to dissect properties of uromodulin mutants and pathways driving kidney disease. A review of 12 missense *UMOD* mutations detected in at least two genetically confirmed ADTKD‐*UMOD* individuals reaching kidney failure in the Belgo‐Swiss ADTKD Registry (Olinger *et al*, 2020), confirmed a strong influence of the mutation on age at kidney failure (test for linear trend: *P* < 0.0001) (Fig 1A). Two clusters of mutations associated with earlier (before ~40 years) and later (50–69 years) onset of kidney failure were identified. The representative mutations, p.Arg185Ser (earlier‐onset) and p.Cys170Tyr (later‐onset), were selected since they: (i) were the most prevalent in both clusters; (ii) involved cysteine and non‐cysteine residues; and (iii) were detected in ADTKD‐*UMOD* families with available kidney biopsy. Patients with the *UMOD* p.Arg185Ser mutation showed a much faster disease progression than those with the p.Cys170Tyr mutation (*N* = 9 in each group; median age of kidney failure: 42 years vs. 69 years), with a similar proportion of males and females reaching ESKD (Appendix Tables S1 and S2). The rapid progression associated with the p.Arg185Ser mutation was clearly distinct from the aggregated data for 60 ADTKD patients harboring 22 other *UMOD* mutations in the registry (median age of kidney failure: 56 years; log rank test: *P* = 0.0035; Fig 1B). Both mutations segregated in typical multiplex ADTKD families and were found to be (likely) pathogenic by *in silico* analyses (Fig 1C; Appendix Tables S1–S3).

Examination of kidney biopsies revealed that the p.Arg185Ser mutation was associated with intracellular mutant uromodulin accumulation, and increased levels of the ER stress marker GRP78/BiP, a key regulator of the unfolded protein response (UPR). These modifications were also observed, to a lesser extent, in the p.Cys170Tyr biopsy (Fig 1D).

Furthermore, a high number of CD3^+^ inflammatory cells (lymphocytes) was observed in the p.Arg185Ser biopsy compared to the p.Cys170Tyr specimen (Fig 1D). Histological analysis using Picrosirius red, a well‐established staining for collagen fibers, evidenced strong interstitial fibrosis in the p.Arg185Ser biopsy, less pronounced in the p.Cys170Tyr specimen (Fig 1E). Periodic acid‐Schiff (PAS) staining showed thickening and fragmentation of the tubular basement membrane in TALs from both biopsies (Fig 1E), a feature long associated with ADTKD (Devuyst *et al*, 2019).

Thus, based on kidney failure, we identified two *UMOD* missense mutations representative of rapid‐ and slow‐progressing ADTKD‐*UMOD* with potentially divergent pathomechanisms, as suggested from multi‐level kidney tissue analysis.

### *Umod* KI mice show allelic and gene dosage effects on uromodulin processing and kidney damage

**Figure 2:**
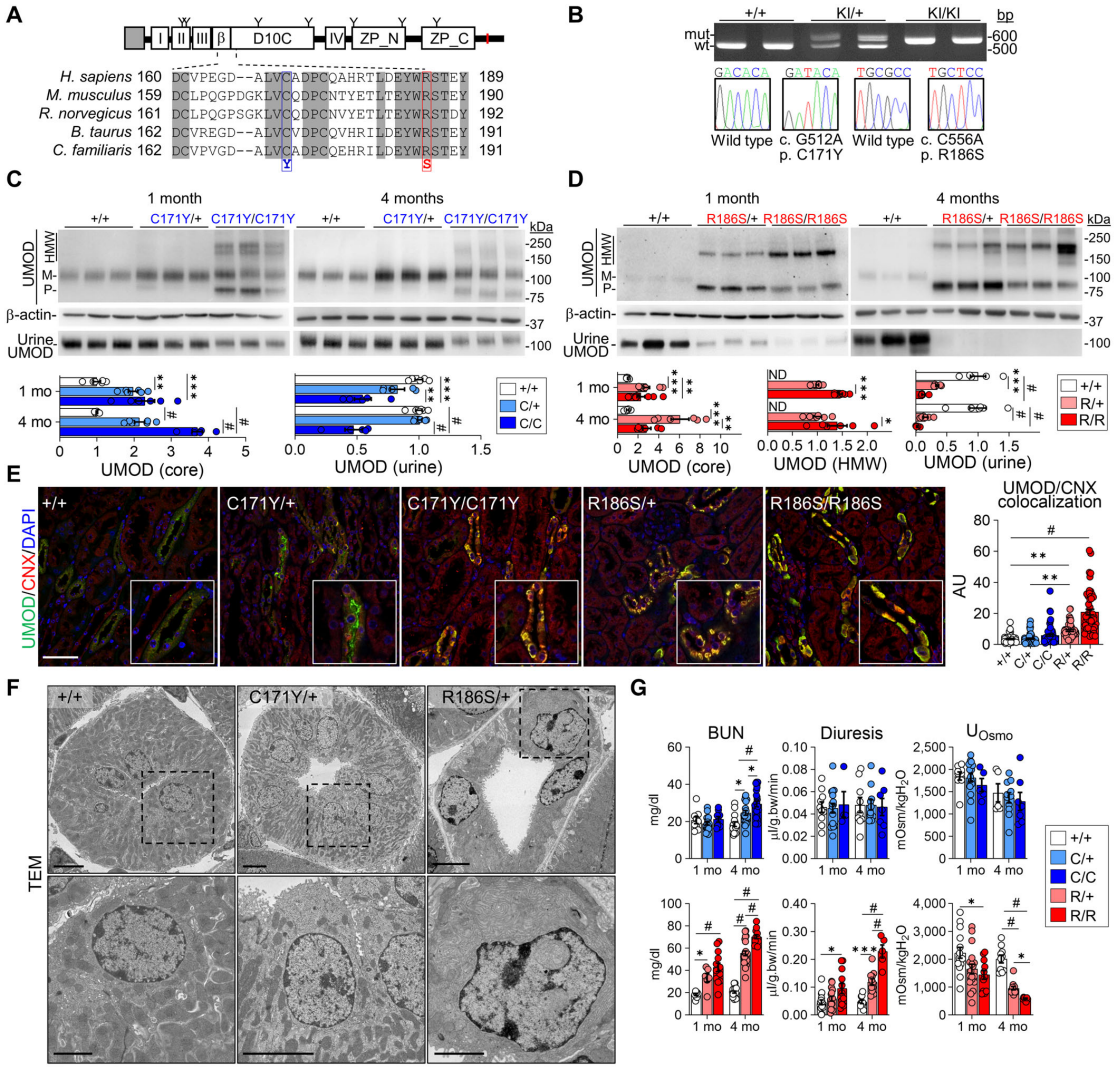
Characterization of the *Umod* ^C171Y^ and *Umod* ^R186S^ mouse models AUromodulin (UMOD) domain architecture including a signal peptide (gray box), four EGF‐like domains (I, II, III, IV), a beta‐hairpin (β), a cysteine‐rich domain (D10C) and a bipartite zona pellucida domain (ZP_N, ZP_C). The GPI‐anchoring site (red line) and glycosylation sites (Y) are indicated. Alignment of uromodulin amino acid sequences in different mammalian species is shown, with identity regions displayed as shadowed. Mutations of interest are indicated in red (p.R186S) and blue (p.C171Y).BGenotyping and sequencing chromatogram of *Umod* KI mice, showing the regions surrounding the two mutations (c.C512, c.C556) in *Umod*.C, DImmunoblot analysis of kidney and urine UMOD in 1‐ and 4‐month‐old *Umod* ^C171Y^ (C) and *Umod* ^R186S^ (D) mice. For kidney lysates, β‐actin was used as a loading control. Urine samples were normalized to creatinine concentration (*n* = 5–9 animals per group). Densitometry analysis is relative to *Umod* ^+/+^ for core and to *Umod* ^R186S/+^ for HMW aggregates. M: mature; P: precursor; HMW: high molecular weight. The mature and precursor UMOD are collectively referred to as the core. (C) UMOD (core), 1 mo: **P = 0.0034, ***P = 0.0003; UMOD (urine), 1 mo: **P = 0.0084, ***P = 0.0002; ^#^ *P* < 0.0001. (D) UMOD (core), 1 mo: ***P = 0.0002, **P = 0.0083; 4 mo: ***P = 0.0001, **P = 0.0038. UMOD (HMW), 1 mo: ***P = 0.0007; 4 mo: *P = 0.0158. UMOD (urine), 1 mo: ***P = 0.0001; ^#^ *P* < 0.0001.ERepresentative immunofluorescence analysis of UMOD (green) and calnexin (CNX, red) in kidney sections from 4‐month‐old mice (*n* = 47–70 tubules per group). Nuclei are counterstained with DAPI (blue). Scale bar: 25 μm. **P(+/+ vs. R/+) = 0.0012, **P(C/+ vs. R/+) = 0.0011, ^#^ *P* < 0.0001.FTransmission electron microscopy (TEM) of kidney sections from 3‐month‐old *Umod* mice, showing progressive ER expansion and hyperplasia. Squares indicate images shown at higher magnification. Scale bar: 5 μm for low magnification, 2 μm for high magnification.GMain clinical parameters of *Umod* ^C171Y^ and *Umod* ^R186S^ mice at 1 and 4 months (*n* = 4–24 animals per group). C171Y‐BUN, 4 mo: *P(+/+ vs. C/+) = 0.0181, *P(C/+ vs. C/C) = 0.0367; R186S‐BUN, 1 mo: *P = 0.0373; Diuresis, 1 mo: *P = 0.0147; 4 mo: ***P = 0.0002. U_Osmo_, 1 mo: *P = 0.0153; 4 mo: *P = 0.03; ^#^ *P* < 0.0001. Data information: Bars indicate the mean ± SEM. One‐way ANOVA with Tukey's *post hoc* test. Source data are available online for this figure.

Based on the representative *UMOD* mutations (C170Y and R185S), we generated two KI mouse models harboring the equivalent *Umod* mutations (C171Y and R186S; Fig 2A and B). These *Umod* KI mice were born in the expected Mendelian ratio and were viable and fertile.

We first verified whether the *Umod* mutations were reflected by maturation and trafficking defects (Fig 2C and D). The mature, fully glycosylated uromodulin band (~100 kDa) was detected with variable intensity in *Umod* ^C171Y^ kidneys (Fig 2C). In homozygous *Umod* ^C171Y/C171Y^ kidneys, the uromodulin signal included the mature form and additional bands at ~80 kDa, potentially corresponding to immature isoforms carrying ER‐type glycans, and high‐molecular‐weight (HMW, ~160–200 kDa) bands, compatible with uromodulin aggregates (Fig 2C). In contrast, the ~100 kDa band corresponding to mature uromodulin was not detected in the *Umod* ^R186S^ kidneys, whereas strong signals for immature (~80 kDa) and HMW aggregate forms of uromodulin were detected at 1 month of age, with higher levels observed in homozygotes (Fig 2D). Treatment with endoglycosidase H (Endo H) or peptide:N‐glycosidase F (PNGase F) confirmed the presence of immature forms of uromodulin carrying ER‐type glycosylation in both homozygous *Umod* ^C171Y^ and *Umod* ^R186S^ kidney samples (Appendix Fig S1A and B). The changes in uromodulin maturation impacted its urinary excretion: striking reductions were observed in *Umod* ^R186S^ mice (Fig 2D), whereas urinary levels were unchanged in *Umod* ^C171Y/+^ mice, being only decreased in the *Umod* ^C171Y/C171Y^ mice (Fig 2C).

We next analyzed whether specific mutations affect the distribution of uromodulin in the *Umod* KI kidneys. Dual labeling with calnexin showed a mild ER retention of uromodulin with residual apical signal in *Umod* ^C171Y^ kidneys, contrasting with a major ER retention of uromodulin and lack of apical signal in the *Umod* ^R186S^ kidneys (Fig 2E), mimicking the human situation (Fig 1D). At the subcellular level, the massive accumulation of uromodulin caused expansion and hyperplasia of the ER in *Umod* ^R186S/+^ kidneys, as shown by electron microscopy (Fig 2F) and correlative light‐electron microscopy (CLEM) analysis (Appendix Fig S1C). The intracellular uromodulin aggregates in *Umod* ^R186S/+^ kidneys were already observed at 2 weeks of age (Appendix Fig S2A–C), with only faint signals for mature uromodulin (Appendix Fig S2B) and apical staining in TAL cells (Appendix Fig S2C) in these samples.

To test whether the changes in uromodulin processing affected disease progression, we analyzed the plasma and urine biochemistry of *Umod* KI mice compared to wild‐type control littermates. The *Umod* ^C171Y^ mice showed a progressive increase in blood urea nitrogen (BUN), with higher levels in homozygotes at 4 months, whereas the *Umod* ^R186S^ mice showed significantly increased BUN levels at 1 month, with a strong urinary concentration defect, reduced fractional excretion of uric acid, and inappropriate urinary calcium excretion (Fig 2G; Appendix Tables S4 and S5).

Collectively, these data indicate that the two *Umod* KI lines present typical manifestations of ADTKD‐*UMOD*, with allelic and gene‐dosage effects on uromodulin processing and severity of kidney damage, in line with observations in humans.

### Distinct mutational effects on the properties of uromodulin aggregates

**Figure 3:**
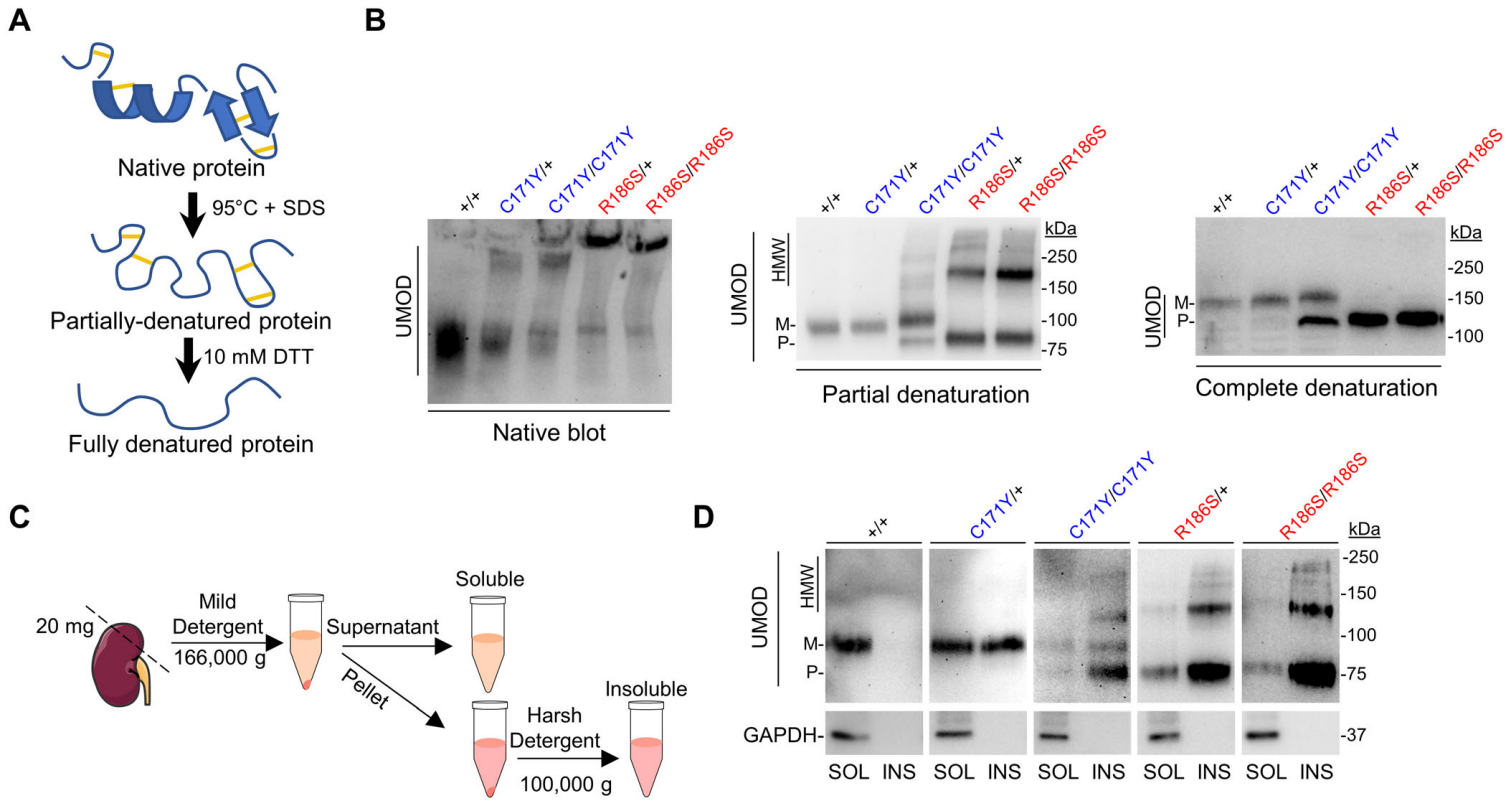
Biochemical profiling of high molecular weight uromodulin aggregates Schematic representation of the partial and total protein denaturation treatments.Immunoblot analysis of kidney lysates from *Umod* KI mice in native (left), partially denatured (center) and completely denatured (right) conditions, showing the sample treatment‐dependent effect on uromodulin (UMOD) migration. Experiment performed in technical triplicate, with three biological replicates per genotype.Schematic representation of the solubility assay protocol.Solubility assay of UMOD in kidney lysates from 4‐month‐old *Umod* KI mice. GAPDH was used as a purity marker for the insoluble fraction. Source data are available online for this figure.

To test the impact of the biochemical nature of uromodulin aggregates on the observed phenotypes, we first analyzed kidney uromodulin in its native form and subsequently performed partial and total protein denaturation (Fig 3A). Under native conditions, *Umod* ^C171Y^ lysates showed an additional higher band, which was not observed in *Umod* ^+/+^ kidneys. Moreover, part of the uromodulin from *Umod* ^C171Y/C171Y^ and most in *Umod* ^R186S^ lysates migrated even less efficiently (Fig 3B). Following partial denaturation, *Umod* ^C171Y/+^ kidney lysates showed only the band corresponding to mature uromodulin, suggesting that aggregates in these samples were mediated by weak interactions. In contrast, HMW bands were still detected in *Umod* ^C171Y/C171Y^ and *Umod* ^R186S^ kidneys, which could only be resolved when using reducing conditions, suggesting the involvement of intermolecular disulfide bridges (Fig 3B). The solubility of uromodulin aggregates was assessed using a soluble/insoluble fractionation protocol (Fig 3C): wild‐type uromodulin was exclusively localized in the soluble fraction, whereas premature forms and HMW uromodulin aggregates were mainly in insoluble fractions (Fig 3D). Lysates from both homozygous mutant kidneys showed an enriched signal in the insoluble fraction compared to heterozygotes. Although no signal for premature or HMW uromodulin was detected in *Umod* ^C171Y/+^ kidneys, mature uromodulin was detected in both soluble and insoluble fractions. This suggests that p.C171Y uromodulin is characterized by a partially tolerated folding defect, still undergoing complete maturation. However, once the mutant protein reaches the apical plasma membrane, misfolding may reoccur, causing increased retention.

These data show that both weak and strong interactions may participate in the formation of aberrant uromodulin aggregates, whose biochemical properties strongly depend on the mutation involved.

### Wild‐type uromodulin protects against mutant uromodulin aggregation

**Figure 4:**
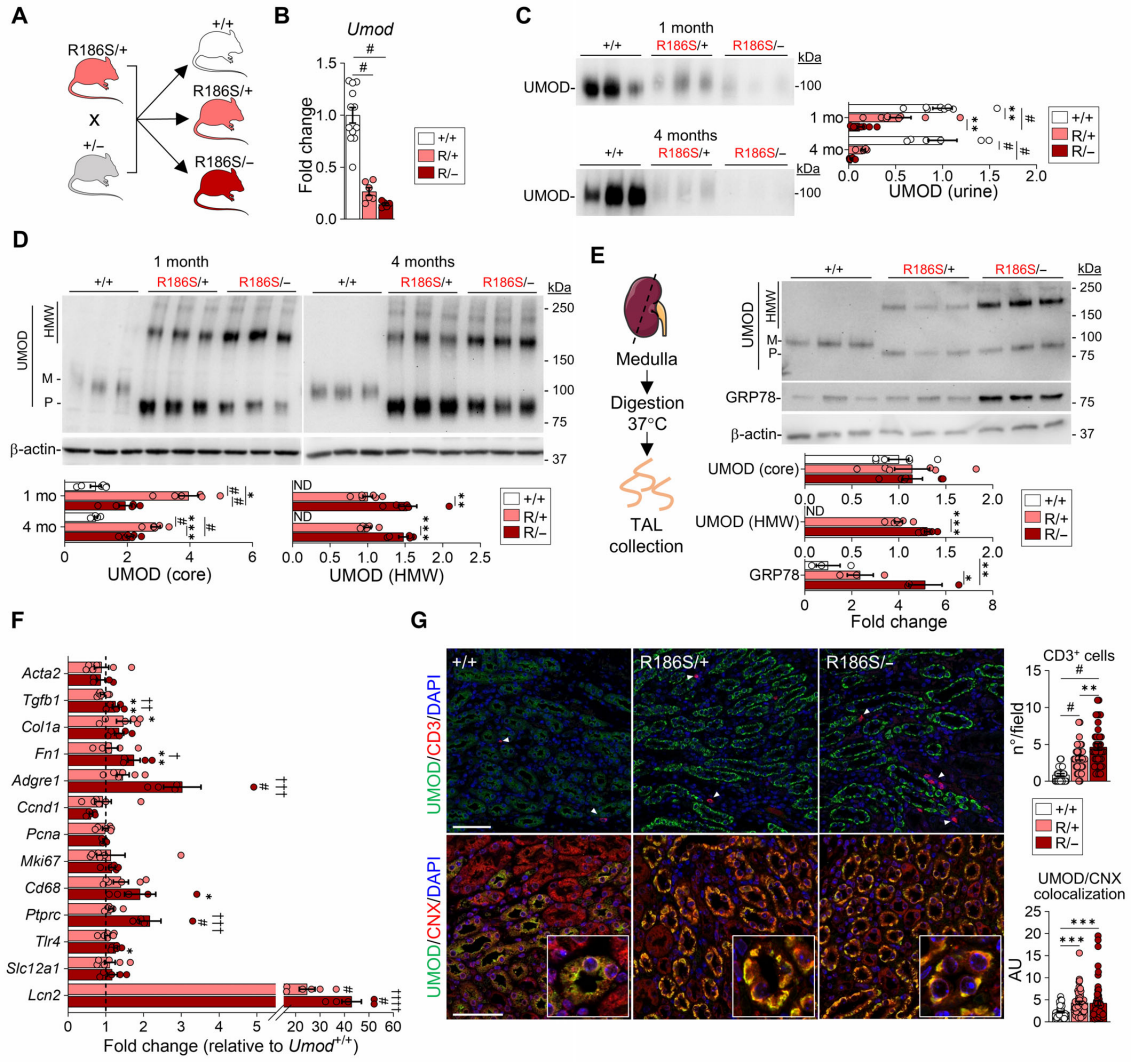
Deletion of wild‐type uromodulin increases mutant uromodulin aggregation AStrategy for the generation of *Umod* ^R186S/−^ mice.B
*Umod* transcript levels evaluated by RT–qPCR on total kidney extracts from 1‐month *Umod* ^R186S/+^, *Umod* ^R186S/*−*^, and *Umod* ^+/+^ mice (*n* = 5–12 animals per group), ^#^ *P* < 0.0001.CImmunoblot analysis of urine uromodulin (UMOD) in 1‐ and 4‐month *Umod* ^R186S/*−*^ mice. Urine was loaded according to creatinine concentration (*n =* 5–9 animals per group). 1 mo: **P(+/+ vs. R/+) = 0.0045, **P(R/+ vs. R/−) = 0.005; ^#^ *P* < 0.0001.D, ERepresentative immunoblot analysis of UMOD in whole kidney at 1 month or 4 months (*n =* 5–6 animals per group) (D) or of UMOD and GRP78 in isolated TAL (*n* = 3–6 TAL fractions per group). (E). β‐actin was used as a loading control. Densitometry analysis is relative to *Umod* ^+/+^ for core and to *Umod* ^R186S/+^ for HMW aggregates. M: mature; P: precursor; HMW: high molecular weight. The mature and precursor UMOD are collectively referred to as the core. (D) UMOD (core), 1 mo: *P = 0.0323; 4 mo: ***P = 0.0001; UMOD (HMW), 1 mo: **P = 0.0031; 4 mo: ***P = 0.0002; ^#^ *P* < 0.0001. (E) UMOD (HMW): ***P = 0.0001. GRP78: *P = 0.0388, **P = 0.0065.FTranscript levels of inflammation, proliferation, fibrotic markers and *Slc12a1* as internal control assessed by RT–qPCR on total kidney extracts from mice at 1 month (*n* = 5–12 animals per group). *Tgfb1*: **P(+/+ vs. R/−) = 0.049, ^††^ *P*(R/+ vs. R/−) = 0.001; *Col1a1*: *P(+/+ vs. R/+) = 0.0377; *Fn1*: **P(+/+ vs. R/−) = 0.0035, ^†^ *P*(R/+ vs. R/−) = 0.0398; *Adgre1*: ^†††^ *P*(R/+ vs. R/−) = 0.0007; *Cd68*: *P(+/+ vs. R/−) = 0.0118; *Ptprc*: ^†††^ *P*(R/+ vs. R/−) = 0.0002; *Tlr4*: *P(+/+ vs. R/+) = 0.0146; ^#^ *P*(vs. +/+) < 0.0001, ^††††^ *P*(R/+ vs. R/−) < 0.0001.GRepresentative immunofluorescence analysis of UMOD (green) and CD3 or calnexin (CNX, red) in kidney sections from 1‐month‐old mice. *N* = 40 fields from four kidneys per condition (up), *n* ≥ 57 tubules from three kidneys per condition (down). Arrowheads indicate CD3^+^ cells. Nuclei are stained with DAPI (blue). Scale bar: 50 μm. CD3^+^ cells: **P = 0.0034, ^#^ *P* < 0.0001; UMOD/CNX: ***P(+/+ vs. R/+) = 0.0007, ***P(+/+ vs. R/−) = 0.0008. Data information: Bars indicate the mean ± SEM. One‐way ANOVA with Tukey's *post hoc* test. Source data are available online for this figure.

Given the gene‐dosage effects and the early‐onset disease in *Umod* ^R186S^ mice, we wondered whether the increased severity observed in *Umod* ^R186S/R186S^ mice was solely due to an increased load of mutant uromodulin, or whether the wild‐type allele may attenuate the phenotype. To this end, we crossed *Umod* ^R186S/+^ with *Umod* ^*+*/*−*^ mice to obtain *Umod* ^R186S/*−*^ mice, with the expected decrease in *Umod* mRNA (Fig 4A and B) and in the urinary levels of uromodulin (Fig 4C). Despite the decreased global expression of uromodulin between *Umod* ^R186S/*−*^ and *Umod* ^R186S/+^ mice, a significant increase in HMW aggregates and a pronounced reduction in premature uromodulin were observed in *Umod* ^R186S/*−*^ compared to *Umod* ^R186S/+^ kidneys (Fig 4D). The parallel increase of HMW aggregates and the ER chaperone GRP78 was confirmed in isolated TAL segments from *Umod* ^R186S^ kidneys (Fig 4E). The impact of HMW uromodulin accumulation in the *Umod* ^R186S/*−*^ kidneys was further evidenced by a strong upregulation in inflammation (*Adgre1*, *Cd68*, *Ptrpc*, *Tlr4*), fibrosis (*Fn1*, *Tgfb*1) and tubular damage (*Lcn2*) markers compared to *Umod* ^+/+^ and *Umod* ^R186S/+^ kidneys (Fig 4F), and by larger numbers of CD3^+^ cells close to uromodulin‐positive TAL segments (Fig 4G). Of note, these changes in *Umod* ^R186S/*−*^ compared to *Umod* ^R186S/+^ mice were not reflected in terms of ER retention of uromodulin (Fig 4G) or by clinical and biological parameters (Appendix Table S6). Globally, the analysis of gender‐split animals did not highlight any major effect of sex on disease progression on any genotype (Appendix Table S7).

### Differential activation of the UPR and kidney damage pathways by mutant uromodulin

**Figure 5:**
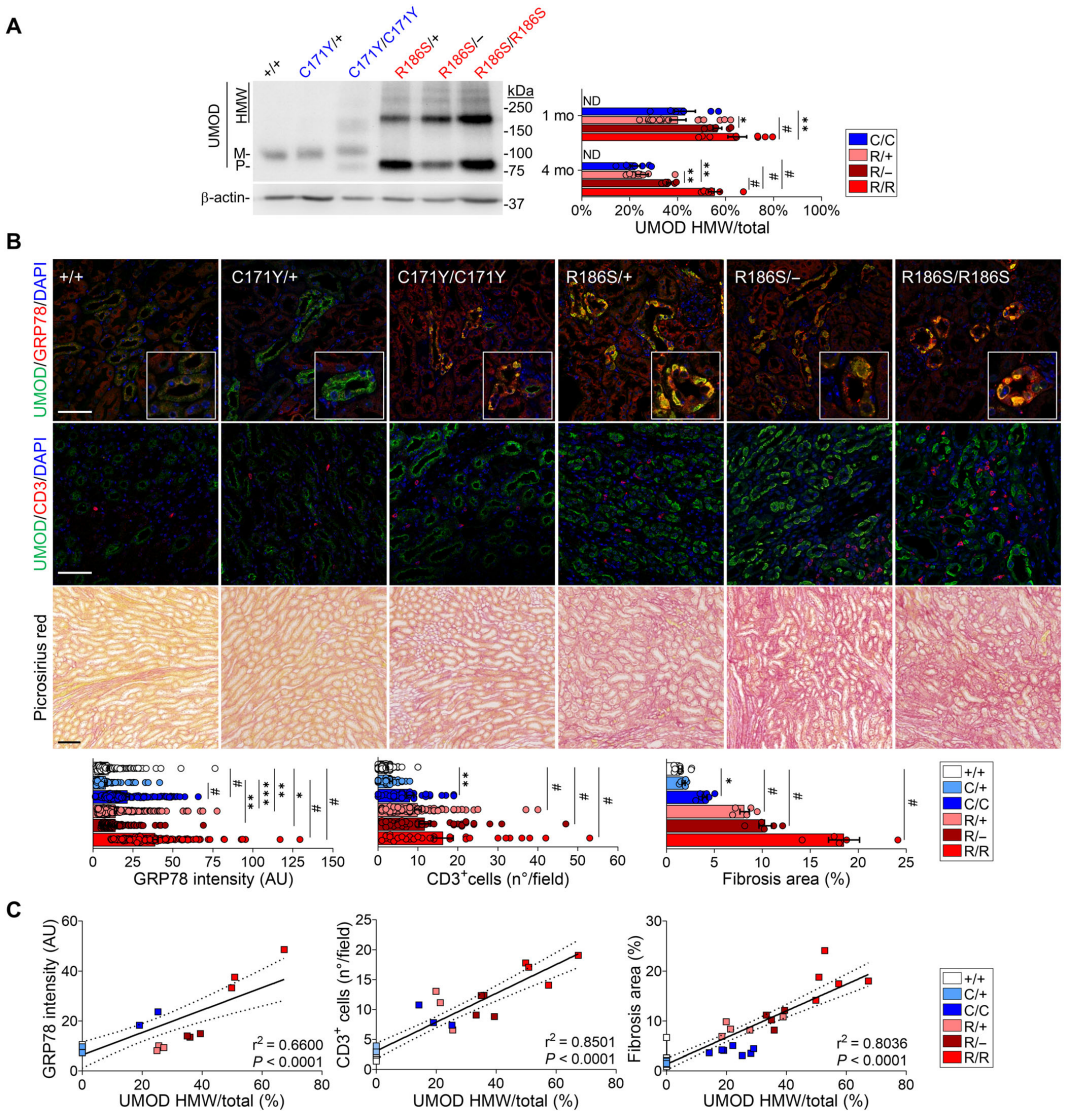
Mutant uromodulin aggregates drive severity of ADTKD‐*UMOD* in mouse models Representative immunoblots for uromodulin (UMOD) in kidneys from 1‐month wild‐type and mutant mice, showing distinct patterns for mature, precursor and HMW bands. β‐actin was used as a loading control (*n =* 6–15 animals per group). M: mature; P: precursor; HMW: high molecular weight. The mature and precursor UMOD are collectively referred to as the core. HMW aggregates are expressed as ratio over total UMOD (core + HMW). 1 mo: *P = 0.0252, **P = 0.0051; 4 mo: **P(C/C vs. R/−) = 0.001, **P(R/+ vs. R/−) = 0.0081; ^#^ *P* < 0.0001.Immunofluorescence analysis of UMOD and GRP78 (top) or CD3 (middle) and picrosirius red staining on kidney sections from 4‐month‐old *Umod* KI mice (*n* = 3–11 animals per group). Scale bar: 25 μm for immunofluorescence, 50 μm for picrosirius red. GRP78: **P(+/+ vs. R/+) = 0.0024, **P(C/+ vs. R/−) = 0.0019, *P = 0.0223, ***P = 0.0002; CD3^+^: **P = 0.0051; Fibrosis: *P = 0.0154; ^#^ *P* < 0.0001.Linear regression (black line) illustrating the correlation between UMOD aggregates and GRP78 intensity (left), CD3^+^ infiltrates (middle) and interstitial fibrosis (right) in 4‐month‐old *Umod* KI mice. The dotted lines show the 95% confidence intervals. Dots represent individual animals. The equations for the curves are *y* = 0.4501*x* + 6.350 (left), *y* = 0.2414*x* + 3.063 (middle), and *y* = 0.2668*x* + 1.342 (right). Data information: Bars indicate the mean ± SEM. One‐way ANOVA with Tukey's *post hoc* analysis. Source data are available online for this figure.

The striking differences in the amount of HMW uromodulin aggregates in the kidneys of the distinct *Umod* mouse lines (Fig 5A) led us to investigate their differential effects on the ER and UPR stress pathways. The signal for the UPR gatekeeper protein GRP78/BiP was low in the (uromodulin‐positive) TAL segments of *Umod* ^+/+^ and *Umod* ^C171Y/+^ mice, reflecting normal uromodulin processing, but strikingly increased in *Umod* ^C171Y/C171Y^, *Umod* ^R186S/+^, *Umod* ^R186S/−^ and *Umod* ^R186S/R186S^ kidneys, consistent with uromodulin accumulation in the ER (Fig 5B). Immunoblot analyses showed that uromodulin storage upregulated the PERK (PERK, ATF4) and IRE1α branches of the UPR in *Umod* ^R186S^ (Appendix Fig S3A, C and D) but not in *Umod* ^C171Y^ kidneys (Appendix Fig S3B). Importantly, activation of PERK did not trigger apoptosis, as indicated by the lack of TUNEL‐positive cells or increased cleavage of caspase‐3 in the *Umod* ^R186S^ kidneys (Appendix Fig S4). These results indicate that the ER accumulation of specific mutant uromodulin triggers the UPR based on activation of the PERK and IRE1α branches.

Importantly, the different *UMOD* mutations induced distinct expression patterns for selective autophagy of the ER (ER‐phagy) genes involved in protein quality control mechanisms. In particular, the expression levels of *Rtn3*, *Sec62*, and *Ccpg1* were significantly higher in *Umod* ^C171Y^ kidneys than in *Umod* ^R186S^ kidneys, while *Retreg1* was downregulated in both *Umod* ^R186S^ and *Umod* ^C171Y/C171Y^ kidneys containing uromodulin aggregates (Appendix Fig S5A). The activation of cellular degradation pathways to mitigate misfolded protein toxicity was evidenced by the upregulation of the ubiquitin‐binding protein SQSTM‐1/p62 and the autophagic marker regulating LC3 lipidation ATG5 in *Umod* KI kidneys that paralleled the allelic and gene dosage effects on aggregate formation (Appendix Figs S5B and S6).

Tubulointerstitial damage and fibrosis characterize end‐organ damage in ADTKD‐*UMOD*. Analyses in age‐ and gender‐matched *Umod* lines revealed that uromodulin accumulation was paralleled by a progressive infiltration of CD3^+^ and F4/80^+^ cells (macrophages) and the onset of fibrosis, evaluated by Picrosirius red and Masson's trichrome, starting around the cortical TALs and later expanding to the outer medulla (Fig 5B; Appendix Figs S7 and S8). Quantitative analyses indicated tight correlations between the amount of uromodulin aggregates and the levels of GRP78 upregulation, the number of CD3^+^ cells and the extent of interstitial fibrosis in the mutant *Umod* kidneys, following R186S > C171Y > WT (Fig 5C). Together, these data substantiate the role of uromodulin aggregates in driving interstitial inflammation and fibrosis in ADTKD‐*UMOD*.

### Mutant uromodulin triggers distinct pathways impacting disease progression

Based on the strong allelic effects on the ER accumulation of uromodulin, formation of aggregates, and generation of ER stress, reflected by divergent disease progression, we obtained a global view of the dynamic (mal)adaptive pathways operating in the mutant *Umod* kidneys. RNA‐sequencing (RNA‐seq) of whole‐kidney lysates revealed a strong clustering of the *Umod* ^R186S/+^ samples, with a marked effect of age, in contrast with a closer proximity between the *Umod* ^+/+^ and *Umod* ^C171Y/+^ samples (Appendix Fig S9A and B).

Compared to *Umod* ^+/+^, the *Umod* ^R186S/+^ kidneys showed an early (1 month) signature of ER stress response, increased inflammation and downregulation of lipid metabolic pathways (Appendix Fig S9C and D; Appendix Table S8), with a later (4 months) increase in immune response and activation of profibrotic pathways (Appendix Fig S10A and B; Appendix Table S9). Transcripts of TAL‐enriched genes (*Umod*, *Car3*, *Egf*) were massively downregulated in the *Umod* ^R186S/+^ kidneys. In contrast, the transcriptomic profile of *Umod* ^C171Y/+^ kidneys was virtually indistinguishable from that of *Umod* ^+/+^ kidneys at 1 month, with only subtle changes, e.g., in transcriptional regulation (*Jun*, *Btg2*, *Ier2*, *Fos*), observed at 4 months (Appendix Fig S10C–E; Appendix Table S10).

**Figure 6:**
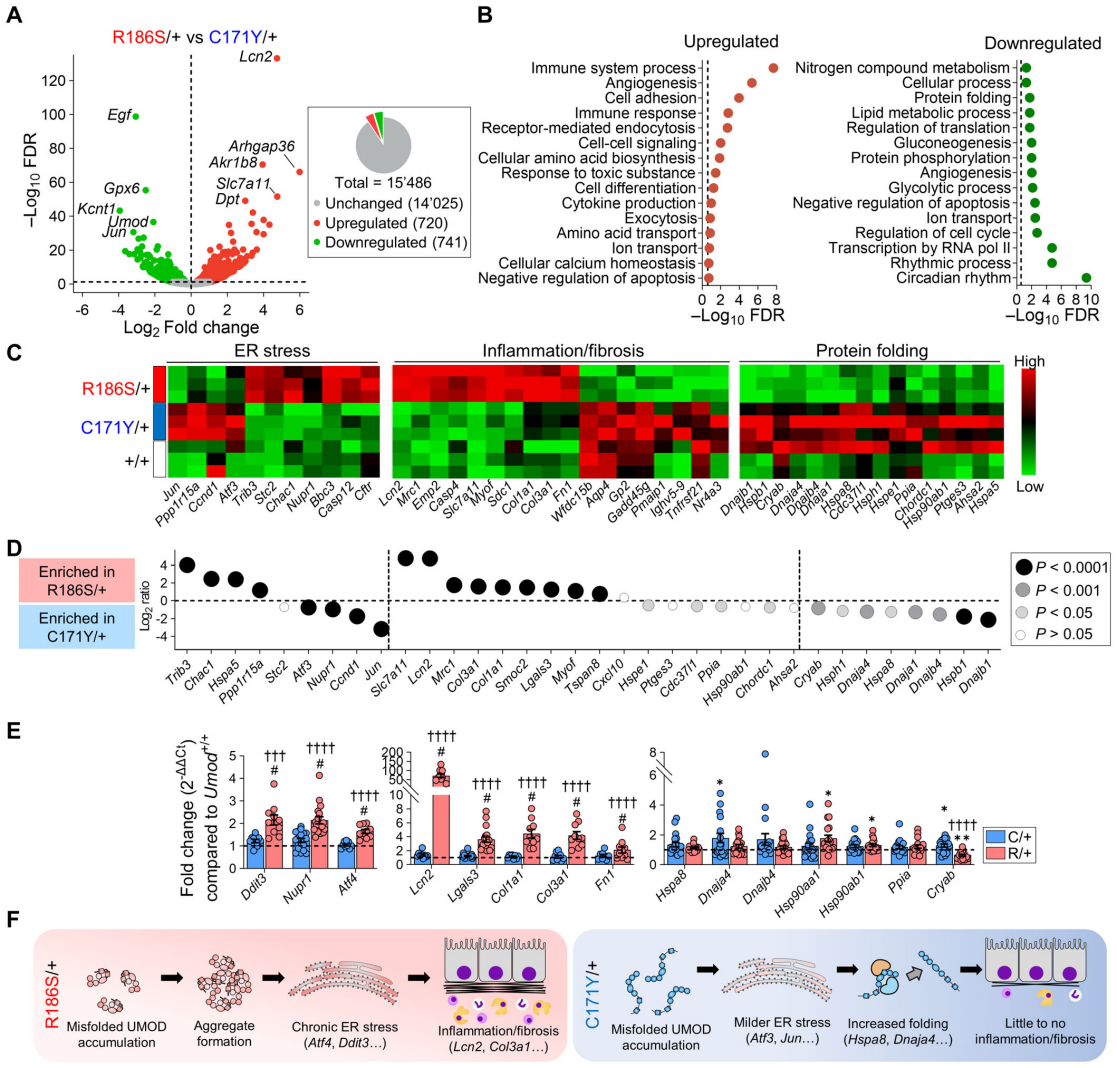
Distinct pathways impacting disease progression are activated in *Umod* KI kidneys Volcano plot showing differentially expressed genes (DEGs) between *Umod* ^C171Y/+^ and *Umod* ^R186S/+^ kidneys at 4 months. Genes not significantly changed (*FDR* >0.05) are shown in gray, whereas genes that are up‐ or downregulated in *Umod* ^R186S/+^ compared to *Umod* ^C171Y/+^ are shown in red and green, respectively. The total numbers of unchanged, up‐ and downregulated genes are summarized in the pie chart.Pathways of biological processes in gene ontology that are up‐ and downregulated in *Umod* ^R186S/+^ compared to *Umod* ^C171Y/+^.Heat map of selected, divergent pathways involved in disease progression of *Umod* KI mice at 4 months.Bubble plot of selected genes for key pathways, showing distinct signatures in the *Umod* ^C171Y/+^ and *Umod* ^R186S/+^ kidneys.Validation of selected targets by RT–qPCR on 4‐month kidneys (*n* = 9–19 animals per group). Values are relative to *Umod* ^+/+^ (black dotted line). Bars indicate the mean ± SEM. One‐way ANOVA with Tukey's *post hoc* test. *Ddit3*: ^†††^ *P*(C/+ vs. R/+) = 0.001; *Dnaja4*: *P(vs. +/+) = 0.0437; *Hsp90aa1*: *P(vs. +/+) = 0.0214; *Hsp90ab1*: *P(vs. +/+) = 0.0494; *Cryab*: *P(vs. +/+) = 0.0490, **P(vs. +/+) = 0.0037; ^#^ *P*(vs. +/+) < 0.0001, ^††††^ *P*(C/+ vs. R/+) < 0.0001.Schematic representation of pathophysiological mechanisms in *Umod* ^R186S^ (left) and *Umod* ^C171Y^ kidneys (right). Source data are available online for this figure.

Comparison of the RNA profiles in *Umod* ^R186S/+^ vs. *Umod* ^C171Y/+^ kidneys gave insight into the differential responses operating in the mutant kidneys. At 1 month (776 DEGs), activation of ER stress markers (*Trib3*, *Stc2*, *Nupr1*) and immune response (*Lcn2*, *Cxcl10*, *Lgals3*), with dysregulation of lipid metabolism (e.g., *Acat3*, *Acox2*) and ion transport (*Kcnt1*, *Clcnka*, *Slc5a3*), were noted in *Umod* ^R186S/+^ vs. *Umod* ^C171Y/+^ kidneys (Appendix Fig S11A and B; Appendix Table S11). At 4 months (1,461 DEGs), increased inflammation (*Lcn2*, *Dpt*, *Slc7a11*) and decreased TAL transcripts (*Umod*, *Egf*, *Kcnt1*) were observed in *Umod* ^R186S/+^ vs. *Umod* ^C171Y/+^ kidneys, whereas an enrichment of genes involved in transcriptional regulation (*Jun*, *Fos*, *Ier3*) or associated with protein folding (*Dnajb1*, *Hspb1*, *Cryab*, *Dnaja4*, *Hsp90ab1*) was observed in *Umod* ^C171Y/+^ vs. *Umod* ^R186S/+^ kidneys (Fig 6A and B; Appendix Table S12). These findings were confirmed by gene set enrichment analysis (GSEA), with enrichment of protein folding observed in *Umod* ^C171Y/+^ and increased immune response in *Umod* ^R86S/+^. An enrichment of Skp1/Cul1/F‐box protein (SCF)‐dependent proteasomal ubiquitin‐dependent protein catabolism was observed in 4‐month *Umod* ^C171Y/+^ compared to *Umod* ^+/+^. The activation of different ER stress response effectors, with prominent inflammation/fibrosis in *Umod* ^R186S/+^ and protein folding enrichment in *Umod* ^C171Y/+^ (Fig 6C and D), was validated by RT–qPCR analysis (Fig 6E).

Together, these signatures indicate that *Umod* ^R186S/+^ mice recapitulate typical/severe ADTKD‐*UMOD*, with induction of ER stress response and development of inflammation and fibrosis. In contrast, *Umod* ^C171Y/+^ kidneys show a milder, delayed activation of ER stress and increased expression of protein folding chaperones that presumably favor the processing and trafficking of uromodulin, resulting in less inflammation and fibrosis (Fig 6F).

### *UMOD‐GFP* cells recapitulate uromodulin trafficking defects and aggregates and activate specific quality control pathways

**Figure 7:**
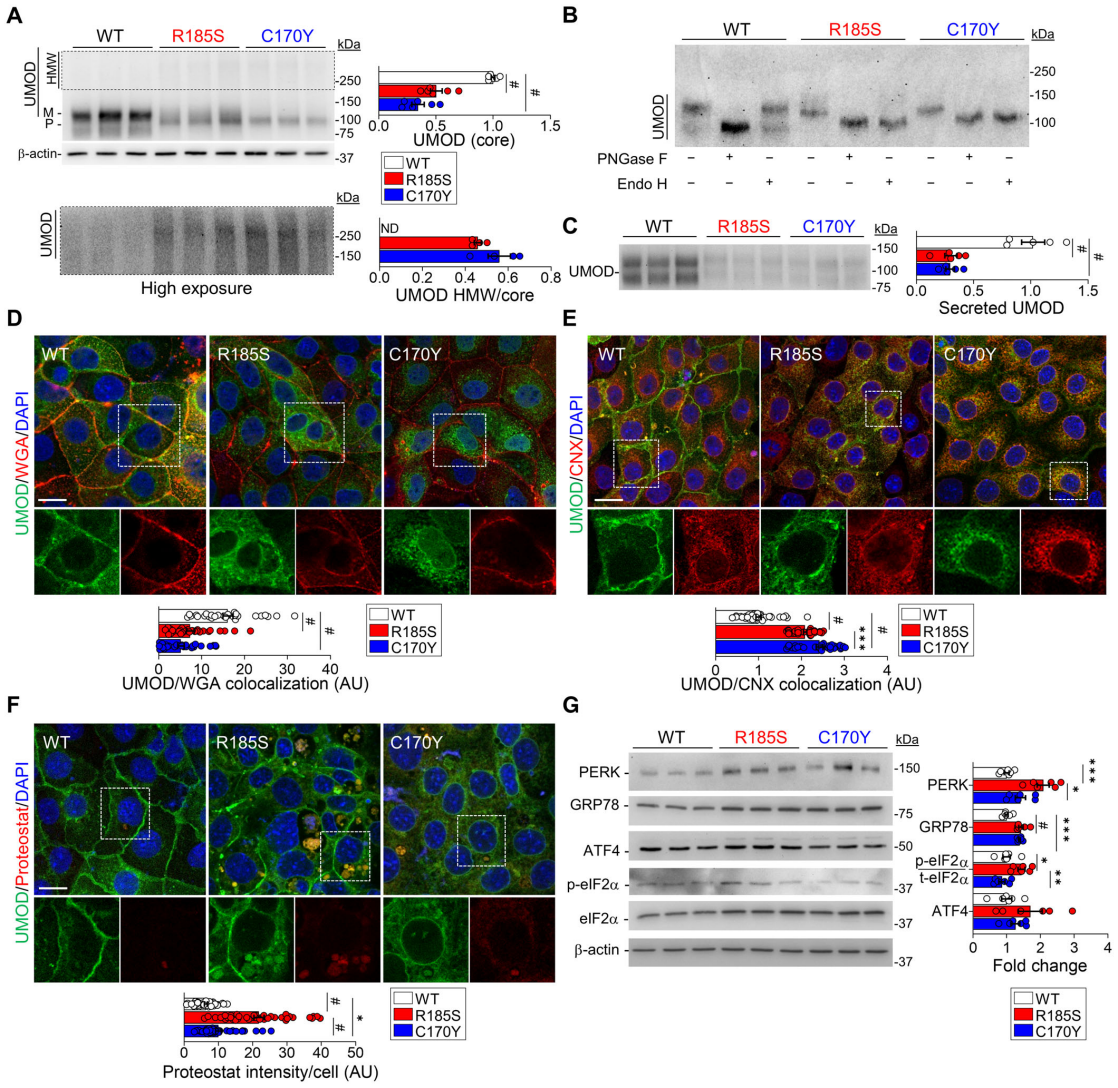
*In vitro* analysis of mutant uromodulin trafficking in mIMCD cells AImmunoblot analysis of uromodulin (UMOD) in *UMOD*‐*GFP* cell lysates. β‐actin used as a loading control (*n* = 6 biological replicates). M: mature, P: precursor, HMW: high molecular weight. Bottom panel: longer exposure of HMW UMOD aggregates, ^#^ *P* < 0.0001.BImmunoblot of UMOD from *UMOD*‐*GFP* cell lysates following PNGaseF or EndoH treatment.CRepresentative immunoblot of UMOD in the apical medium of *UMOD*‐*GFP* cells (*n* = 5 biological replicates per group), ^#^ *P* < 0.0001.D–FRepresentative immunofluorescence analysis of UMOD (green) and WGA (D), calnexin (CNX) (E) or PROTEOSTAT® (F) (red) in *UMOD*‐GFP cells (*n* = 29–39 cells per genotype). White squares indicate images shown at higher magnification. Scale bar: 30 μm (D, E), 15 μm (F). (E): ***P = 0.0005; (F): *P = 0.0375; ^#^ *P* < 0.0001.GImmunoblot analysis of UPR markers in *UMOD*‐*GFP* cell lysates. β‐actin used as a loading control (*n* = 6–9 biological replicates per group). Quantification is relative to UMOD WT. PERK: ***P = 0.0002, *P = 0.0107; GRP78: ***P = 0.0001; p‐eIF2α/t‐eIF2α: *P = 0.02, **P = 0.0027; ^#^ *P* < 0.0001. Data information: Bars indicate the mean ± SEM. One‐way ANOVA with Tukey's *post hoc* test. Source data are available online for this figure.

To gain mechanistic insight into mutant uromodulin processing, we transduced immortalized mouse inner medullary collecting duct (mIMCD‐3) cells with GFP‐tagged human wild‐type (WT) or mutant (p.R185S, p.C170Y) *UMOD*. These cells (hereafter referred to as *UMOD*‐*GFP* cells) recapitulated the main biochemical features of ADTKD‐*UMOD*, including the formation of HMW (~above 250 kDa) aggregates (Fig 7A) that were resolved by treatment with DTT (Appendix Fig S12A), accumulation of EndoH‐sensitive premature uromodulin (Fig 7B) and reduced uromodulin secretion (Fig 7C). Wild‐type uromodulin is trafficked properly to the plasma membrane (Fig 7D), whereas both mutated isoforms show ER retention (Fig 7E).

The localization of intracellular aggregates was investigated using the PROTEOSTAT® dye, which labels aggresomes and related amyloid‐like aggregates. Compared to WT cells, both mutant *UMOD* cell lines showed an enrichment for the PROTEOSTAT dye signal, colocalizing with uromodulin at the perinuclear area; the signal was significantly higher in R185S cells (Fig 7F). The R185S PROTEOSTAT‐positive structures appeared to be surrounded by calnexin, suggesting that uromodulin aggregates are localized into ER inclusions (Appendix Fig S12B).

Analysis of the level of ER stress showed an increased signal for GRP78/BiP in both R185S and C170Y mutant cells, colocalizing with uromodulin (Appendix Fig S12C). Coimmunoprecipitation showed that both wild‐type and mutant uromodulin interacted with GRP78 (Appendix Fig S12D). However, higher expression of PERK, p‐eIF2α, ATF4 and CHOP at either the mRNA (Appendix Fig S12E) or protein (Fig 7G) levels was detected in R185S cells, whereas increased levels of the *Xbp1* transcript were observed in C170Y cells (Appendix Fig S12E).

When checking for quality control mechanisms, we detected higher levels of *Dnaja4* in C170Y cells, as in *Umod* ^C171Y/+^ kidneys, confirming the involvement of the protein folding machinery (Appendix Fig S12F). The C170Y cells also showed higher levels of the translocon component *Sec62* and a significant upregulation of ER‐phagy genes (*Rtn3*, *Retreg1*, *Sec62*), in contrast with the downregulation of *Ccpg1* in R185S cells (Appendix Fig S12F). These results suggest that the UPR is activated through the PERK and IRE1 branches in mutant *UMOD* cell lines, with specific quality control mechanisms activated in C170Y cells, supporting our *in vivo* findings. The differential expression of ER‐phagy transcripts observed in *Umod* KI kidneys was confirmed in *UMOD*‐GFP cells, suggesting potential involvement of ER‐phagy in mutant uromodulin clearance.

### Clearance of mutant uromodulin relies on distinct degradation mechanisms

**Figure 8:**
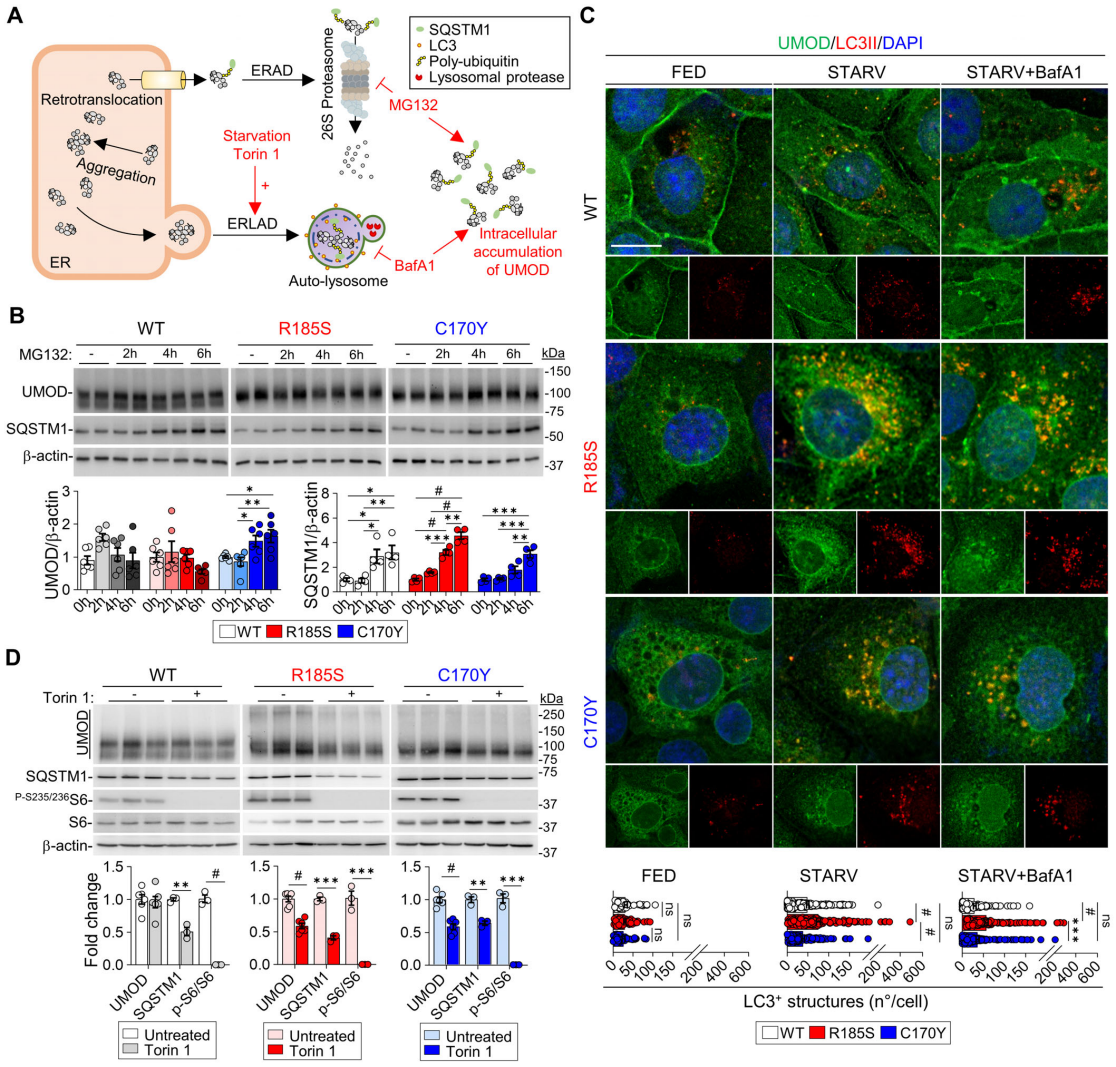
Degradative mechanisms involved in uromodulin clearance *in vitro* Diagram illustrating the degradation mechanisms of misfolded proteins and effect of different treatments. ERAD, ER‐associated degradation; ERLAD, ER‐to‐lysosome‐associated degradation.Immunoblot analysis of uromodulin (UMOD) and SQSTM1 in *UMOD*‐*GFP* cells following MG132 treatment (*n* = 4–6 biological replicates per condition). UMOD, C170Y: *P(0 h vs. 6 h) = 0.0286, *P(2 h vs. 4 h) = 0.0323, **P = 0.0065; SQSTM1, WT: *P(0 h vs. 4 h) = 0.0312, *P(0 h vs. 6 h) = 0.0121, *P(2 h vs. 4 h) = 0.0234, **P = 0.0091; R185S: ***P = 0.0002, **P = 0.0011; C170Y: ***P(0 h vs. 6 h) = 0.001, ***P(2 h vs. 6 h) = 0.0002, **P(4 h vs. 6 h) = 0.0066; ^#^ *P* < 0.0001.Immunofluorescence analysis of UMOD (green) and LC3 (red) in *UMOD*‐*GFP* cells following starvation and bafilomycin A1 treatment. Nuclei are counterstained with DAPI (blue). Scale bar: 10 μm (*n* = 41–59 cells per condition). Separate channels are shown below each panel. FED: ^*ns*^ *P*(WT vs. R185S) = 0.8672, ^*ns*^ *P*(WT vs. C170Y) = 0.4889, ^*ns*^(R185S vs. C170Y)*P* = 0.7483; STARV: ^*ns*^ *P* = 0.1153; STARV+BafA1: ^*ns*^ *P* = 0.4738, ***P = 0.0002; ^#^ *P* < 0.0001.Immunoblot analysis of UMOD, SQSTM1 and mTOR effectors following treatment with Torin 1 in *UMOD*‐*GFP* cells. β‐actin was used as a loading control. Densitometry analysis relative to untreated samples. WT, SQSTM1: **P = 0.0018; R185S, SQSTM1: ***P = 0.0001; p‐S6/S6: ***P = 0.0007; C170Y, SQSTM1: **P = 0.0027; p‐S6/S6: ***P = 0.0001; ^#^ *P* < 0.0001. Data information: Bars indicate the mean ± SEM. One‐way ANOVA with Tukey's *post hoc* test. Source data are available online for this figure.

To test whether the observed differences in trafficking, aggregates and UPR induction reflect distinct clearance systems of mutant uromodulin, we investigated the ER‐associated degradation (ERAD) and ER‐to lysosome‐associated degradation (ERLAD). To this end, we focus on the terminal stations of both pathways exposing the cells to either proteasome (MG132) or autophagy (bafilomycin A1) inhibitors respectively (Fig 8A). Indeed, higher baseline levels of the ubiquitin‐binding protein SQSTM1 were observed in *UMOD* C170Y cells, hinting at potential mechanistic differences underlying degradation of mutant uromodulin (Appendix Fig S13A and B). Following MG132 treatment, which increased ubiquitin and SQSTM1 in all cells, uromodulin levels strongly increased in C170Y‐expressing cells, whereas no significant change was detected in *UMOD* WT and R185S cells (Fig 8B; Appendix Fig S13C). Of note, MG132 treatment did not affect the *UMOD* mRNA expression in *UMOD*‐GFP cells, whereas *Sqstm1* mRNA expression had a similar time‐dependent increase than that observed by immunoblot, confirming that the observed effect is exclusively caused by proteasomal inhibition (Appendix Fig S13D). Immunofluorescence analysis confirmed the higher SQSTM1 signal intensity in C170Y mutant cells, further enhanced by MG132‐treatment (Appendix Fig S13E). The specificity of proteasomal blockage effect on uromodulin accumulation was confirmed by using the more selective proteasome inhibitor Bortezomib (Appendix Fig S13F). These data suggest that mutant cells respond differently to proteasomal inhibition, with C170Y mutant uromodulin being mainly targeted to ERAD.

Since R185S uromodulin did not increase following MG132 treatment, we investigated the potential involvement of the autophagy‐lysosomal degradation machinery (Fig 8A). Starvation induced a significant decrease in uromodulin levels in R185S and C170Y cells, while levels in WT cells were unaffected (Appendix Fig S14A). Treatment with BafA1 caused accumulation of uromodulin in R185S cells and C170Y cells, in contrast with no significant changes in WT cells (Appendix Fig S14A). The increase in LC3II and SQSTM1 levels suggested no impairment of autophagic flux (Appendix Fig S14A). After starvation, we detected a higher number of punctated LC3‐positive structures colocalizing with uromodulin in R185S cells than in C170Y and WT cells, an effect that was further enhanced by BafA1 treatment (Fig 8C). Treatment with BafA1 led to a significant shift of uromodulin signal to LAMP1^+^ structures in both mutant R185S (Mean delta +37%, *P* < 0.0001) and C170Y cells (Mean delta +69%, *P* < 0.0001), compared to untreated cells respectively. Of note, BafA1 treatment had a significantly higher impact on *UMOD* C170Y cells, suggesting a higher propensity of the C170Y mutant for degradation (Appendix Fig S14B). The importance of ERLAD in the clearance of mutant uromodulin was further confirmed by using the VPS34 inhibitor SAR405, which had a similar effect on mutant uromodulin than BafA1 (Appendix Fig S14C).

These data suggest that the ERAD and ERLAD pathways may preferentially clear distinct uromodulin mutants. They also indicate the potential therapeutic value of inducing the autophagy‐lysosomal pathway in ADTKD‐*UMOD*.

### Stimulating autophagy improves the clearance of mutant uromodulin

Considering the potential role of ERLAD in the clearance of mutant uromodulin, we targeted this pathway by exposing the cells to the canonical mTOR complex 1 (mTORC1) inhibitor Torin 1. The efficient inhibition of mTORC1 signaling, as indicated by lower phosphorylation levels of mTORC1 target protein S6, was reflected by a significant decrease in both core and HMW uromodulin aggregates in the R185S and C170Y mutant cells (Fig 8D). Treatment with bafilomycin A1 (4 h following Torin 1) confirmed that the uromodulin reduction following Torin 1 treatment was due to autophagy induction. Bafilomycin A1 treatment inhibited lysosomal degradation and autophagy, as indicated by increased LC3B‐II and SQSTM1 levels and significantly increased uromodulin levels in mutant cells compared to treatment with Torin 1 alone (Appendix Fig S14D). These data confirm the role of ERLAD in the clearance of mutant uromodulin.

## Discussion

ADTKD‐*UMOD* is one of the most common monogenic kidney diseases, invariably causing kidney failure. A pressing need for ADTKD, as for other dominantly inherited diseases, is to bridge the gap linking postulated gain‐of‐function mutations with clinically relevant end points. By combining human, mouse, and cellular studies, we show how representative missense mutations of uromodulin, associated with divergent disease severity, lead to distinct trafficking defects and propensity to form intracellular aggregates, with differential activation of ER stress and UPR, reflected by variable end‐organ damage. The *Umod* ^C171Y/+^ mice showed a distinct protein folding chaperone response, preventing accumulation of premature uromodulin in the kidneys. Conversely, the *Umod* ^R186S/+^ mice presented a massive accumulation of premature uromodulin in the kidney, triggering the formation of intracellular aggregates and a pathogenic cascade. Studies in kidney cells confirmed differences in the formation of aggregates, with activation of distinct clearance mechanisms. Enhancement of autophagy by starvation and mTORC1 inhibition decreased uromodulin aggregates, suggesting a potential therapeutic strategy. These observations substantiate a model for allelic effects and the role of toxic aggregates in the progression of ADTKD‐*UMOD*, with relevance for autosomal dominant disorders with toxic gain‐of‐function mechanisms.

More than 95% of the *UMOD* mutations associated with ADTKD are missense, clustering in exon 3, and, in more than 50% of the cases, involving cysteine links (Olinger *et al*, 2020). The novel *Umod* mice were aimed at investigating the impact of two mutations of *UMOD* standing for divergent clinical progression. Intuitively, one could assume that substitution of a conserved cysteine would affect uromodulin stability through disruption of disulfide bridges, potentially triggering the formation of aggregates and disease progression (Dahan *et al*, 2003; Devuyst *et al*, 2019; Olinger *et al*, 2020). A previous study comparing the severity of two *Umod* mutant mice (p.Cys93Phe and p.Ala227Thr) obtained by ENU random mutagenesis evidenced differences in uromodulin maturation, although corresponding mutations are not reported in human ADTKD‐*UMOD* (Kemter *et al*, 2013). Yet, it was shown recently that among patients with ADTKD‐*UMOD*, carriers of missense mutations involving a cysteine did not experience worse disease progression than those involving another residue (Olinger *et al*, 2020). Analysis of clinical endpoints revealed that patients harboring the *UMOD* p.Arg185Ser mutation progressed to kidney failure much faster, by more than 25 years, than those with the p.Cys170Tyr mutation. This difference was reflected by findings on human kidney biopsies, where the p.Arg185Ser mutation was associated with stronger intracellular accumulation, ER stress, inflammation, and fibrosis, whereas the p.Cys170Tyr mutant showed an overall milder damage. Remarkably, these distinct disease manifestations were substantiated in the *Umod* KI lines, with strong allelic and gene‐dosage effects on uromodulin processing and kidney damage and fibrosis.

While trafficking defects and accumulation of misfolded uromodulin in TAL cells have been reported in human kidney biopsies (Dahan *et al*, 2003; Rampoldi *et al*, 2003; Vylet'al *et al*, 2006) and previous mutant *Umod* mouse models (Kemter *et al*, 2009, 2013; Bernascone *et al*, 2010; Johnson *et al*, 2017; Piret *et al*, 2017), the role and fate of uromodulin aggregates remain essentially unknown. The availability of the new KI lines, matched for age and sex, allowed to decipher the link between trafficking, maturation defects, and formation of uromodulin aggregates in the kidney. These aggregates, associated with the disappearance of mature uromodulin, were consistently detected in *Umod* ^R186S/+^ kidneys, even at 2 weeks of age. Conversely, uromodulin aggregates were only detected lately, and in homozygous *Umod* ^C171Y/C171Y^ kidneys, together with the mature form and ER‐glycosylated uromodulin. The distinct mutations influenced the biochemical properties of the uromodulin aggregates, reflected in the differential induction of ER stress, as evidenced by the signal for GRP78/BiP and the PERK and IRE1α branches of the UPR in *Umod* ^R186S^ but not in *Umod* ^C171Y^ kidneys. Together, these analyses indicate the crucial role of uromodulin aggregates in driving the induction of ER stress and UPR, and the extent of kidney damage.

Importantly, deletion of the wild‐type *Umod* allele in heterozygous *Umod* ^R186S^ mice increased the amount of uromodulin aggregates and worsened inflammation, tubular damage markers and fibrosis. The fact that these effects are not reflected by changes in clinical parameters may be due to the very rapid disease progression in *Umod* ^R186S^ mice, overcoming the subcellular modifications. The fact that, despite the global reduction in uromodulin expression, there is increased aggregation, ER stress, and inflammatory parameters compared to the *Umod* ^R186S/+^ kidneys strongly supports the pathogenic role of aggregates. Since *Umod* KO mice do not show any phenotype related to ADTKD‐*UMOD* (Raffi *et al*, 2006), these results suggest that wild‐type uromodulin may protect against the formation of uromodulin aggregates. Studies on Huntington disease (HD), also characterized by a toxic gain of function, reported a similar protective role of wild‐type huntingtin (Htt) protein in the disease: the absence of wild‐type protein in HD mutant mouse and Drosophila models of Htt toxicity leads to a more severe phenotype (Van Raamsdonk *et al*, 2005; Zhang *et al*, 2009). The protective role of the wild‐type allele may be to facilitate the trafficking of mutant protein to the plasma membrane or, alternatively, to balance the propensity of mutant uromodulin to form aggregates.

Our results suggest potential mechanisms accounting for the strong allelic and gene dosage effects on the formation of uromodulin aggregates and their toxicity to tubular cells. *First*, the transcriptional profiles revealed a canonical signature of ADTKD‐*UMOD* progression, i.e., inflammation, fibrosis, and kidney failure in the *Umod* ^R186S^ kidneys, contrasting with an increased expression of genes involved in protein folding in the *Umod* ^C171Y^ kidneys. The changes driven by the R186S mutation are in line with the emerging role of ER proteostasis in kidney disease, as evidenced for intermediate‐effect size variants in *UMOD* (Olinger *et al*, 2022) and for mutations in *MUC1* also associated with ADTKD (Dvela‐Levitt *et al*, 2019). Notably, *Umod* ^R186S^ kidneys show increased expression of the key autophagy markers SQSTM1 and ATG5. These baseline levels are not sufficient to counteract the mutant uromodulin accumulation, supporting the potential value of enhancing autophagy as a therapeutic strategy. Furthermore, the strong induction of lipocalin‐2 (LCN2) in the R186S kidneys may provide a link between ER stress, inflammation, and fibrosis, as LCN2 is a known homing factor for inflammatory cells and an active player in kidney disease progression (Viau *et al*, 2010; El Karoui *et al*, 2016).

Recent structural data for human uromodulin allowed us to generate *in silico* model predictions for the two mutations (Appendix Fig S15). The p.Cys170Tyr substitution results in the loss of a disulphide bridge with Cys155. Furthermore, due to the higher bulkiness of tyrosine compared to cysteine, several clashes with the surrounding residues are predicted by our model (Appendix Fig S15A). On the other hand, the p.Arg185Ser mutation causes the loss of a salt bridge with Asp196 (Appendix Fig S15B). The Arg185 residue falls into a very conserved region in the D10C domain, required for the proper orientation of two critical N‐glycans targeted by several mutations associated with ADTKD (Stsiapanava *et al*, 2022). In particular, the loss of either Arg185 or Asp196 is predicted to disrupt the interaction between the helix 3_10_B and loop 3_10_B‐βB (Stsiapanava *et al*, 2022). Conversely, the increased folding capacity detected in the C171Y kidneys could explain why most of the uromodulin in these kidneys is terminally glycosylated and localized at the apical plasma membrane, with no decrease in its urinary excretion. Over time, these folding mechanisms may become metabolically challenging and/or insufficient to balance the sustained production of mutant uromodulin (Di Domenico & Lanzillotta, 2022).

*Second*, studies in R185S and C170Y cells demonstrated a different retention of mutant uromodulin altering ER homeostasis, reflected by differences in the formation of uromodulin aggregates – consistently higher in cells expressing R185S compared to the C170Y mutant. The upregulation of specific translocon components and ER‐phagy genes suggests that specific quality control mechanisms are activated in C170Y cells, modulating the formation of toxic aggregates.

*Third*, investigations of cell degradative mechanisms suggest that uromodulin mutants emerge as emblematic clients of ERAD and ERLAD, with C170Y uromodulin primarily degraded through the former pathway, while both mutants are substrates of the latter. These results are in line with the fact that if chaperone‐mediated folding cannot rescue misfolding, proteasomal degradation occurs since UPS is the main ER quality control mechanism (Lim & Yue, 2015). However, UPS can degrade misfolded proteins generated in the ER via ERAD, whereas ER portions containing ERAD‐resistant misfolded proteins (e.g., aggregates) are mainly cleared by ERLAD (Rudinskiy & Molinari, 2023).

The central role of ER‐phagy in clearing uromodulin aggregates offers perspectives for a therapeutic strategy. Previous studies suggested that autophagy could be impaired in ADTKD‐*UMOD* and rescued by mTOR inhibition (Johnson *et al*, 2017). Here, induction of autophagy by either starvation or mTORC1 inhibition led to a decrease in mutant uromodulin and HMW aggregates in both mutant cell lines, suggesting the potential value of activators of autophagy to clear mutant uromodulin. Further studies will be needed to validate this attractive possibility, taking advantage of experience in neurogenerative disorders and recently developed systems of targeted degradation of aggregation‐prone proteins (Cui *et al*, 2022; Ji *et al*, 2022).

In conclusion, we demonstrated that the differential effect of specific missense *UMOD* mutations on kidney disease progression is due to their propensity to misfold and aggregate, overcoming the capacity to activate chaperone and folding systems. The fact that toxic uromodulin aggregates drive end‐organ damage implies that treatment strategies should aim to decrease uromodulin aggregation and/or to promote folding capacity. As in other dominant, toxic gain‐of‐function disorders, the protective role of wild‐type uromodulin suggests the importance of allele‐specific targeting to decrease the levels of uromodulin. In addition to activators of autophagy, activation of specific degradative pathways may be required to promote the clearance of uromodulin mutants.

## Materials and Methods

### Human studies

For the assessment of kidney phenotypes according to *UMOD* mutations (age at onset of kidney failure and Kaplan–Meier analysis), only physician‐verified data from ADTKD‐*UMOD* patients/families recruited in Belgium or Switzerland were entered (in total, 78 patients from 30 families with 24 different *UMOD* mutations and with verified clinical data were analyzed). Kidney failure was defined as eGFR<15 ml/min/1.73 m^2^ or initiation of renal replacement therapy (dialysis or kidney transplantation). Patients harboring the p.Cys170Tyr and p.Arg185Ser mutations are the most prevalent among the slow‐ and fast‐progressing disease subgroups, respectively. The individuals are part of a previously described ADTKD‐*UMOD* cohort (Olinger *et al*, 2020).

### Animal experiments

All experiments were performed on age‐ and sex‐matched littermates on the C57BL/6 background. *Umod* ^C171Y^ and *Umod* ^R186S^ mice were generated by PolyGene Transgenetics (Rümlang, Switzerland). *Umod* ^−/−^ mice (Mo *et al*, 2004b) were kindly provided by Prof. Xue‐Ru Wu (New York University) and used to generate *Umod* ^R186S/−^ mice. All animals were housed at the laboratory animal service center of the University of Zurich under specific pathogen‐free conditions and maintained under controlled temperature and humidity, with 12‐h dark/light cycle and *ad libitum* access to tap water and standard chow (Diet AO3, SAFE). The number of replicates for each experiment is indicated in the figure legends. Samples from the same experiment were processed simultaneously.

### Cell models

mIMCD‐3 (ATCC CRL‐2123) cells were transduced with lentiviral particles for the expression of the indicated GFP‐tagged human uromodulin isoforms (WT, C170Y, and R185S). Lentiviral constructs were generated as described in Schaeffer *et al* (2017). The primers used for mutagenesis using the Quickchange Lightning mutagenesis kit (Stratagene, La Jolla, CA) are as follows:

hUmod_R185S_F 5′‐GTACTCGGTGCTGCTCCAGTACTCGTCCA‐3′;
hUmod_R185S_R 5′‐TGGACGAGTACTGGAGCAGCACCGAGTAC‐3′;
hUmod_C170Y_F 5′‐GGATCCGCGTACACGAGCGCGTCGC‐3′;
hUmod_C170Y_R 5′‐GCGACGCGCTCGTGTACGCGGATCC‐3′.

Cells were cultured in Iscove's modified Dulbecco's medium (IMDM, Thermo Fisher Scientific, Waltham, MA, USA) supplemented with 10% fetal bovine serum (FBS, Thermo Fisher Scientific) and 1% Penicillin/Streptavidin (Thermo Fisher Scientific). The cells were authenticated and tested for mycoplasma contamination.

### Histological analysis and immunostaining

Immunodetection of uromodulin, GRP78 and CD3 was performed on 5 μm‐thick kidney sections obtained from nephrectomy samples of ADTKD‐*UMOD* patients with p.Arg185Ser mutation in *UMOD* (female, 41 years old, ESKD; nephrectomy during kidney transplantation) and p.Cys170Tyr in *UMOD* (male, 58 years old, ESKD; nephrectomy due to kidney neoplasia). After 1 h in blocking solution, the slides were incubated with sheep anti‐uromodulin primary antibody, followed by 1 h Alexa Fluor 488‐conjugated donkey anti‐sheep. The slides were then probed with either rabbit anti‐GRP78 or rabbit anti‐CD3 primary antibody, followed by 1 h incubation with AlexaFluor647‐conjugated donkey anti‐rabbit antibody. Coverslips were mounted with Prolong gold antifade reagent with 4′,6‐diamidino‐2‐phenylindole (DAPI, P36931, Thermo Fisher Scientific) and analyzed under a Leica STELLARIS 5 microscope (Leica Camera, Wetzlar, Germany) with a ×63/1.4 Plan‐ Apochromat oil‐immersion objective. Picrosirius red (ab150681, Abcam, Cambridge, UK) was performed according to the manufacturer's instructions. Briefly, samples were rehydrated and incubated for 1 h in picrosirius red solution, rinsed briefly in 0.5% acetic acid, dehydrated in absolute ethanol, cleared and mounted. Periodic acid‐Schiff (PAS) staining was performed according to standard protocols. Both Picrosirius red and PAS were evaluated by use of a Zeiss AxioScanner.Z1 (Carl Zeiss, Oberkochen, Germany).

For mouse tissues, kidneys were harvested and processed as previously described (Olinger *et al*, 2019; Schiano *et al*, 2019). Five‐micrometer‐thick sections were incubated with the primary antibody for 2 h at RT or overnight at 4°C. The sections were then incubated with the appropriate AlexaFluor‐conjugated secondary antibody (1:300, Life Technologies, Carlsbad, CA) for 1 h at RT. The sections were mounted using Prolong Gold antifade reagent with DAPI (P36931, Thermo Fisher Scientific) and viewed under a confocal microscope (Leica Microsystems GmbH, Wetzlar, Germany) using a ×63 1.4 NA oil immersion objective.

### Generation of the *Umod* targeting vectors

Both targeting vectors contained 8.8 kb of the *Umod* gene with the respective mutations, a FRT‐flanked neomycin resistance cassette and a SalI restriction site that was used for linearization of the vectors prior to electroporation of C57BL/6N‐derived embryonic stem (ES) cells. A total of 300 p.R186S and 400 p.C171Y clones were resistant to selection with Geneticin, with 23 p.R186S and 24 p.C171Y clones that had undergone homologous recombination and were subsequently expanded. Presence of the mutations was established by restriction/PCR combined analysis and by Southern blot (Unpublished observation). Selected positive clones were injected in blastocysts from gray C57BL/6 mice (R186S: 4 clones in 60 blastocysts; C171Y: 2 clones in 36 blastocysts) and the surviving blastocysts were transferred in CD‐1 foster mice. The resulting highly chimeric mice (80–100%) were mated to gray C57BL/6N Flp‐deleter mice to obtain offspring in which the neomycin resistance cassette was excised.

### Biochemical analysis and tissue collection

Urine samples were collected using individual metabolic cages (UNO Roestvastaal BV, Zevenaar, Netherlands) after appropriate training. Blood was collected by venous puncture or cardiac puncture at the time of sacrifice and centrifuged at 2,000 *g* for 15 min at 4°C in heparin‐coated tubes (Sarstedt AG, Nürnbrecht, Germany) to separate plasma from cells. Creatinine, electrolytes, uric acid, and urea were measured on a UniCel® DxC 800 Synchron® Clinical System or AU480 Clinical Chemistry System (Beckman Coulter, Brea, CA, USA) according to the manufacturer's instructions. Urine and plasma osmolality were measured on a multisample osmometer (Advanced Instruments Model 2020, Norwood, MA, USA). Mice were anesthetized by intraperitoneal injection with a combination of ketamine (100 mg/ml) and xylazine (20 mg/ml) (Streuli Pharma AG, Uznach, Switzerland) in 0.9% NaCl for the collection of the kidneys. One kidney was flash‐frozen for RNA extraction, while the other was further processed for protein extraction and histological analyses. For medulla‐enriched kidney fractions, kidneys were dissected in PBS under a Stereo microscope (VWR, SB350H VisiScope). Medulla‐ and cortex‐enriched fractions were collected for protein extraction and validation of enrichment by Western blotting. For EM sample preparation, mice were anesthetized and perfused with a fixative solution containing 0.1 M sodium cacodylate (820670, Merck), 0.1 M sucrose (107687, Merck), 2% formaldehyde (#15710, Electron Microscopy Sciences, Hatfield, PA, USA) and 0.1% glutaraldehyde (#16220, Electron Microscopy Sciences). The kidneys were harvested, cut into ~1 mm‐thick slices and stored at 4°C in fixative solution. Further processing was performed at the Center of Microscopy and Image Analysis of the University of Zurich.

### Protein sample preparation and immunoblotting

Kidneys and cells were lysed in radioimmunoprecipitation assay (RIPA) buffer (Sigma‐Aldrich, St. Louis, MO, USA) containing protease and phosphatase inhibitors (Sigma‐Aldrich) using an IKA T10 basic tissue homogenizer (IKA, Staufen, Germany) or cell scraper (99002, TPP Techno Plastic Products, Trasadingen, Switzerland). The lysates were briefly sonicated and centrifuged for 15 min at 1,000 *g* and 4°C to remove debris. Protein concentrations were determined using the bicinchoninic acid (BCA) protein assay kit (Thermo Fisher Scientific), while urine was loaded according to creatinine concentration. For native PAGE, kidney lysates were diluted in sample buffer containing 0.01% bromophenol blue (Sigma‐Aldrich), 25% glycerol, and 12.5% Tris–HCl buffer, pH 6.8 (Bio‐Rad, Hercules, CA, USA) and were run on 4–20% Mini‐PROTEAN TGX Gel (Bio‐Rad) in the absence of SDS. For deglycosylation analysis, samples were treated with PNGase F (P70404L, New England Biolabs, Ipswich, MA, USA) or Endo H (P0702, New England Biolabs) as previously described (Schiano *et al*, 2019), according to the manufacturer's instructions and analyzed by SDS–PAGE followed by immunoblotting. For semiquantitative immunoblot analysis, samples were reduced with DTT (except for uromodulin) and denatured by boiling. For partial uromodulin denaturation, samples were diluted in Laemmli sample buffer (Bio‐Rad) in absence of DTT, which was added for total uromodulin denaturation. These experiments were performed in technical triplicates, with three different mice per genotype. Samples were diluted in Laemmli sample buffer (Bio‐Rad), separated on a 7.5–12% SDS–PAGE gel and blotted onto methanol‐activated PVDF membranes. Membranes were blocked for 1 h in 5% w/v nonfat dry milk solution, followed by overnight incubation at 4°C with primary antibodies. Blots were subsequently incubated with peroxidase‐conjugated secondary antibodies and visualized by Western HRP Substrate (Millipore, Burlington, MA, USA). Immunoblots were quantified by densitometric analysis using ImageJ or Image Lab. Quantification of kidney samples loaded on the same membranes was normalized over β‐actin. According to the blots, the HMW uromodulin aggregates were expressed as ratio over total uromodulin or normalized over β‐actin. A list of primary antibodies used in this study is available in Appendix Table S13. Full length Western blot images can be found in Appendix Fig S16.

### Electron microscopy

For transmission electron microscopy (TEM), 1 mm thick kidney sections were immersed in 0.1 M cacodylate buffer containing 100 mM sucrose and 2.5% glutaraldehyde and embedded in epoxy resin. Correlative light‐electron microscopy (CLEM) was performed as previously published (Mateos *et al*, 2018). Briefly, sections were collected on wafers and washed with PBS (0.1 M, pH 7.4, 0°C), followed by incubation with 0.15% glycine in PBS (3 × 1 min) and washing with PBS (3 × 1 min). Blocking solution composed of 0.5% BSA (albumin fraction V, Applichem) and 0.2% gelatin (from bovine skin, Sigma) in PBS was applied for 5 min, and samples were incubated with anti‐uromodulin antibody (1:20) dissolved in blocking solution for 40 min at RT. After washing with blocking solution (6× 20 s), the wafers were incubated with anti‐sheep Alexa 647 secondary antibody (1:100, Life Technologies) in blocking solution (40 min at RT). After immunolabeling, sections were incubated with DAPI (4′,6‐diamidino‐2‐phenylindole dihydrochloride, Roche, 1:250 dilution) for 10 s and washed in PBS.

### Solubility assay

Solubility assay on kidneys from 4‐month‐old *Umod* KI mice was based on a differential centrifugation protocol already described to extract insoluble aggregates from brain tissue (Arseni *et al*, 2022). Briefly, 20 mg of frozen kidneys were extracted and homogenized in extraction buffer (10 mM Tris–HCL pH 7.5, 800 mM NaCl, 10% sucrose and 1 mM EGTA). The homogenates were adjusted to 2% Sarkosyl (L7414, Sigma Aldrich) and incubated for 30 min at 37°C. Following 10 min of centrifugation at 27,000 *g* at RT, the supernatants were spun at 166,000 *g* for 20 min at RT. Insoluble pellets were resuspended in 20 μl of 20 mM Tris–HCl pH 7.4, 150 mM NaCl. To preserve the HMW aggregates, all these steps were performed in non‐reducing conditions. Following a last centrifugation step at 166,000 *g* for 20 min, the sarkosyl‐insoluble pellets were resuspended in 15 μl of 20 mM Tris–HCl pH 7.4, 150 mM NaCl, followed by 30 s sonication. Protein concentrations of soluble fractions were determined using the BCA protein assay kit. Finally, 10 μg of soluble material and the total volume of insoluble material supplemented with Laemmli were boiled for 5 min and analyzed by Western blotting.

### RNA isolation, reverse transcription, and quantitative PCR

Total RNA was extracted from kidney and cells using Aurum TM Total RNA Fatty and Fibrous Tissue Kit (Bio‐Rad) or RNAqueous™ Micro Total RNA Isolation Kit (Thermo Fisher Scientific) following the manufacturer's protocol. Analysis of gene expression levels in mouse kidneys and cells was performed based on the MIQE guidelines. Reverse transcriptase reaction with iScript TM cDNA Synthesis Kit (Bio‐Rad) was executed with up to 1 μg of RNA. When needed, PCR products were size fractionated on a 1.5% agarose gel and stained with EZ‐VisionR One (AMRESCO, Solon, OH). The variations in mRNA levels of the target genes were established by relative RT–qPCR with a CFX96TM Real‐Time PCR Detection System and the iQ™ SYBR Green Supermix (Bio‐Rad) for the detection of single PCR product accumulation. Primers specific to targets were designed with Beacon Design 2.0 (Premier Biosoft International, Palo Alto, CA). The PCR conditions were 95°C for 3 min, followed by 40 cycles of 15 s at 95°C and 30 s at 60°C. The efficiency of each set of primers was determined by dilution curves (Appendix Table S14). GeNorm version 3.4 was used to characterize the expression stability of the candidate reference genes in the kidney. The normalization factor was determined using 6 housekeeping genes (*18S*, *36B4*, *Actb*, *Gapdh*, *Hprt1*, and *Ppia*) for tissue samples. The relative changes were determined by the formula: 2^−ΔΔct^ and expressed as a percentage relative to *Umod* ^+/+^ mice or *UMOD* WT cells.

### Isolation of TAL segments from mouse kidneys

The TAL segments were isolated from mice as previously described (Glaudemans *et al*, 2014). Briefly, kidneys were harvested, the outer medulla was cut into 1‐mm‐square pieces, digested for 30 min with 245 units/ml type‐2 collagenase (Worthington Biochemical Corp, Lakewood, USA) and 96 μg/ml soybean trypsin inhibitor (Sigma), and segments were sieved through a 250‐μm filter (BVBA Prosep, Zaventem, Belgium) and collected on an 80‐μm filter (BVBA). TALs were selected under a light microscope based on morphology (Leica DMIL, Bensheim, Germany). Pooled TAL segments (∼100) were resuspended in 50 μl of Laemmli sample buffer (Bio‐Rad) and analyzed by Western blot.

### Semiquantitative analysis of fibrosis

Quantification of interstitial fibrosis was performed as previously described (Courtoy *et al*, 2020). Briefly, full section‐scans of 4 month‐old *Umod* ^+/+^, *Umod* ^R186S^ and *Umod* ^C171Y^ kidneys were acquired at a resolution of 200× by a Zeiss Axio Scan.z1 slide scanner (Carl Zeiss, Oberkochen, Germany), equipped with a Hitachi HV‐F202FCL digital video camera (Hitachi, Tokyo, Japan). Images were acquired using ZENblue software (Carl Zeiss SpA) and analyzed using ImageJ. Large blood vessels and inner medulla were manually excluded from quantification. Images were deconvoluted to obtain separate collagen signal (red) and cytoplasmic signal (yellow). Fibrotic area was calculated as percentage of the collagen area over the whole tissue section.

### RNA‐sequencing and bioinformatic analysis

RNA sequencing of whole kidney lysates from age matched male *Umod* KI mice was performed at Eurofins Genomics (Ebersberg, Germany) using an Illumina HiSeq 2500 sequencer (Illumina, San Diego, CA, USA). Information on RNA quality and yield can be found in the Appendix (Appendix Table S15). Network Analyst 3.0 (Zhou *et al*, 2019) was used for processing and analysis of RNA‐Seq data, principal component analysis (PCA), generation of heat maps and overrepresentation analysis (ORA). Data were log_2_‐normalized and analyzed using the DESeq2 algorithm (Love *et al*, 2014). Volcano plots and ORA scatterplots were generated with GraphPad Prism.

### Cell culture and treatments

For UPS treatment, *UMOD*‐*GFP* cells were treated for 6 h with 2.5 μM proteasome inhibitor MG132 (M8699, Sigma‐Aldrich). Protein samples were collected at 2, 4, and 6 h post treatment for Western blot analysis. For the autophagy experiment, cells were incubated for 6 h with serum‐free medium (12440‐061, IMDM Gibco) and for 4 h with 250 nM bafilomycin A1 (b1793, Sigma‐Aldrich). For mTOR treatment, cells were incubated for 16 h with 250 nM mTOR inhibitor Torin 1 (4247, TOCRIS Bioscience, Bristol, UK) and 4 h with 200 nM bafilomycin A1. Samples were analyzed by both Western blot and immunofluorescence analyses.

### Cells immunofluorescence and image analysis

*UMOD*‐*GFP* cells were fixed in 4% PFA, quenched with 50 mM NH_4_Cl, and permeabilized in blocking buffer solution containing 0.5% saponin and 0.5% BSA dissolved in PBS. Subsequently, cells were incubated overnight at 4 °C with the appropriate primary antibody or stained for 30 min at RT° with PROTEOSTAT® dye (ENZ‐51035‐K100) as previously described (Shen *et al*, 2011). For the PROTEOSTAT® Aggresome detection kit, all components were prepared according to the manufacturer's instructions. The samples were incubated for 45 min with suitable fluorophore‐conjugated Alexa secondary antibodies (1:400, Thermo Fisher Scientific), counterstained with DAPI (Invitrogen), mounted with Prolong Gold antifade reagent (P36931, Thermo Fisher Scientific) and analyzed by a Leica SP8 confocal laser scanning microscope (Leica Microsystems GmbH, Wetzlar, Germany). Quantitative image analysis was performed by randomly selecting five visual fields pooled from biological triplicates, with each field including at least 20–25 cells. The quantitative analyses were performed using open‐source cell image analysis software for cell phenotype (CellProfiler) and ImageJ.

### *In situ* cell death detection

For apoptosis labeling, Terminal transferase dUTP nick end labeling (TUNEL) assay was performed with In Situ Cell Death Detection Kit, Fluorescein (11 684 795 910, Roche, Basel, Switzerland), according to the manufacturer's instructions. The labeling procedure for difficult tissue was used. Briefly, slides were deparaffinized in xylene and rehydrated in a graded ethanol series. Antigen retrieval was carried out for 10 min with citrate buffer (pH 6.0) at 98°C, following by blocking for 30 min in Tris–HCl 0.1 M (pH 7.4) containing 3% BSA and 20% normal bovine serum. Slides were incubated with the TUNEL reaction mix for 1 h at 37°C. Slides were then probed with sheep anti‐uromodulin primary antibody (1/400; Meridien Life Science Inc. K90071C) for 1 h at room temperature, followed by AlexaFluor633‐conjugated donkey anti‐sheep (1/400; Invitrogen). Coverslips were mounted with Prolong gold antifade reagent with DAPI (P36931, Thermo Fisher Scientific) and analyzed under a Confocal microscope (Leica Microsystems GmbH) using a × 63 1.4 NA oil immersion objective.

### Co‐immunoprecipitation

*UMOD*‐*GFP* cells were grown to confluence in T75 flasks and lysed in 1 ml of RIPA buffer containing protease and phosphatase inhibitors, for 15 min at 4°C followed by 10 min centrifugation at 15,000 *g*. Cell lysates (1 mg) were incubated under rotation overnight at 4°C with 50 μl protein G‐Sepharose beads pre‐conjugated with 3 μl of rabbit anti GRP78 antibody (ab21685, Abcam) or with 6 μl of sheep anti‐uromodulin antibody (K90071C, Meridian Life Science Inc., Cincinnati, OH, USA). Beads were washed 3 times in PBS and the immunoprecipitated material was eluted with 20 μl of 50 mM glycine pH 2.8 and 1 M Tris–HCl pH 7.4 was used to neutralize the samples. Samples were boiled 5 min at 95°C for Western blot analysis.

### Proteasomal and autophagy inhibition

For proteasomal inhibition, *UMOD*‐*GFP* cells were treated with 1 μM Bortezomib (5043140001, Sigma‐Aldrich) from 2 to 6 h. Protein samples were collected at 2, 4, and 6 h post treatment for Western blot analysis. For autophagy inhibition, cells were incubated for 5 h with serum‐free medium (12440‐061, IMDM Gibco) supplemented with 5 μM PIK3C3/Vps34 inhibitor SAR‐405 (A8883, APExBIO). For baseline autophagy characterization, cells were incubated for 2 h with 200 nM bafilomycin A1, without prior incubation in serum‐free medium. Samples were analyzed by immunofluorescence analyses.

### *In silico* modeling of ADTKD‐*UMOD* mutations

Structural data for the full cryo‐EM structure of human uromodulin were downloaded from the RCSB protein data bank (https://www.rcsb.org/structure/7PFP), since the available experimental models for mouse uromodulin do not cover the region of the two mutations. Both mutations were modeled using the mutagenesis wizard of PyMol (Schrödinger LLC).

### Statistics

All values are expressed as the mean ± SEM. Prior to statistical analysis, sample populations were tested for normality (Shapiro–Wilk test) and equality of variances (*F*‐test). Two‐group comparisons were performed using either unpaired, two‐tailed Student's *t*‐test or the Mann–Whitney test in cases of nonnormal distribution of the samples. Comparisons between three or more groups were performed using one‐way ANOVA followed by Tukey–Kramer's *post hoc* analysis. *P* values < 0.05 were considered statistically significant. Sample size was estimated based on publicly available studies on ADTKD‐*UMOD* mouse models. As the characterization of the mouse models relies on knowledge on the genotype, blinding was not implemented. The robust regression and outlier removal (ROUT) method was used to determine samples to be excluded from further analysis.

### Study approval

Clinical and genetic information for ADTKD‐*UMOD* patients were retrieved from the Belgo‐Swiss ADTKD registry, comprising a subset of patients recruited in the International ADTKD Cohort (Olinger *et al*, 2020). Informed and written consent was obtained from all participants. All experiments conform to the principles set out in the WMA Declaration of Helsinki and the Department of Health and Human Services Belmont Report. The ADTKD registry was approved by the Institutional Review Board of the UCLouvain Medical School and Saint Luc University Hospital, Belgium (2011/04MAI/184) and the European Community's Seventh Framework Programme European Consortium for High‐Throughput Research in Rare Kidney Diseases (EURenOmics) Ethics Advisory Board and its participating centers. The use of human samples was approved by the UCLouvain Ethical Review Board. All animal experiments were performed in accordance with the ethical guidelines at University of Zurich (Zurich, Switzerland) and the legislation of animal care and experimentation of Canton Zurich, Switzerland. The experimental protocols were approved by the appropriate licensing committee (Kanton Zürich Gesundheitsdirektion Veterinäramt; l ZH049/17 and ZH195/20) at the University of Zurich. All animal handling, experimental procedures and data reports were conducted in accordance with the ARRIVE 2.0 guidelines (Percie du Sert *et al*, 2020).

## Author contributions

**Guglielmo Schiano:** Conceptualization; formal analysis; investigation; writing – original draft; writing – review and editing. **Jennifer Lake:** Conceptualization; formal analysis; investigation; writing – original draft; writing – review and editing. **Marta Mariniello:** Conceptualization; formal analysis; investigation; writing – original draft; writing – review and editing. **Céline Schaeffer:** Conceptualization; investigation; writing – original draft; resources. **Marianne Harvent:** Investigation. **Luca Rampoldi:** Conceptualization; investigation; writing – original draft; writing – review and editing; resources. **Eric Olinger:** Conceptualization; investigation; writing – original draft; writing – review and editing. **Oliver Devuyst:** Conceptualization; supervision; funding acquisition; writing – original draft; writing – review and editing.

## Disclosure and competing interest statement

The authors declare that they have no conflict of interest.

## For more information

OMIM – Uromodulin: https://www.omim.org/entry/191845.

OMIM – ADTKD‐*UMOD*: https://www.omim.org/entry/162000.

Orphanet: https://www.orpha.net/consor/cgi‐bin/OC_Exp.php?lng=EN&Expert=34149.

NORD: https://rarediseases.org/gard‐rare‐disease/autosomal‐dominant‐tubulointerstitial‐kidney‐disease/.

NIH‐GARD: https://rarediseases.info.nih.gov/diseases/10801/autosomal‐dominant‐tubulointerstitial‐kidney‐disease.

European Rare Kidney Disease Reference Network (ERKNET): https://www.erknet.org/.

Federation of European Patient Groups affected by Rare/Genetic Kidney Diseases (FEDERG): https://federg.org/.

## Supporting information



Appendix

Source Data for Figure 1

Source Data for Figure 2

Source Data for Figure 3

Source Data for Figure 4

Source Data for Figure 5

Source Data for Figure 6

Source Data for Figure 7

Source Data for Figure 8

## Data Availability

Primary RNA‐sequencing data have been deposited on Gene Expression Omnibus with the dataset identifier GSE214491 (https://www.ncbi.nlm.nih.gov/geo/query/acc.cgi?acc=GSE214491).
