# Supplementary material for: Allelic effects on uromodulin aggregates drive autosomal dominant tubulointerstitial kidney disease
Source: EMBO Mol Med. 2023 Oct 26;15(12):e18242. doi: 10.15252/emmm.202318242 (PMC10701617; doi:10.15252/emmm.202318242)
Supplement: Supplementary file 1 — Appendix [file EMMM-15-e18242-s008.pdf]

# Allelic Effects on Uromodulin Aggregates Drive Autosomal Dominant Tubulointerstitial Kidney Disease

## Appendix

## Table of contents

Appendix Figure S1: Uromodulin characteristics in the C171Y and R186S mouse models.................3
Appendix Figure S2: Early uromodulin processing defects in R186S/+ mice. .....................................4
Appendix Figure S3: Unfolded protein response in kidneys from *Umod* KI mice................................5
Appendix Figure S4: Lack of apoptosis or caspase activation in *Umod* KI kidneys.............................6
Appendix Figure S5: Mutant UMOD degradation relies on mutation-specific mechanisms. ................7
Appendix Figure S6: Markers of autophagy induction in *Umod* KI mouse kidneys.............................8
Appendix Figure S7: Macrophage infiltration in *Umod* KI mouse kidneys. .........................................9
Appendix Figure S8: Characterization of interstitial fibrosis in *Umod* KI mouse kidneys. .................10
Appendix Figure S9: RNA-seq analyses in 1-month-old R186S/+ kidneys. .......................................12
Appendix Figure S10: Differential expression and affected pathways in 4-month-old *Umod* KI kidneys. .....................................................................................................................13
Appendix Figure S11: Disease progression signature in *Umod* KI kidneys.......................................14
Appendix Figure S12: Distinct UMOD mutations trigger differential ER quality control responses. . 15
Appendix Figure S13: Proteasomal inhibition induces mutant UMOD accumulation.........................17
Appendix Figure S14: Autophagy modulation impacts on mutant UMOD levels. .............................19
Appendix Figure S15: Structural modelling of UMOD mutations.....................................................21
Appendix Figure S16: Uncropped Western blot membranes. ............................................................22
Appendix Table S1: Clinical characteristics of p. (Arg185Ser) ADTKD-UMOD family..................26
Appendix Table S2: Clinical characteristics of p. (Cys170Tyr) ADTKD-*UMOD* families...............27
Appendix Table S3: In silico analysis of selected *UMOD* missense variants. ...................................28
Appendix Table S4: Clinical and biochemical parameters of *Umod*$^{C171Y}$ mice.................................29
Appendix Table S5: Clinical and biochemical parameters of *Umod*$^{R186S}$ mice. ................................30
Appendix Table S6: Clinical and biochemical parameters of *Umod*$^{R186S/-}$ mice................................31
Appendix Table S7: Clinical and biological parameters of 4-month *Umod* KI mice per gender. ........32
Appendix Table S8: Top 50 DEGs in *Umod*$^{R186S/+}$ kidneys at 1 month.............................................33
Appendix Table S9: Top 50 DEGs in *Umod*$^{R186S/+}$ kidneys at 4 months. ..........................................34
Appendix Table S10: Top 50 DEGs in *Umod*$^{C171Y/+}$ kidneys at 4 months. .........................................35
Appendix Table S11: Top 50 DEGs in *Umod*$^{R186S/+}$ compared to *Umod*$^{C171Y/+}$ kidneys at 1 month.....36
Appendix Table S12: Top 50 DEGs in *Umod*$^{R186S/+}$ compared to *Umod*$^{C171Y/+}$ at 4 months................37
Appendix Table S13: List of primary antibodies................................................................................38
Appendix Table S14: Primers used for real-time RT-PCR analysis.....................................................39
Appendix Table S15: RNA-Seq quality and yield...............................................................................40
Appendix References ........................................................................................................................41

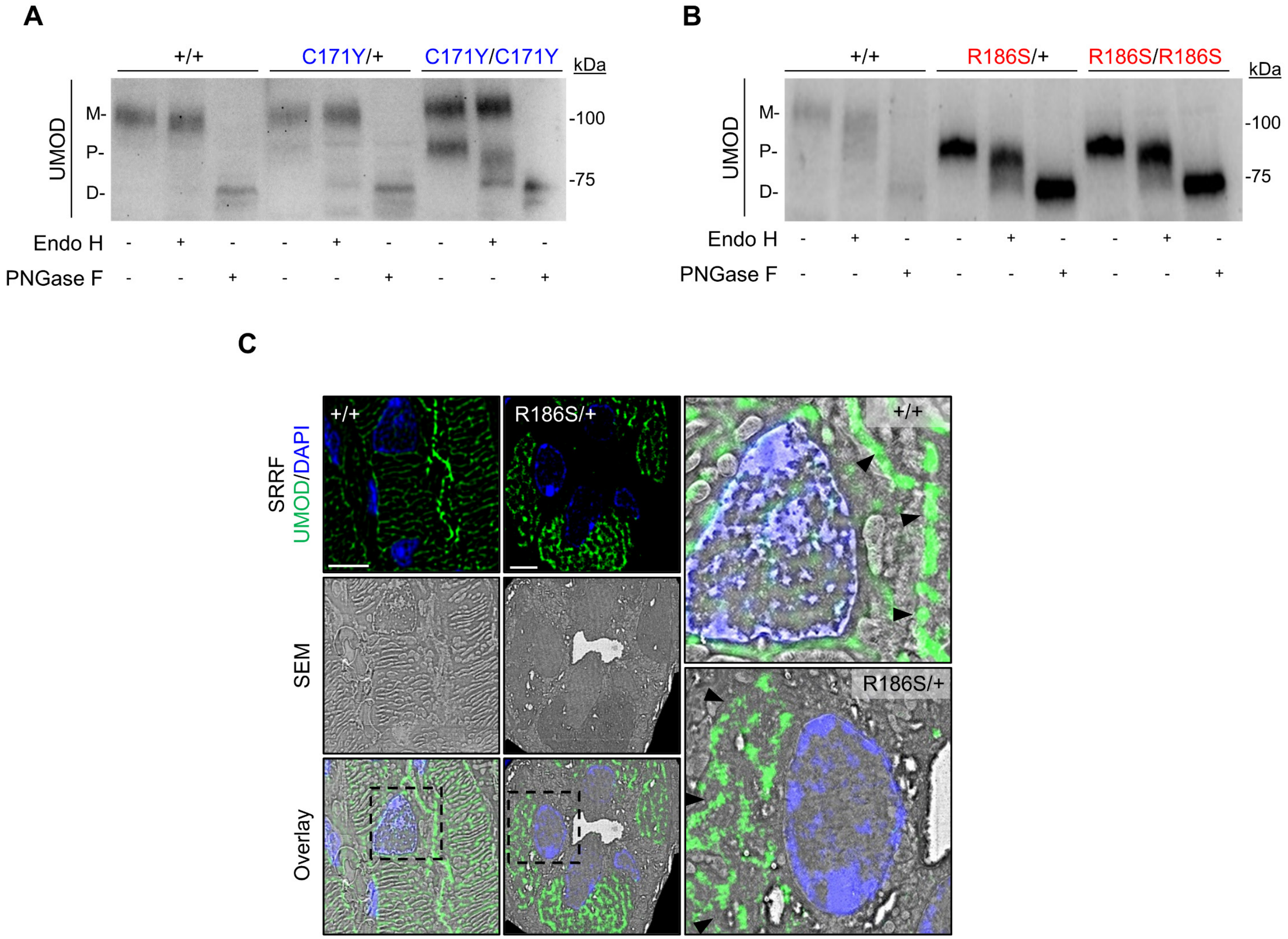


**Appendix Figure S1: Uromodulin characteristics in the C171Y and R186S mouse models.**

(**A-B**) Immunoblot analysis of UMOD following Endo H or PNGase F treatment in kidneys from 1-month-old C171Y (**A**) and R186S (**B**) mice. M: mature; P: precursor; D: deglycosylated. (**C**) Correlative Light Electron Microscopy (CLEM) of UMOD (green) on kidney sections from 3-month-old +/+ and R186S/+ mice. Nuclei are counterstained with DAPI (blue). Black arrowheads indicate UMOD localization (apical plasma membrane in +/+, endoplasmic reticulum in R186S/+). Scale bar: 5 µm. SRRF, super-resolution radial fluctuation; SEM, scanning electron microscopy.

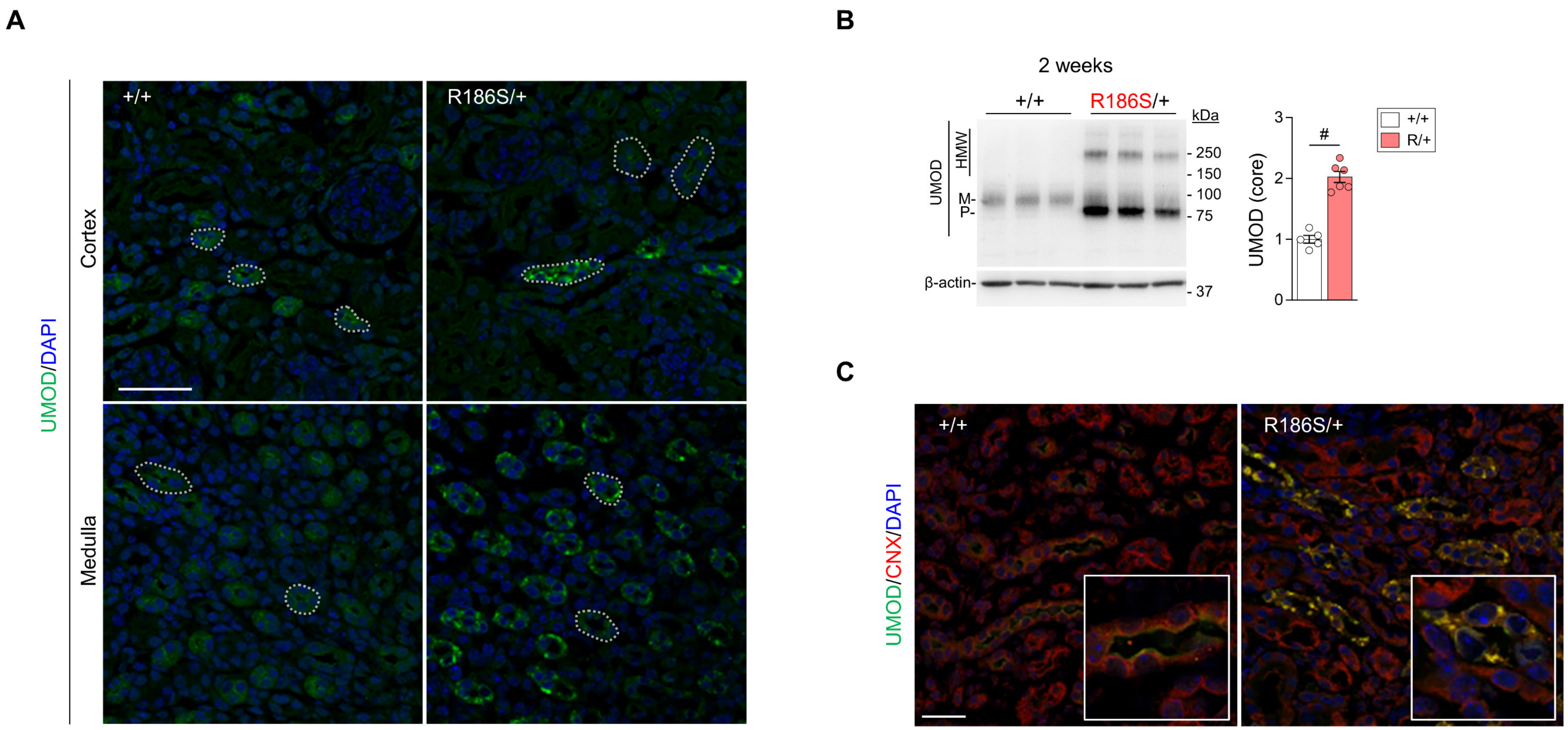


**Appendix Figure S2: Early uromodulin processing defects in R186S/+ mice.**

(**A**) Representative immunofluorescence analysis of UMOD (green) on kidney sections from 2-week-old +/+ and R186S/+ mice. Nuclei counterstained with DAPI (blue). Scale bar: 25 µm. Dotted lines identify different tubules with apical or intracellular UMOD accumulation.

(**B**) Representative immunoblot of UMOD in whole kidney samples from 2-week-old +/+ and R186S/+ mice (n=5 to 6 animals per group). β-actin used as loading control. M: mature UMOD; P: premature UMOD; HMW: high molecular weight. Densitometry analysis relative to +/+. Bars indicate mean ± SEM. Unpaired two-tailed t test, $^{\#}P<0.0001$.

(**C**) Representative immunofluorescence analysis of UMOD (green) and CNX (red) on kidney sections from 2-week-old mice. Nuclei are counterstained with DAPI (blue). Scale bar: 25 µm.

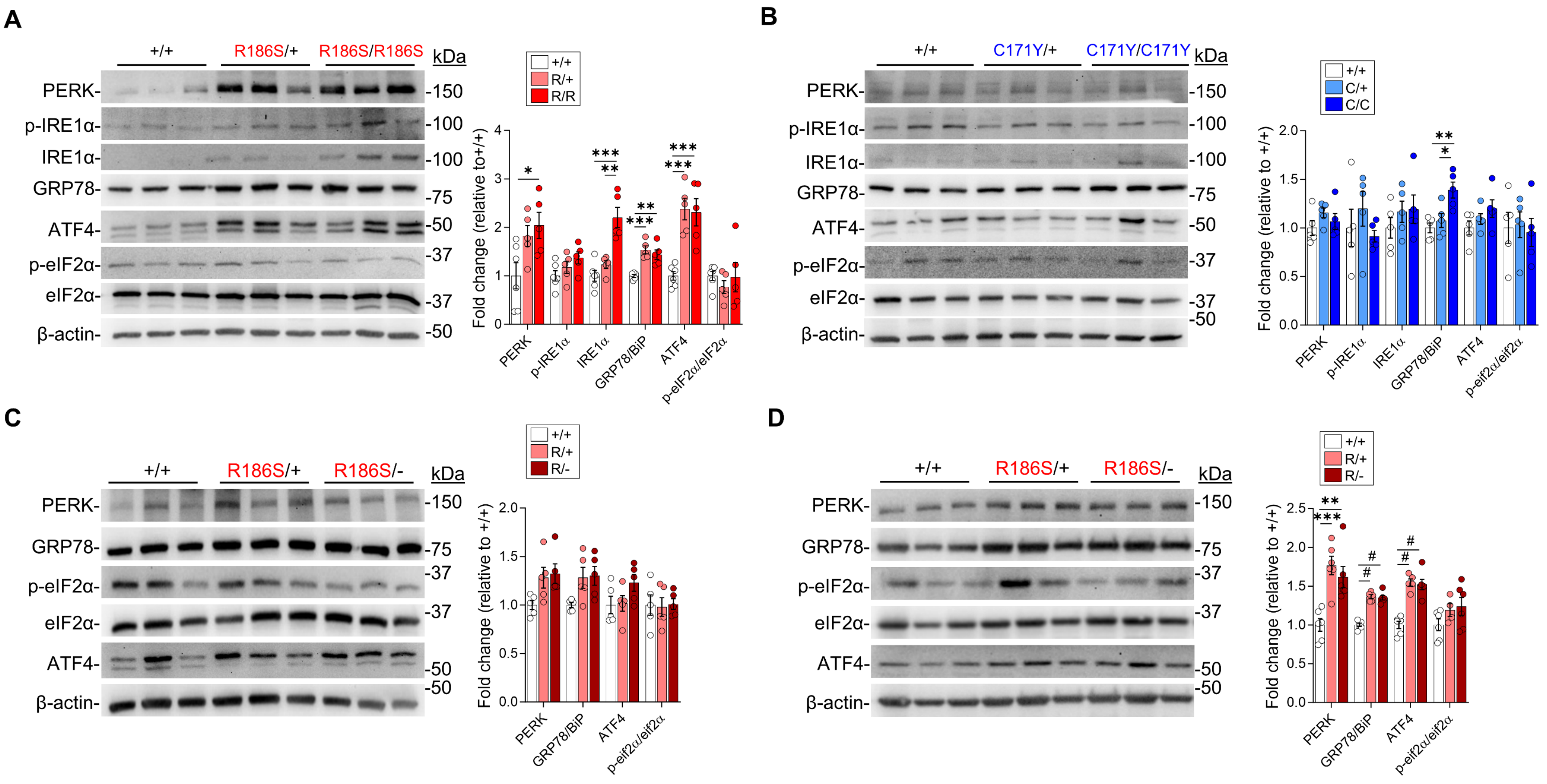


**Appendix Figure S3: Unfolded protein response in kidneys from *Umod* KI mice.**

(**A-B**) Representative immunoblot analysis of ER stress markers in medulla-enriched kidney fractions from 4-months-old R186S (**A**) and C171Y (**B**) mice (n=5 to 6 animals per group).
(**A**) PERK: $^{*}P$=0.0311; IRE1α: $^{**}P$=0.0011, $^{***}P$=0.0001; GRP78/BiP: $^{***}P$=0.0009, $^{**}P$=0.0040; ATF4: $^{***}P$(+/+ vs. R/+)=0.0008, $^{***}P$(+/+ vs. R/R)=0.0012. (**B**) GRP78/BiP: $^{*}P$=0.019, $^{**}P$=0.0049.
(**C-D**) Representative immunoblot analysis of ER stress markers in 1-month-old medulla-enriched kidney fractions (**C**) or 4-months-old total kidney lysates from +/+, R186S/+ and R186S/- mice (n=5 to 6 animals per group). β-actin used as loading control. Densitometry analysis is relative to +/+.
(**D**) PERK: $^{***}P$=0.0007, $^{**}P$=0.0045; $^{\#}P$<0.0001.
Bars indicate mean ± SEM. One-way ANOVA followed by Tukey's post-hoc test.

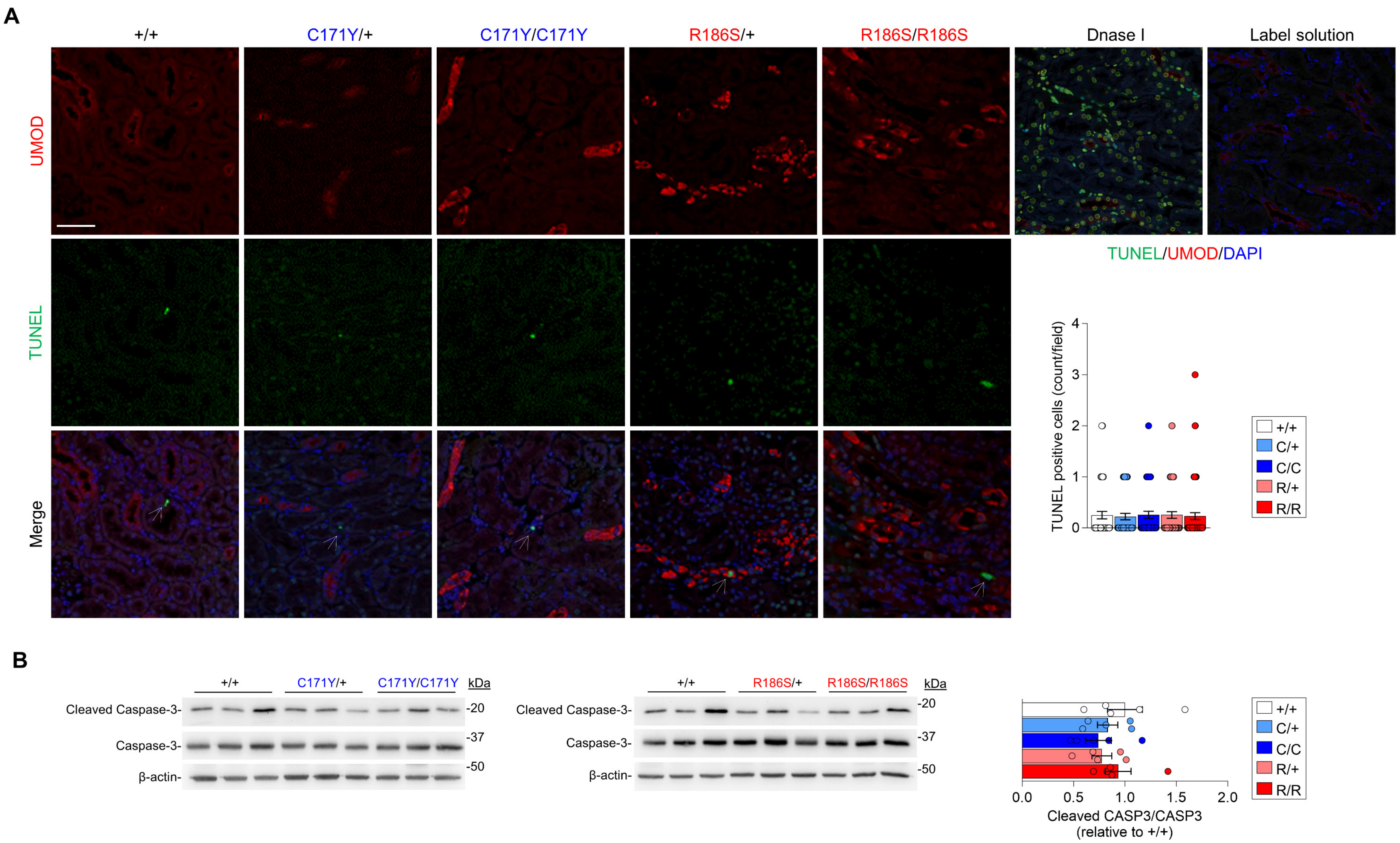


## Appendix Figure S4: Lack of apoptosis or caspase activation in *Umod* KI kidneys.

(**A**) Immunofluorescence analysis of TUNEL (green) and UMOD (red) in kidney sections from 4-month-old *Umod* mutant (n ≥ 45 fields from 3-5 kidneys per condition). Nuclei are counterstained with DAPI (blue). Positive control incubated with DNase I and negative control incubated with label solution shown on right panel. Scale bar: 50 µm. Each point of the quantification represents the number of TUNEL+ cells in one field.

(**B**) Representative immunoblot analysis of caspase-3 (CASP3) and cleaved caspase-3 in whole kidney lysates from 4-month-old +/+, C171Y/+ and C171Y/C171Y mice (left) or +/+, R186S/+ and R186S/R186S mice (right). β-actin used as a loading control. Densitometry analysis is relative to +/+.

Bars indicate mean ± SEM. One-way ANOVA followed by Tukey's post-hoc test, (n = 5 animals per group).

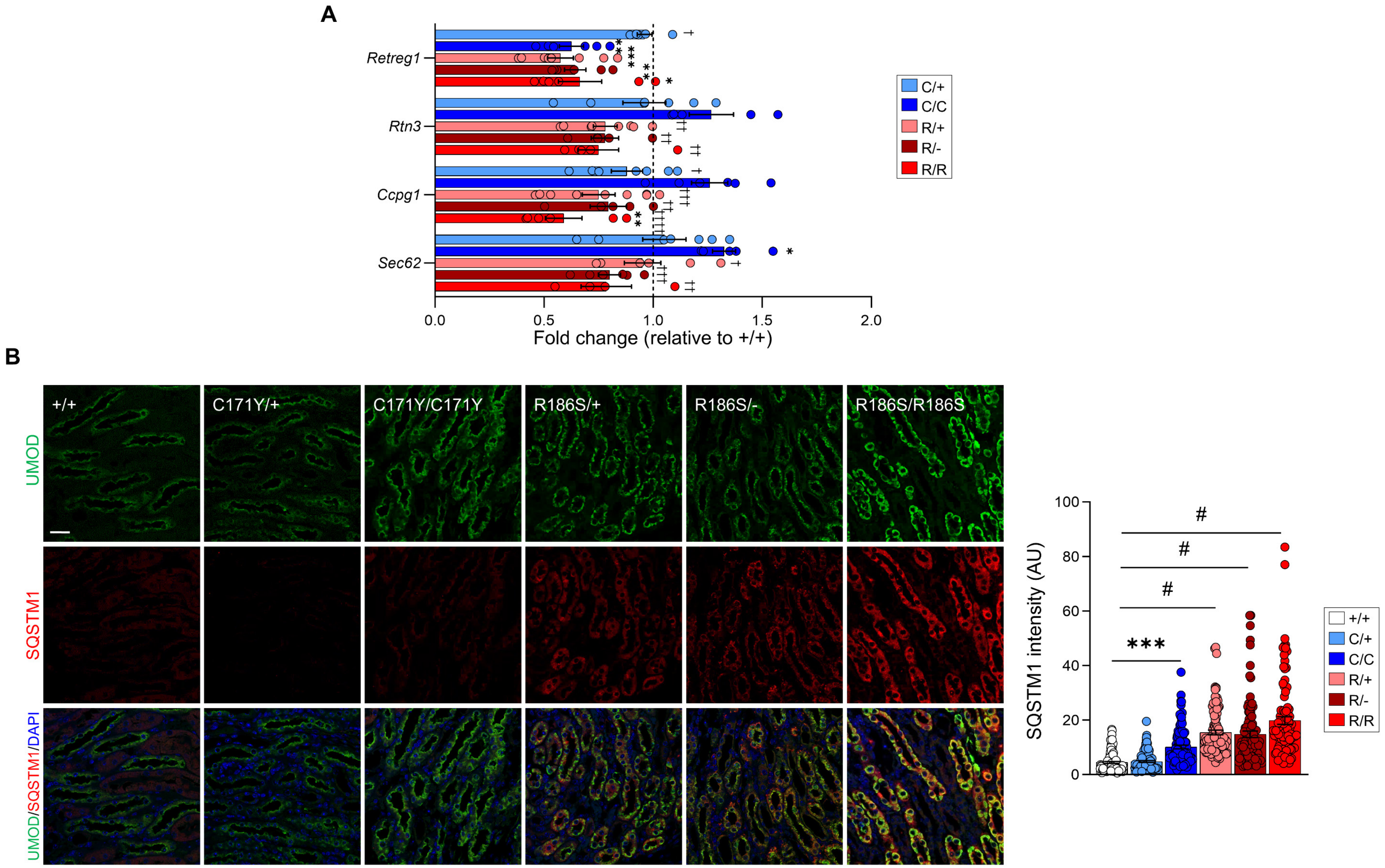


**Appendix Figure S5: Mutant UMOD degradation relies on mutation-specific mechanisms.**

(**A**) RT-qPCR analysis of ER-phagy genes in *Umod* KI kidneys (n=4 to 9 animals per group). Values are expressed as relative to +/+ (black dotted line). *Retreg1*: $^{**}P$(+/+ vs. C/C)=0.0038, $^{***}P$ = 0.0004, $^{**}P$(+/+ vs. R/-)=0.0061, $^{*}P$=0.0113, $^{\dagger}P$=0.0114; *Rtn3*: $^{\dagger\dagger}P$(C/C vs. R/+)=0.0012, $^{\dagger\dagger}P$(C/C vs. R/-)=0.004, $^{\dagger\dagger}P$(C/C vs. R/R)=0.0021; *Ccpg1*: $^{**}P$=0.0059, $^{\dagger}P$=0.0127, $^{\dagger\dagger\dagger}P$=0.0002, $^{\dagger\dagger}P$=0.0015, $^{\dagger\dagger\dagger\dagger}P$<0.0001; *Sec62*: $^{*}P$=0.0494, $^{\dagger}P$=0.016, $^{\dagger\dagger\dagger}P$=0.0006, $^{\dagger\dagger}P$=0.0016.

(**B**) Representative immunofluorescence analysis of UMOD (green) and SQSTM1 (red) on kidney sections from 1-month-old *Umod* KI mice. Nuclei are counterstained with DAPI (blue). Scale bar: 25 µm, n = 100 tubules from 3 kidneys per condition. $^{***}P$=0.0002, $^{\#}P$<0.0001.

Bars indicate mean ± SEM. One-way ANOVA followed by Tukey's post-hoc test.

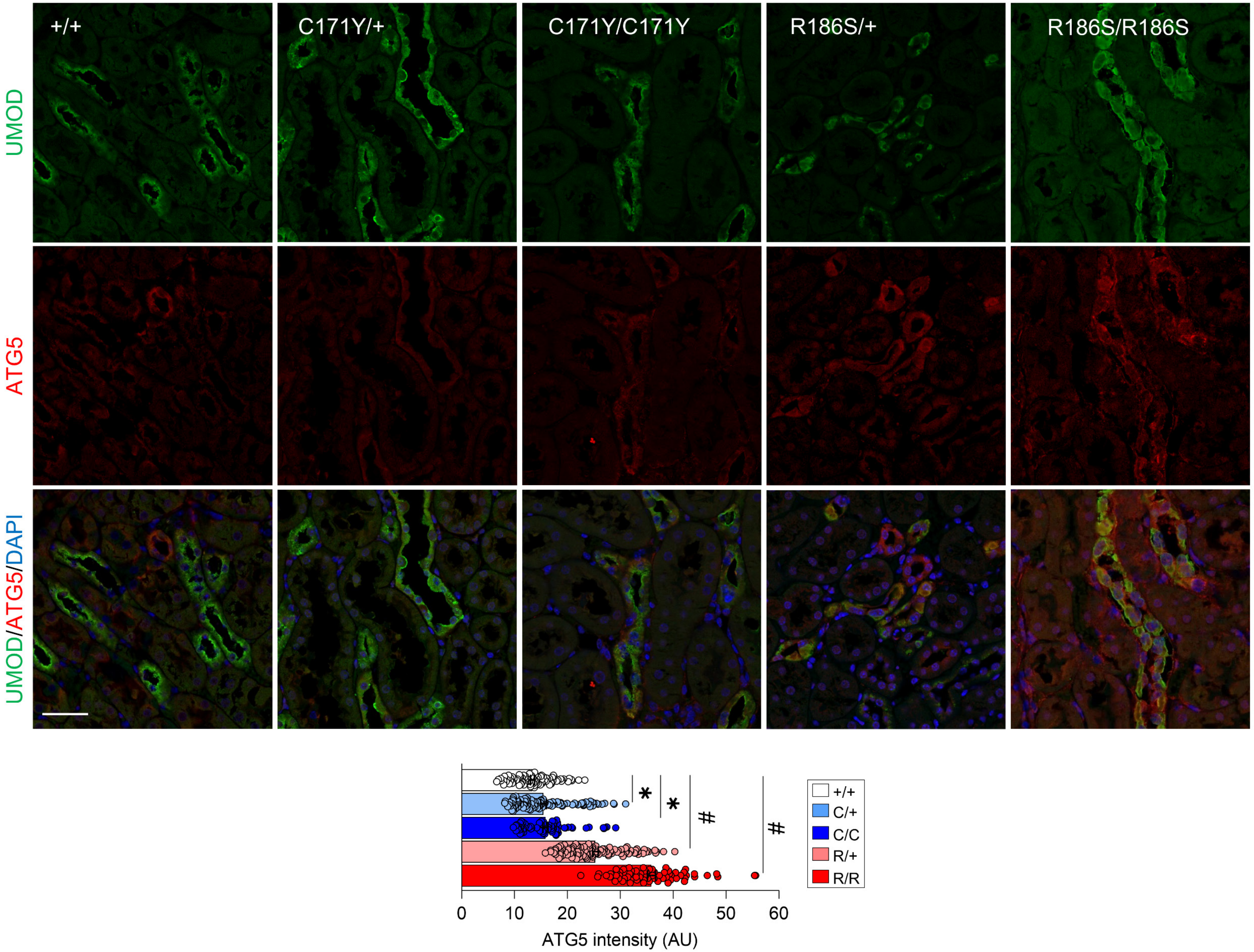


**Appendix Figure S6: Markers of autophagy induction in *Umod* KI mouse kidneys.**

Representative immunofluorescence analysis of UMOD (green) and ATG5 (red) on kidney sections from 4-month-old *Umod* KI mice. Nuclei are counterstained with DAPI (blue). Scale bar: 25 µm, n ≥ 67 tubules from 3 kidneys per condition. Bars indicate mean ± SEM. One-way ANOVA followed by Tukey's post-hoc test, $^{*}P$(+/+ vs. C/+)=0.032, $^{*}P$(+/+ vs. C/C)=0.0153, $^{\#}P$<0.0001.

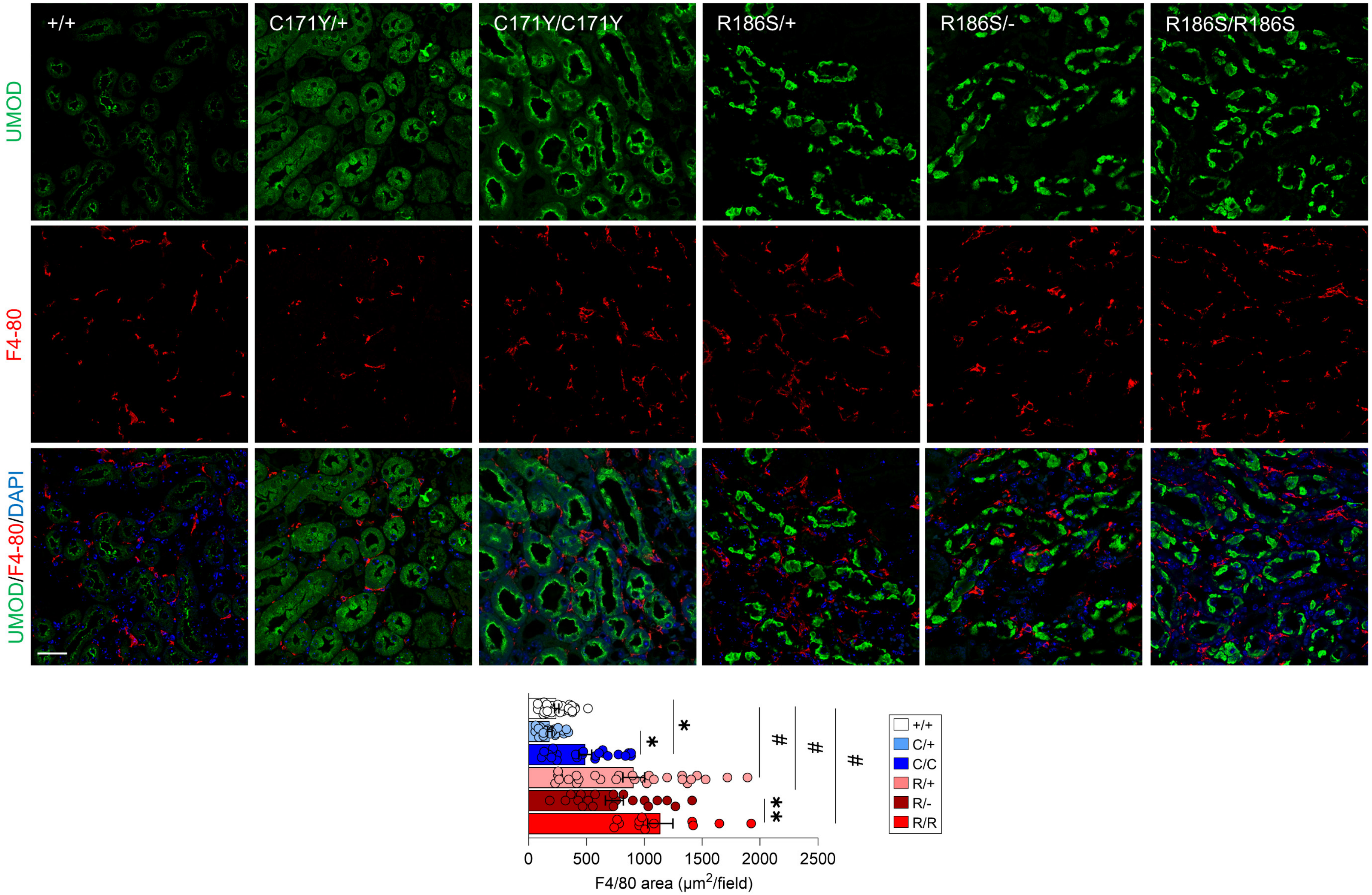


**Appendix Figure S7: Macrophage infiltration in *Umod* KI mouse kidneys.**

Confocal analysis of kidney sections from 4-month mice stained with anti-uromodulin (green) and anti-F4-80 (red). n ≥12 fields from 3 kidneys per condition. Nuclei are stained with DAPI (blue). Scale bar: 25 μm. Bars indicate the mean ± SEM. One-way ANOVA with Tukey's post hoc test, $^{*}P$(+/+ vs. C/C)=0.0342, $^{*}P$(C/+ vs. C/C)=0.0103, $^{**}P$=0.0059, $^{\#}P$<0.0001.

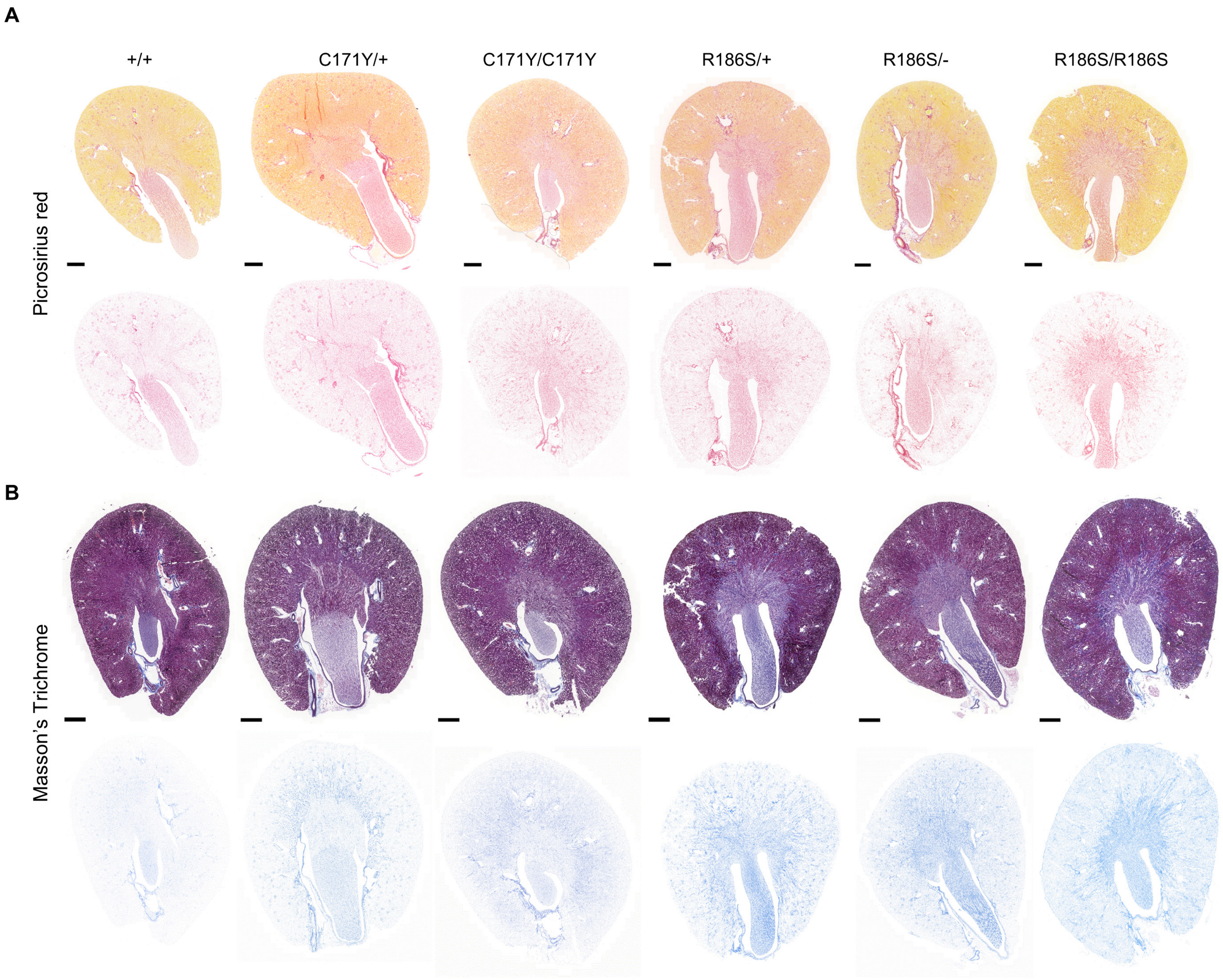
A
+/+
C171Y/+
C171Y/C171Y
R186S/+
R186S/-
R186S/R186S
Picrosirius red
B
Masson's Trichrome

**Appendix Figure S8: Characterization of interstitial fibrosis in *Umod* KI mouse kidneys.**

(**A**-**B**) Representative Picrosirius red (**A**) and Masson's Trichrome (**B**) stainings on kidney sections from 4-month-old *Umod* KI mice (n=3 to 11 animals per group). Deconvoluted collagen signal is shown below each image. Scale bars: 100 µm

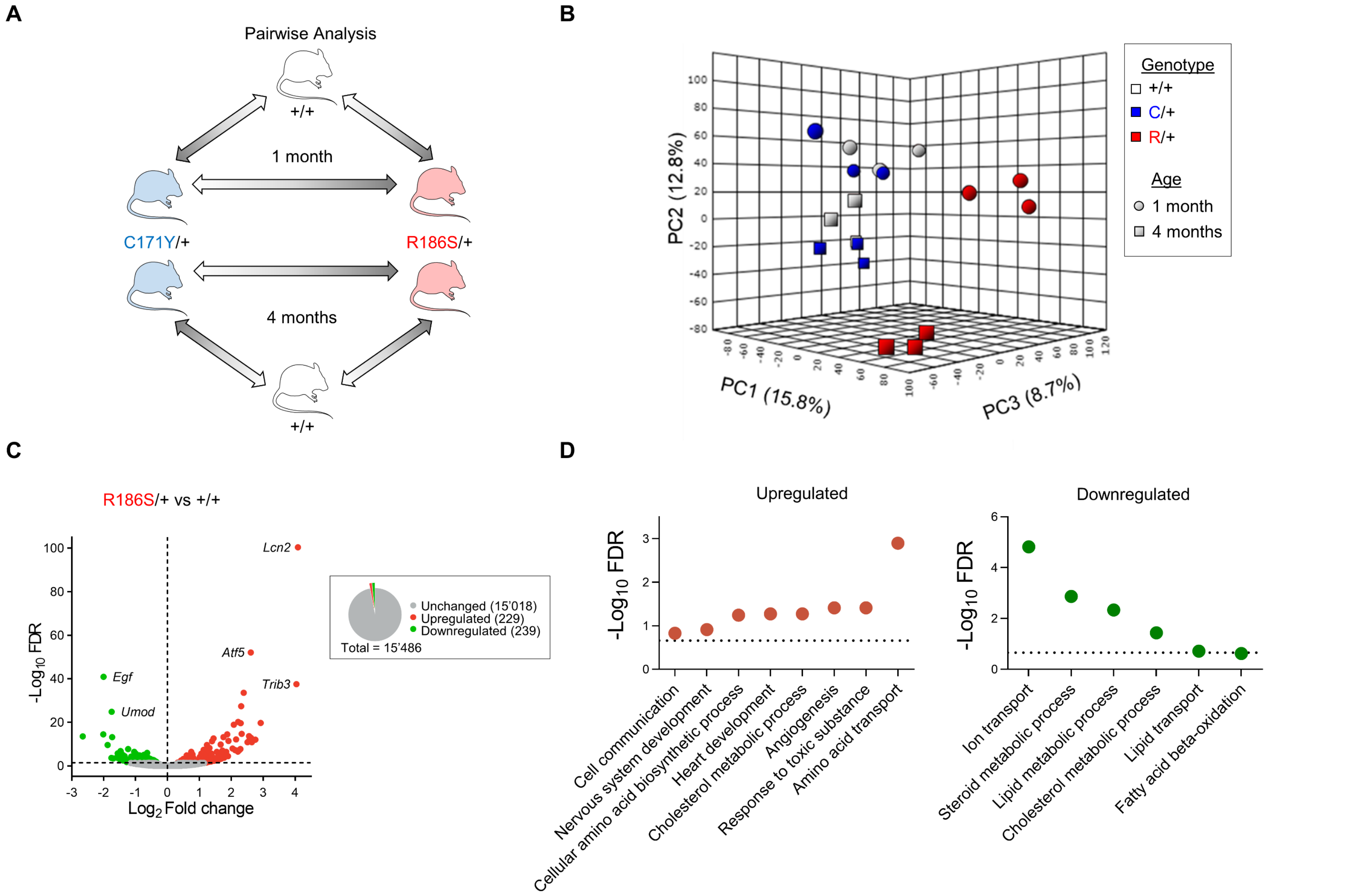


**Appendix Figure S9: RNA-seq analyses in 1-month-old R186S/+ kidneys.**

(**A**) Experimental design for RNA-Seq on whole kidney lysates from 1 and 4 months male *Umod* KI mice.
(**B**) Principal component analysis (PCA) of RNA-Seq data of kidneys from 1-month-old and 4-month-old *Umod* KI mice.
(**C**) Volcano plot showing differently expressed genes (DEGs) between R186S/+ and +/+ 1-month-old kidneys. Genes not significantly changed (FDR > 0.05) are shown in grey, whereas genes that are up- or downregulated in R186S/+ are shown in red and green respectively. The total number of unchanged, up- and downregulated genes are summarized in the pie chart.
(**D**) Over-representation analysis showing up- (red) and downregulated (green) biological processes of gene ontology in 1-month-old R186S/+ kidneys.

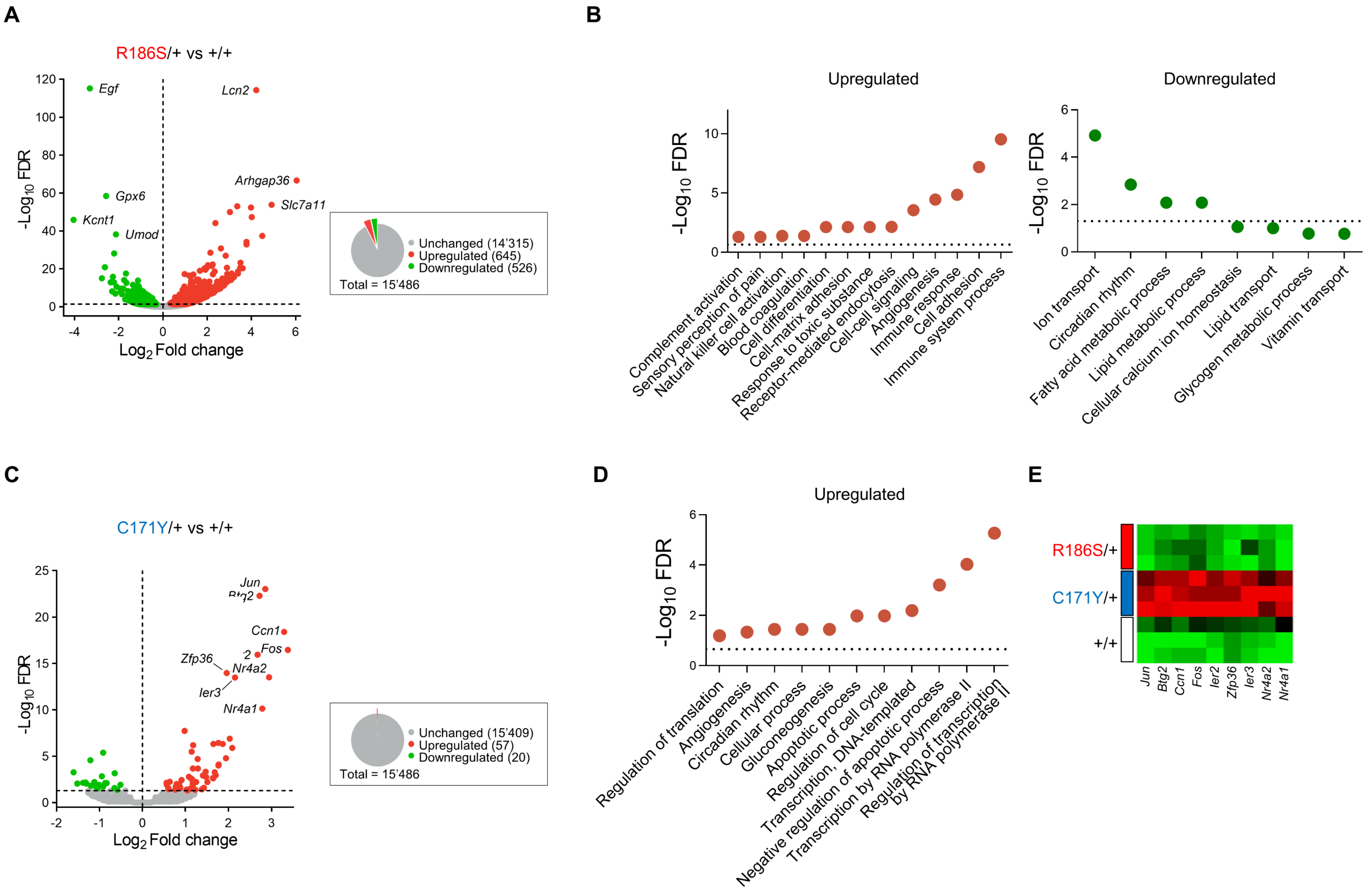


**Appendix Figure S10: Differential expression and affected pathways in 4-month-old *Umod* KI kidneys.**

(**A**) Volcano plot showing differently expressed genes (DEGs) between +/+ and R186S/+ kidneys at 4 months.

(**B**) Over-representation analysis (ORA) showing up- and downregulated biological processes in 4-months-old R186S/+ kidneys.

(**C**) Volcano plot showing DEGs between C171Y/+ and +/+ kidneys at 4 months. Genes not significantly changed (FDR > 0.05) are shown in grey, whereas genes that are up- or downregulated in *Umod* KI are shown in red and green respectively. The total number of unchanged, up- and downregulated genes are summarized in the pie chart.

(**D**) ORA showing upregulated biological processes in 4-month-old C171Y/+ kidneys. No significantly downregulated pathways were identified. (**e**) Heatmap of transcriptional regulation genes upregulated in 4-month-old C171Y/+ kidneys.

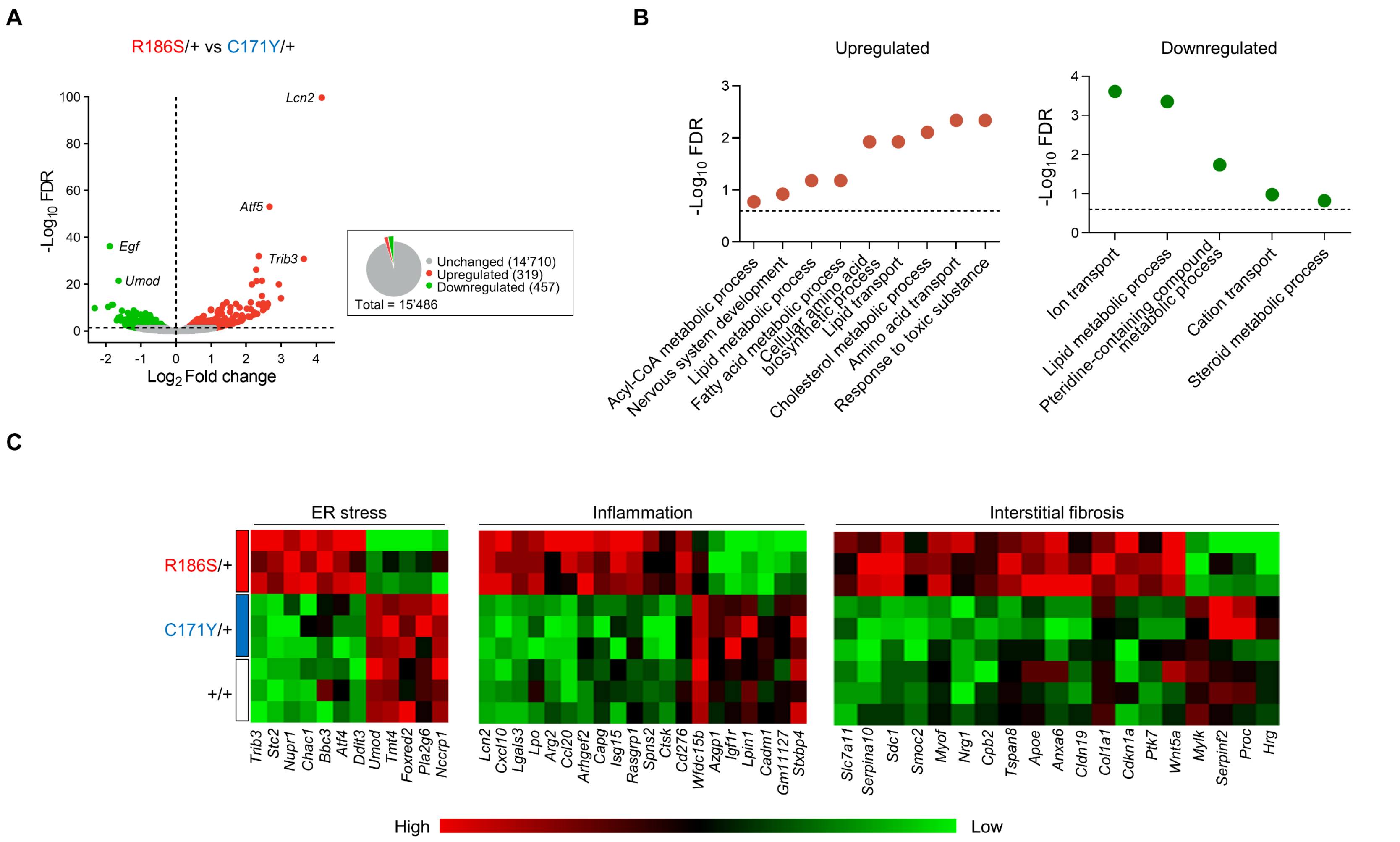


**Appendix Figure S11: Disease progression signature in *Umod* KI kidneys.**

(**A**) Volcano plot showing differently expressed genes (DEGs) between R186S/+ and C171Y/+ kidneys at 1 month. Genes not significantly changed (FDR > 0.05) are shown in grey, whereas genes that are up- or downregulated in R186S/+ are shown in red and green respectively. The total number of unchanged, up- and downregulated genes are summarized in the pie chart.

(**B**) Over-representation analysis (ORA) showing up- (red) and downregulated (green) biological processes in 1-month-old R186S/+ kidneys compared to C171Y/+.

(**C**) Heat map of selected pathways involved in the disease progression of *Umod* KI mice at 1 month. R186S/+ kidneys showed upregulation of ER stress (*Atf4*, *Ddit3*, *Nupr1*, *Trib3*), increased expression of genes associated with inflammation (*Lcn2*, *Lgals3*) and fibrosis (*Serpina10*, *Col1a1*), whereas C171Y/+ kidneys were virtually indistinguishable from +/+.

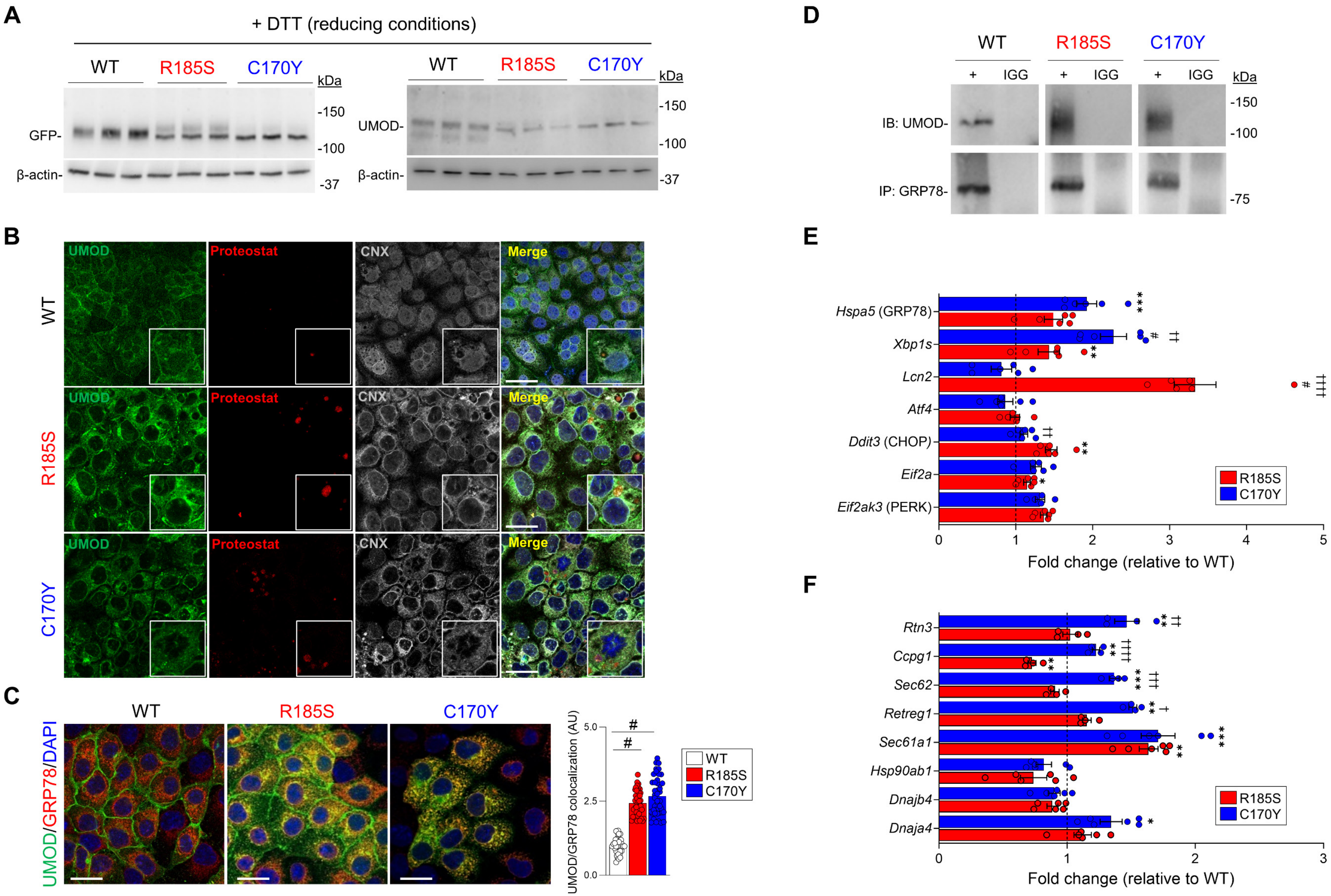
A
+ DTT (reducing conditions)
WT
R185S
C170Y
kDa
-150
-100
-37
GFP-
β-actin-
UMOD-
B
UMOD
Proteostat
CNX
Merge
WT
R185S
C170Y
C
UMOD/GRP78/DAPI
UMOD/GRP78 colocalization (AU)
D
+
IGG
IB: UMOD-
IP: GRP78-
-75
E
Hspa5 (GRP78)
Xbp1s
Lcn2
Atf4
Ddit3 (CHOP)
Eif2a
Eif2ak3 (PERK)
Fold change (relative to WT)
F
Rtn3
Ccpg1
Sec62
Retreg1
Sec61a1
Hsp90ab1
Dnajb4
Dnaja4
Fold change (relative to WT)

**Appendix Figure S12: Distinct UMOD mutations trigger differential ER quality control responses.**

(**A**) Immunoblot analysis of GFP and UMOD in lysates from *UMOD-GFP* cells. Samples were run in reducing conditions. β-actin used as a loading control.

(**B**) Immunofluorescence analysis of UMOD (green), Proteostat (red) and CNX (gray) in *UMOD-GFP* cells. Scale bar: 30 μm.

(**C**) Immunofluorescence analysis of UMOD (green) and GRP78/BiP (red) in *UMOD-GFP* cells. Co-localization is expressed as arbitrary units (AU). Scale bar: 30 μm (n=33 to 44 cells per group), $^{\#}P$<0.0001.

(**D**) Co-immunoprecipitation experiments in *UMOD-GFP* cells showing interaction between UMOD and GRP78/BiP.

(**E**) RT-qPCR analysis of unfolded protein response (UPR) effectors in in *UMOD-GFP* cells. Values are expressed as relative to WT (black dotted line, n = 6 biological replicates). *Hspa5*: $^{***}P$(WT vs. C170Y)=0.0008; *Xbp1s:* $^{\#}P$(WT vs. C170Y)<0.0001, $^{††}P$(C170Y vs. R185S)=0.0018, $^{**}P$(WT vs. R185S)=0.0045; *Lcn2*: $^{\#}P$(WT vs. R185S)<0.0001, $^{††††}P$(C170Y vs. R185S)<0.0001; *Ddit3:* $^{**}P$(WT vs. R185S)=0.0083, $^{††}P$(WT vs. C170Y)=0.0019, *Eif2a:* $^{*}P$(WT vs. R185S)=0.0118.

(**F**) RT-qPCR analysis of protein folding/degradation genes in *UMOD-GFP* cells. Values are expressed as relative to WT (black dotted line) (n =4 to 6 biological replicates). *Rtn3:* $^{**}P$(WT vs. C170Y)=0.002, $^{††}P$=0.0028 (C170Y vs. R185S); *Ccpg1:* $^{**}P$(WT vs. C170Y)=0.0078, $^{††††}P$(C170Y vs. R185S)<0.0001, $^{**}P$(WT vs. R185S)=0.0023; *Sec62*: $^{***}P$(WT vs. C170Y)=0.0008, $^{†††}P$(C170Y vs. R185S)=0.0002; *Retreg1:* $^{**}P$(WT vs. C170Y)=0.002, $^{†}P$(R185S vs. C170Y)=0.017; *Sec61a1:* $^{***}P$(WT vs. C170Y)=0.0005, $^{**}P$(WT vs. R185S)=0.0013; *Dnaja4:* $^{*}P$(WT vs. C170Y)=0.0209.

Bars indicate mean ± SEM. One-way ANOVA followed by Tukey's post-hoc test.

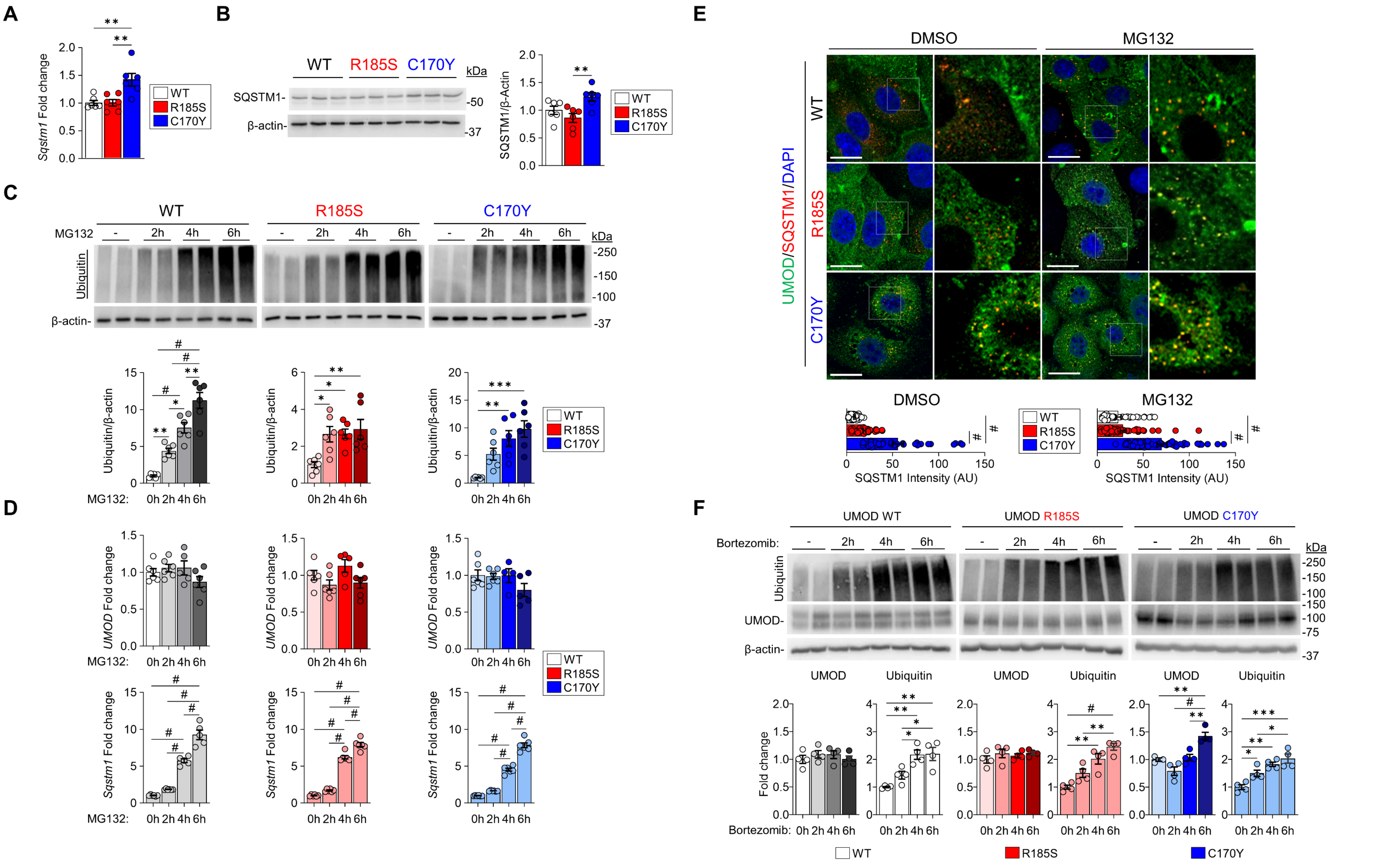
A
B
C
D
E
F
Sqstm1 Fold change
WT
R185S
C170Y
SQSTM1-
β-actin-
kDa
SQSTM1/β-Actin
MG132
Ubiquitin
Ubiquitin/β-actin
MG132:
0h 2h 4h 6h
UMOD Fold change
Sqstm1 Fold change
DMSO
UMOD/SQSTM1/DAPI
SQSTM1 Intensity (AU)
UMOD WT
UMOD R185S
UMOD C170Y
Bortezomib:
UMOD-
UMOD
Fold change

**Appendix Figure S13: Proteasomal inhibition induces mutant UMOD accumulation.**

(**A**) RT-qPCR analysis of *Sqstm1* gene in *UMOD-GFP* cells. Values are expressed as relative to WT cells (n=6 biological replicates), $^{**}$*P*(R185S vs. C170Y)=0.0058, $^{**}$*P*(WT vs. C170Y)=0.0056.

(**B**) Immunoblot analysis of SQSTM1 in *UMOD-GFP* cells. β-actin used as a loading control. Densitometry analysis relative to WT cells (n=6 biological replicates), $^{**}$*P*=0.0084.

(**C**) Immunoblot analysis of ubiquitin in *UMOD-GFP* cell lysates following MG123 time course. Densitometry analysis relative to untreated cells (n = 6 biological replicates). WT: $^{**}$*P*(0h vs. 2h)=0.0096, $^{*}$*P*=0.0177, $^{**}$*P*(4h vs. 6h)= 0.0041; R185S: $^{*}$*P*(0h vs. 2h)=0.0177, $^{*}$*P*(0h vs. 4h)=0.0216, $^{**}$*P*=0.0079; C170Y: $^{**}$*P*=0.002, $^{***}$*P*=0.0002.

(**D**) RT-qPCR analysis of *UMOD* and *Sqstm1* genes in *UMOD-GFP* cells following time-dependent MG132 treatment. Values are relative to untreated (0h) cells (n ≥4 biological replicates), $^{\#}$*P*<0.0001.

(**E**) Representative immunofluorescence of UMOD (green) and SQSTM1 (red) in *UMOD-GFP* cells. Nuclei counterstained with DAPI (blue). Scale bar: 15 μm (n = 30 cells per group), $^{\#}$*P*<0.0001.

(**F**) Immunoblot analysis of ubiquitin, UMOD in *UMOD-GFP* cell lysates following Bortezomib time-course. β-actin used as a loading control. Densitometry analysis relative to fed cells (n ≥4 biological replicates). WT, Ubiquitin: $^{**}$*P*(0h vs. 4h)=0.0013, $^{**}$*P*(0h vs. 6h)=0.0011, $^{*}$*P*(2h vs. 4h)=0.0322, $^{*}$*P*(4h vs. 6h)=0.0267; R185S, Ubiquitin: $^{**}$*P*(0h vs. 4h)=0.0013, $^{**}$*P*(2h vs. 6h)=0.0022; C170Y, UMOD: $^{**}$*P*(0h vs. 6h)=0.0045, $^{**}$*P*(4h vs. 6h)=0.0051; Ubiquitin: $^{*}$*P*(0h vs. 2h)=0.0429, $^{**}$*P*=0.0014, $^{***}$*P*=0.0002, $^{*}$*P*(2h vs. 6h)=0.031; $^{\#}$*P* <0.0001.

Bars indicate mean ± SEM. One-way ANOVA followed by Tukey’s post-hoc test.

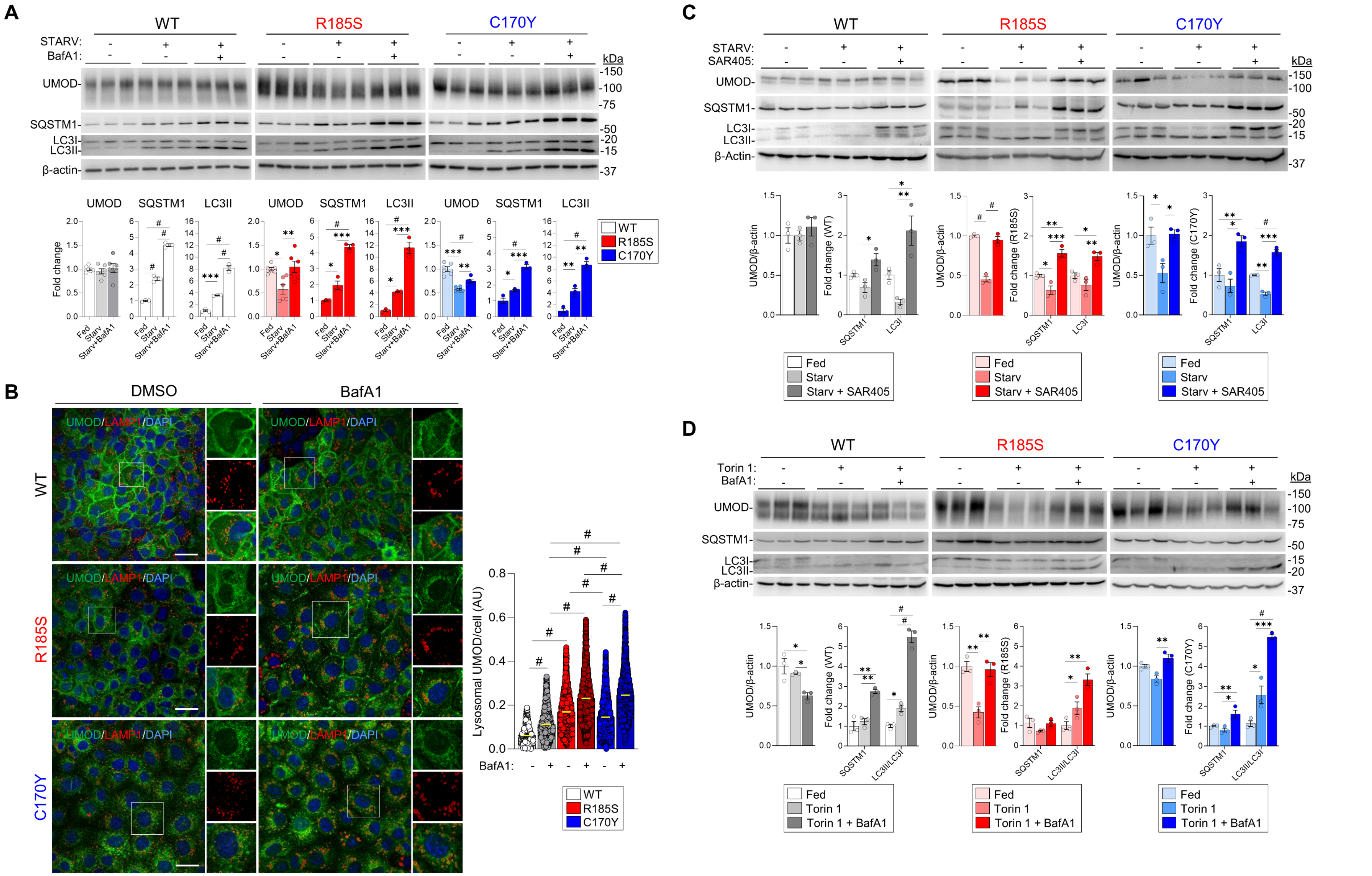
A
WT
R185S
C170Y
STARV:
BafA1:
kDa
UMOD-
SQSTM1-
LC3I-
LC3II-
β-actin-
-150
-100
-75
-50
-20
-15
-37
UMOD
SQSTM1
LC3II
Fold change
Fed
Starv
Starv+BafA1
WT
R185S
C170Y
B
DMSO
BafA1
UMOD/LAMP1/DAPI
Lysosomal UMOD/cell (AU)
BafA1:
WT
R185S
C170Y
C
SAR405:
UMOD-
SQSTM1-
LC3I-
LC3II-
β-Actin-
UMOD/β-actin
Fold change (WT)
Fold change (R185S)
Fold change (C170Y)
SQSTM1
LC3I
Fed
Starv
Starv + SAR405
D
Torin 1:
BafA1:
LC3II/LC3I
Fed
Torin 1
Torin 1 + BafA1

## Appendix Figure S14: Autophagy modulation impacts on mutant UMOD levels.

(**A**) Immunoblot analysis of UMOD, SQSTM1 and LC3 in *UMOD-GFP* cell lysates following starvation and Bafilomycin A1 treatment. β-actin used as a loading control. Densitometry analysis relative to fed (n=3 biological replicates), WT, LC3II: $^{***}P$=0.0007; R185S, UMOD: $^{*}P$=0.0136,$^{**}P$=0.0067; SQSTM1: $^{*}P$=0.0175, $^{***}P$=0.0002; LC3II: $^{*}P$=0.0164, $^{***}P$=0.0002; C170Y, UMOD: $^{***}P$=0.0003,$^{**}P$ = 0.0096; SQSTM1: $^{*}P$=0.0282, $^{***}P$=0.0004; LC3II: $^{**}P$(Fed vs. Starv)=0.006, $^{**}P$(Starv vs. Starv+ BafA1)=0.0013; $^{\#}P$<0.0001.

(**B**) Representative immunofluorescence of UMOD (green) and LAMP1 (red) in *UMOD-GFP* cells following BafA1 treatment. Nuclei counterstained with DAPI (blue). Scale bar: 15 µm (n ≥ 20 cells/field, 5 fields per condition), $^{\#}P$<0.0001.

(**C**) Immunoblot analysis of UMOD, SQSTM1 and LC3 in *UMOD-GFP* cell lysates following SAR405 treatment. Densitometry analysis relative to fed (n=3 biological replicates).
WT, SQSTM1: $^{*}P$=0.0129; LC3I: $^{**}P$=0.0033, $^{*}P$=0.0296; R185S, SQSTM1: $^{*}P$=0.0486, $^{**}P$=0.0073, $^{***}P$=0.0005; LC3I: $^{**}P$=0.0070, $^{*}P$=0.0410; C170Y, UMOD: $^{*}P$(Fed vs. Starv)=0.0342, $^{*}P$(Starv vs. Starv + SAR405)=0.0283; SQSTM1: $^{**}P$=0.055, $^{*}P$=0.0198; LC3I: $^{**}P$=0.0011, $^{***}P$=0.0004; $^{\#}P$<0.0001.

(**D**) Immunoblot analysis of UMOD, SQSTM1 and LC3 in *UMOD-GFP* cell lysates following Torin 1 and BafA1 treatment. Densitometry analysis relative to fed cells (n=3 biological replicates). WT, UMOD: $^{*}P$(Fed vs. Torin 1)=0.0118, $^{*}P$(Torin 1 vs. Torin 1+BafA1)=0.0399; SQSTM1: $^{**}P$(Fed vs. Torin 1+BafA1)=0.001, $^{**}P$(Torin 1 vs. Torin 1+BafA1)=0.0022; LC3II/I: $^{*}P$=0.0476; R185S, UMOD: $^{**}P$(Fed vs. Torin 1)=0.0035, $^{**}P$(Torin 1 vs. Torin 1+BafA1)=0.005; LC3II/I: $^{*}P$=0.023, $^{**}P$=0.0023; C170Y, UMOD: $^{**}P$=0.0096; SQSTM1: $^{*}P$=0.0336, $^{**}P$=0.0094; LC3II/I: $^{*}P$=0.0229, $^{***}P$=0.0007; $^{\#}P$< 0.0001.

Bars indicate mean ± SEM. One-way ANOVA followed by Tukey's post-hoc test.

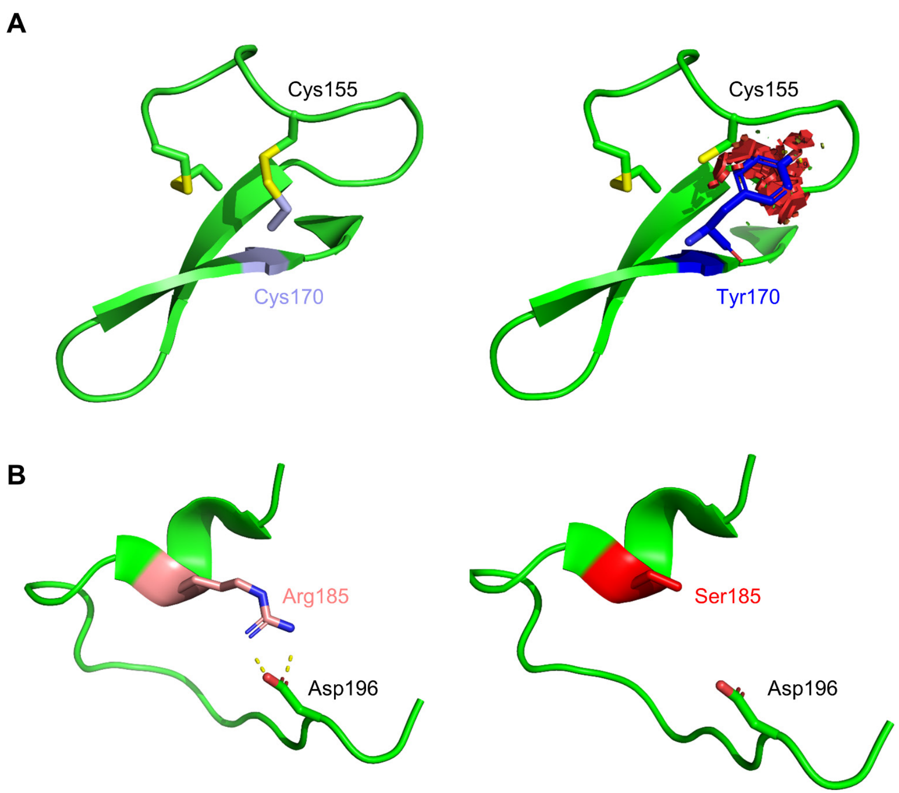


**Appendix Figure S15: Structural modelling of UMOD mutations**

(**A**, **B**) Modeling of the UMOD p.Cys170Tyr (**A**) and p.Arg185Ser (**B**) mutants. Clashes in the structure are represented by red dots and are visible only in the p.Cys170Tyr mutant. Models were generated with PyMOL (Schrödinger LLC).

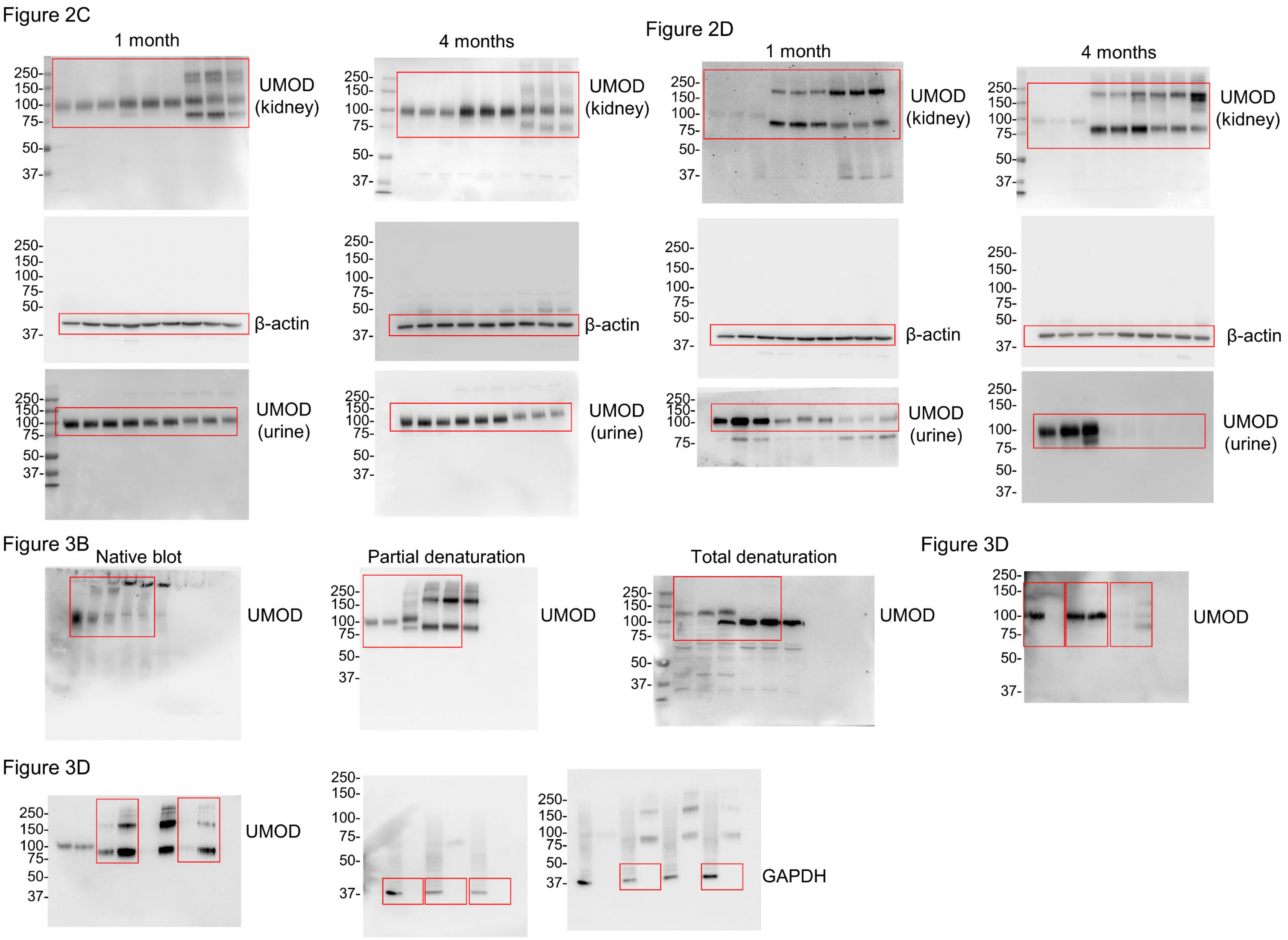


**Appendix Figure S16: Uncropped Western blot membranes.**

Figure 4C

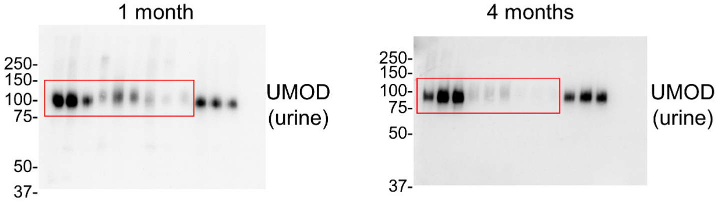


Figure 4D

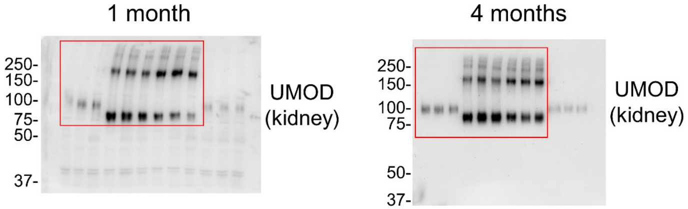


Figure 4D

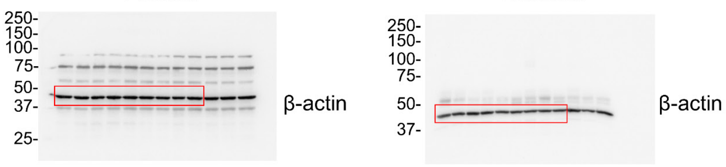


Figure 4E

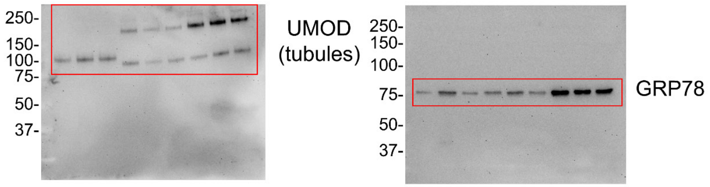


Figure 4E

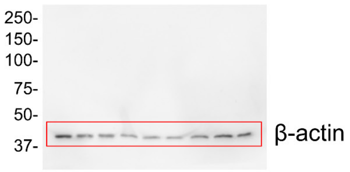


Figure 5A

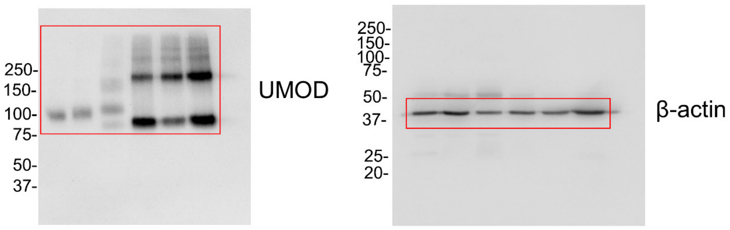


Figure 7A

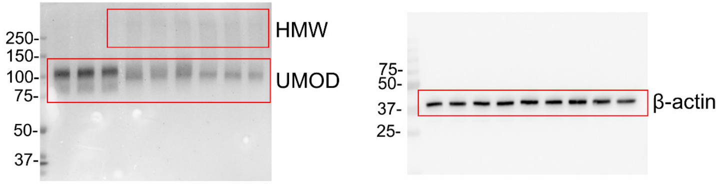


Figure 7B

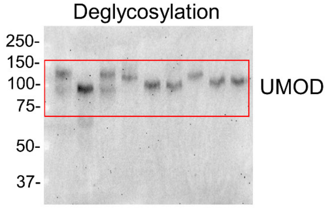


Figure 7C

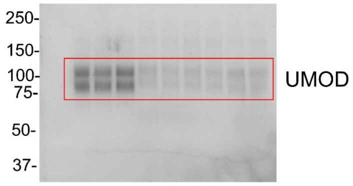


**Appendix Figure S16 (continued).**

Figure 7G

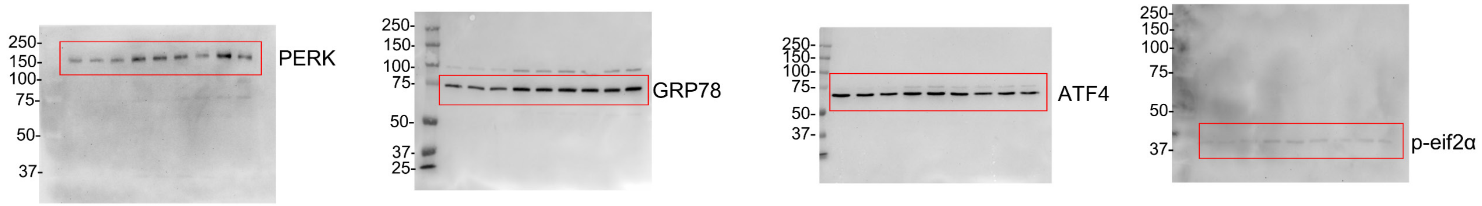


Figure 7G

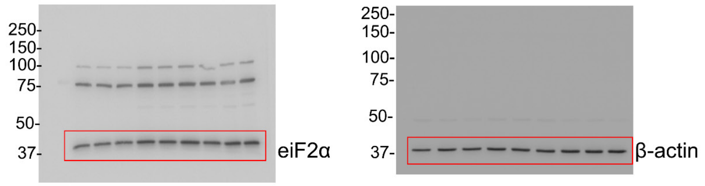


Figure 8B

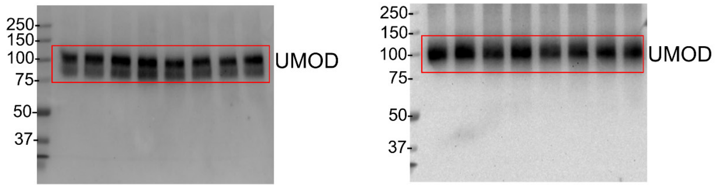


Figure 8B

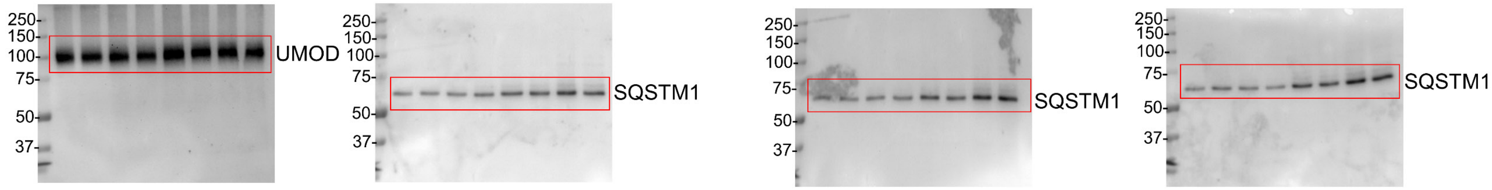


Figure 8B

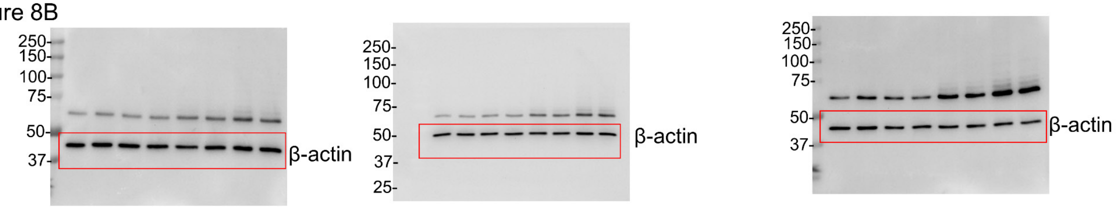


**Appendix Figure 16 (continued).**

Figure 8D

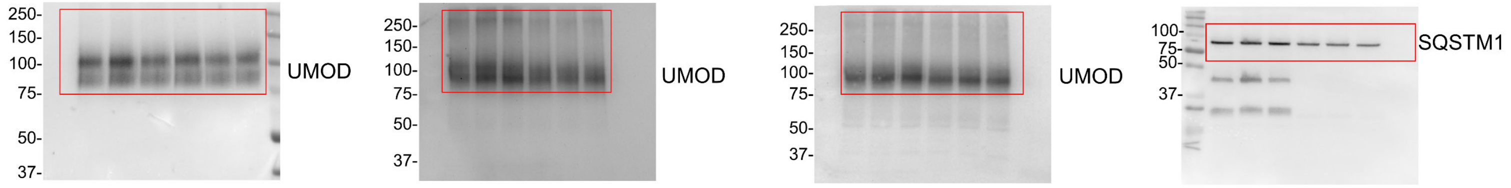


Figure 8D

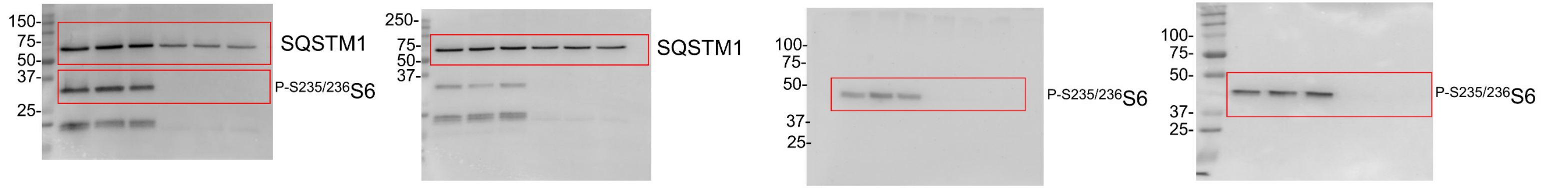


Figure 8D

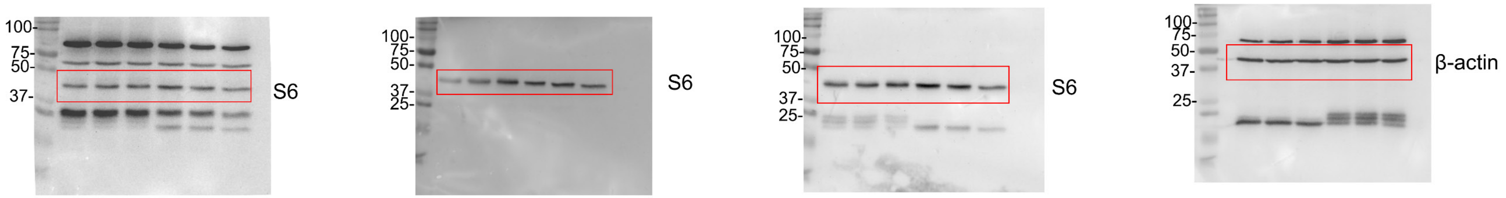


Figure 8D

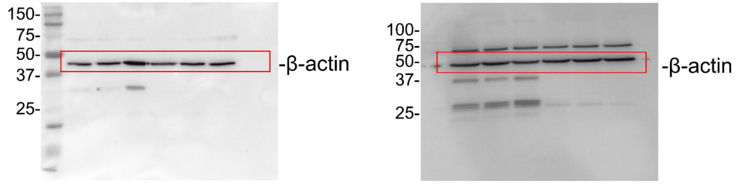


**Appendix Figure 16 (continued).**

**Appendix Table S1: Clinical characteristics of p. (Arg185Ser) ADTKD-UMOD family.**

| | Gender | CKD (age) | Kidney failure (age) | Hyperuricemia (age) | Gout (age) |
|---|---|---|---|---|---|
| p.R185S III.1 | F | Y | Y (NA) | Y | Y (40y) |
| p.R185S III.2 | F | Y | Y (50y) | NA | NA |
| p.R185S IV.1 | M | Y | Y (43y) | Y | Y |
| p.R185S IV.2 | M | Y (32y) | Y (36y) | Y | Y (32y) |
| p.R185S IV.3 | M | Y (19y) | Y (28y) | Y | Y (9y) |
| p.R185S IV.4 | F | Y | N (41y) | Y (34y) | N |
| p.R185S IV.5 | F | Y | Y (38y) | Y | Y (18y) |
| p.R185S IV.6 | M | Y (38y) | Y (42y) | Y | Y (26y) |
| p.R185S V.1 | M | Y | N (24y) | NA | NA |
| p.R185S V.2 | M | Y | N (34y) | Y | Y (25y) |

M, male; F, female; Y, yes; N, no; NA, not available; CKD, chronic kidney disease; eGFR, estimated glomerular filtration rate.

**Appendix Table S2: Clinical characteristics of p. (Cys170Tyr) ADTKD-*UMOD* families.**

| F1 | Gender | CKD (age) | Kidney failure (age) | Hyperuricemia | Gout (age) |
|---|---|---|---|---|---|
| p.C170Y I.1 | M | Y | Y (63y) | Y | Y |
| p.C170Y II.1 | M | Y (41y) | Y (58y; post-nephrectomy)* | Y | Y (55y) |
| p.C170Y III.1 | F | Y (43y) | N | Y | N |
| p.C170Y III.2 | F | Y | N (28y) | NA | NA |
| p.C170Y IV.1 | F | N (12y) | N | N | N |

| F2 | Gender | CKD (age) | Kidney failure (age) | Hyperuricemia | Gout (age) |
|---|---|---|---|---|---|
| p.C170Y I.1 | M | Y | Y (69y) | Y | Y |
| p.C170Y II.1 | F | Y | N (73y: eGFR 33mL/min; 86y: no ESKD) | Y | N |
| p.C170Y II.2 | F | Y | Y (84y) | Y | N |
| p.C170Y III.1 | F | Y (33y) | N (54y) | Y | N |

M, male; F, female; Y, yes; N, no; NA, not available; CKD, chronic kidney disease; eGFR, estimated glomerular filtration rate.
* Nephrectomy for clear cell carcinoma – abundant hematuria

**Appendix Table S3: In silico analysis of selected *UMOD* missense variants.**

| Genomic coordinates (GRCh38) | Nucleotide change | Predicted amino acid change | Control & patient databases* | Pathogenicity predictions¥ | Comment | ACMG classification (PMID 25741868) |
|---|---|---|---|---|---|---|
| 16:20348792:C:T | c.509G>A | p.(Cys170Tyr) | 0¶ | Pathogenic computational verdict (7 path. vs. 4 ben. predictions), REVEL metascore: 0.63† | Not in ClinVar; located between EGF-like 3 domain and D8C UniProt classifies this variant as Pathogenic, associated with Tubulointerstitial kidney disease, autosomal dominant, 1. | **Likely Pathogenic (PM2, PP2, PP3, PP5, PP1)** |
| 16:20348748:G:T | c.553C>A | p.(Arg185Ser) | 0 | Pathogenic computational verdict (9 path. vs. 2 ben. predictions), REVEL metascore: 0.84† | Not in ClinVar; located between EGF-like 3 domain and D8C; inside a mutational hot-spot# UniProt classifies this variant as Pathogenic, associated with Tubulointerstitial kidney disease, autosomal dominant, 1. | **Pathogenic (PM1, PM5, PM2, PP2, PP3, PP5, PP1)** |

UMOD transcript: NM_001008389.3

* Includes the Genome Aggregation Database (gnomAD) (Karczewski *et al*, 2020), the UK Biobank (Van Hout *et al*, 2020), the Genomics England 100,000 Genomes Project (Turro *et al*, 2020) and the UK Rare Disease Registry (RaDaR) (https://ukkidney.org/rare-renal/radar).

¶ Has been reported before in 1 additional French family with slowly progressive CKD (2 individuals with 72y & 73y not yet in ESKD) (Dahan *et al*, 2003)

¥ Generated using Varsome (Kopanos et al, 2019).

† REVEL, rare exome variant ensemble learner (7) (a score > 0.75 corresponds to a sensitivity of ~0.5 and a specificity of ~0.95 for pathogenic variants in the training dataset).

#Hot-spot of length 17 amino-acids has 9 missense/in-frame/non-synonymous variants (5 pathogenic, 3 uncertain, and 1 benign), which qualifies as a dense hot-spot (Ioannidis et al, 2016).

Abbreviations: path., pathogenic; ben., benign; EGF-like, epidermal growth factor-like domain; D8C, cysteine-rich domain of unknown function.

**Appendix Table S4: Clinical and biochemical parameters of *Umod*$^{C171Y}$ mice.**

| | 1 month | | | 4 months | | |
|---|---|---|---|---|---|---|
| **Parameter** | *Umod*$^{+/+}$ n=9 | *Umod*$^{C17Y/+}$ n=16 | *Umod*$^{C171Y/C171Y}$ n=5 | *Umod*$^{+/+}$ n=5 | *Umod*$^{C17Y/+}$ n=11 | *Umod*$^{C171Y/C171Y}$ n=7 |
| Body weight (BW), g | 19.7 ± 0.7 | 20.2 ± 0.6 | 19.1 ± 1.5 | 25.6 ± 1.5 | 28.3 ± 1.0 | 27.4 ± 1.5 |
| Water intake, $\mu L \cdot min^{-1} \cdot g\ BW^{-1}$ | 0.23 ± 0.03 | 0.27 ± 0.01 | 0.29 ± 0.02 | 0.17 ± 0.02 | 0.14 ± 0.01 | 0.09 ± 0.02* |
| **Urine** | | | | | | |
| Diuresis, $\mu L \cdot min^{-1} \cdot g\ BW^{-1}$ | 0.07 ± 0.02 | 0.05 ± 0.01 | 0.04 ± 0.01 | 0.05 ± 0.01 | 0.05 ± 0.01 | 0.05 ± 0.01 |
| $Na^{+}$, $g \cdot g\ creat^{-1}$ | 5.6 ± 0.3 | 5.7 ± 0.2 | 7.2 ± 0.7 | 4.5 ± 0.6 | 6.6 ± 0.4* | 4.9 ± 0.7 |
| $K^{+}$, $g \cdot g\ creat^{-1}$ | 19.5 ± 1.0 | 20.1 ± 0.6 | 23.6 ± 2.0 | 15.7 ± 0.9 | 19.2 ± 1.0 | 15.7 ± 1.0 |
| $Cl^{-}$, $g \cdot g\ creat^{-1}$ | 14.4 ± 0.8 | 14.3 ± 0.4 | 17.9 ± 1.0* | 9.8 ± 0.8 | 12.6 ± 1.0 | 10.1 ± 0.8 |
| $Ca^{2+}$, $g \cdot g\ creat^{-1}$ | 0.14 ± 0.02 | 0.14 ± 0.01 | 0.17 ± 0.02 | 0.17 ± 0.04 | 0.09 ± 0.01* | 0.17 ± 0.02 |
| $Mg^{2+}$, $g \cdot g\ creat^{-1}$ | 1.32 ± 0.06 | 1.24 ± 0.08 | 1.39 ± 0.04 | 0.95 ± 0.04 | 1.03 ± 0.03 | 1.02 ± 0.07 |
| Creatinine, $mg \cdot dL^{-1}$ | 44 ± 3 | 43 ± 2 | 32 ± 3* | 41 ± 5 | 32 ± 2 | 35 ± 5 |
| $FE_{UA}$,% | 1.16 ± 0.26 | 0.97 ± 0.12 | 1.41 ± 0.34 | 0.59 ± 0.11 | - | - |
| Osmolality, $mOsm \cdot kg\ H_2O^{-1}$ | 1854 ± 85 | 1816 ± 93 | 1648 ± 132 | 1476 ± 184 | 1364 ± 109 | 1284 ± 188 |
| **Plasma** | n=9 | n=21 | n=9 | n=16 | n=20 | n=24 |
| $Na^{+}$, $mmol \cdot L^{-1}$ | 147 ± 2 | 146 ± 1 | 148 ± 1 | 147 ± 1 | 149 ± 1 | 149 ± 1 |
| $Cl^{-}$, $mmol \cdot L^{-1}$ | 108 ± 1 | 108 ± 2 | 108 ± 2 | 111 ± 1 | 110 ± 0.8 | 111 ± 1 |
| $Ca^{2+}$, $mmol \cdot L^{-1}$ | 2.8 ± 0.04 | 2.8 ± 0.05 | 2.8 ± 0.6 | 2.5 ± 0.05 | 2.5 ± 0.03 | 2.50 ± 0.3 |
| Creatinine, $mg \cdot dL^{-1}$ | 0.14 ± 0.02 | 0.13 ± 0.01 | 0.15 ± 0.03 | 0.12 ± 0.01 | 0.13 ± 0.01 | 0.15 ± 0.01 |
| BUN, $mg \cdot dL^{-1}$ | 21 ± 2 | 19 ± 1 | 21 ± 1 | 20 ± 2 | 25 ± 1* | 29 ± 1*** |
| Uric acid, $mg \cdot dL^{-1}$ | 6.4 ± 0.4 | 5.9 ± 0.3 | 5.2 ± 0.4 | 5.2 ± 0.5 | 4.6 ± 0.4 | 4.0 ± 0.3 |
| Osmolality, $mOsm \cdot kg\ H_2O^{-1}$ | 351 ± 3 | 343 ± 3 | 347 ± 4 | 335 ± 3 | 342 ± 2* | 343 ± 2 |

Values are presented as average ± SEM. *$P < 0.05$, ***$P < 0.001$, versus age matched *Umod*$^{+/+}$ mice. n: number of animals, FEUA: fractional excretion of uric acid, BUN: blood urea nitrogen.

**Appendix Table S5: Clinical and biochemical parameters of *Umod*$^{R186S}$ mice.**

| Parameter | 1 month *Umod*$^{+/+}$ n=15 | 1 month *Umod*$^{R186S/+}$ n=18 | 1 month *Umod*$^{R186S/R186S}$ n=12 | 4 months *Umod*$^{+/+}$ n=10 | 4 months *Umod*$^{R186S/+}$ n=13 | 4 months *Umod*$^{R186S/R186S}$ n=7 |
|---|---|---|---|---|---|---|
| Body weight (BW), g | 16.5 ± 0.6 | 16.4 ± 0.6 | 16.4 ± 0.6 | 26.3 ± 1.4 | 23.3 ± 1.0 | 26.5 ± 0.9 |
| Water intake, $\mu L \cdot min^{-1} \cdot g\ BW^{-1}$ | 0.29 ± 0.02 | 0.27 ± 0.02 | 0.28 ± 0.03 | 0.15 ± 0.01 | 0.31 ± 0.02*** | 0.44 ± 0.04*** |
| **Urine** | | | | | | |
| Diuresis, $\mu L \cdot min^{-1} \cdot g\ BW^{-1}$ | 0.041 ± 0.006 | 0.059 ± 0.009 | 0.14 ± 0.04* | 0.046 ± 0.007 | 0.12 ± 0.01*** | 0.23 ± 0.02*** |
| $Na^+$, $g \cdot g\ creat^{-1}$ | 5.8 ± 0.6 | 6.3 ± 0.7 | 6.4 ± 0.8 | 5.6 ± 0.3 | 5.7 ± 0.5 | 4.7 ± 0.6 |
| $K^+$, $g \cdot g\ creat^{-1}$ | 27.7 ± 1.3 | 23.8 ± 0.9* | 27.06 ± 1.1 | 19.7 ± 1.2 | 21.4 ± 1.16 | 19.4 ± 0.6 |
| $Cl^-$, $g \cdot g\ creat^{-1}$ | 19.8 ± 1.2 | 17.5 ± 1.0 | 21.3 ± 1.1 | 14.5 ± 1.1 | 15.9 ± 1.0 | 14.2 ± 0.7 |
| $Ca^{2+}$, $g \cdot g\ creat^{-1}$ | 0.15 ± 0.02 | 0.28 ± 0.04* | 0.24 ± 0.03* | 0.08 ± 0.07 | 0.21 ± 0.02# | 0.20 ± 0.03*** |
| $Mg^{2+}$, $g \cdot g\ creat^{-1}$ | 2.2 ± 0.9 | 2.0 ± 0.1 | 2.3 ± 0.1 | 1.3 ± 0.1 | 1.41 ± 0.13 | 1.65 ± 0.08 |
| Creatinine, $mg \cdot dL^{-1}$ | 43 ± 3 | 34 ± 3 | 29 ± 3** | 50 ± 2 | 25 ± 2# | 15 ± 1# |
| $FE_{UA}$,% | 1.16 ± 0.26 | 1.18 ± 0.31 | 0.72 ± 0.36 | 0.25 ± 0.1 | 0.74 ± 0.4*** | 0.16 ± 0.05** |
| Osmolality, $mOsm \cdot kg\ H_2O^{-1}$ | 2227 ± 186 | 1647 ± 168* | 1440 ± 152** | 2012 ± 132 | 958 ± 58# | 583 ± 20# |
| **Plasma** | n=9 | n=6 | n=13 | n=23 | n=24 | n=14 |
| $Na^+$, $mmol \cdot L^{-1}$ | 149 ± 2 | 149 ± 1 | 149 ± 1 | 147 ± 1 | 149 ± 1* | 153 ± 1*** |
| $Cl^-$, $mmol \cdot L^{-1}$ | 109 ± 1 | 109 ± 1 | 108 ± 1 | 119 ± 1 | 108 ± 1 | 107 ± 1* |
| $Ca^{2+}$, $mmol \cdot L^{-1}$ | 2.72 ± 0.05 | 2.63 ± 0.06 | 2.64 ± 0.06 | 2.50 ± 0.03 | 2.51 ± 0.03 | 2.65 ± 0.04* |
| Creatinine, $mg \cdot dL^{-1}$ | 0.17 ± 0.04 | 0.12 ± 0,01 | 0.15 ± 0.01 | 0.11 ± 0.01 | 0.14 ± 0.01* | 0.18 ± 0.01*** |
| BUN, $mg \cdot dL^{-1}$ | 19 ± 1 | 33 ± 4*** | 43 ± 4# | 21 ± 1 | 55 ± 2# | 70 ± 2# |
| Uric acid, $mg \cdot dL^{-1}$ | 6.6 ± 0.7 | 5.2 ± 1.1 | 5.9 ± 0.5 | 5.4 ± 0.3 | 4.8 ± 0.4 | 5.1 ± 0.8 |
| Osmolality, $mOsm \cdot kg\ H_2O^{-1}$ | 353 ± 4 | 351 ± 4 | 356 ± 3 | 340 ± 2 | 355 ± 2# | 366 ± 3# |

Values are presented as average ± SEM. *$P < 0.05$ , **$P < 0.01$, ***$P < 0.001$ , #$P < 0.0001$ versus age matched *Umod*$^{+/+}$ mice.
n: number of animals, $FE_{UA}$: fractional excretion of uric acid, BUN: blood urea nitrogen.

**Appendix Table S6: Clinical and biochemical parameters of *Umod*$^{R186S/-}$ mice.**

| | 1 month | | | 4 months | | |
|---|---|---|---|---|---|---|
| **Parameter** | *Umod*$^{+/+}$ n=11 | *Umod*$^{R186S/+}$ n=10 | *Umod*$^{R186S/-}$ n=9 | *Umod*$^{+/+}$ n=8 | *Umod*$^{R186S/+}$ n=7 | *Umod*$^{R186S/-}$ n=13 |
| Body weight (BW), g | 16.1 ± 0.7 | 15.6 ± 0.6 | 16.6 ± 0.6 | 29.2 ± 1.7 | 26.6 ± 1.3 | 29.4 ± 0.6 |
| Water intake, $\mu L \cdot min^{-1} \cdot g\ BW^{-1}$ | 0.36 ± 0.03 | 0.30 ± 0.01 | 0.33 ± 0.02 | 0.15 ± 0.02 | 0.29 ± 0.02*** | 0.25 ± 0.02** |
| **Urine** | | | | | | |
| Diuresis, $\mu L \cdot min^{-1} \cdot g\ BW^{-1}$ | 0.048 ± 0.008 | 0.057 ± 0.009 | 0.04 ± 0.01 | 0.03 ± 0.01 | 0.13 ± 0.02*** | 0.11 ± 0.01# |
| $Na^+$, $g \cdot g\ creat^{-1}$ | 8.3 ± 0.5 | 9.0 ± 0.4 | 9.0 ± 0.6 | 3.2 ± 0.5 | 4.0 ± 0.3 | 3.9 ± 0.3 |
| $K^+$, $g \cdot g\ creat^{-1}$ | 27.8 ± 0.9 | 30.0 ± 0.8 | 30.8 ± 1.4 | 18.7 ± 1.2 | 19.1 ± 1.2 | 18.5 ± 0.8 |
| $Cl^-$, $g \cdot g\ creat^{-1}$ | 23.9 ± 0.8 | 25.5 ± 1.0 | 26.3 ± 1.4 | 10.8 ± 0.4 | 12.4 ± 1.0 | 11.6 ± 0.8 |
| $Ca^{2+}$, $g \cdot g\ creat^{-1}$ | 0.23 ± 0.03 | 0.27 ± 0.02 | 0.28 ± 0.03 | 0.14 ± 0.02 | 0.22 ± 0.01** | 0.22 ± 0.01** |
| $Mg^{2+}$, $g \cdot g\ creat^{-1}$ | 2.0 ± 0.2 | 2.2 ± 0.1 | 2.2 ± 0.2 | 1.2 ± 0.1 | 1.43 ± 0.07 | 1.32 ± 0.09 |
| Creatinine, $mg \cdot dL^{-1}$ | 35.2 ± 3.0 | 30.7 ± 1.4 | 37.2 ± 4.1 | 44.2 ± 5.5 | 21.7 ± 2.3** | 20.3 ± 1.1# |
| $FE_{UA}$,% | - | - | - | 0.55 ± 0.09 | 0.14 ± 0.02** | 0.16 ± 0.03** |
| Osmolality, $mOsm \cdot kg\ H_2O^{-1}$ | 1855 ± 120 | 1766 ± 81 | 2091 ± 217 | 1577 ± 201 | 771 ± 42** | 731 ± 29# |
| **Plasma** | n=13 | n=10 | n=11 | n=14 | n=15 | n=20 |
| $Na^+$, $mmol \cdot L^{-1}$ | 145.9 ± 0.8 | 145.3 ± 0.8 | 145.1 ± 0.7 | 147.3 ± 0.7 | 149.1 ± 0.5* | 148.2 ± 0.5 |
| $Cl^-$, $mmol \cdot L^{-1}$ | 107.8 ± 0,7 | 107.1 ± 0.5 | 110.3 ± 0.9 | 110.0 ± 1.1 | 107.1 ± 0.7* | 106 .6 ± 0.3** |
| $Ca^{2+}$, $mmol \cdot L^{-1}$ | 2.67 ± 0.07 | 2.82 ± 0.05 | 2.77 ± 0.05 | 2.46 ± 0.05 | 2.62 ± 0.03** | 2.62 ± 0.03** |
| Creatinine, $mg \cdot dL^{-1}$ | 0.15 ± 0.02 | 0.142 ± 0.02 | 0.17 ± 0.03 | 0.10 ± 0.02 | 0.17 ± 0.02* | 0.16 ± 0.01* |
| BUN, $mg \cdot dL^{-1}$ | 19 ± 1 | 32 ± 2# | 32 ± 1.# | 20 ± 1.0 | 53 ± 4 # | 56 ± 1.# |
| Uric acid, $mg \cdot dL^{-1}$ | 3.3 ± 0.5 | 4.0 ± 0.9 | 4.8 ± 0.5 | 4.1 ± 0.3 | 4.1 ± 0.4 | 3.8 ± 0.4 |
| Osmolality, $mOsm \cdot kg\ H_2O^{-1}$ | 341 ± 4 | 353 ± 3 | 355 ± 4* | 338 ± 3 | 353 ± 3** | 357 ± 3# |

Values are presented as average ± SEM. *$P < 0.05$, **$P < 0.01$, ***$P < 0.001$, #$P < 0.0001$ versus age-matched *Umod*$^{+/+}$ mice or Ϯ $P \leq 0.05$ versus age-matched *Umod*$^{R186S/+}$ mice. n: number of animals, $FE_{UA}$: fractional excretion of uric acid, BUN: blood urea nitrogen.

**Appendix Table S7: Clinical and biological parameters of 4-month *Umod* KI mice per gender.**

| | Female | | | Male | | |
|---|---|---|---|---|---|---|
| **Plasma** | *Umod*$^{+/+}$ n=10 | *Umod*$^{C171Y/+}$ n=12 | *Umod*$^{C171Y/C171Y}$ n=17 | *Umod*$^{+/+}$ n=5 | *Umod*$^{C171Y/+}$ n=8 | *Umod*$^{C171Y/C171Y}$ n=7 |
| BUN, mg·dL$^{-1}$ | 18 ± 2 | 23 ± 1 | 27 ± 2** | 19 ± 2 | 28 ± 2* | 36 ± 2*** |

| **Urine** | *Umod*$^{+/+}$ n=5 | *Umod*$^{R186S/+}$ n=9 | *Umod*$^{R186S/R186S}$ n=3 | *Umod*$^{+/+}$ n=5 | *Umod*$^{R186S/+}$ n=4 | *Umod*$^{R186S/R186S}$ n=4 |
|---|---|---|---|---|---|---|
| Diuresis, µL·min$^{-1}$·g BW$^{-1}$ | 0.05 ± 0.01 | 0.11 ± 0.02[a] | 0.3 ± 0.00[a] | 0.04 ± 0.01 | 0.14 ± 0.01** | 0.21 ± 0.02# |
| Osmolality, mOsm·kg $H_2O^{-1}$ | 2087 ± 246 | 950 ± 87*** | 564 ± 50*** | 1936 ± 157 | 974 ± 47*** | 598 ± 17# |
| **Plasma** | n=17 | n=14 | n=7 | n=7 | n=11 | n=7 |
| Creatinine, mg·dL$^{-1}$ | 0.10 ± 0.01[b] | 0.18 ± 0.01[c]# | 0.21 ± 0.02# | 0.14 ± 0.02 | 0.10 ± 0.02[c] | 0.156 ± 0.004 |
| BUN, mg·dL$^{-1}$ | 19 ± 1 | 53 ± 3# | 71 ± 3# | 25 ± 1 | 58 ± 3# | 70 ± 3# |

| **Urine** | *Umod*$^{+/+}$ n=5 | *Umod*$^{R186S/+}$ n=9 | *Umod*$^{R186S/R186S}$ n=3 | *Umod*$^{+/+}$ n=5 | *Umod*$^{R186S/+}$ n=4 | *Umod*$^{R186S/R186S}$ n=4 |
|---|---|---|---|---|---|---|
| Diuresis, µL·min$^{-1}$·g BW$^{-1}$ | 0.008 ± 0.001 | 0.13 ± 0.06 | 0.14 ± 0.03 | 0.04 ± 0.02 | 0.13 ± 0.02** | 0.10 ± 0.01** |
| Osmolality, mOsm·kg $H_2O^{-1}$ | 2046 ± 438 | 752 ± 64 | 701 ± 89 | 1296 ± 140 | 778 ± 62*** | 736 ± 31# |
| **Plasma** | n=6 | n=6 | n=10 | n=8 | n=9 | n=10 |
| Creatinine, mg·dL$^{-1}$ | 0.10 ± 0.03[d] | 0.17 ± 0.02 | 0.17 ± 0.02 | 0.10 ± 0.03[d] | 0.17 ± 0.03[d] | 0.15 ± 0.02 |
| BUN, mg·dL$^{-1}$ | 18 ± 1 | 54 ± 4# | 56 ± 4# | 22 ± 1 | 52 ± 6# | 56 ± 2# |

Values are presented as average ± SEM. One-way ANOVA with Tukey's post hoc per gender. *$P$<0.05, **$P$<0.01, ***$P$<0.001, #P<0.0001 versus gender-matched *Umod*$^{+/+}$ mice, n: number of animals, BUN: blood urea nitrogen. [a]2 samples were not available for analysis, [b]3 samples were undetectable. [c]1 sample was undetectable, [d]2 samples were undetectable.

**Appendix Table S8: Top 50 DEGs in *Umod*$^{R186S/+}$ kidneys at 1 month.**

| Symbol | Gene name | Fold change (log2) | FDR (-log10) |
|---|---|---|---|
| *Lcn2* | Lipocalin 2 | 4.09 | 100.44 |
| *Atf5* | Activating transcription factor 5 | 2.61 | 52.02 |
| *Trib3* | Tribbles pseudokinase 3 | 4.04 | 37.43 |
| *Asns* | Asparagine synthetase | 2.39 | 33.55 |
| *Mthfd2* | Methylenetetrahydrofolate dehydrogenase (NAD+ dependent), methenyltetrahydrofolate cyclohydrolase | 2.31 | 27.30 |
| *Akr1b8* | Aldo-keto reductase family 1, member B8 | 2.21 | 20.17 |
| *Cxcl10* | Chemokine (C-X-C motif) ligand 10 | 2.92 | 19.70 |
| *Stc2* | Stanniocalcin 2 | 2.29 | 19.52 |
| *Aldh18a1* | Aldehyde dehydrogenase 18 family, member A1 | 2.08 | 18.88 |
| *Angptl6* | Angiopoietin-like 6 | 2.33 | 14.69 |
| *Aldh1l2* | Aldehyde dehydrogenase 1 family, member L2 | 1.87 | 14.36 |
| *Slc7a11* | Solute carrier family 7 (cationic amino acid transporter, y+ system), member 11 | 2.56 | 13.53 |
| *Mt2* | Metallothionein 2 | 1.76 | 13.17 |
| *Slc7a3* | Solute carrier family 7 (cationic amino acid transporter, y+ system), member 3 | 2.62 | 12.81 |
| *Nupr1* | Nuclear protein transcription regulator 1 | 1.45 | 12.61 |
| *Cbr3* | Carbonyl reductase 3 | 2.15 | 12.12 |
| *Arhgap36* | Rho GTPase activating protein 36 | 2.76 | 11.99 |
| *Soat2* | Sterol O-acyltransferase 2 | 2.56 | 11.48 |
| *Pappa* | Pregnancy-associated plasma protein A | 1.91 | 11.33 |
| *Slc38a1* | Solute carrier family 38, member 1 | 1.40 | 11.25 |
| *Wnt10a* | Wingless-type MMTV integration site family, member 10A | 2.65 | 10.75 |
| *Gabrp* | Gamma-aminobutyric acid (GABA) A receptor, pi | 2.51 | 10.74 |
| *Loxl4* | Lysyl oxidase-like 4 | 1.66 | 9.76 |
| *Lgals3* | Lectin, galactose binding, soluble 3 | 1.24 | 9.48 |
| *Fam129a* | Family with sequence similarity 129, member A | 1.32 | 8.13 |
| *Egf* | Epidermal growth factor | -2.00 | 40.86 |
| *Umod* | Uromodulin | -1.74 | 24.75 |
| *Car3* | Carbonic anhydrase 3 | -2.01 | 14.36 |
| *Gm36797* | Predicted gene, 36797 | -2.65 | 13.46 |
| *Gm32960* | Predicted gene, 32960 | -1.74 | 13.17 |
| *Kcnt1* | Potassium channel, subfamily T, member 1 | -1.87 | 9.55 |
| *Wfdc15b* | WAP four-disulfide core domain 15B | -1.23 | 7.59 |
| *Azgp1* | Aalpha-2-glycoprotein 1, zinc | -1.47 | 6.76 |
| *Dusp15* | Dual specificity phosphatase-like 15 | -1.46 | 6.47 |
| *Cyp2a4* | Cytochrome P450, family 2, subfamily a, polypeptide 4 | -1.04 | 6.32 |
| *Slc6a6* | Solute carrier family 6 (neurotransmitter transporter, taurine), member 6 | -0.63 | 5.88 |
| *Atp8b4* | ATPase, class I, type 8B, member 4 | -1.56 | 5.67 |
| *Fam107a* | Family with sequence similarity 107, member A | -0.99 | 5.53 |
| *Slco1a6* | Solute carrier organic anion transporter family, member 1a6 | -0.74 | 4.95 |
| *Tmem207* | Transmembrane protein 207 | -1.39 | 4.87 |
| *BC040756* | cDNA sequence BC040756 | -0.87 | 4.74 |
| *Pex5l* | Peroxisomal biogenesis factor 5-like | -1.48 | 4.49 |
| *Esrrb* | Estrogen related receptor, beta | -0.97 | 4.49 |
| *Cwh43* | Cell wall biogenesis 43 C-terminal homolog | -0.78 | 4.49 |
| *6330410L21Rik* | RIKEN cDNA 6330410L21 gene | -1.35 | 4.41 |
| *Ugt8a* | UDP galactosyltransferase 8A | -0.85 | 4.41 |
| *Omd* | Osteomodulin | -1.12 | 4.19 |
| *Rnf169* | Ring finger protein 169 | -0.88 | 4.18 |
| *Dok2* | Docking protein 2 | -1.13 | 4.13 |
| *Perm1* | PPARGC1 and ESRR induced regulator, muscle 1 | -1.36 | 4.09 |

FDR: False discovery rate-adjusted *P* value (Benjamini-Hochberg correction).

**Appendix Table S9: Top 50 DEGs in *Umod*$^{R186S/+}$ kidneys at 4 months.**

| Symbol | Gene name | Fold change (log2) | FDR (-log10) |
|---|---|---|---|
| *Lcn2* | Lipocalin 2 | 4.23 | 114.29 |
| *Arhgap36* | Rho GTPase activating protein 36 | 6.04 | 66.64 |
| *Slc7a11* | Solute carrier family 7 (cationic amino acid transporter, y+ system), member 11 | 4.91 | 53.68 |
| *Akr1b8* | Aldo-keto reductase family 1, member B8 | 3.36 | 52.89 |
| *Nefl* | Neurofilament, light polypeptide | 3.98 | 52.36 |
| *Dpt* | Dermatopontin | 3.03 | 49.97 |
| *Cbr3* | Carbonyl reductase 3 | 4.02 | 47.22 |
| *Atf5* | Activating transcription factor 5 | 2.38 | 44.14 |
| *Ppp2r2c* | Protein phosphatase 2, regulatory subunit B, gamma | 4.49 | 37.30 |
| *Trib3* | Tribbles pseudokinase 3 | 3.78 | 34.16 |
| *B4galnt2* | Beta-1,4-N-acetyl-galactosaminyl transferase 2 | 3.78 | 32.79 |
| *Aldh18a1* | Aldehyde dehydrogenase 18 family, member A1 | 2.62 | 30.76 |
| *Asns* | Asparagine synthetase | 2.14 | 28.48 |
| *Stc2* | Stanniocalcin 2 | 2.84 | 26.86 |
| *Gabrp* | Gamma-aminobutyric acid (GABA) A receptor, pi | 3.51 | 23.20 |
| *Aldh1l2* | Aldehyde dehydrogenase 1 family, member L2 | 2.25 | 22.19 |
| *Abcc3* | ATP-binding cassette, sub-family C (CFTR/MRP), member 3 | 2.21 | 21.80 |
| *Smoc2* | SPARC related modular calcium binding 2 | 2.04 | 21.77 |
| *Astn2* | Astrotactin 2 | 2.22 | 21.40 |
| *Fcrls* | Fc receptor-like S, scavenger receptor | 3.50 | 20.52 |
| *Kif1a* | Kinesin family member 1A | 2.90 | 20.50 |
| *Lyz2* | Lysozyme 2 | 1.98 | 20.30 |
| *Gm32857* | Predicted gene, 32857 | 3.62 | 20.28 |
| *Mrc1* | Mannose receptor, C type 1 | 1.89 | 20.28 |
| *Mthfd2* | Methylenetetrahydrofolate dehydrogenase (NAD+ dependent), methenyltetrahydrofolate cyclohydrolase | 1.92 | 19.46 |
| *Egf* | Epidermal growth factor | -3.30 | 115.22 |
| *Gpx6* | Glutathione peroxidase 6 | -2.57 | 58.29 |
| *Kcnt1* | Potassium channel, subfamily T, member 1 | -4.04 | 45.73 |
| *Umod* | Uromodulin | -2.13 | 38.09 |
| *Wfdc15b* | WAP four-disulfide core domain 15B | -2.21 | 28.02 |
| *Tmem207* | Transmembrane protein 207 | -2.62 | 20.68 |
| *Aldoc* | Aldolase C, fructose-bisphosphate | -1.67 | 17.42 |
| *Dusp15* | Dual specificity phosphatase-like 15 | -2.27 | 15.75 |
| *Gm36797* | Predicted gene, 36797 | -2.76 | 14.99 |
| *Gm32960* | Predicted gene, 32960 | -1.76 | 14.00 |
| *Ppp1r1a* | Protein phosphatase 1, regulatory inhibitor subunit 1A | -1.13 | 13.72 |
| *Pex5l* | Peroxisomal biogenesis factor 5-like | -2.36 | 12.85 |
| *Perm1* | PPARGC1 and ESRR induced regulator, muscle 1 | -2.21 | 12.77 |
| *Lrrc66* | Leucine rich repeat containing 66 | -1.65 | 12.49 |
| *Ckb* | Creatine kinase, brain | -1.36 | 11.45 |
| *Ank2* | Ankyrin 2, brain | -1.18 | 10.87 |
| *Mfsd4a* | Major facilitator superfamily domain containing 4A | -1.10 | 10.75 |
| *6330410L21Rik* | RIKEN cDNA 6330410L21 gene | -2.09 | 10.48 |
| *Pcsk6* | Proprotein convertase subtilisin/kexin type 6 | -1.27 | 10.45 |
| *Slc6a12* | Solute carrier family 6 (neurotransmitter transporter, betaine/GABA), member 12 | -1.90 | 9.48 |
| *Spag5* | Sperm associated antigen 5 | -1.78 | 9.48 |
| *Clcnka* | Chloride channel, voltage-sensitive Ka | -1.29 | 9.46 |
| *Gcgr* | Glucagon receptor | -1.19 | 9.23 |
| *Pla1a* | Phospholipase A1 member A | -1.00 | 8.76 |
| *Aqp4* | Aquaporin 4 | -1.37 | 8.33 |

FDR: False discovery rate-adjusted *P* value (Benjamini-Hochberg correction).

## Appendix Table S10: Top 50 DEGs in *Umod*$^{C171Y/+}$ kidneys at 4 months.

| Symbol | Gene name | Fold change (log2) | FDR (-log10) |
|---|---|---|---|
| *Jun* | Jun proto-oncogene | 2.86 | 23.00 |
| *Btg2* | BTG anti-proliferation factor 2 | 2.72 | 22.27 |
| *Ccn1* | Cellular communication network factor 1 | 3.29 | 18.39 |
| *Fos* | FBJ osteosarcoma oncogene | 3.38 | 16.46 |
| *Ier2* | Immediate early response 2 | 2.68 | 15.92 |
| *Zfp36* | Zinc finger protein 36 | 1.96 | 13.97 |
| *Nr4a2* | Nuclear receptor subfamily 4, group A, member 2 | 2.94 | 13.51 |
| *Ier3* | Immediate early response 3 | 2.15 | 13.47 |
| *Nr4a1* | Nuclear receptor subfamily 4, group A, member 1 | 2.79 | 10.13 |
| *Fosb* | FBJ osteosarcoma oncogene B | 2.03 | 6.90 |
| *Snord14e* | Small nucleolar RNA, C/D box 14° | 1.77 | 6.43 |
| *Csrnp1* | Cysteine-serine-rich nuclear protein 1 | 1.65 | 6.32 |
| *Gdf15* | Growth differentiation factor 15 | 1.87 | 6.32 |
| *Ccn2* | Cellular communication network factor 2 | 1.18 | 6.18 |
| *Gm30591* | Predicted gene, 30591 | 2.09 | 5.88 |
| *Tob1* | Transducer of ErbB-2.1 | 1.14 | 5.47 |
| *Egr1* | Early growth response 1 | 1.94 | 4.77 |
| *Gm17971* | Predicted gene, 17971 | 1.28 | 4.69 |
| *Snord14d* | Small nucleolar RNA, C/D box 14D | 1.77 | 4.11 |
| *Egr3* | Early growth response 3 | 1.76 | 3.99 |
| *Rasd1* | RAS, dexamethasone-induced 1 | 1.29 | 3.68 |
| *Dusp6* | Dual specificity phosphatase 6 | 1.17 | 3.67 |
| *Ighv5-9* | Immunoglobulin heavy variable 5-9 | 1.47 | 3.31 |
| *Ch25h* | Cholesterol 25-hydroxylase | 1.68 | 3.27 |
| *Ighg2b* | Immunoglobulin heavy constant gamma 2B | 1.70 | 2.93 |
| *mt-Tl1* | tRNA leucine 1, mitochondrial | -1.21 | 4.57 |
| *Gm30238* | predicted gene, 30238 | -1.60 | 3.27 |
| *Igkv13-85* | immunoglobulin kappa chain variable 13-85 | -1.12 | 2.21 |
| *Cyp2d26* | cytochrome P450, family 2, subfamily d, polypeptide 26 | -1.32 | 2.16 |
| *Lpl* | lipoprotein lipase | -1.38 | 2.14 |
| *Synpr* | synaptoporin | -1.30 | 2.06 |
| *Mir6236* | microRNA 6236 | -1.51 | 2.05 |
| *1700067K01Rik* | RIKEN cDNA 1700067K01 gene | -1.01 | 1.91 |
| *Atp1a2* | ATPase, Na+/K+ transporting, alpha 2 polypeptide | -1.20 | 1.82 |
| *Itih1* | inter-alpha trypsin inhibitor, heavy chain 1 | -1.09 | 1.76 |
| *Apoc1* | apolipoprotein C-I | -1.04 | 1.55 |
| *Adig* | adipogenin | -0.91 | 5.38 |
| *Slc5a1* | solute carrier family 5 (sodium/glucose cotransporter), member 1 | -0.64 | 3.17 |
| *Rn18s-rs5* | 18s RNA, related sequence 5 | -0.94 | 2.83 |
| *LOC108167485* | putative uncharacterized protein FLJ37770 pseudogene | -0.87 | 2.11 |
| *Gm17597* | predicted gene, 17597 | -0.83 | 2.11 |
| *Mri1* | methylthioribose-1-phosphate isomerase 1 | -0.51 | 1.92 |
| *Gm34472* | predicted gene, 34472 | -0.91 | 1.73 |
| *Gm28023* | predicted gene, 28023 | -0.65 | 1.56 |
| *Tcf24* | transcription factor 24 | -0.58 | 1.37 |
| *Ugt3a1* | UDP glycosyltransferases 3 family, polypeptide A1 | -0.39 | 1.29 |
| *Cfd* | complement factor D (adipsin) | -1.26 | 1.25 |
| *AI429214* | expressed sequence AI429214 | -0.75 | 1.25 |
| *AA536875* | expressed sequence AA536875 | -0.92 | 1.15 |
| *Gm15564* | predicted gene 15564 | -1.22 | 1.15 |

FDR: False discovery rate-adjusted *P* value (Benjamini-Hochberg correction).

**Appendix Table S11: Top 50 DEGs in *Umod*$^{R186S/+}$ compared to *Umod*$^{C171Y/+}$ kidneys at 1 month.**

| Symbol | Gene name | Fold change (log2) | FDR (-log10) |
|---|---|---|---|
| *Lcn2* | Lipocalin 2 | 4.16 | 99.72 |
| *Atf5* | Activating transcription factor 5 | 2.67 | 53.11 |
| *Asns* | Asparagine synthetase | 2.36 | 32.01 |
| *Trib3* | Tribbles pseudokinase 3 | 3.65 | 30.80 |
| *Mthfd2* | Methylenetetrahydrofolate dehydrogenase (NAD+ dependent), methenyltetrahydrofolate cyclohydrolase | 2.28 | 26.20 |
| *Stc2* | Stanniocalcin 2 | 2.45 | 21.46 |
| *Akr1b8* | Aldo-keto reductase family 1, member B8 | 2.29 | 21.33 |
| *Cxcl10* | Chemokine (C-X-C motif) ligand 10 | 2.94 | 19.82 |
| *Aldh18a1* | Aldehyde dehydrogenase 18 family, member A1 | 2.16 | 19.82 |
| *Cbr3* | Carbonyl reductase 3 | 2.45 | 14.95 |
| *Arhgap36* | Rho GTPase activating protein 36 | 2.99 | 13.98 |
| *Nupr1* | Nuclear protein transcription regulator 1 | 1.44 | 12.23 |
| *Angptl6* | Angiopoietin-like 6 | 2.13 | 12.16 |
| *Samd5* | Sterile alpha motif domain containing 5 | 2.49 | 11.82 |
| *Soat2* | Sterol O-acyltransferase 2 | 2.62 | 11.70 |
| *Aldh1l2* | Aldehyde dehydrogenase 1 family, member L2 | 1.71 | 11.70 |
| *Gabrp* | Gamma-aminobutyric acid (GABA) A receptor, pi | 2.62 | 11.40 |
| *Slc7a11* | Solute carrier family 7 (cationic amino acid transporter, y+ system), member 11 | 2.36 | 11.40 |
| *Lgals3* | Lectin, galactose binding, soluble 3 | 1.34 | 11.16 |
| *Slc7a3* | Solute carrier family 7 (cationic amino acid transporter, y+ system), member 3 | 2.44 | 10.98 |
| *Wnt10a* | Wingless-type MMTV integration site family, member 10A | 2.58 | 10.07 |
| *Mt2* | Metallothionein 2 | 1.55 | 10.03 |
| *Pappa* | Pregnancy-associated plasma protein A | 1.81 | 9.98 |
| *Slc38a1* | Solute carrier family 38, member 1 | 1.31 | 9.58 |
| *Serpina10* | Serine (or cysteine) peptidase inhibitor, clade A (alpha-1 antiproteinase, antitrypsin), member 10 | 2.02 | 9.52 |
| *Egf* | Epidermal growth factor | -1.89 | 36.25 |
| *Umod* | Uromodulin | -1.63 | 21.44 |
| *Azgp1* | Alpha-2-glycoprotein 1, zinc | -1.83 | 11.19 |
| *Car3* | Carbonic anhydrase 3 | -1.80 | 11.16 |
| *Kcnt1* | Potassium channel, subfamily T, member 1 | -1.93 | 10.25 |
| *Gm36797* | Predicted gene, 36797 | -2.32 | 9.74 |
| *Gm34472* | Predicted gene, 34472 | -1.21 | 8.77 |
| *Gm34861* | Predicted gene, 34861 | -1.54 | 8.63 |
| *Cyp2a4* | Cytochrome P450, family 2, subfamily a, polypeptide 4 | -1.14 | 7.88 |
| *Tc2n* | Tandem C2 domains, nuclear | -1.37 | 7.77 |
| *Agps* | Alkylglycerone phosphate synthase | -1.01 | 7.08 |
| *Ksr2* | Kinase suppressor of ras 2 | -1.10 | 7.05 |
| *Wfdc15b* | WAP four-disulfide core domain 15B | -1.18 | 7.01 |
| *Gm32960* | Predicted gene, 32960 | -1.30 | 6.72 |
| *Trpm6* | Transient receptor potential cation channel, subfamily M, member 6 | -1.26 | 6.64 |
| *Atp8b4* | ATPase, class I, type 8B, member 4 | -1.58 | 5.95 |
| *Syn3* | Synapsin III | -1.33 | 5.87 |
| *Col19a1* | Collagen, type XIX, alpha 1 | -1.60 | 5.79 |
| *Idi1* | Isopentenyl-diphosphate delta isomerase | -1.21 | 5.77 |
| *Slco1a1* | Solute carrier organic anion transporter family, member 1a1 | -1.63 | 5.67 |
| *Ubiad1* | UbiA prenyltransferase domain containing 1 | -1.26 | 5.31 |
| *Dusp15* | Dual specificity phosphatase-like 15 | -1.33 | 5.23 |
| *Gm38481* | Predicted gene, 38481 | -1.22 | 4.87 |
| *Gm7537* | Predicted gene 7537 | -1.27 | 4.81 |
| *Perm1* | PPARGC1 and ESRR induced regulator, muscle 1 | -1.43 | 4.80 |

FDR: False discovery rate-adjusted *P* value (Benjamini-Hochberg correction).

**Appendix Table S12: Top 50 DEGs in *Umod*$^{R186S/+}$ compared to *Umod*$^{C171Y/+}$ at 4 months.**

| Symbol | Gene name | Fold change (log2) | FDR (-log10) |
|---|---|---|---|
| *Lcn2* | Lipocalin 2 | 4.73 | 132.87 |
| *Akr1b8* | Aldo-keto reductase family 1, member B8 | 3.95 | 70.59 |
| *Arhgap36* | Rho GTPase activating protein 36 | 5.99 | 65.97 |
| *Slc7a11* | Solute carrier family 7 (cationic amino acid transporter, y+ system), member 11 | 4.76 | 51.56 |
| *Dpt* | Dermatopontin | 2.99 | 49.40 |
| *Nefl* | Neurofilament, light polypeptide | 3.41 | 42.31 |
| *Trib3* | Tribbles pseudokinase 3 | 4.00 | 37.82 |
| *Cbr3* | Carbonyl reductase 3 | 3.34 | 35.67 |
| *Atf5* | Activating transcription factor 5 | 2.10 | 35.22 |
| *Ppp2r2c* | Protein phosphatase 2, regulatory subunit B, gamma | 4.32 | 35.17 |
| *B4galnt2* | Beta-1,4-N-acetyl-galactosaminyl transferase 2 | 3.62 | 30.75 |
| *Asns* | Asparagine synthetase | 2.21 | 30.44 |
| *Gabrp* | Gamma-aminobutyric acid (GABA) A receptor, pi | 4.00 | 29.49 |
| *Aldh18a1* | Aldehyde dehydrogenase 18 family, member A1 | 2.32 | 25.09 |
| *Gsta4* | Glutathione S-transferase, alpha 4 | 1.57 | 22.20 |
| *Stc2* | Stanniocalcin 2 | 2.40 | 20.49 |
| *Kif1a* | Kinesin family member 1A | 2.88 | 20.41 |
| *Gm32857* | Predicted gene, 32857 | 3.64 | 20.41 |
| *Col18a1* | Collagen, type XVIII, alpha 1 | 1.05 | 20.29 |
| *Abcc3* | ATP-binding cassette, sub-family C (CFTR/MRP), member 3 | 2.05 | 19.39 |
| *Astn2* | Astrotactin 2 | 2.08 | 19.23 |
| *Slc38a1* | Solute carrier family 38, member 1 | 1.78 | 19.08 |
| *Mthfd2* | Methylenetetrahydrofolate dehydrogenase (NAD+ dependent), methenyltetrahydrofolate cyclohydrolase | 1.89 | 19.08 |
| *Eda2r* | Ectodysplasin A2 receptor | 2.53 | 18.69 |
| *Ugt2b35* | UDP glucuronosyltransferase 2 family, polypeptide B35 | 3.39 | 18.43 |
| *Egf* | Epidermal growth factor | -3.06 | 99.15 |
| *Gpx6* | Glutathione peroxidase 6 | -2.50 | 55.12 |
| *Kcnt1* | Potassium channel, subfamily T, member 1 | -3.94 | 43.24 |
| *Umod* | Uromodulin | -2.09 | 36.46 |
| *Jun* | Jun proto-oncogene | -3.19 | 30.59 |
| *Zfp36* | Zinc finger protein 36 | -2.62 | 27.09 |
| *Btg2* | BTG anti-proliferation factor 2 | -2.90 | 26.64 |
| *Tmem207* | Transmembrane protein 207 | -2.71 | 22.26 |
| *Ckb* | Creatine kinase, brain | -1.84 | 22.20 |
| *Ier2* | Immediate early response 2 | -2.95 | 20.44 |
| *Wfdc15b* | WAP four-disulfide core domain 15B | -1.90 | 20.41 |
| *Nr4a1* | Nuclear receptor subfamily 4, group A, member 1 | -3.63 | 19.45 |
| *Pex5l* | Peroxisomal biogenesis factor 5-like | -2.83 | 19.25 |
| *Fos* | FBJ osteosarcoma oncogene | -3.41 | 17.78 |
| *Ccn1* | Cellular communication network factor 1 | -3.15 | 17.74 |
| *Ppp1r1a* | Protein phosphatase 1, regulatory inhibitor subunit 1A | -1.27 | 17.43 |
| *Snord14e* | Small nucleolar RNA, C/D box 14E | -2.76 | 17.17 |
| *Dusp15* | Dual specificity phosphatase-like 15 | -2.35 | 17.14 |
| *Csrnp1* | Cysteine-serine-rich nuclear protein 1 | -2.46 | 17.07 |
| *Ier3* | Immediate early response 3 | -2.28 | 16.10 |
| *Gm36797* | Predicted gene, 36797 | -2.79 | 15.52 |
| *Nr4a2* | Nuclear receptor subfamily 4, group A, member 2 | -2.96 | 14.57 |
| *Tob1* | Transducer of ErbB-2.1 | -1.65 | 14.36 |
| *Ccn2* | Cellular communication network factor 2 | -1.62 | 14.05 |
| *Gm17971* | Predicted gene, 17971 | -2.05 | 14.02 |

FDR: False discovery rate-adjusted *P* value (Benjamini-Hochberg correction).

**Appendix Table S13: List of primary antibodies.**

| Target Antigen | Host species | Dilution | Cat # | Registry ID | Source |
|---|---|---|---|---|---|
| Uromodulin | Sheep | IF 1:300 | K90071C | AB_153128 | Meridian Life Science |
| | | WB 1:500 | | | |
| GRP78 | Rabbit | IF 1:300 | ab21685 | AB_2119834 | Abcam |
| | | WB 1:1000 | | | |
| β-Actin | Mouse | WB 1:10000 | A5441 | AB_476744 | Sigma-Aldrich |
| Calnexin | Rabbit | IF 1:300 | C4731 | AB_476845 | Sigma-Aldrich |
| CD3 | Rabbit | IF 1:300 | ab16669 | AB_443425 | Abcam |
| PERK | Rabbit | WB 1:500 | 3192 | AB_2095847 | Cell Signaling Technology |
| IRE1 (phospho Ser724) | Rabbit | WB 1:500 | ab48187 | AB_873899 | Abcam |
| IRE1-α | Rabbit | WB 1:500 | 3294 | AB_823545 | Cell Signaling Technology |
| ATF4 | Rabbit | WB 1:500 | ab105383 | AB_10861973 | Abcam |
| eIF2α (phospho Ser51) | Rabbit | WB 1:250 | 3398 | AB_2096481 | Cell Signaling Technology |
| eIF2α | Rabbit | WB 1:500 | 5324 | AB_10692650 | Cell Signaling Technology |
| Lipocalin 2 | Rabbit | IF 1:300 | ab63929 | AB_1140965 | Abcam |
| | Goat | WB 1:500 | AF1857 | AB_355022 | R and D Systems |
| Cleaved Caspase-3 | Rabbit | WB 1:250 | 9661 | AB_2341188 | Cell Signaling Technology |
| Caspase-3 | Rabbit | WB 1:1000 | 9662 | AB_331439 | Cell Signaling Technology |
| SQSTM1/p62 | Mouse | WB 1:1000 | ab56416 | AB_945626 | Abcam |
| | Rabbit | IF 1:200 | ab109012 | AB_2810880 | Abcam |
| S6 Ribosomal Protein (phospho S235/236) | Rabbit | WB 1:500 | 4858 | AB_916156 | Cell Signaling Technology |
| S6 Ribosomal Protein | Rabbit | WB 1:500 | 2217 | AB_331355 | Cell Signaling Technology |
| LC3 | Rabbit | IF 1:300 | PM036 | AB_2274121 | MBL International |
| | | WB 1:500 | | | |
| GFP | Goat | WB 1:1000 | AB0020 | AB_2333100 | SICGEN |
| Ubiquitin | Mouse | WB 1:1000 | sc-8017 | AB_628423 | Santa Cruz Biotechnology |
| F4/80 | Rabbit | IF 1:300 | 70076 | AB_2799771 | Cell Signaling Technology |
| LAMP1 | Rat | IF 1:300 | sc-19992 | AB_2134495 | Santa Cruz Biotchnology |
| ATG5 | Rabbit | IF 1:300 | ab108327 | AB_2650499 | Abcam |

## Appendix Table S14: Primers used for real-time RT-PCR analysis.

| Gene product | Forward primer (5'-3') | Reverse primer (5'-3') | PCR Product (bp) | Efficiency |
|---|---|---|---|---|
| *18S* | GTA ACC CGT TGA ACC CCA TT | CCA TCC AAT CGG TAG TAG CG | 151 | 0.98 ± 0.02 |
| *36B4* | CTT CAT TGT GGG AGC AGA CA | TTC TCC AGA GCT GGG TTG TT | 150 | 1.02 ± 0.02 |
| *Acox1* | CTG GTG GGT GGT ATG GTG TC | GTG ACT CAC TTG GGC CTG AA | 186 | 1.03 ± 0.03 |
| *Acox2* | AAG CCT CAT CCA ACG TGA CC | AAT GCG TTC AGG ACC GTC TT | 151 | 0.99 ± 0.02 |
| *Acox3* | CAT GTA CGA CTG GTC CCT GG | CCC ATG ACT CAG TTC GGT GA | 160 | 1.02 ± 0.03 |
| *Acta2* | TGT GCT GGA CTC TGG AGA TG | GAA GGA ATA GCC ACG CTC AG | 148 | 1.03 ± 0.02 |
| *Actg1* | TGC CCA TCT ATG AGG GCT AC | CCC GTT CAG TCA GGA TCT TC | 102 | 1.03 ± 0.04 |
| *Adgre1* | CCA GGA GTG GCT TTT GTC TC | GGC TTG GAG AAG TCC TCC TT | 152 | 0.97 ± 0.03 |
| *Atf3* | CCA GGT CTC TGC CTC AGA AG | CCG ATG GCA GAG GTG TTT AT | 151 | 0.98 ± 0.03 |
| *Atf4* | CAT GCC AGA TGA GCT CTT GA | GGC AAC CTG GTC GAC TTT TA | 145 | 0.96 ± 0.03 |
| *Ccnd1* | AGC AGA AGT GCG AAG AGG AG | CAA GGG AAT GGT CTC CTT CA | 149 | 0.98 ± 0.03 |
| *Cd68* | CCA ACA AAA CCA AGG TCC AG | ATT GTA TTC CAC CGC CAT GT | 152 | 1.03 ± 0.03 |
| *Col1a1* | GAT CTC CTG GTG CTG ATG GA | GAC CTT GTT TGC CAG GTT CA | 156 | 0.98 ± 0.03 |
| *Col3a1* | TCC TGG TGG TCC TGG TAC T | TTG CCA GGA GAA CCA CTG TT | 154 | 0.96 ± 0.04 |
| *Cpt1a* | TGG CAG TCG ACT CAC CTT TC | ACA CCA TAG CCG TCA TCA GC | 166 | 0.98 ± 0.02 |
| *Cpt2* | TTG ACG CCA TTC AGT TTC AG | GCA GTG CTG CAG GAT TCA TA | 148 | 1.02 ± 0.03 |
| *Cryab* | ACT TCC CTG AGC CCC TTC TA | CTT GCC GTG GAC CTC AAT CA | 186 | 0.98 ± 0.02 |
| *Dnaja4* | TGA AGG CAT CGG TGG GAA AA | AGT TCT CAC AGC GGT CCT TG | 176 | 1.02 ± 0.03 |
| *Dnajb4* | GAC CCT CCC GTC TCA AAC AA | TGA TTT TGG TGC CTT CTT TCC AC | 195 | 1.01 ± 0.02 |
| *Ddit3* | CCA GGA GGA AGA GGA GGA AG | CCG CTC GTT CTC TTC AGC TA | 148 | 1.02 ± 0.03 |
| *Eif2a* | ATT ATC ACC ATC CCC GCC AC | GCA GGC ACA GAC AGT CTC AT | 166 | 0.97 ± 0.04 |
| *Eif2ak3* | GAC TGC GGA GAC AAC AGT GA | GGA CGT TCC TTC CCT AGA CC | 173 | 0.99 ± 0.02 |
| *Fn1* | GCA AGC CAG TTT CCA TCA AT | CAT TTT TGG GAG TGG TGG TC | 150 | 0.98 ± 0.02 |
| *Gapdh* | TGC ACC ACC AAC TGC TTA GC | GGA TGC AGG GAT GGG GGA GA | 176 | 1.04 ± 0.03 |
| *Hprt1* | ACA TTG TGG CCC TCT GTG TG | TTA TGT CCC CCG TTG ACT GA | 162 | 0.99 ± 0.01 |
| *Hsp90aa1* | CCC GTG AAA TGC TGC AAC AA | GTA CCG CAA CAG CTC TGA AAG | 200 | 0.98 ± 0.03 |
| *Hsp90ab1* | GAC CTG CCC CTG AAC ATC TC | GGC GTC GGT TAG TGG AAT CT | 196 | 1.04 ± 0.02 |
| *Hspa5* | CAC TGT GGT ACC CAC CAA GA | GCA GGA GGA ATT CCA GTC AG | 149 | 1.01 ± 0.02 |
| *Lcn2* | ATG TCA CCT CCA TCC TGG TC | GTG GCC ACT TGC ACA TTG TA | 148 | 0.97 ± 0.03 |
| *Lgals3* | GCC TAC CCC AGT GCT CCT | TTG CGT TGG GTT TCA CTG TG | 151 | 0.98 ± 0.02 |
| *Mki67* | TGC AAA GGT AGA GGC TCC AT | CAG GTA GGC CAG AGC AAG T | 152 | 0.98 ± 0.03 |
| *Nupr1* | ACC CTT CCC AGC AAC CTC TA | TGG AAC TTG GTC AGC AGC TT | 187 | 0.97 ± 0.02 |
| *Pcna* | TTG GAA TCC CAG AAC AGG AG | ATT GCC AAG CTC TCC ACT TG | 155 | 1.04 ± 0.03 |
| *Ppia* | CGT CTC CTT CGA GCT GTT TG | CCA CCC TGG CAC ATG AAT C | 139 | 1.02 ± 0.02 |
| *Ptprc* | GGA GAC CAG GAA GTC TGT GC | GTT CTG GGC TCC TTC CTC TT | 145 | 0.97 ± 0.03 |
| *Retreg1* | ACT TGA CCA GGC AGA GCT AG | TTT ATC TGT GCT TCG CTG GC | 160 | 1.01 ± 0.02 |
| *Rtn3* | AGA GTG TGC TTT CCC CTC TC | TCC TCC TCC ACA GAC TGA GA | 187 | 0.99 ± 0.01 |
| *Sec61a1* | CGT TGG TGG CCT GTG TTA CT | CAC CAT CTG CTG CTC CTT CA | 190 | 1.02 ± 0.03 |
| *Sec62* | TCT GGC CAG CAG AAA TGA GA | CAG TCA GGT TTG GCA GGA AC | 164 | 0.97 ± 0.02 |
| *Slc12a1* | ATT GGC CTG AGC GTA GTT GT | AGC AAA GAT CAA GCC TAT TGA CC | 150 | 1.01 ± 0.03 |
| *Sqstm1* | CCC CAA TGT GAT CTG TGA TG | AAG GGG TTG GGA AAG ATG AG | 127 | 0.99 ± 0.03 |
| *Tgfb1* | GTG GAA ATC AAC GGG ATC AG | GTT GGT ATC CAG GGC TCT C | 150 | 0.96 ± 0.03 |
| *Tlr4* | GTG GCC CTA CCA AGT CTC AG | GAC CCA TGA AAT TGG CAC TC | 154 | 1.01 ± 0.02 |
| *Umod* | TCA GCC TGA AGA CCT CCC TA | GAA AAG CCT CAG TGG ACA GC | 156 | 0.99 ± 0.02 |
| *Xbp1s* | GCC ATT GTC TGA GAC CAC CT | AGC TGG GGG AAA AGT TCA TT | 151 | 0.98 ± 0.04 |

## Appendix Table S15: RNA-Seq quality and yield.

| Sample | Genotype (*Umod*) | Age (months) | Yeld (Mbp) | %Q30 | Mean Q |
|---|---|---|---|---|---|
| WT_1.1 | +/+ | 1 | 4'802 | 91.63 | 34.75 |
| WT_1.2 | +/+ | 1 | 6'401 | 90.87 | 34.47 |
| WT_1.3 | +/+ | 1 | 5'373 | 93.09 | 35.16 |
| C171Y_1.1 | C171Y/+ | 1 | 5'937 | 91.76 | 34.82 |
| C171Y_1.2 | C171Y/+ | 1 | 6'435 | 95.13 | 35.73 |
| C171Y_1.3 | C171Y/+ | 1 | 5'301 | 91.87 | 34.83 |
| R186S_1.1 | R186S/+ | 1 | 5'132 | 92.7 | 35.07 |
| R186S_1.2 | R186S/+ | 1 | 4.891 | 92.81 | 35.07 |
| R186S_1.3 | R186S/+ | 1 | 5'212 | 89.28 | 34.22 |
| WT_4.1 | +/+ | 4 | 6'592 | 90.15 | 34.45 |
| WT_4.2 | +/+ | 4 | 7'648 | 91.63 | 34.75 |
| WT_4.3 | +/+ | 4 | 8'959 | 93.77 | 35.42 |
| C171Y_4.1 | C171Y/+ | 4 | 6'932 | 92.41 | 35.01 |
| C171Y_4.2 | C171Y/+ | 4 | 9'851 | 93.28 | 35.36 |
| C171Y_4.3 | C171Y/+ | 4 | 7'213 | 92.23 | 34.97 |
| R186S_4.1 | R186S/+ | 4 | 6'392 | 91.81 | 34.79 |
| R186S_4.2 | R186S/+ | 4 | 6'809 | 91.74 | 34.72 |
| R186S_4.3 | R186S/+ | 4 | 5'311 | 91.77 | 34.98 |

All reads have passed the Illumina chastity filter. %Q30, percentage of bases with quality score ≥30; Mean Q, prediction of the probability of a wrong base call.

## Appendix References


Dahan K, Devuyst O, Smaers M, Vertommen D, Loute G, Poux JM, Viron B, Jacquot C, Gagnadoux MF, Chauveau D *et al* (2003) A cluster of mutations in the UMOD gene causes familial juvenile hyperuricemic nephropathy with abnormal expression of uromodulin. *J Am Soc Nephrol* 14(11):2883-93.

Ioannidis NM, Rothstein JH, Pejaver V, Middha S, McDonnell SK, Baheti S, Musolf A, Li Q, Holzinger E, Karyadi D *et al* (2016) REVEL: An Ensemble Method for Predicting the Pathogenicity of Rare Missense Variants. *Am J Hum Genet* 99(4):877-85.

Karczewski KJ, Francioli LC, Tiao G, Cummings BB, Alföldi J, Wang Q, Collins RL, Laricchia KM, Ganna A, Birnbaum DP *et al* (2020) The mutational constraint spectrum quantified from variation in 141,456 humans. *Nature* 581(7809):434-43.

Kopanos C, Tsiolkas V, Kouris A, Chapple CE, Albarca Aguilera M, Meyer R, Massouras A (2019) VarSome: the human genomic variant search engine. *Bioinformatics* 35(11):1978-80.

Turro E, Astle WJ, Megy K, Gräf S, Greene D, Shamardina O, Allen HL, Sanchis-Juan A, Frontini M, Thys C *et al* (2020) Whole-genome sequencing of patients with rare diseases in a national health system. *Nature* 583(7814):96-102.

Van Hout CV, Tachmazidou I, Backman JD, Hoffman JD, Liu D, Pandey AK, Gonzaga-Jauregui C, Khalid S, Ye B, Banerjee N *et al* (2020) Exome sequencing and characterization of 49,960 individuals in the UK Biobank. *Nature* 586(7831):749-56.
