# Supplementary figures and images for: Allelic effects on uromodulin aggregates drive autosomal dominant tubulointerstitial kidney disease (part 1 of 2)
Source: EMBO Mol Med. 2023 Oct 26;15(12):e18242. doi: 10.15252/emmm.202318242 (PMC10701617; doi:10.15252/emmm.202318242)

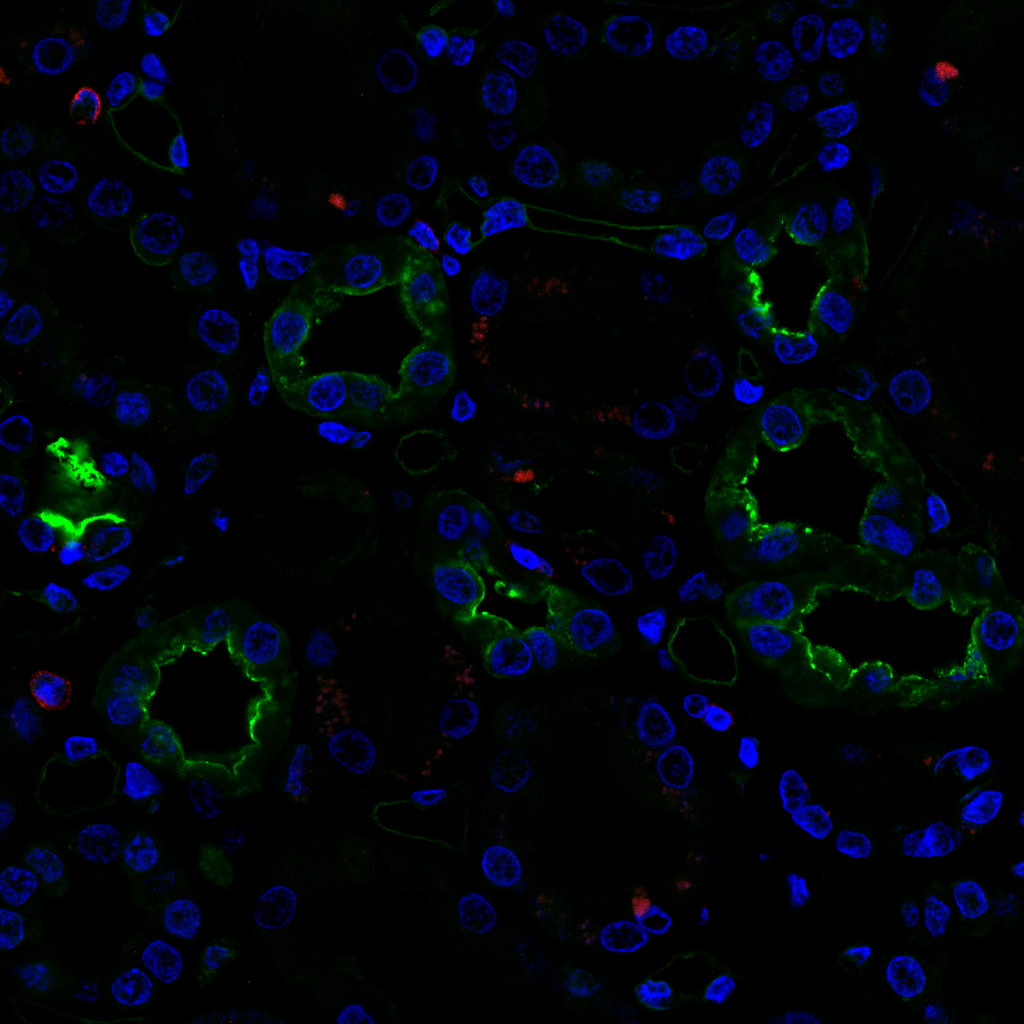

Supplement: Supplementary file 2 — Source Data for Figure 1 [file EMMM-15-e18242-s003.zip › Figure_1/1D/Normal_Kidney_-_UMOD,_CD3.tif]

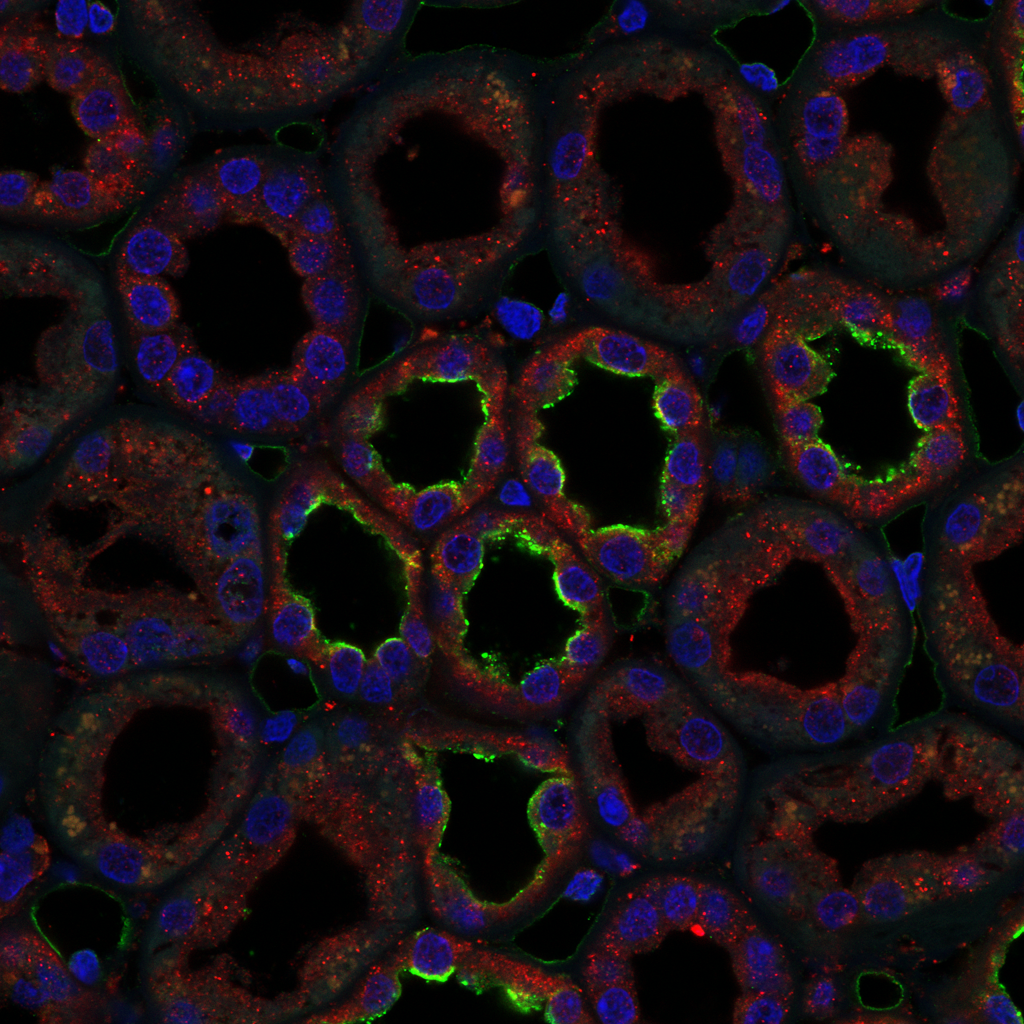

Supplement: Supplementary file 2 — Source Data for Figure 1 [file EMMM-15-e18242-s003.zip › Figure_1/1D/Normal_Kidney_-_UMOD,_GRP78.tif]

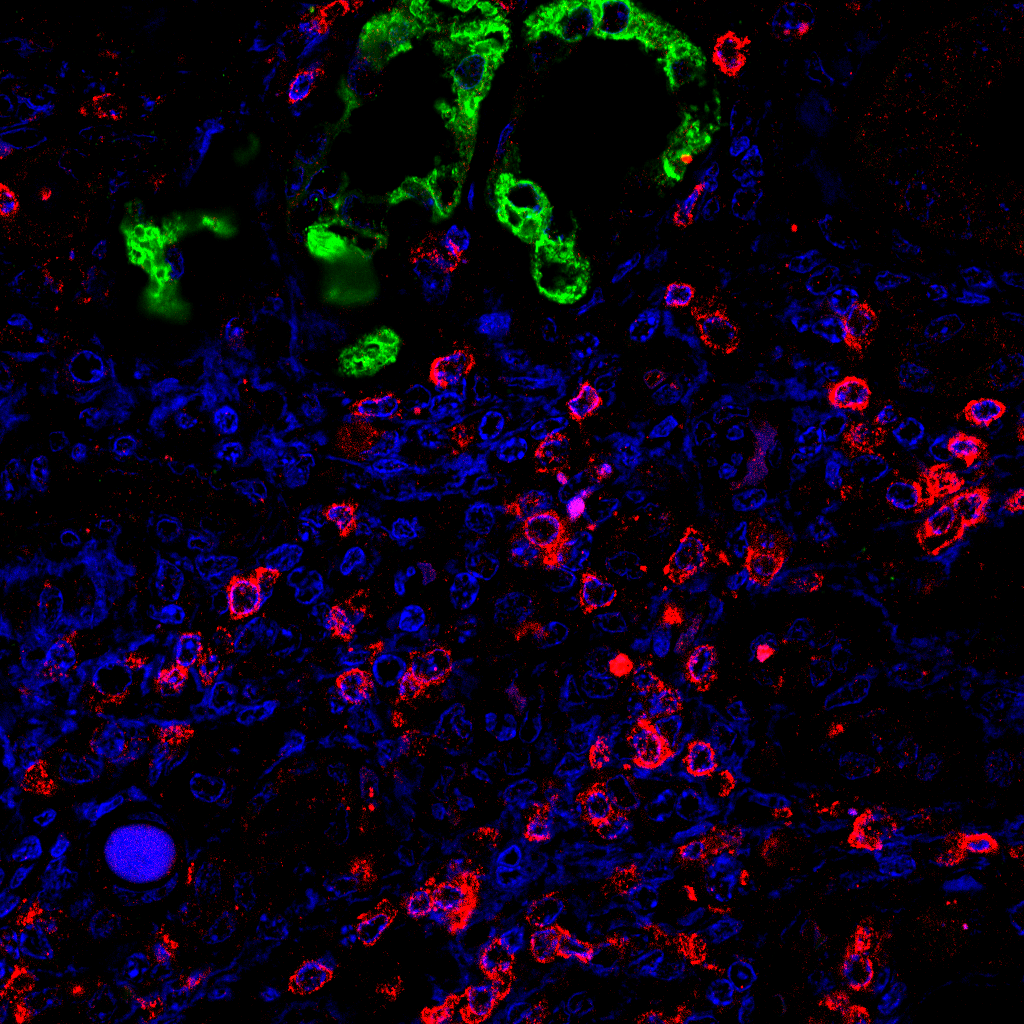

Supplement: Supplementary file 2 — Source Data for Figure 1 [file EMMM-15-e18242-s003.zip › Figure_1/1D/UMOD_p.Arg185Ser_-_UMOD,_CD3.tif]

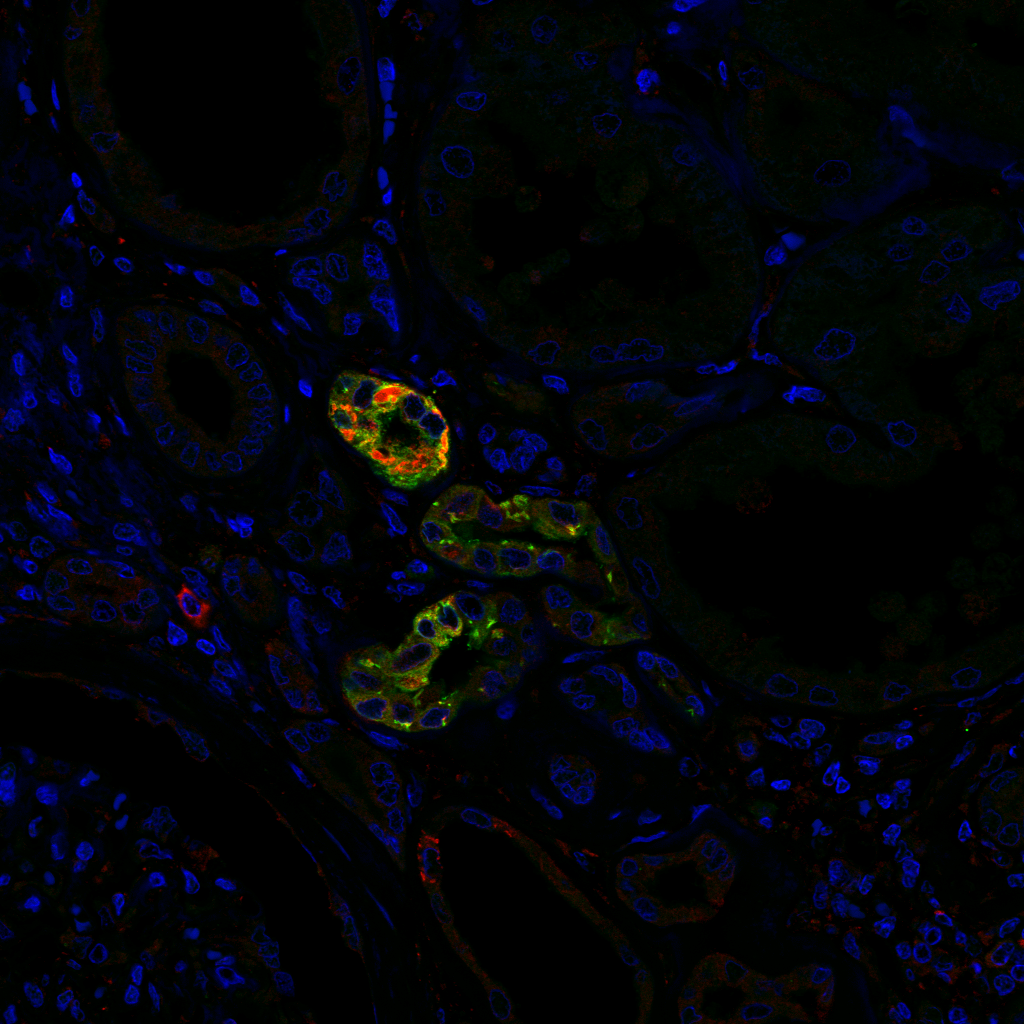

Supplement: Supplementary file 2 — Source Data for Figure 1 [file EMMM-15-e18242-s003.zip › Figure_1/1D/UMOD_p.Arg185Ser_-_UMOD,_GRP78.tif]

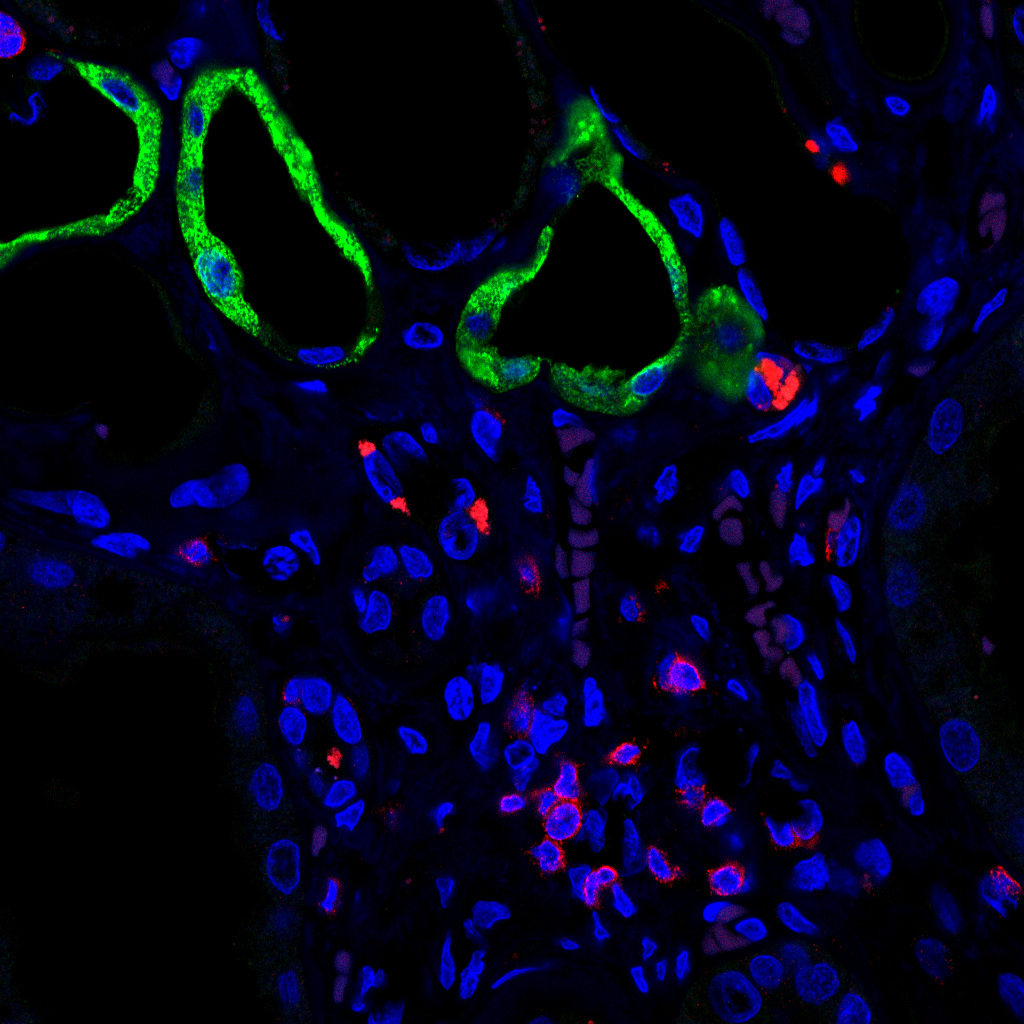

Supplement: Supplementary file 2 — Source Data for Figure 1 [file EMMM-15-e18242-s003.zip › Figure_1/1D/UMOD_p.Cys170Tyr_-_UMOD,_CD3.tif]

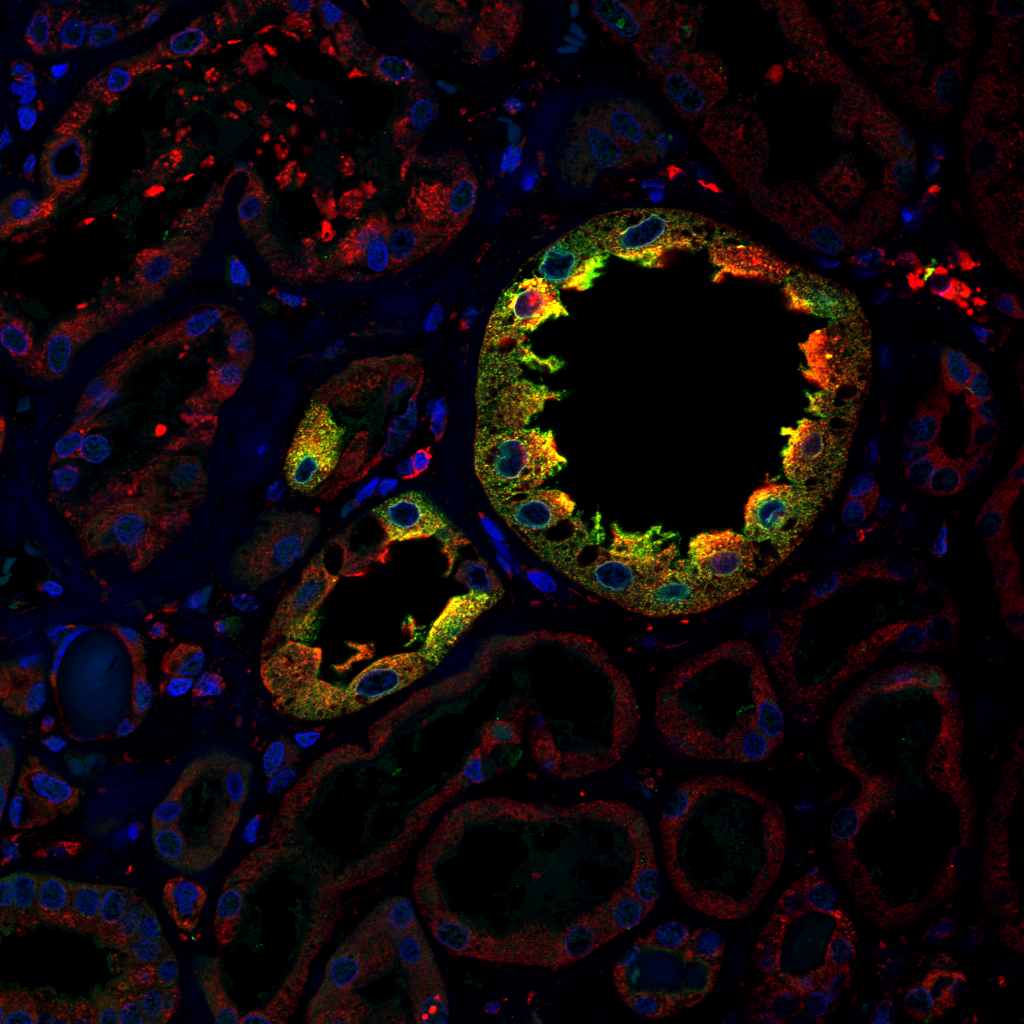

Supplement: Supplementary file 2 — Source Data for Figure 1 [file EMMM-15-e18242-s003.zip › Figure_1/1D/UMOD_p.Cys170Tyr_-_UMOD,_GRP78.tif]

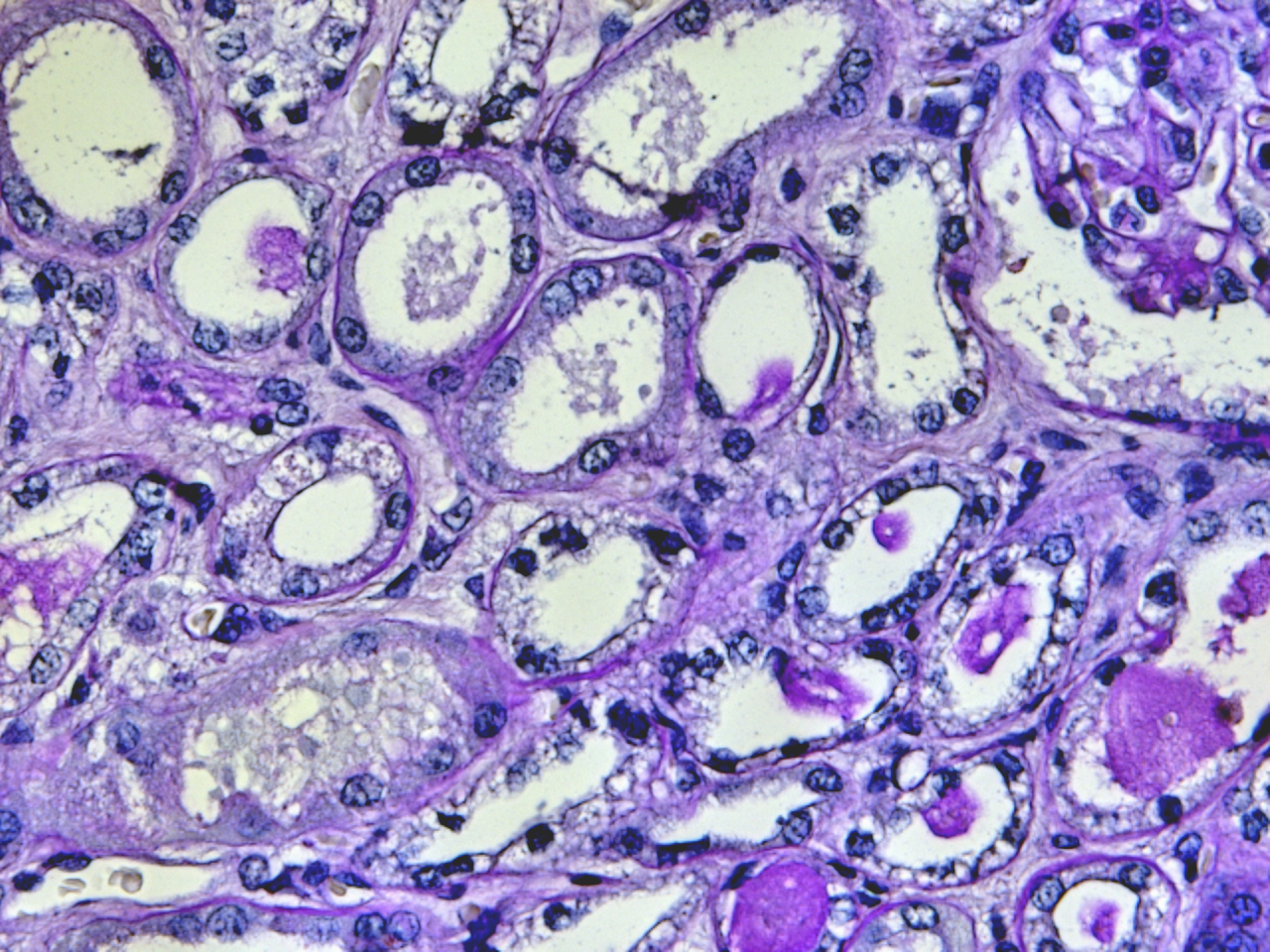

Supplement: Supplementary file 2 — Source Data for Figure 1 [file EMMM-15-e18242-s003.zip › Figure_1/1E/Normal_Kidney_-_PAS.tif]

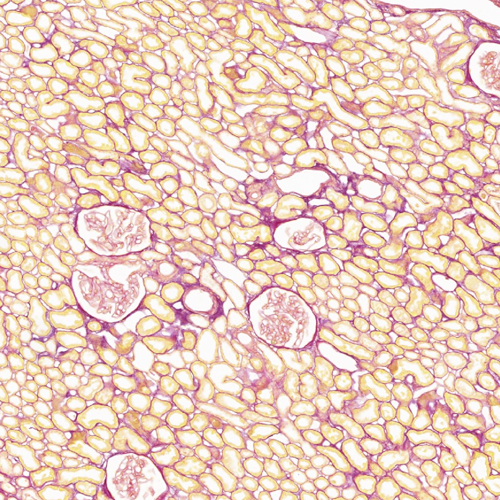

Supplement: Supplementary file 2 — Source Data for Figure 1 [file EMMM-15-e18242-s003.zip › Figure_1/1E/Normal_kidney_-_Picrosirius_red.tif]

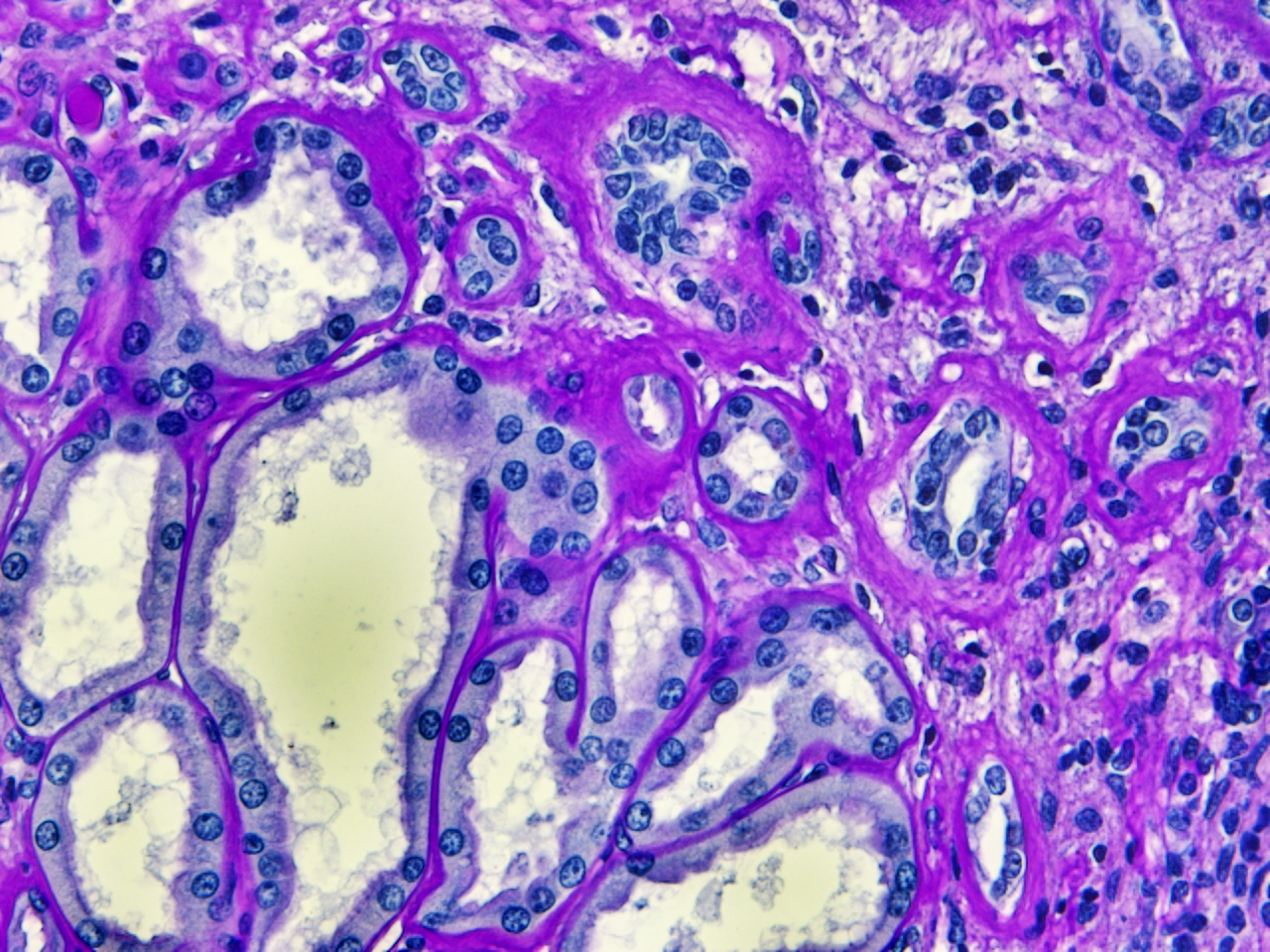

Supplement: Supplementary file 2 — Source Data for Figure 1 [file EMMM-15-e18242-s003.zip › Figure_1/1E/UMOD_p.Arg185Ser_-_PAS.tif]

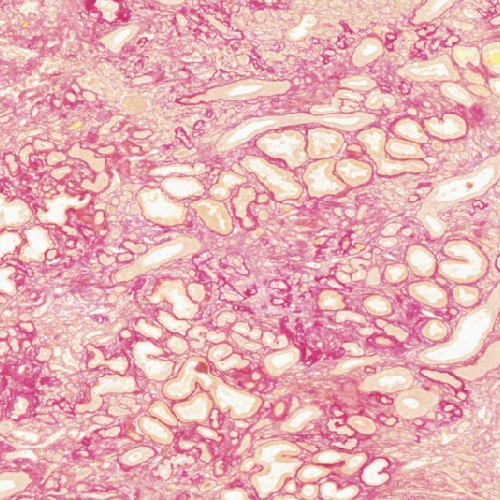

Supplement: Supplementary file 2 — Source Data for Figure 1 [file EMMM-15-e18242-s003.zip › Figure_1/1E/UMOD_p.Arg185Ser_-_Picrosirius_red.tif]

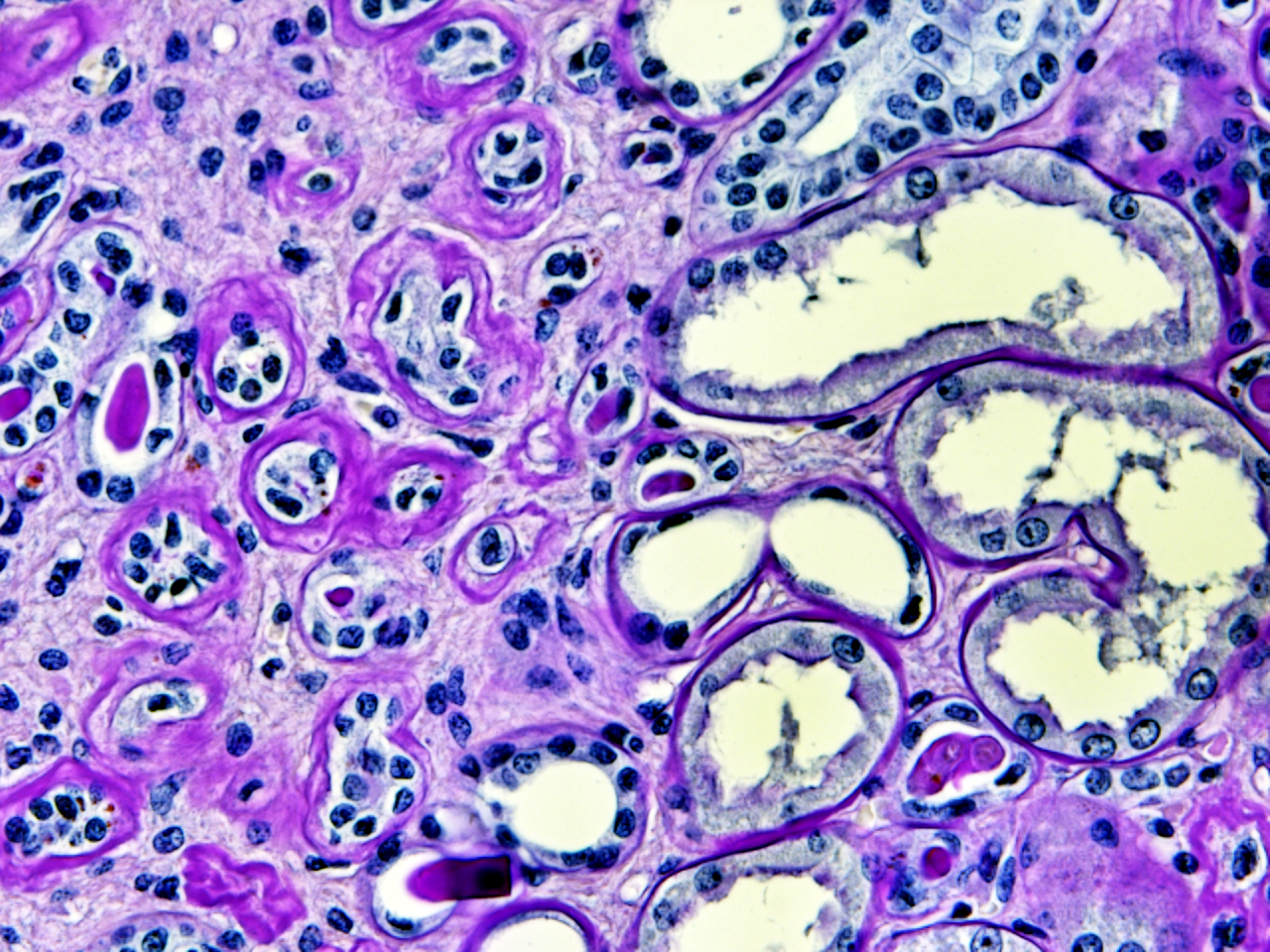

Supplement: Supplementary file 2 — Source Data for Figure 1 [file EMMM-15-e18242-s003.zip › Figure_1/1E/UMOD_p.Cys170Tyr_-_PAS.tif]

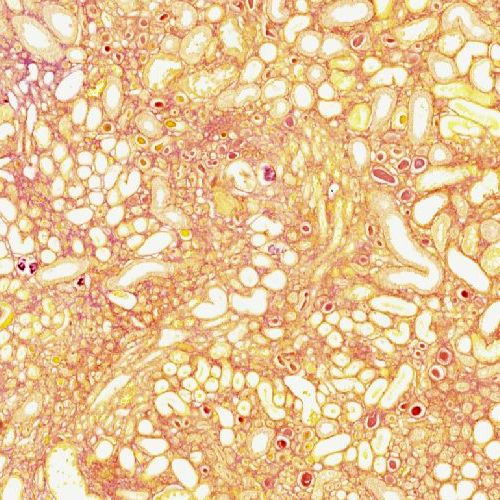

Supplement: Supplementary file 2 — Source Data for Figure 1 [file EMMM-15-e18242-s003.zip › Figure_1/1E/UMOD_p.Cys170Tyr_-_Picrosirius_red.tif]

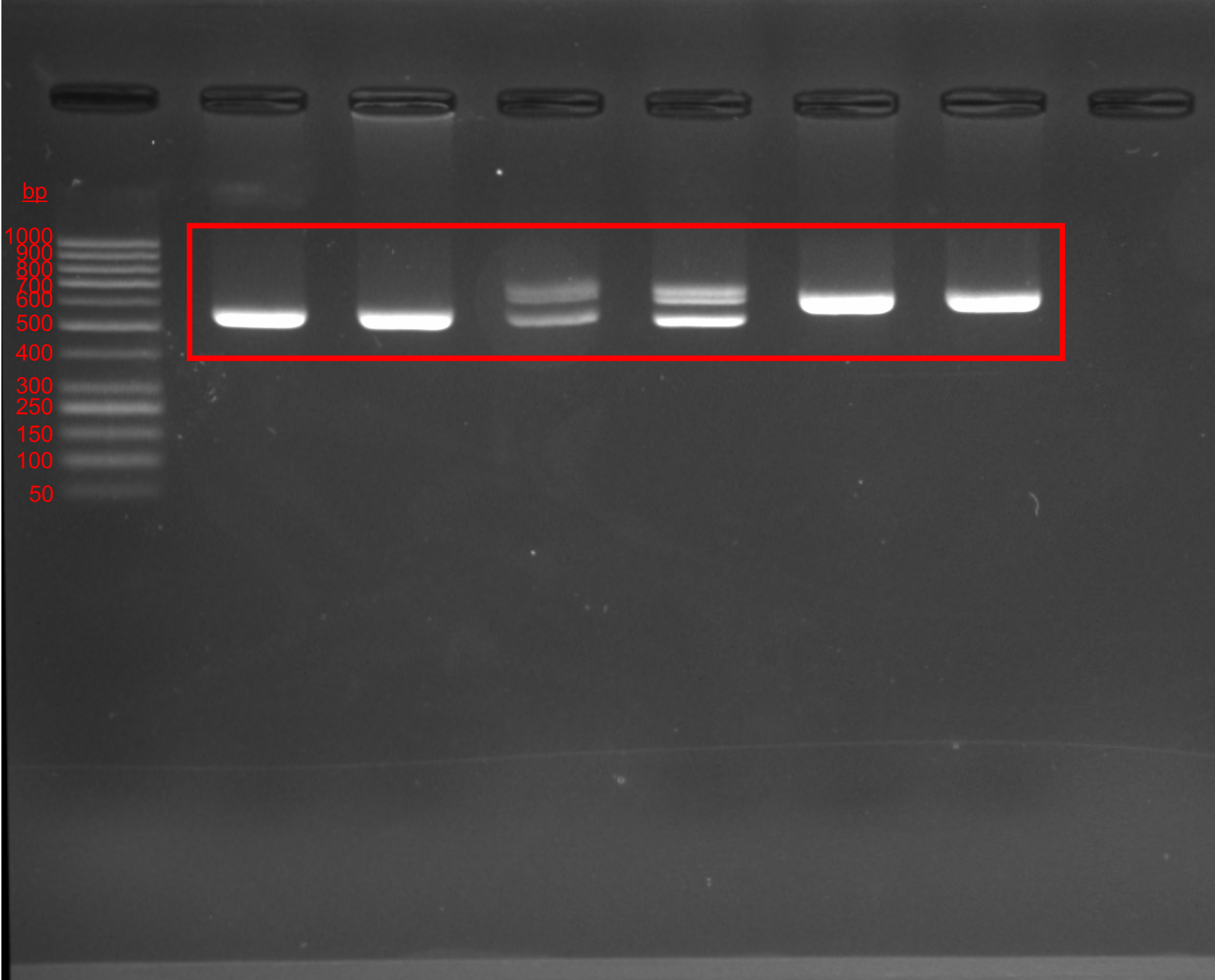

Supplement: Supplementary file 3 — Source Data for Figure 2 [file EMMM-15-e18242-s007.zip › Figure_2/2B/Umod_KI_Genotype.tif]

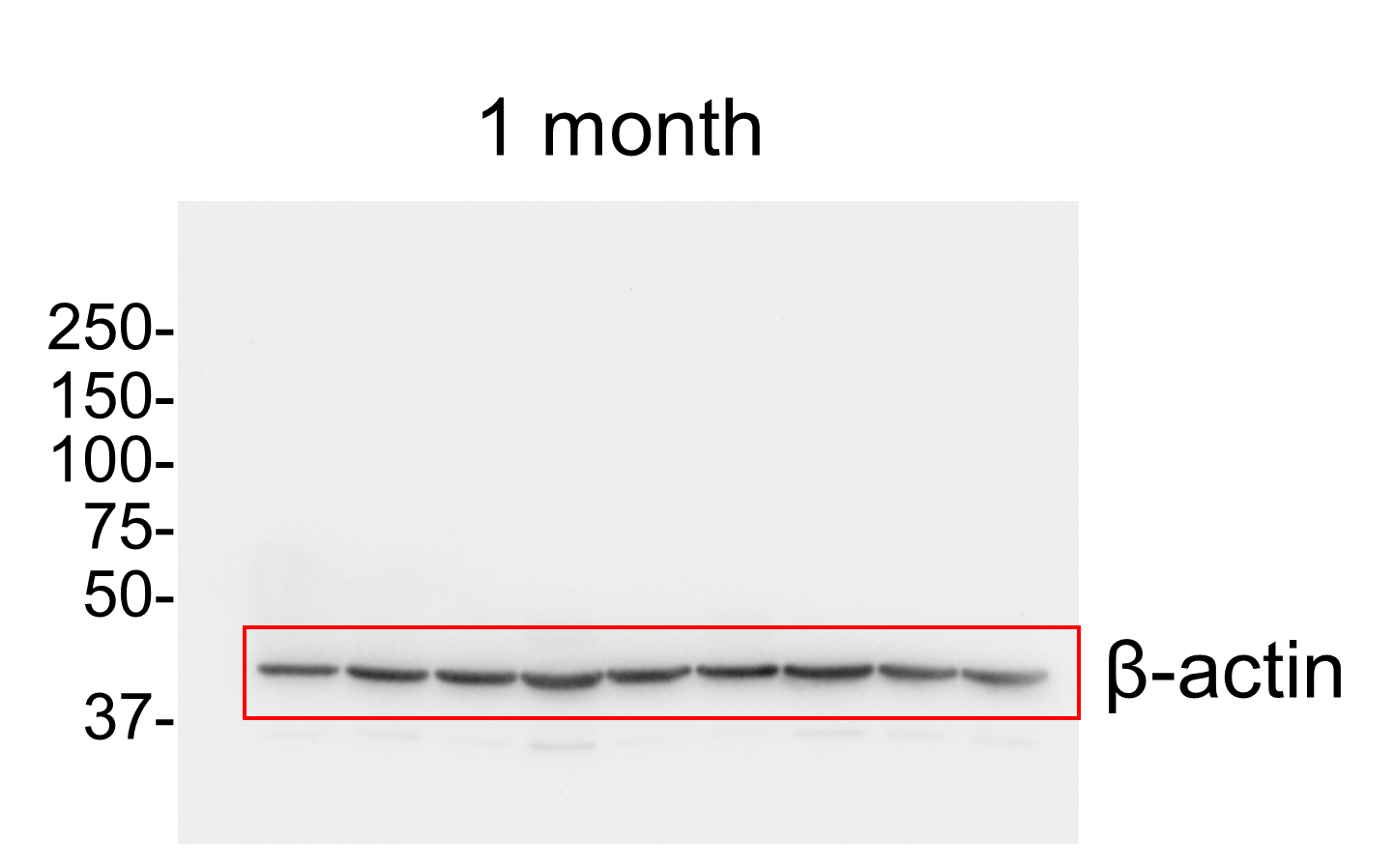

Supplement: Supplementary file 3 — Source Data for Figure 2 [file EMMM-15-e18242-s007.zip › Figure_2/2C/WB_beta-actin_1_mo.tif]

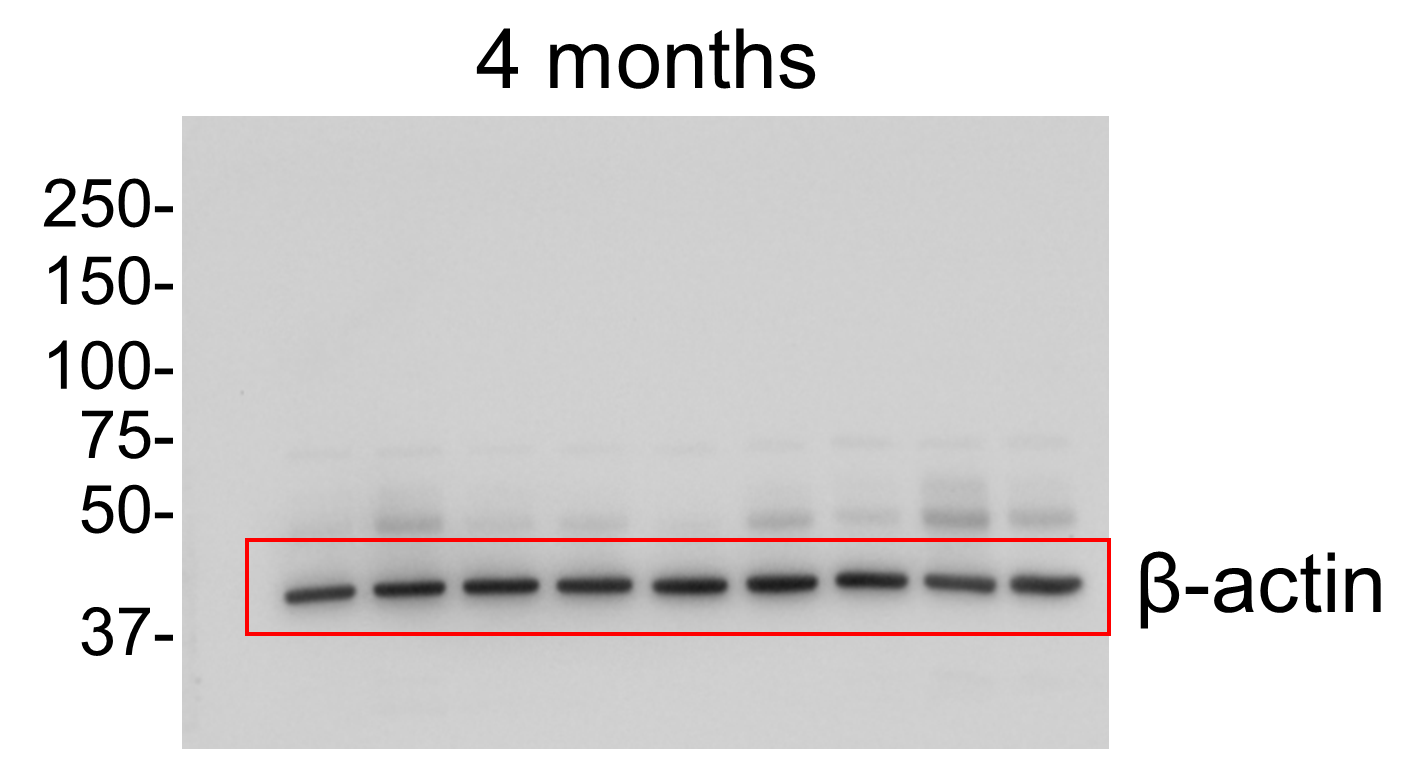

Supplement: Supplementary file 3 — Source Data for Figure 2 [file EMMM-15-e18242-s007.zip › Figure_2/2C/WB_beta-actin_4_mo.tif]

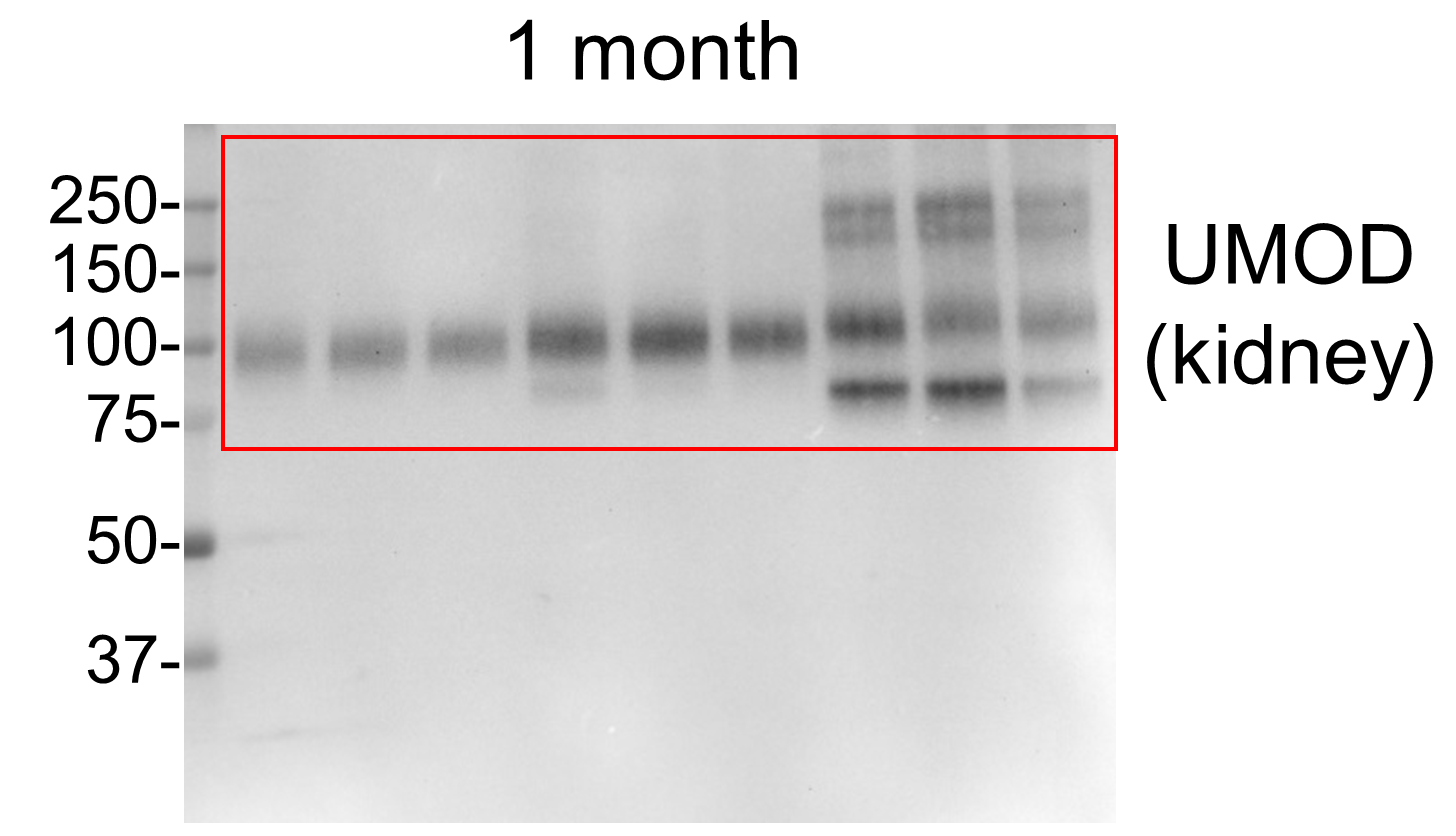

Supplement: Supplementary file 3 — Source Data for Figure 2 [file EMMM-15-e18242-s007.zip › Figure_2/2C/WB_kidney_UMOD_1_mo.tif]

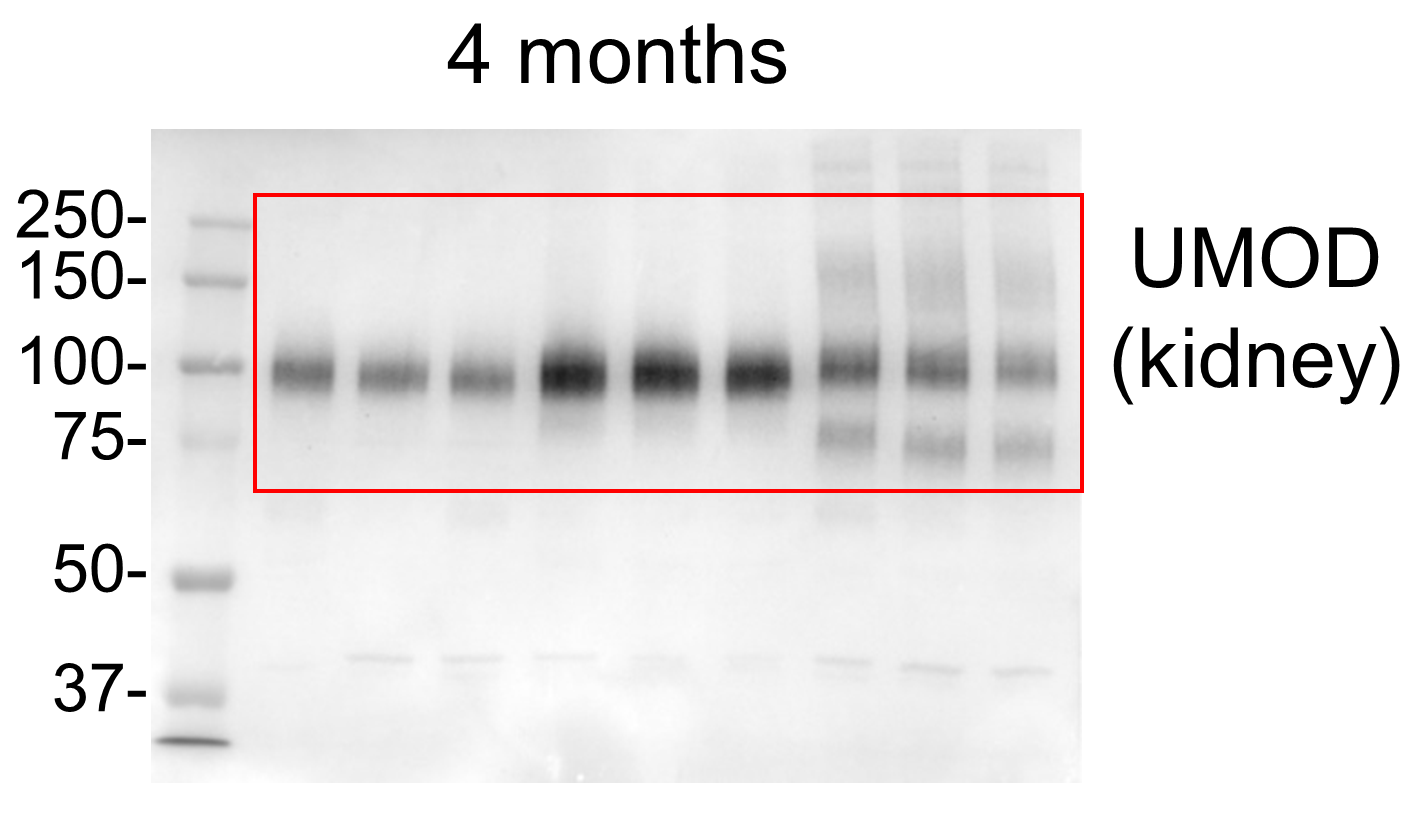

Supplement: Supplementary file 3 — Source Data for Figure 2 [file EMMM-15-e18242-s007.zip › Figure_2/2C/WB_kidney_UMOD_4_mo.tif]

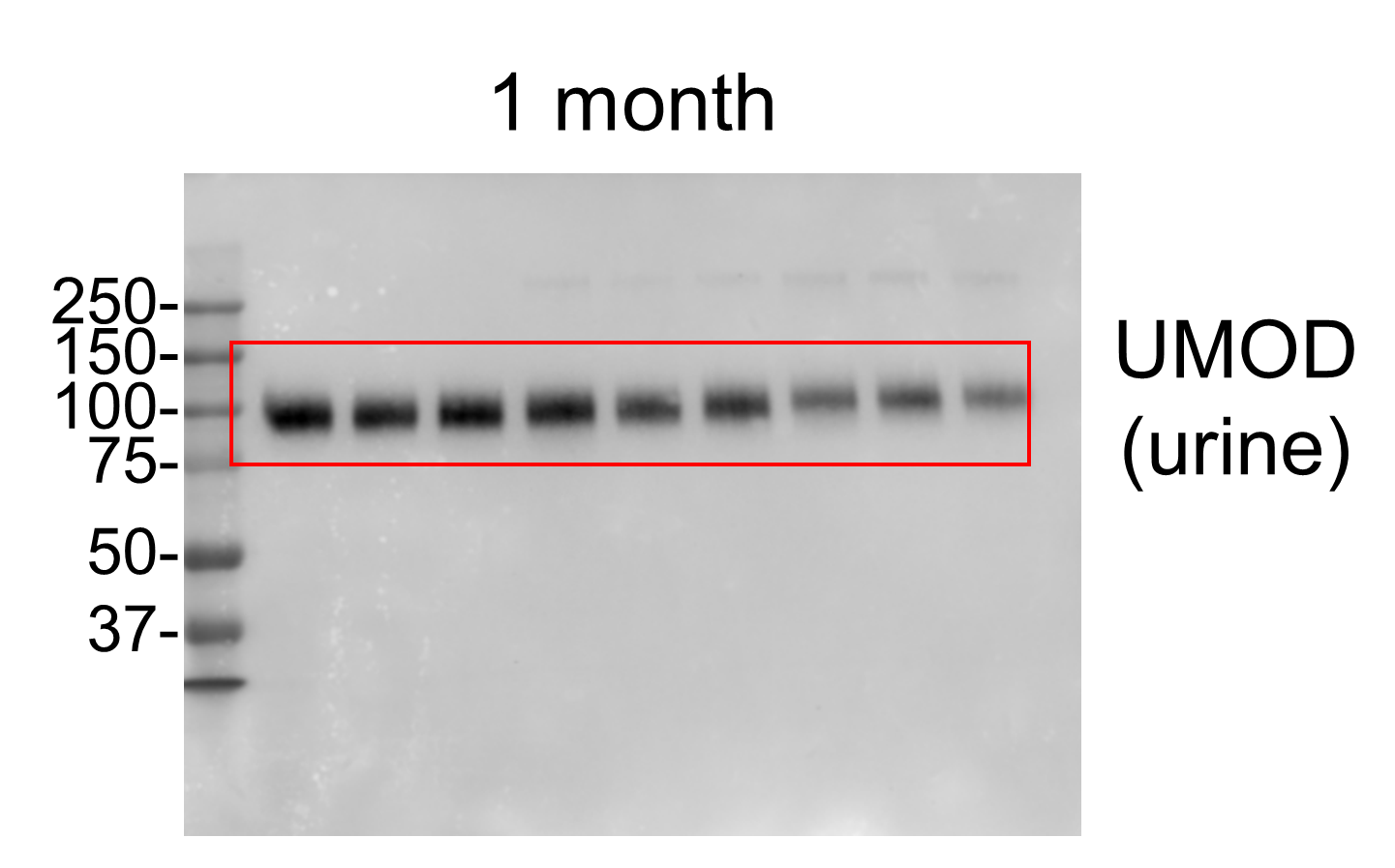

Supplement: Supplementary file 3 — Source Data for Figure 2 [file EMMM-15-e18242-s007.zip › Figure_2/2C/WB_urine_UMOD_1_mo.tif]

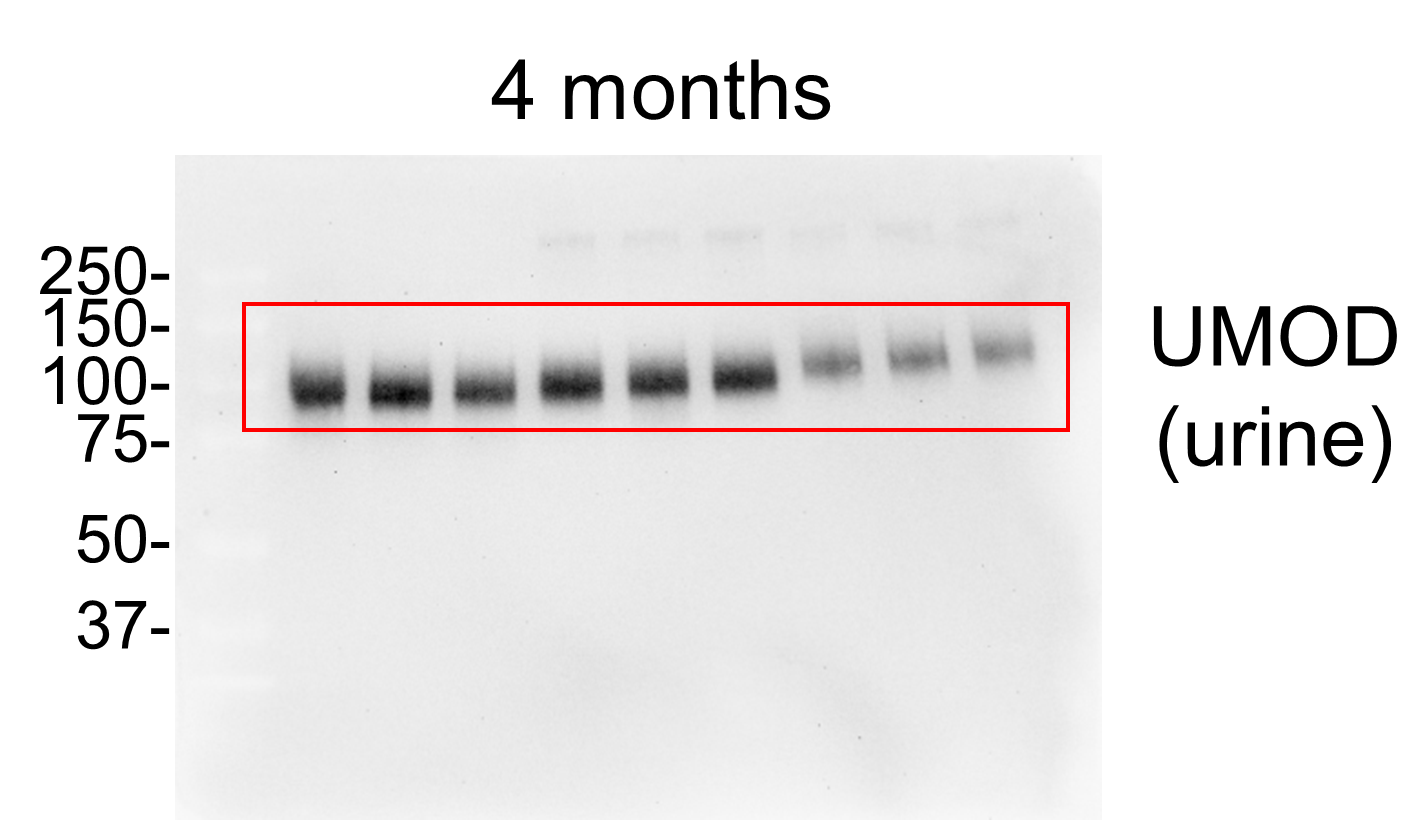

Supplement: Supplementary file 3 — Source Data for Figure 2 [file EMMM-15-e18242-s007.zip › Figure_2/2C/WB_urine_UMOD_4_mo.tif]

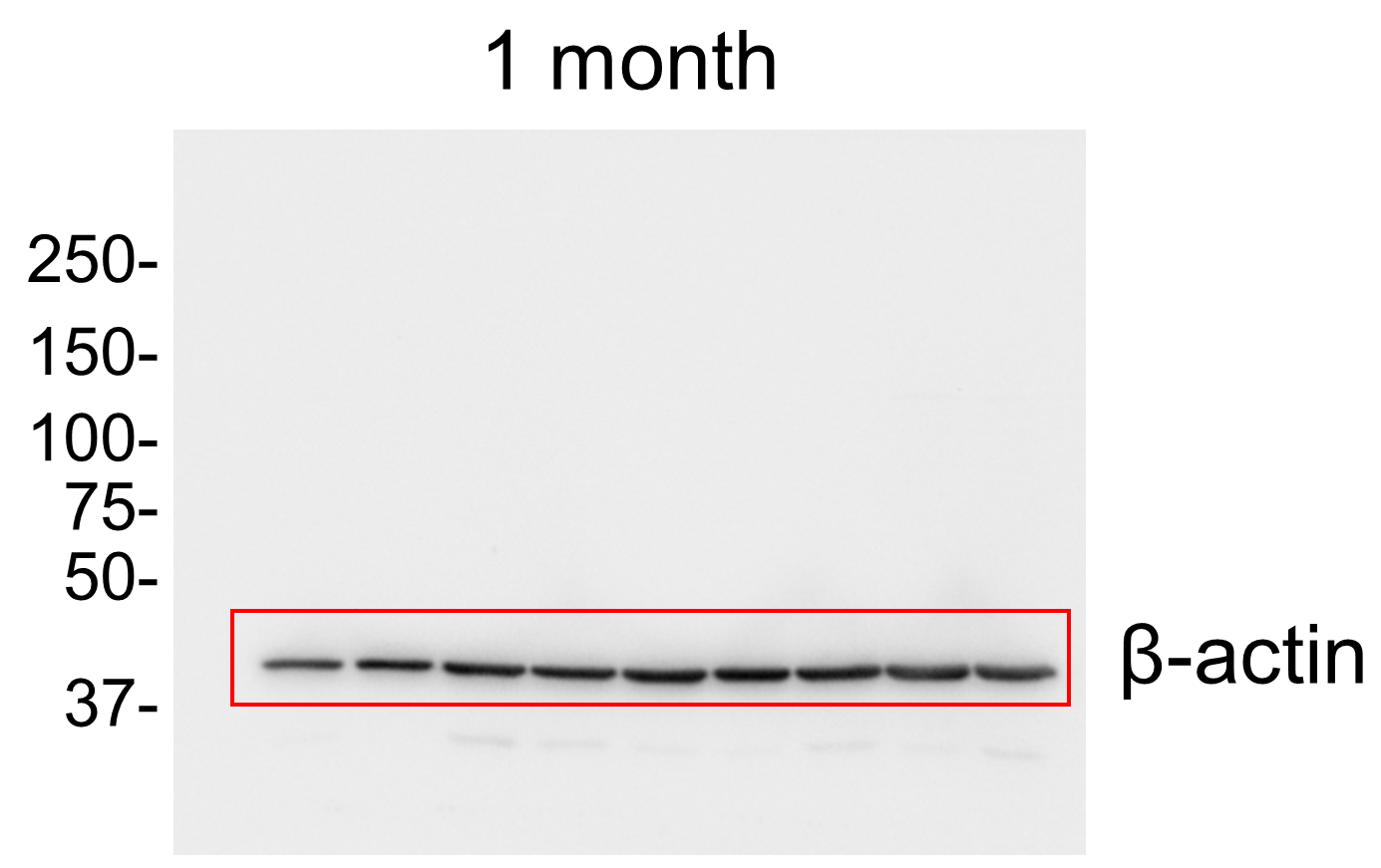

Supplement: Supplementary file 3 — Source Data for Figure 2 [file EMMM-15-e18242-s007.zip › Figure_2/2D/WB_beta-actin_1_mo.tif]

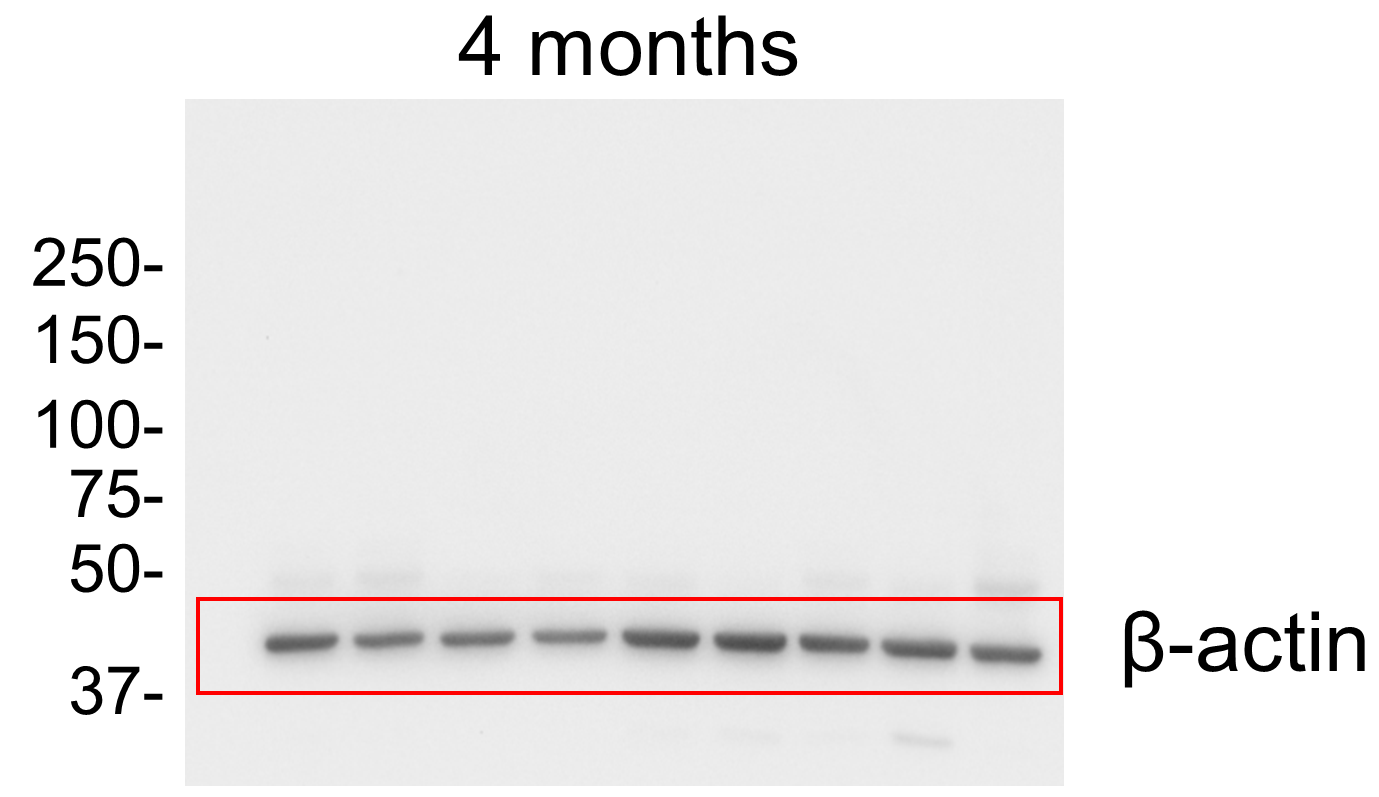

Supplement: Supplementary file 3 — Source Data for Figure 2 [file EMMM-15-e18242-s007.zip › Figure_2/2D/WB_beta-actin_4_mo.tif]

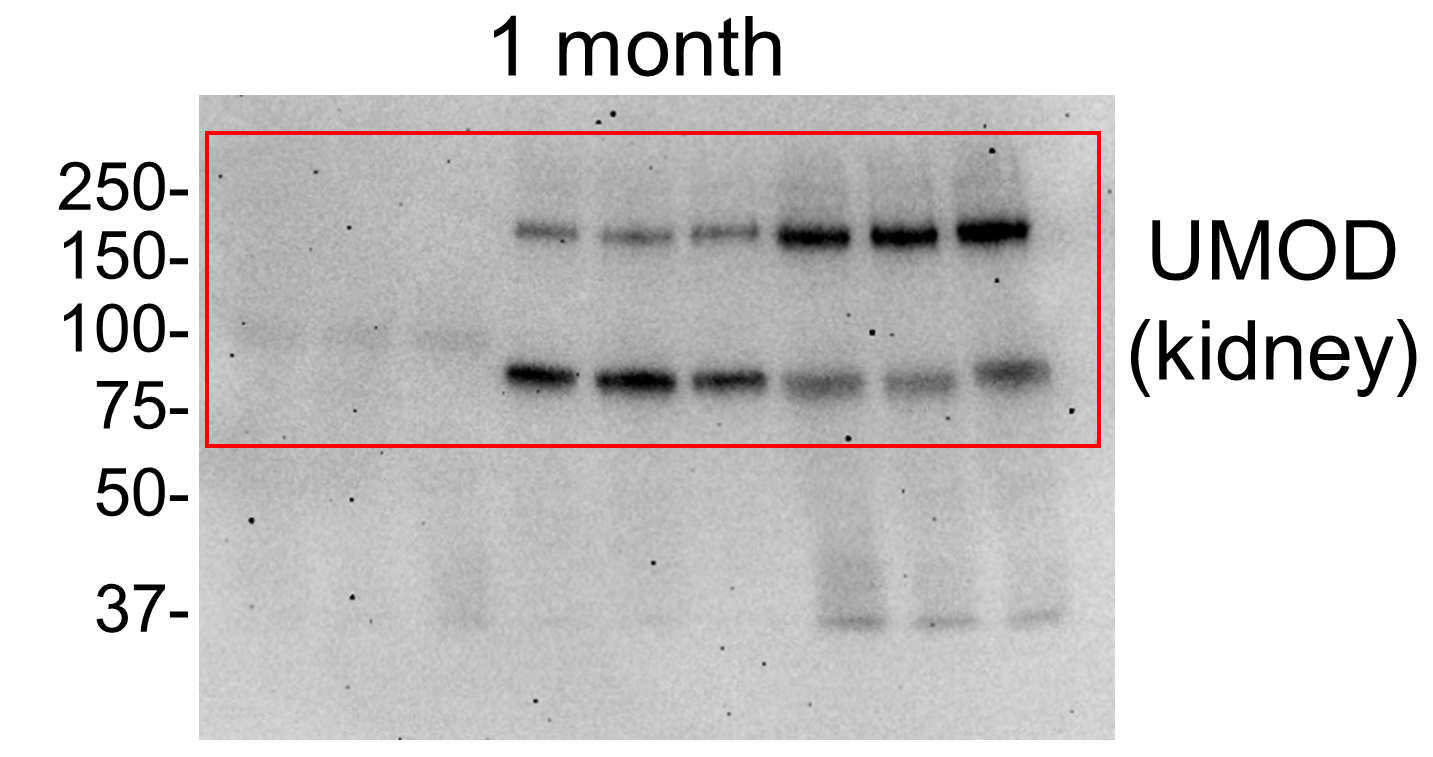

Supplement: Supplementary file 3 — Source Data for Figure 2 [file EMMM-15-e18242-s007.zip › Figure_2/2D/WB_kidney_UMOD_1_mo.tif]

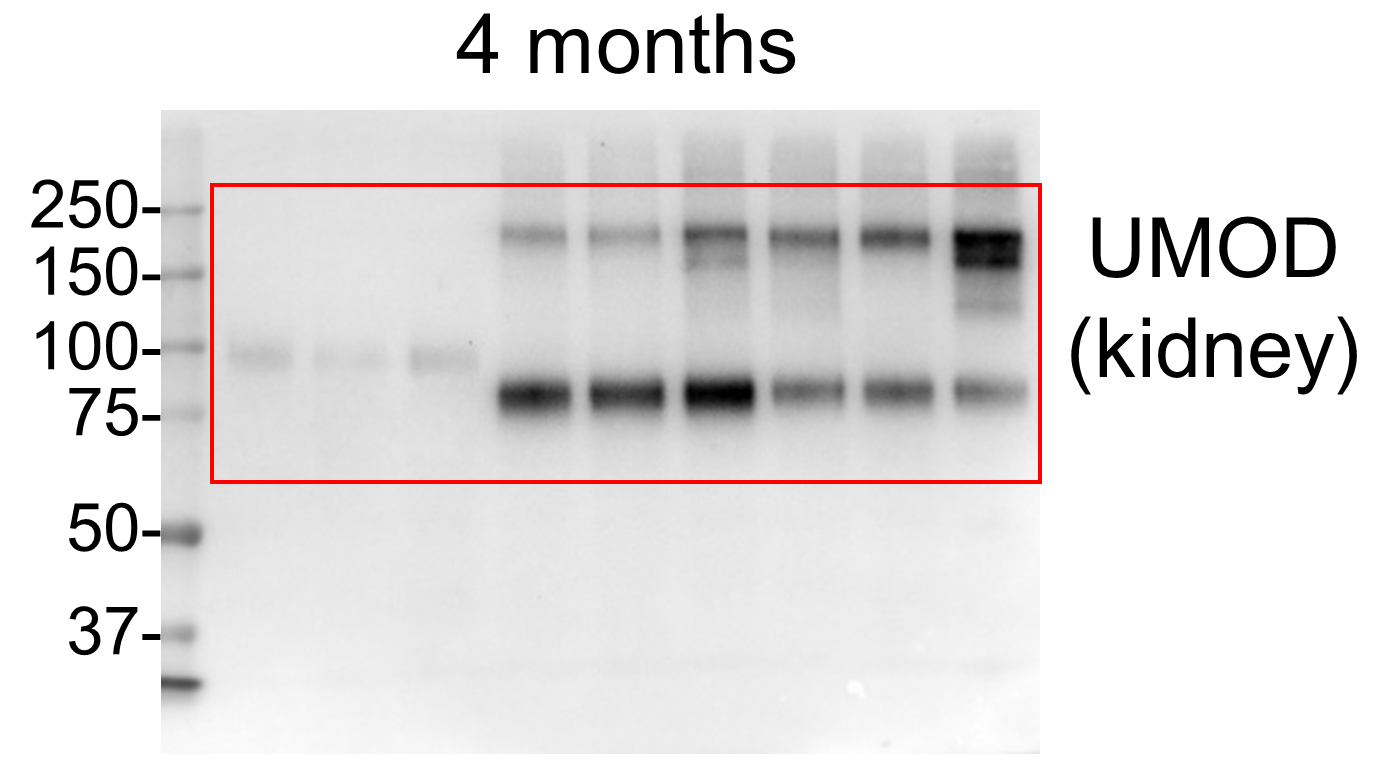

Supplement: Supplementary file 3 — Source Data for Figure 2 [file EMMM-15-e18242-s007.zip › Figure_2/2D/WB_kidney_UMOD_4_mo.tif]

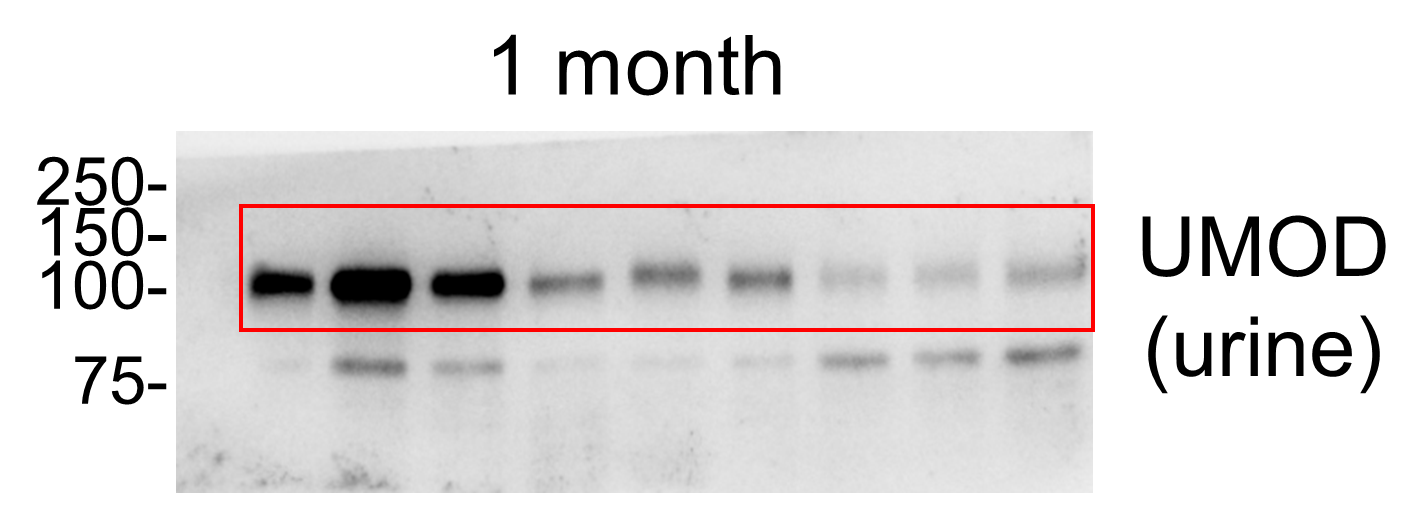

Supplement: Supplementary file 3 — Source Data for Figure 2 [file EMMM-15-e18242-s007.zip › Figure_2/2D/WB_urine_UMOD_1_mo.tif]

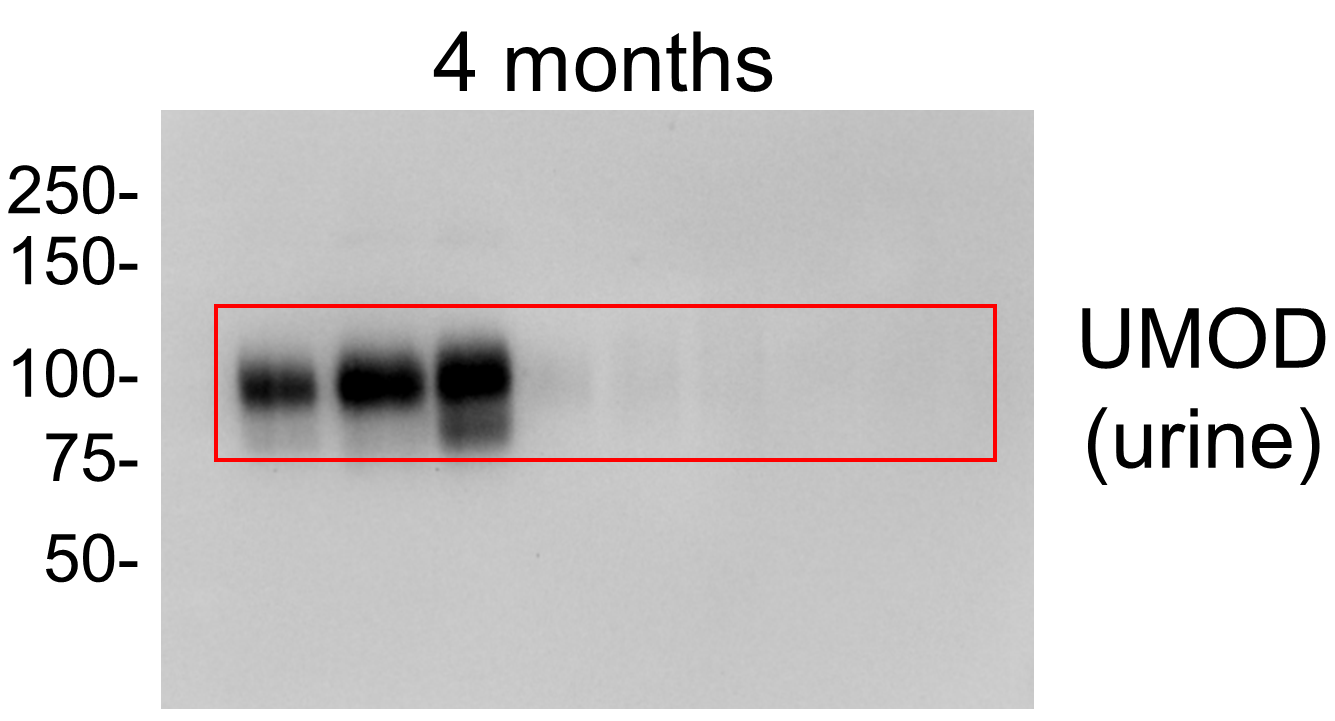

Supplement: Supplementary file 3 — Source Data for Figure 2 [file EMMM-15-e18242-s007.zip › Figure_2/2D/WB_urine_UMOD_4_mo.tif]

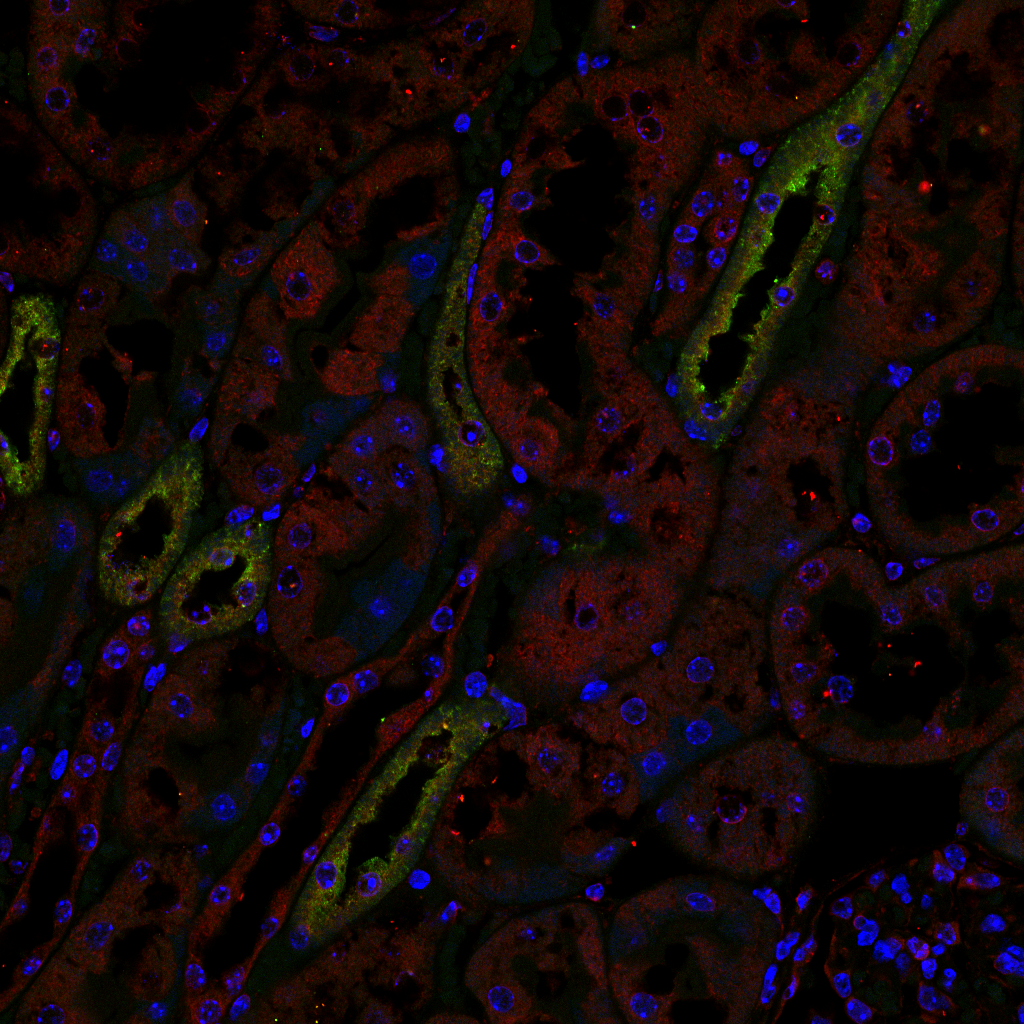

Supplement: Supplementary file 3 — Source Data for Figure 2 [file EMMM-15-e18242-s007.zip › Figure_2/2E/++_-_UMOD,_CNX.tif]

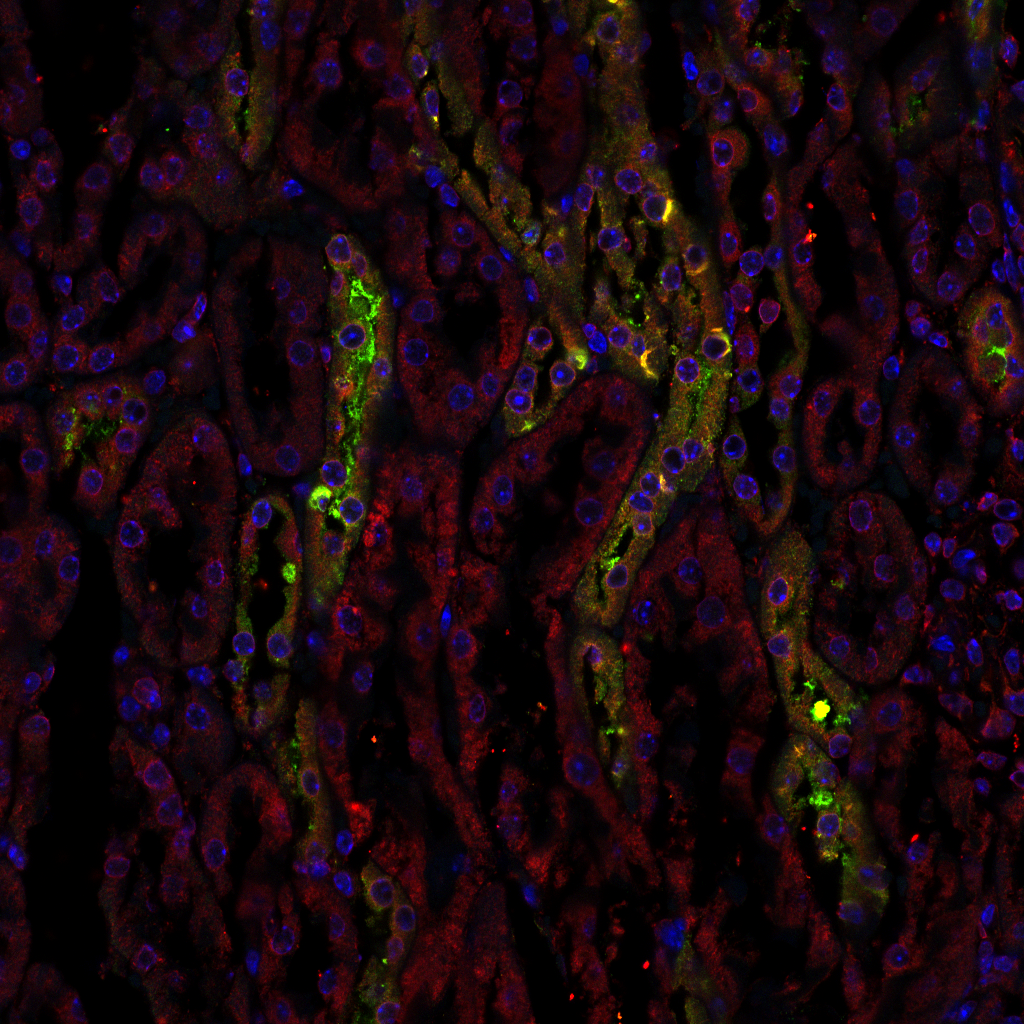

Supplement: Supplementary file 3 — Source Data for Figure 2 [file EMMM-15-e18242-s007.zip › Figure_2/2E/C171Y+_-_UMOD,_CNX.tif]

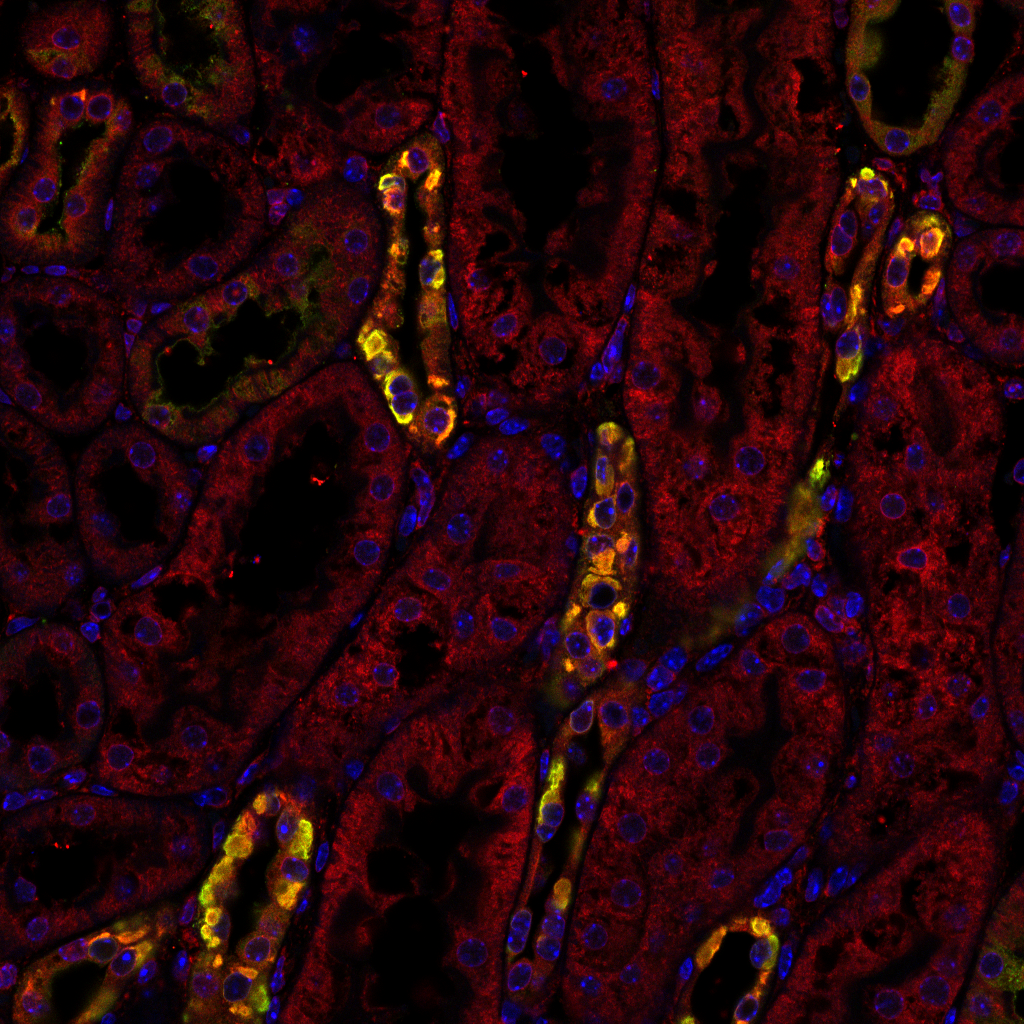

Supplement: Supplementary file 3 — Source Data for Figure 2 [file EMMM-15-e18242-s007.zip › Figure_2/2E/C171YC171Y_-_UMOD,_CNX.tif]

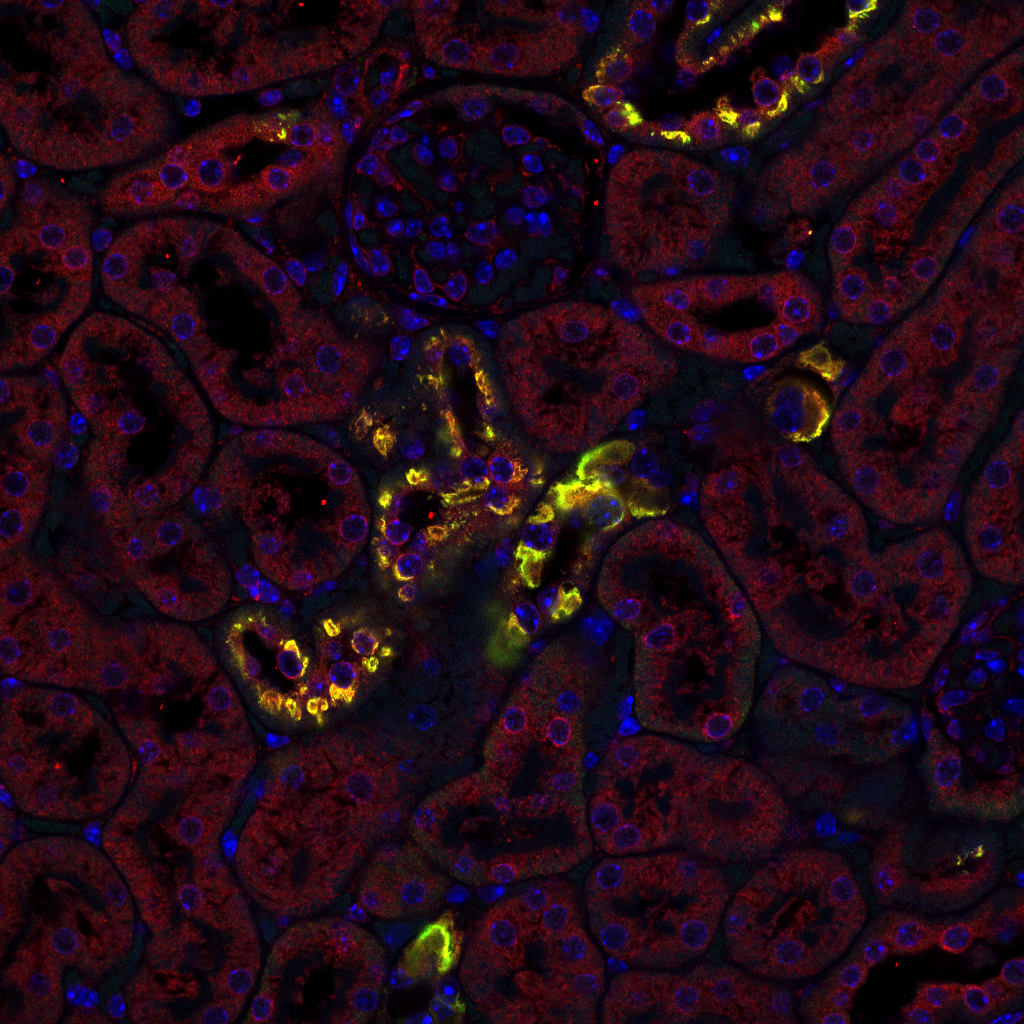

Supplement: Supplementary file 3 — Source Data for Figure 2 [file EMMM-15-e18242-s007.zip › Figure_2/2E/R186S+_-_UMOD,_CNX.tif]

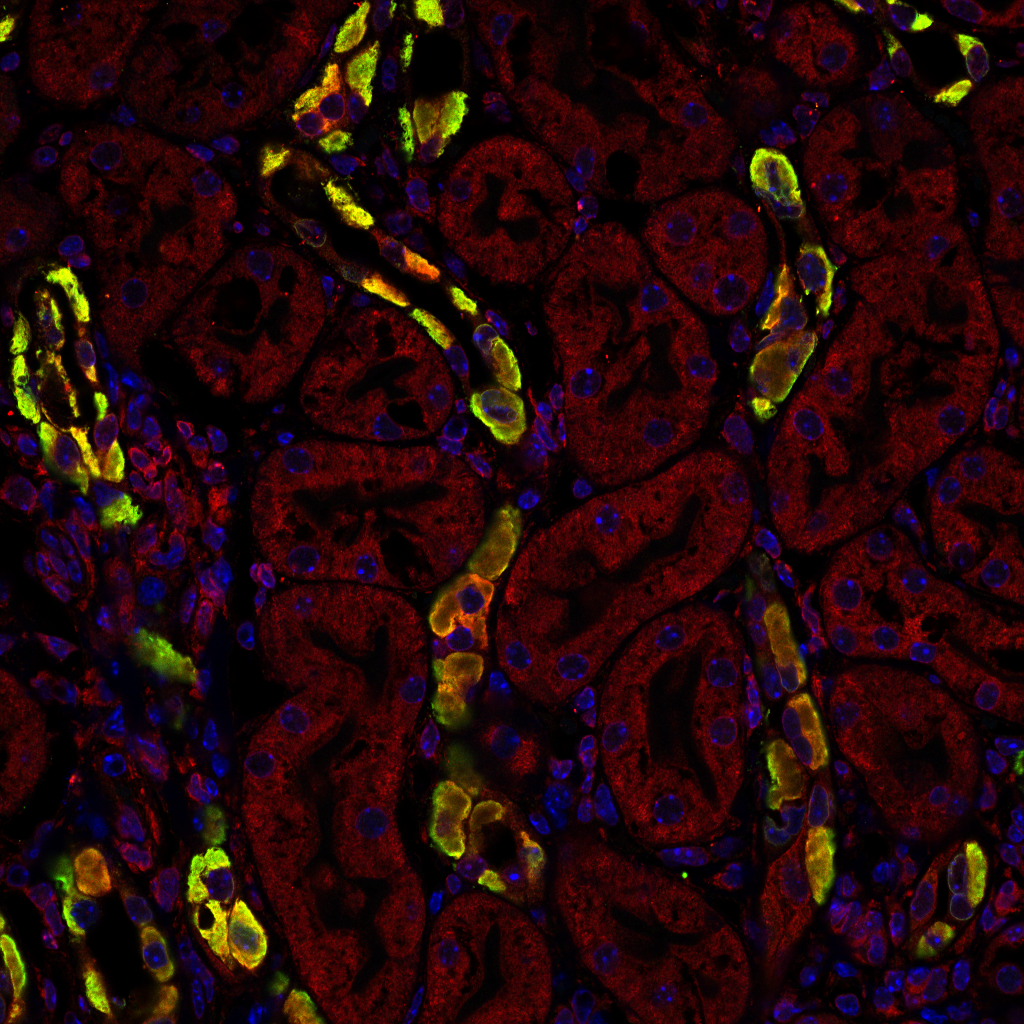

Supplement: Supplementary file 3 — Source Data for Figure 2 [file EMMM-15-e18242-s007.zip › Figure_2/2E/R186SR186S_-_UMOD,_CNX.tif]

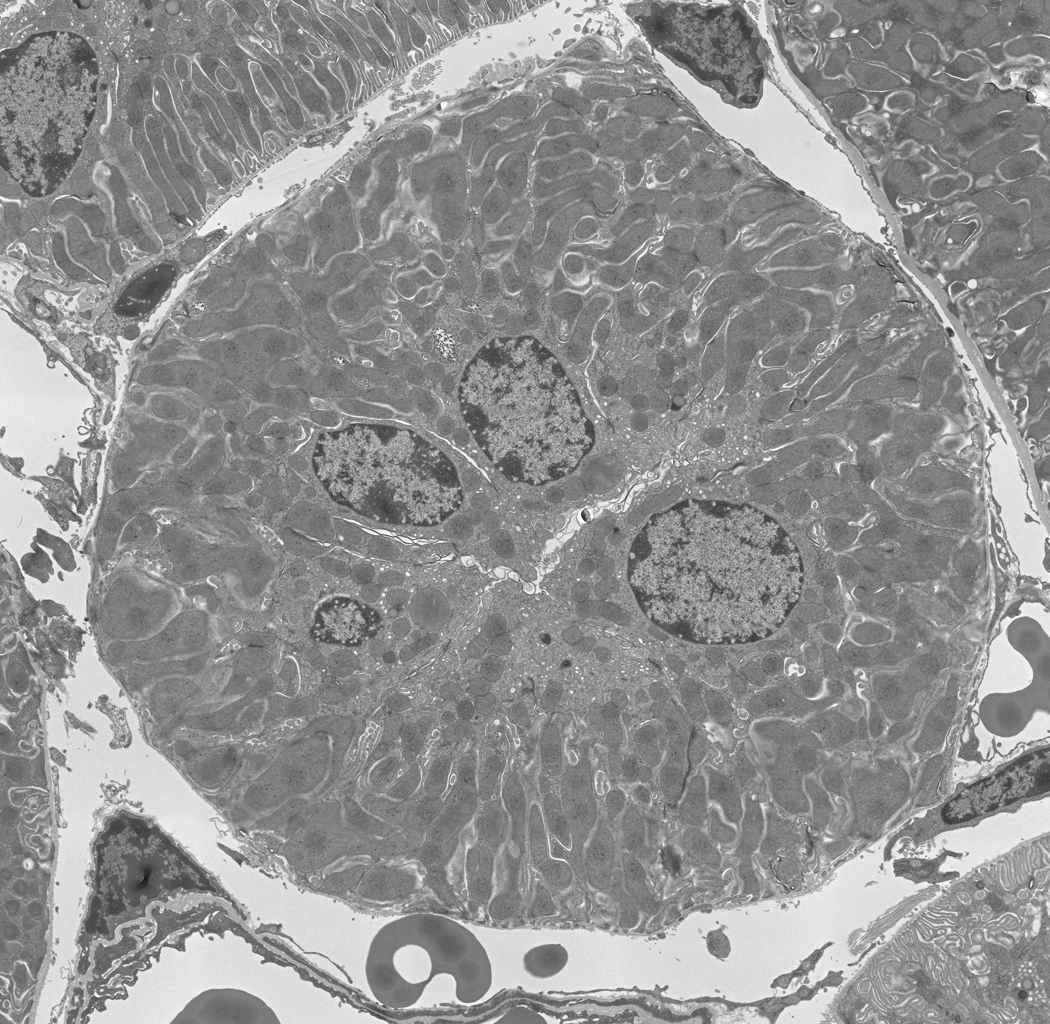

Supplement: Supplementary file 3 — Source Data for Figure 2 [file EMMM-15-e18242-s007.zip › Figure_2/2F/++_EM.tif]

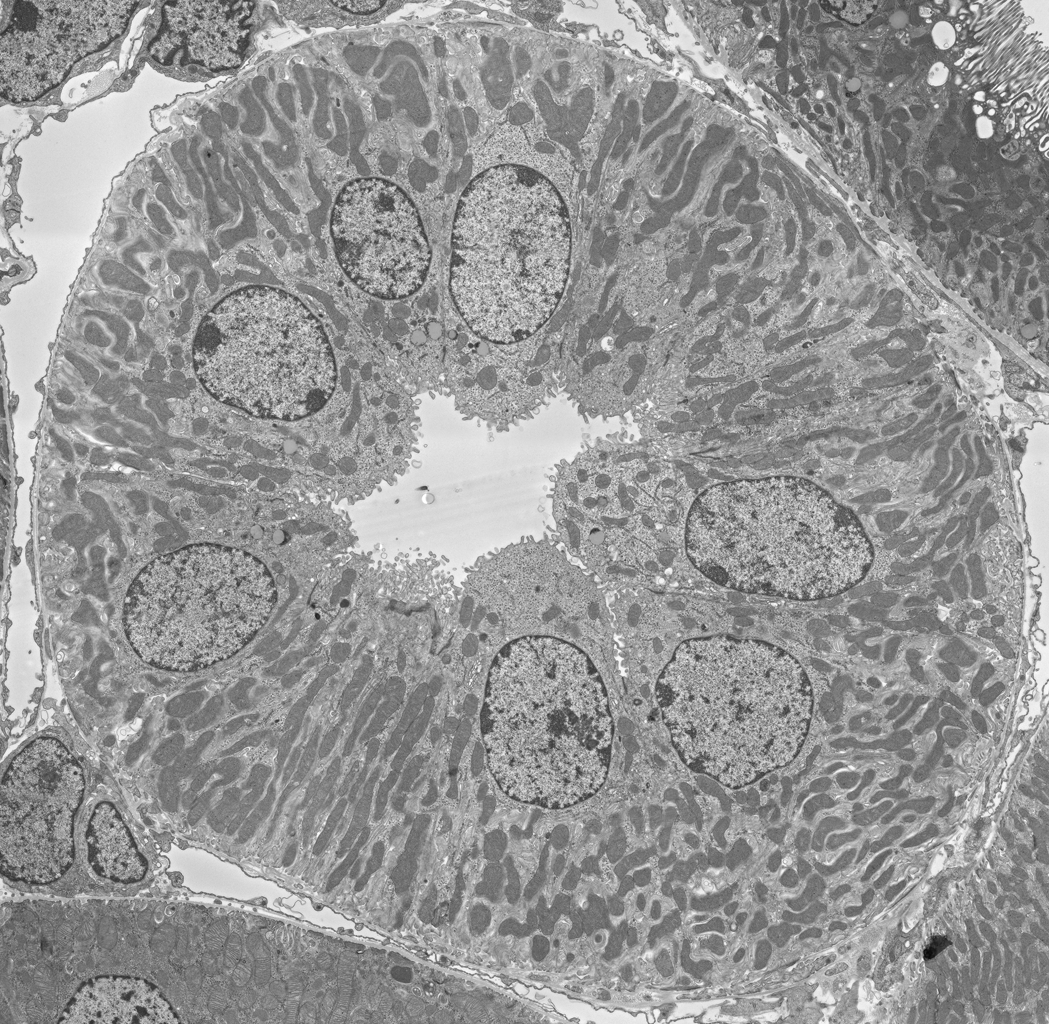

Supplement: Supplementary file 3 — Source Data for Figure 2 [file EMMM-15-e18242-s007.zip › Figure_2/2F/C171Y+_EM.tif]

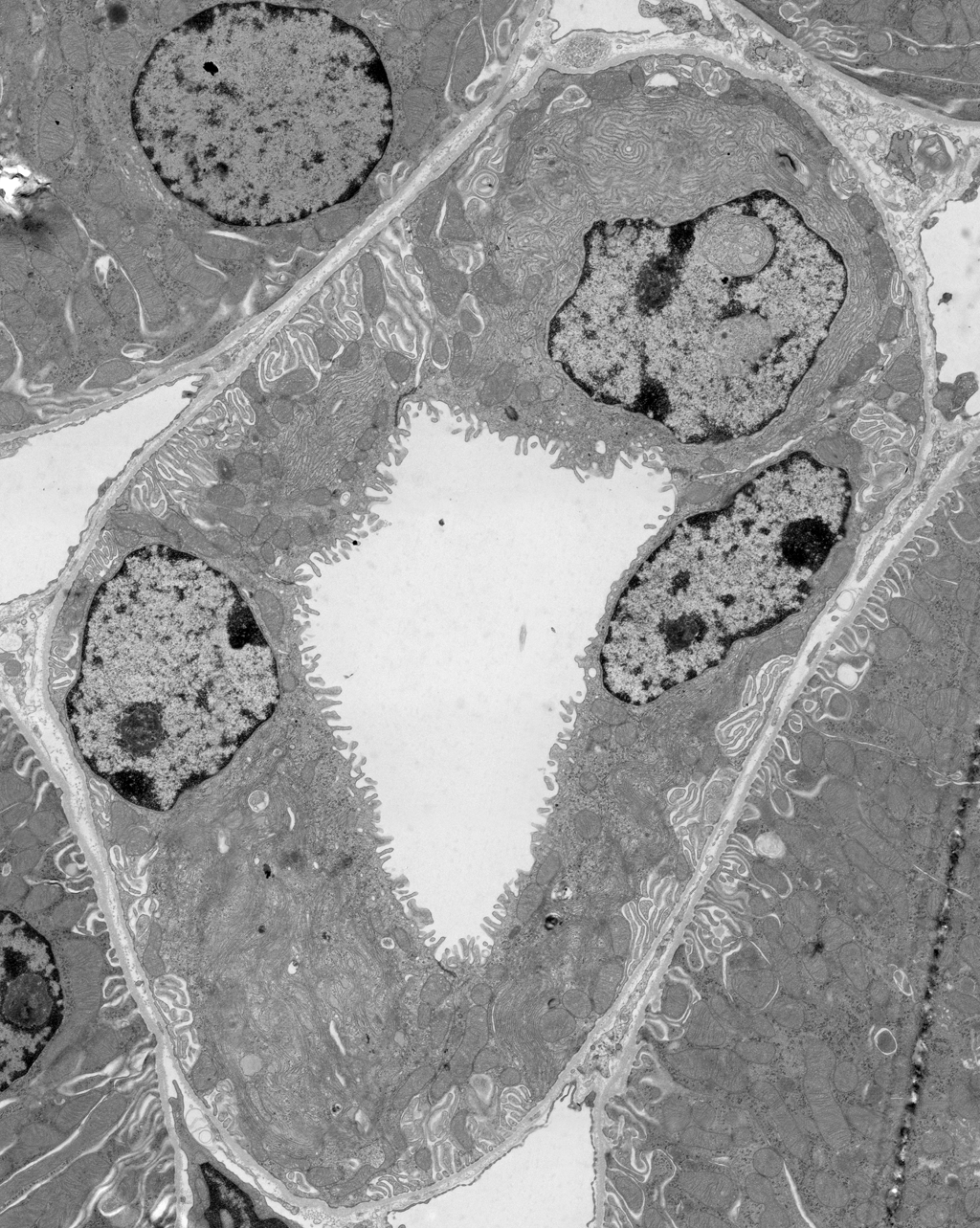

Supplement: Supplementary file 3 — Source Data for Figure 2 [file EMMM-15-e18242-s007.zip › Figure_2/2F/R186S+_EM.tif]

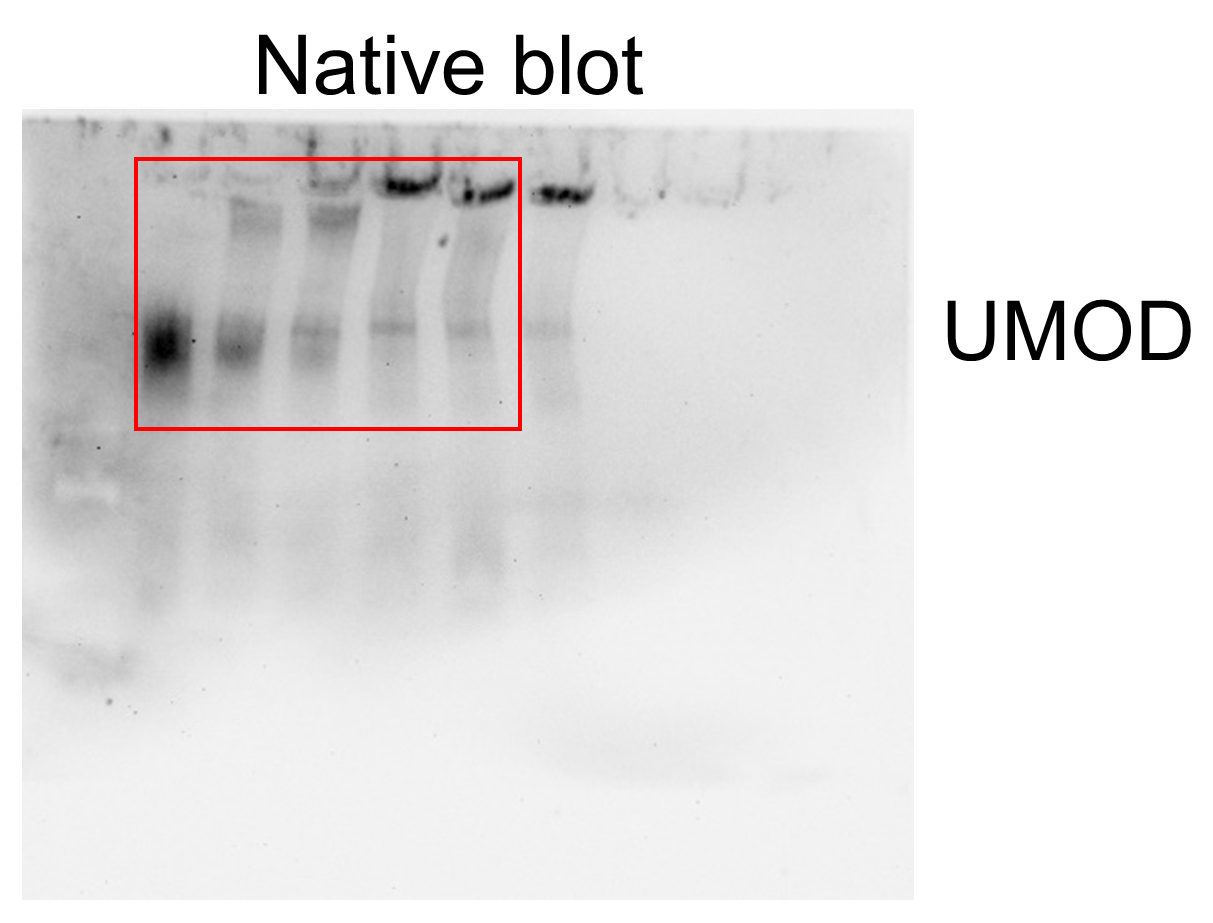

Supplement: Supplementary file 4 — Source Data for Figure 3 [file EMMM-15-e18242-s001.zip › Figure_3/3B/Native_blot.tif]

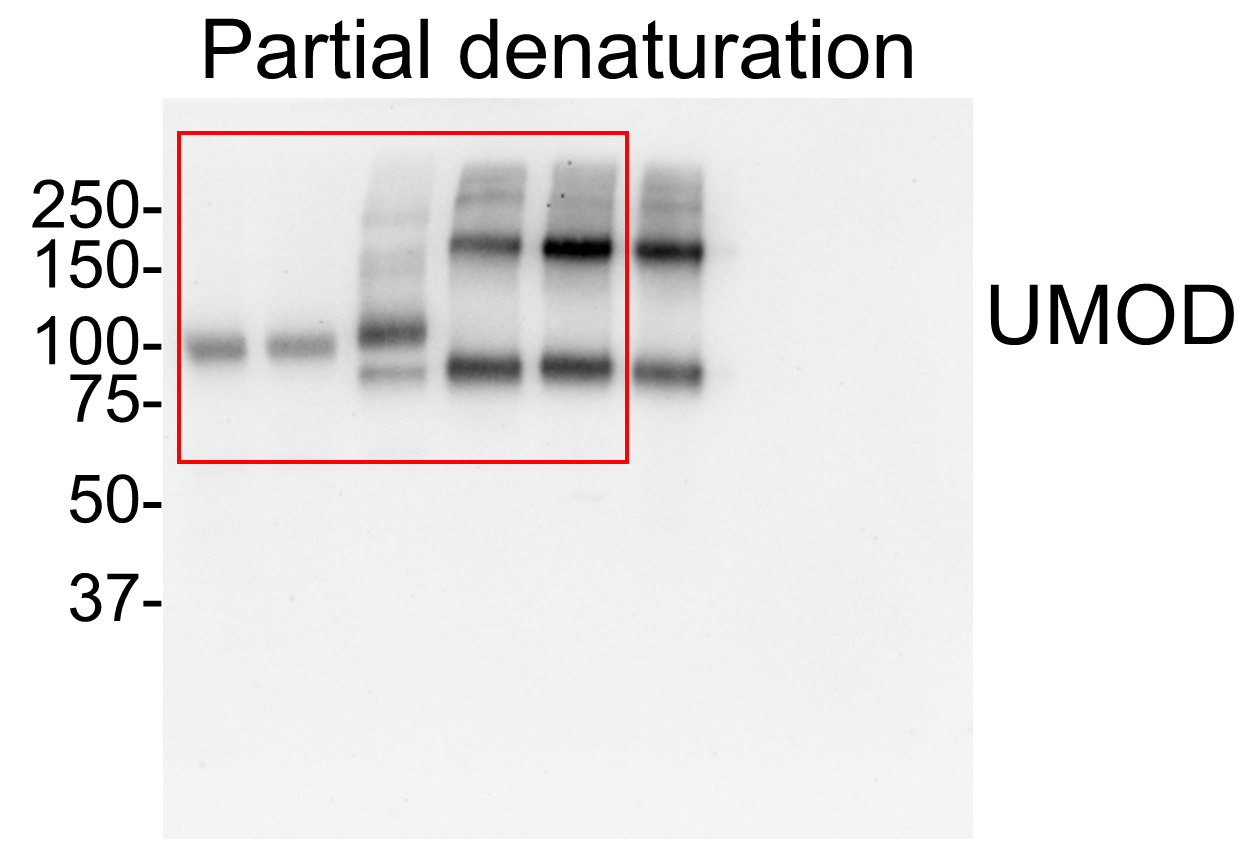

Supplement: Supplementary file 4 — Source Data for Figure 3 [file EMMM-15-e18242-s001.zip › Figure_3/3B/Partial_Denaturation.tif]

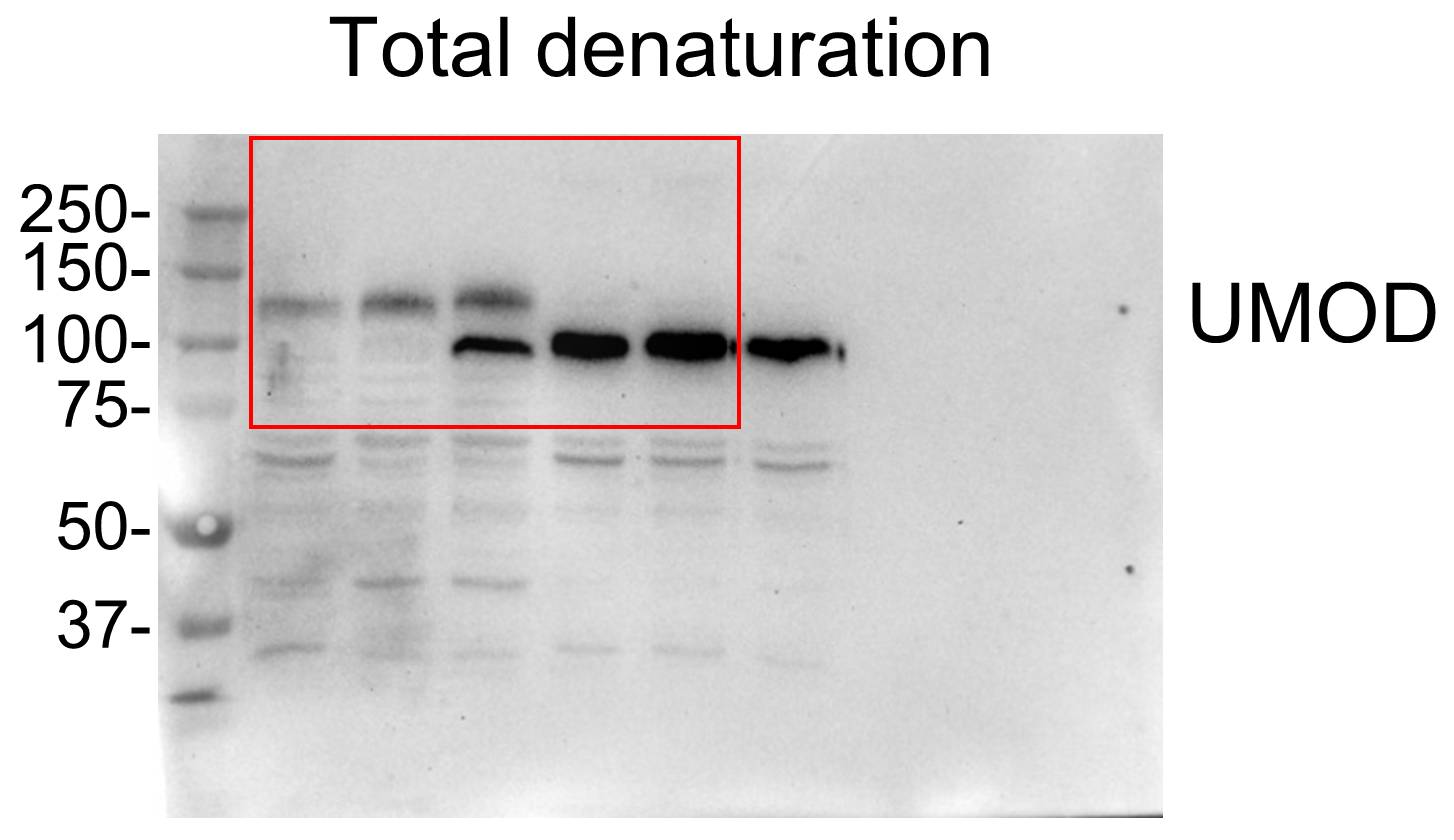

Supplement: Supplementary file 4 — Source Data for Figure 3 [file EMMM-15-e18242-s001.zip › Figure_3/3B/Total_Denaturation.tif]

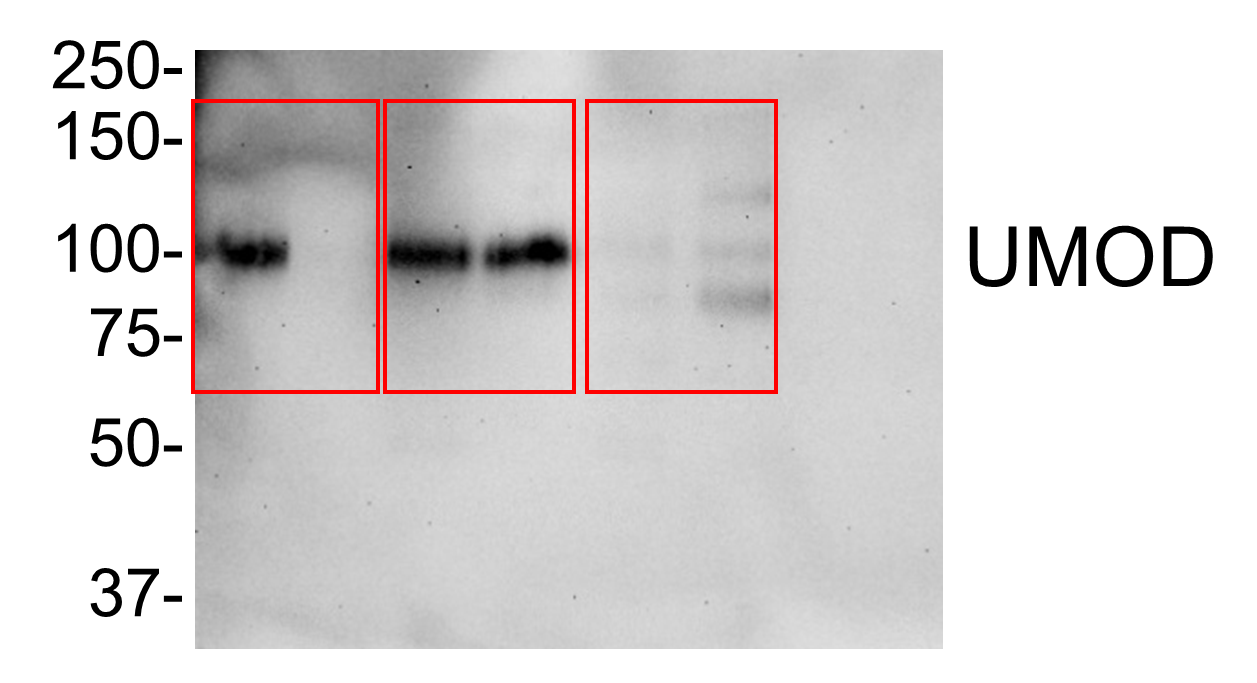

Supplement: Supplementary file 4 — Source Data for Figure 3 [file EMMM-15-e18242-s001.zip › Figure_3/3D/UMOD_C171Y_Solubility.tif]

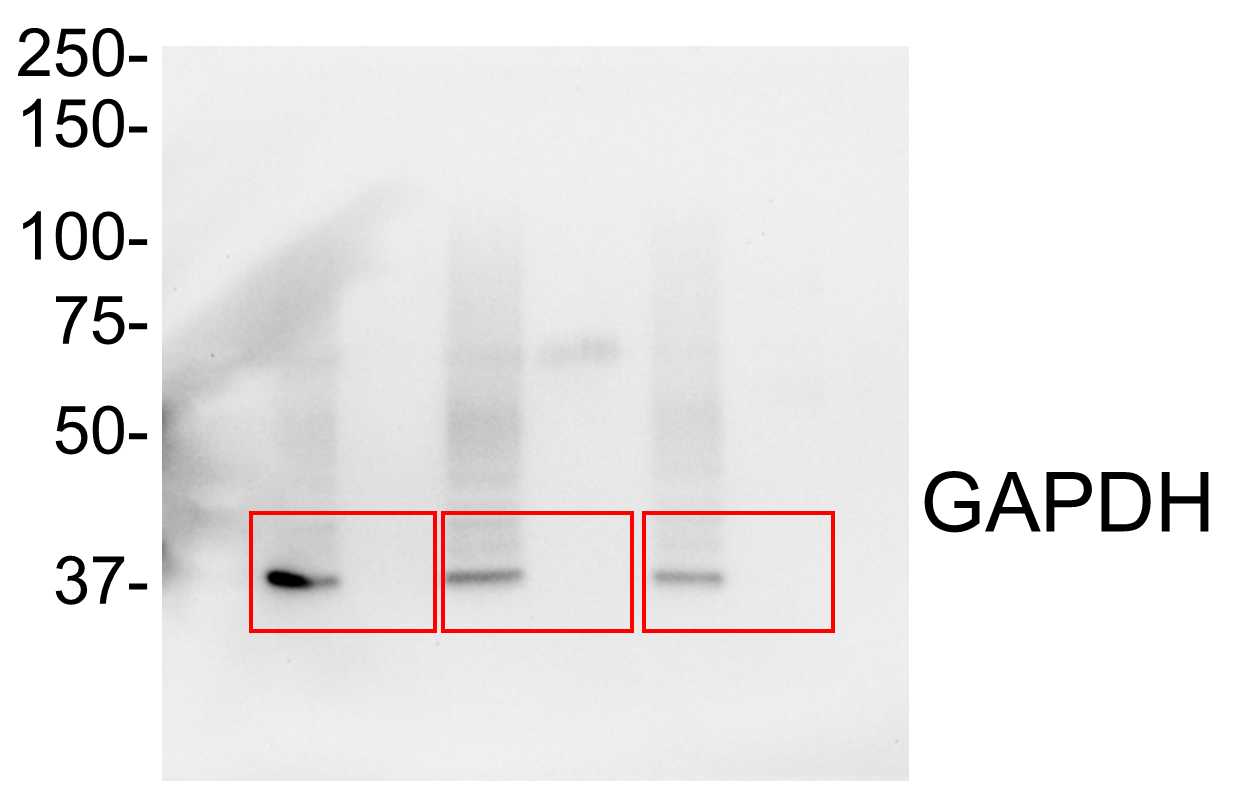

Supplement: Supplementary file 4 — Source Data for Figure 3 [file EMMM-15-e18242-s001.zip › Figure_3/3D/UMOD_C171Y_Solubility_GAPDH.tif]

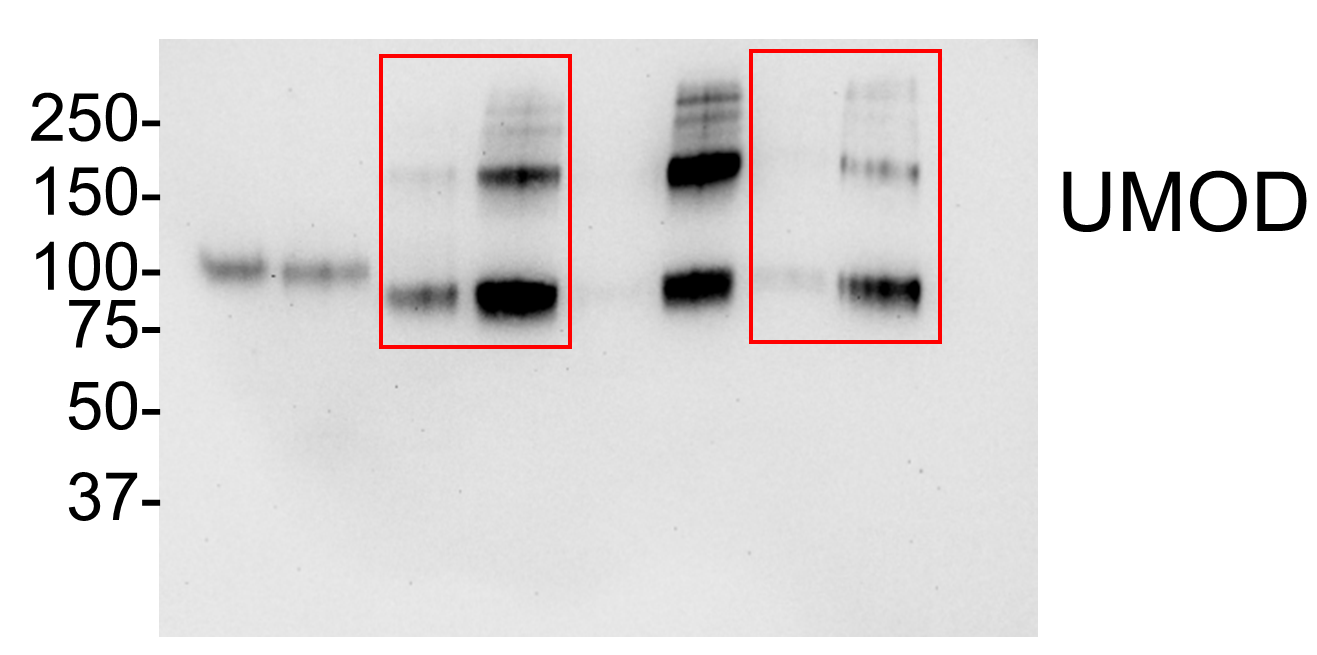

Supplement: Supplementary file 4 — Source Data for Figure 3 [file EMMM-15-e18242-s001.zip › Figure_3/3D/UMOD_R186S_Solubility.tif]

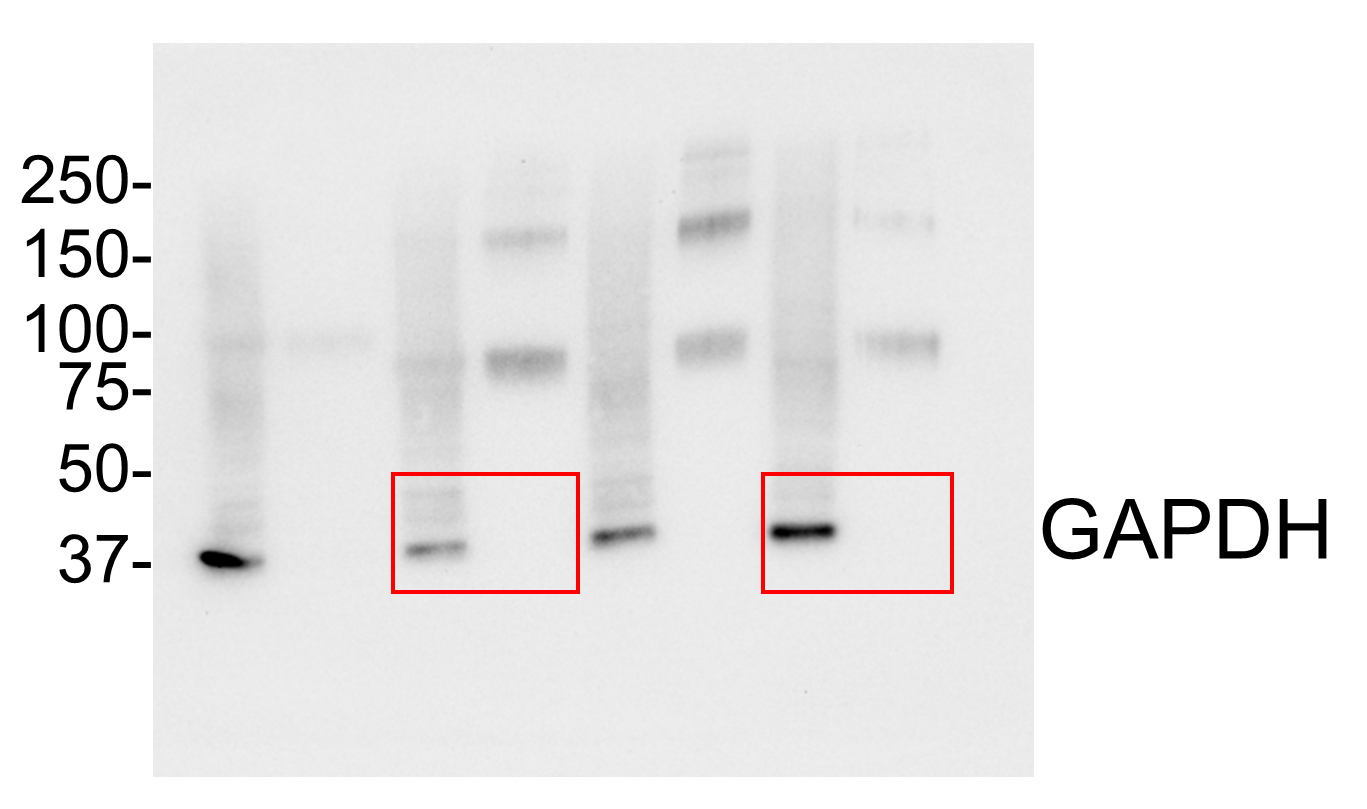

Supplement: Supplementary file 4 — Source Data for Figure 3 [file EMMM-15-e18242-s001.zip › Figure_3/3D/UMOD_R186S_Solubility_GAPDH.tif]

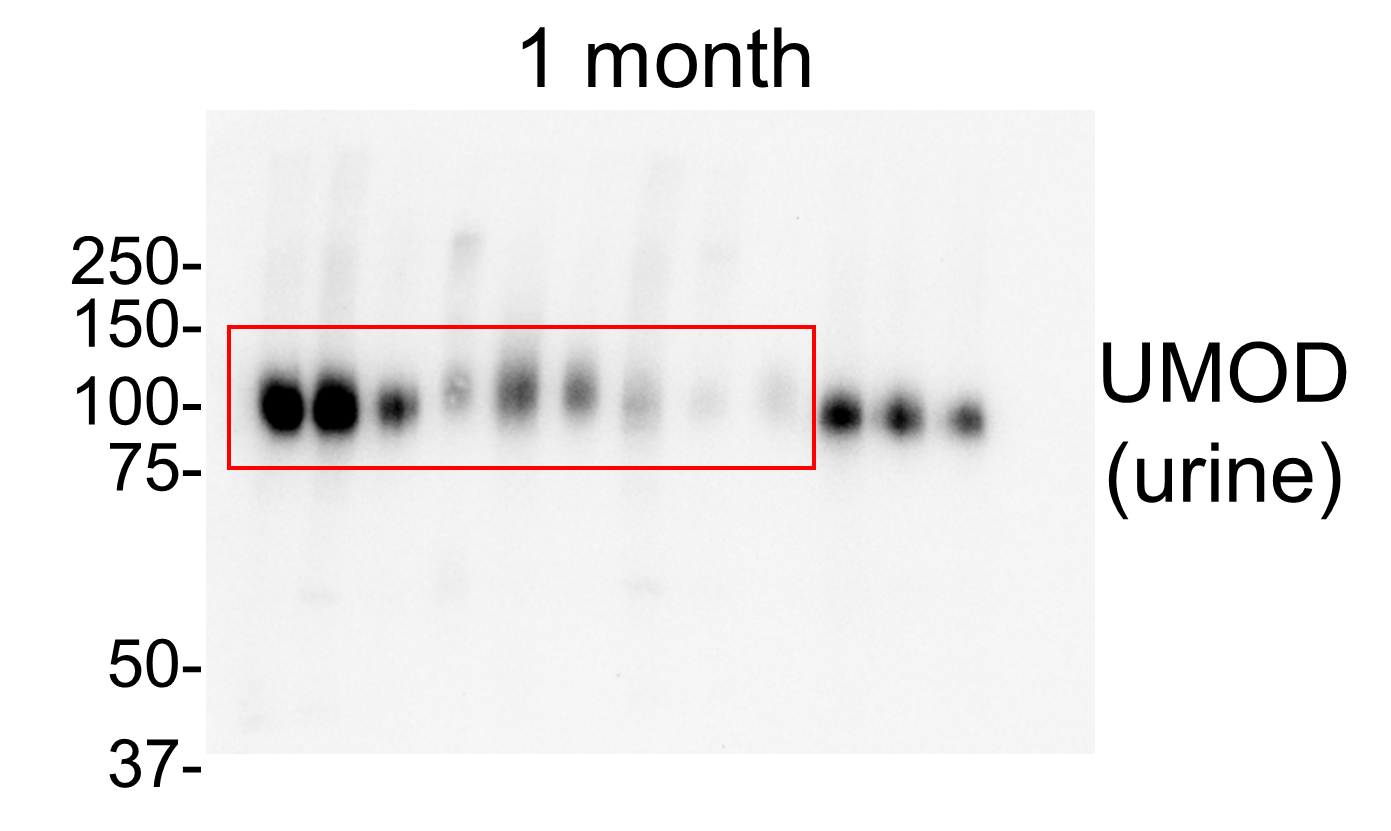

Supplement: Supplementary file 5 — Source Data for Figure 4 [file EMMM-15-e18242-s009.zip › Figure_4/4C/WB_urine_UMOD_1_mo.tif]

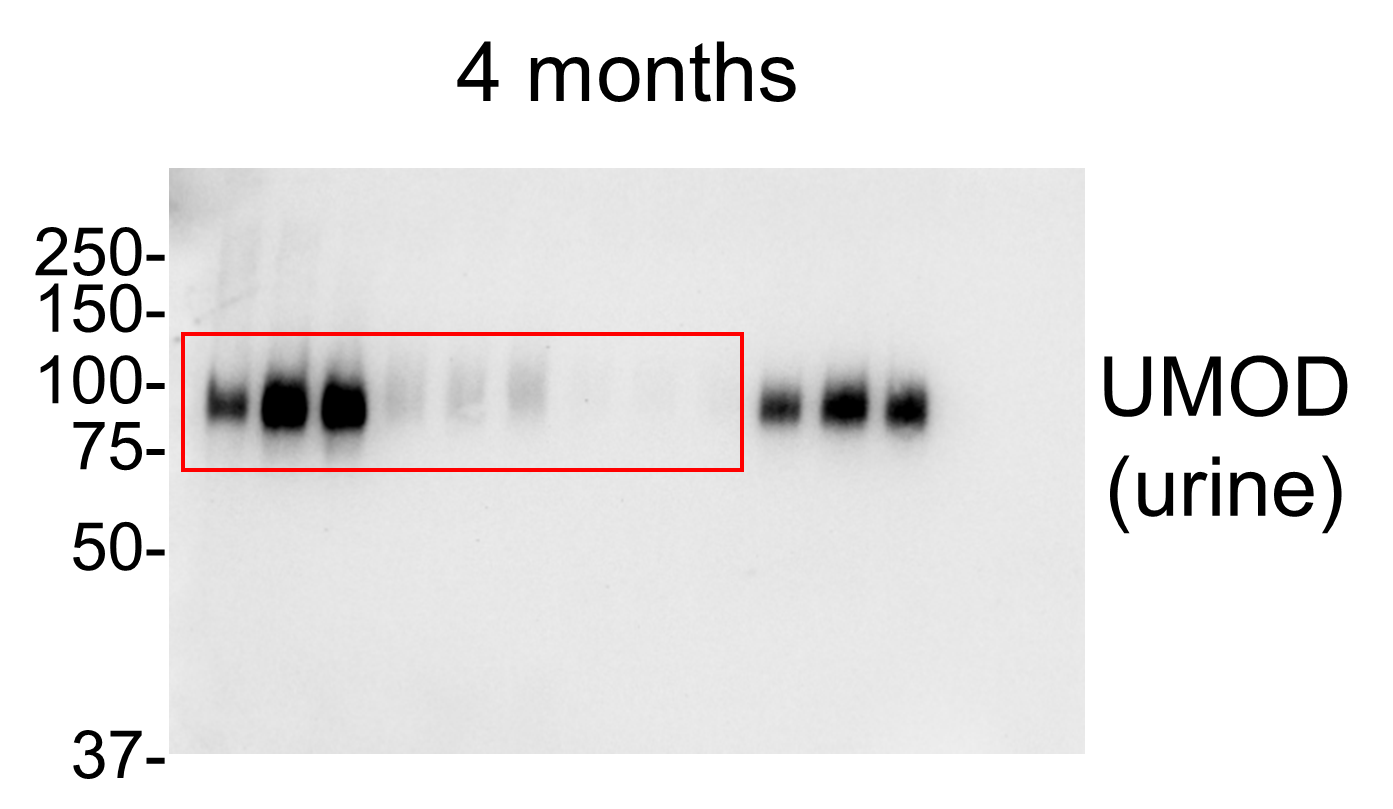

Supplement: Supplementary file 5 — Source Data for Figure 4 [file EMMM-15-e18242-s009.zip › Figure_4/4C/WB_urine_UMOD_4_mo.tif]

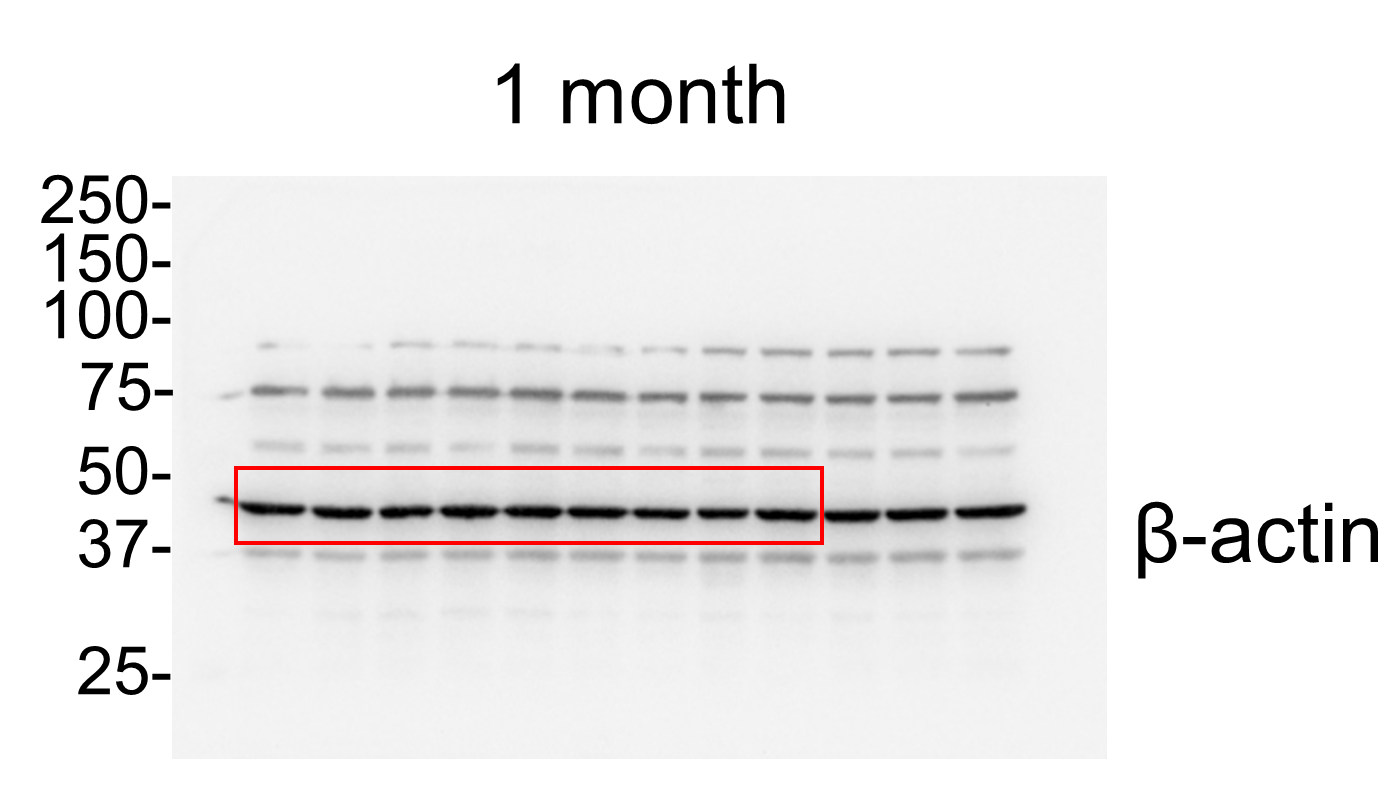

Supplement: Supplementary file 5 — Source Data for Figure 4 [file EMMM-15-e18242-s009.zip › Figure_4/4D/WB_beta-actin_1_mo.tif]

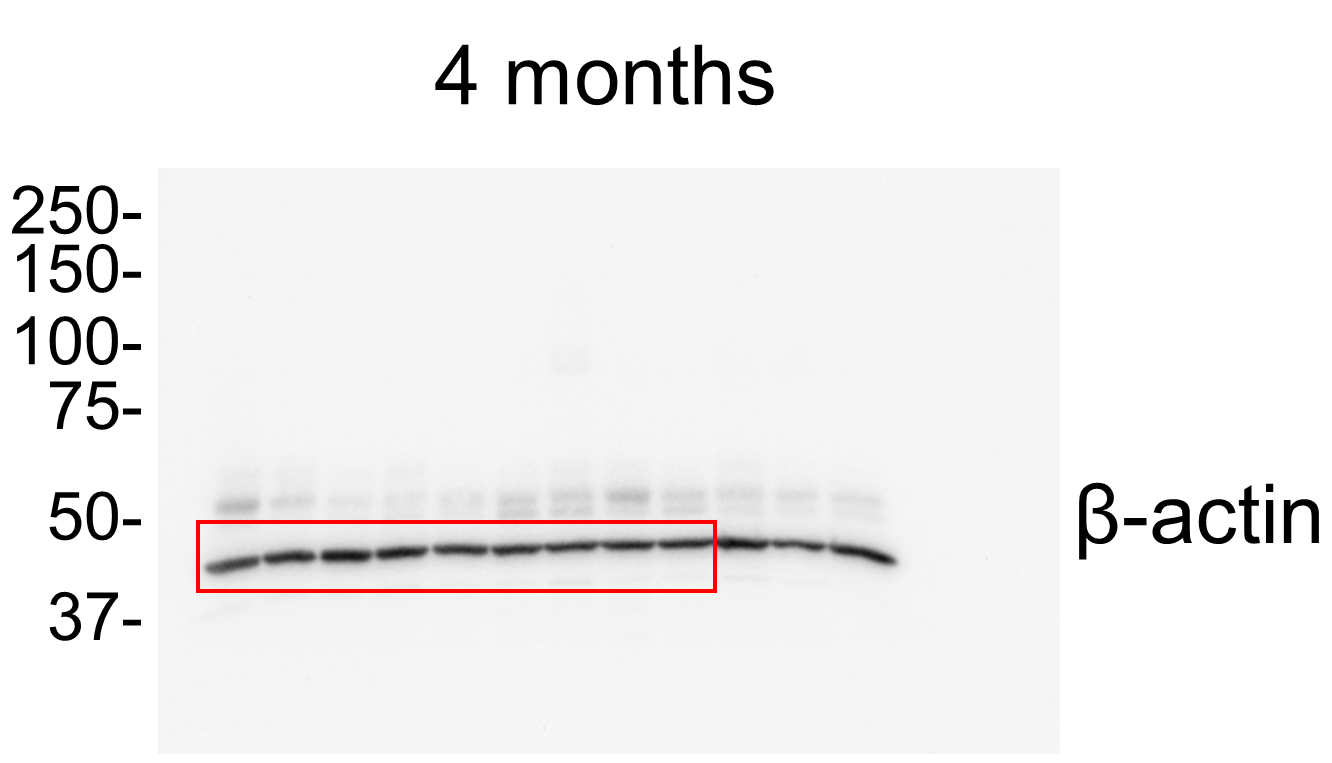

Supplement: Supplementary file 5 — Source Data for Figure 4 [file EMMM-15-e18242-s009.zip › Figure_4/4D/WB_beta-actin_4_mo.tif]

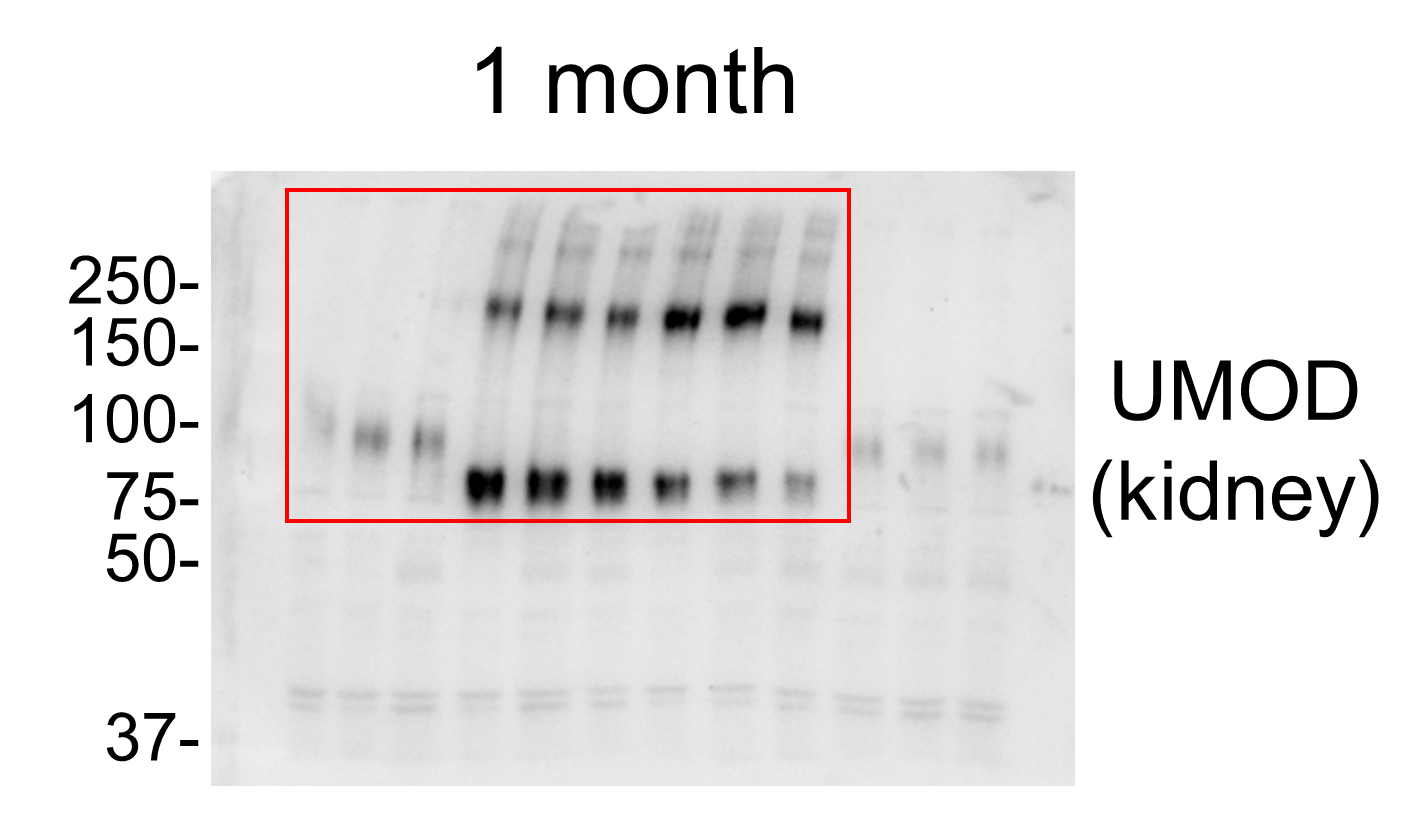

Supplement: Supplementary file 5 — Source Data for Figure 4 [file EMMM-15-e18242-s009.zip › Figure_4/4D/WB_kidney_UMOD_1_mo.tif]

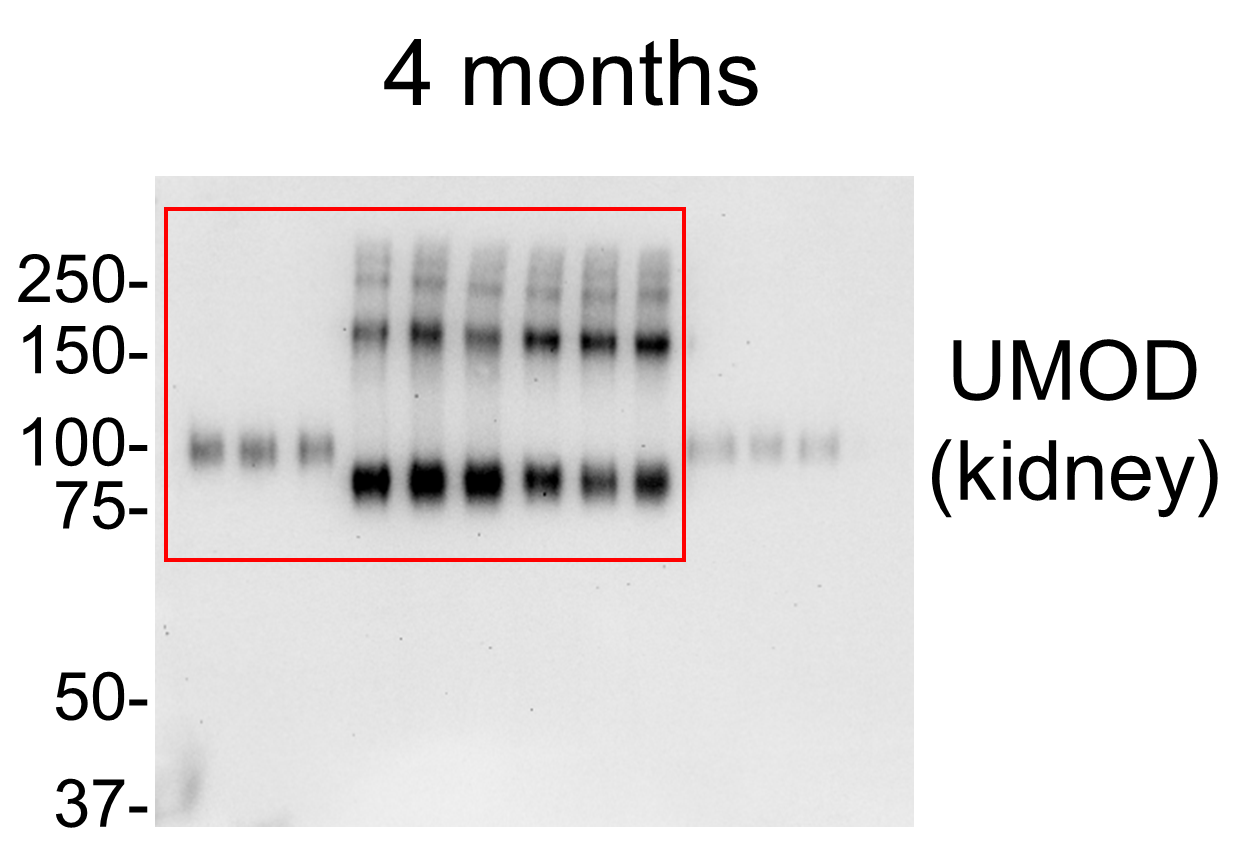

Supplement: Supplementary file 5 — Source Data for Figure 4 [file EMMM-15-e18242-s009.zip › Figure_4/4D/WB_kidney_UMOD_4_mo.tif]

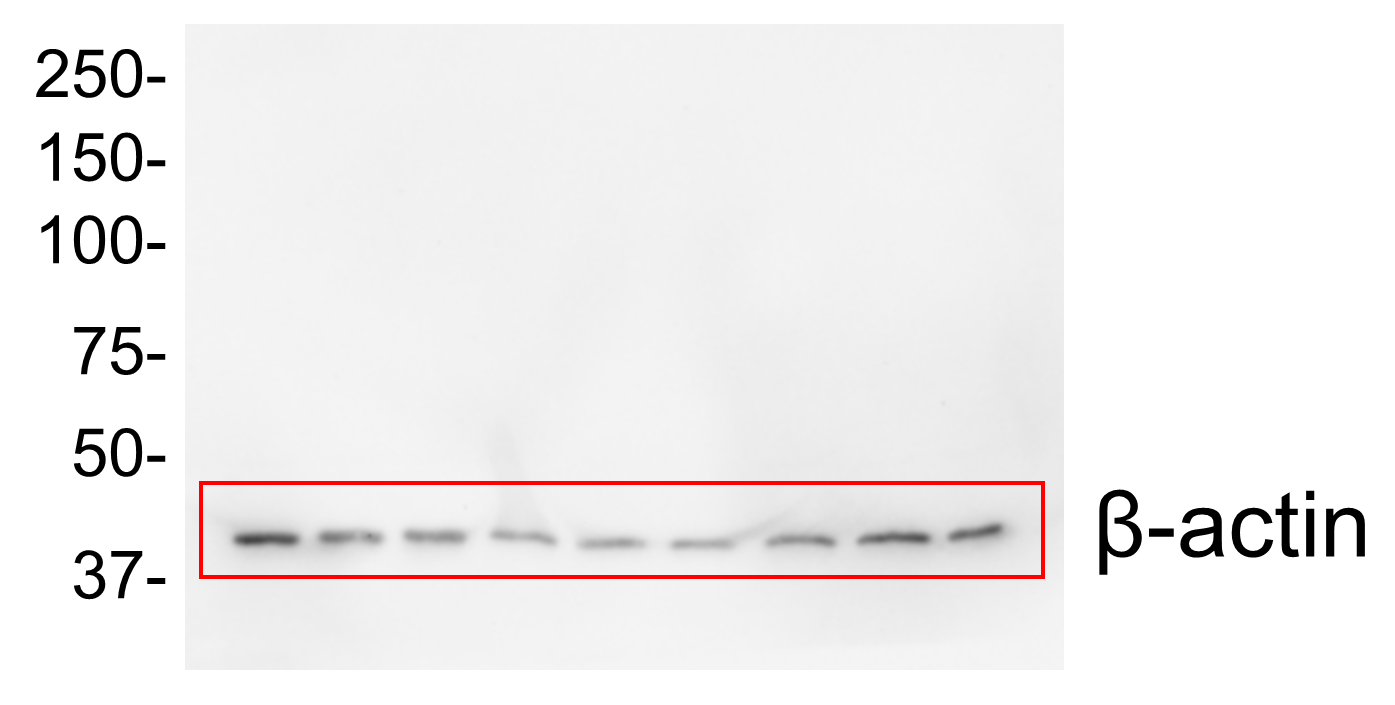

Supplement: Supplementary file 5 — Source Data for Figure 4 [file EMMM-15-e18242-s009.zip › Figure_4/4E/WB_tubules_beta-actin.tif]

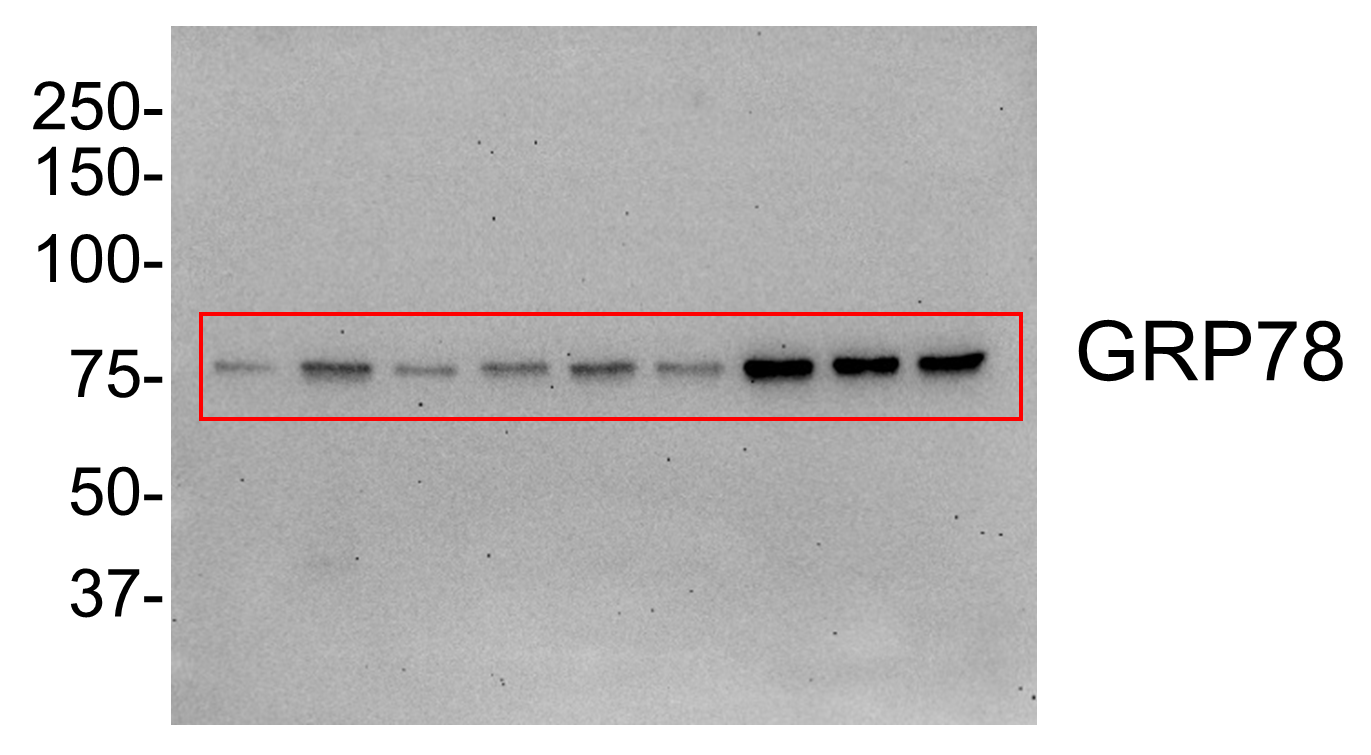

Supplement: Supplementary file 5 — Source Data for Figure 4 [file EMMM-15-e18242-s009.zip › Figure_4/4E/WB_tubules_GRP78.tif]

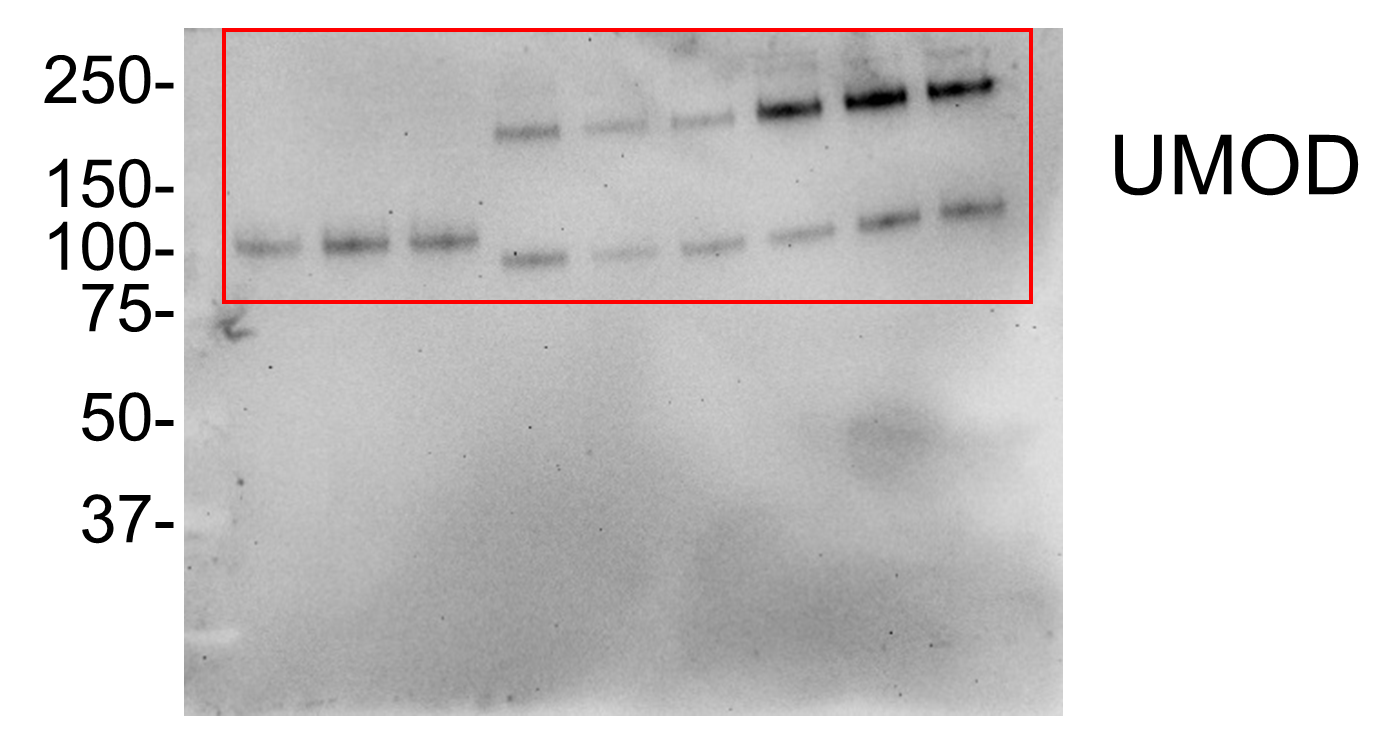

Supplement: Supplementary file 5 — Source Data for Figure 4 [file EMMM-15-e18242-s009.zip › Figure_4/4E/WB_tubules_UMOD.tif]

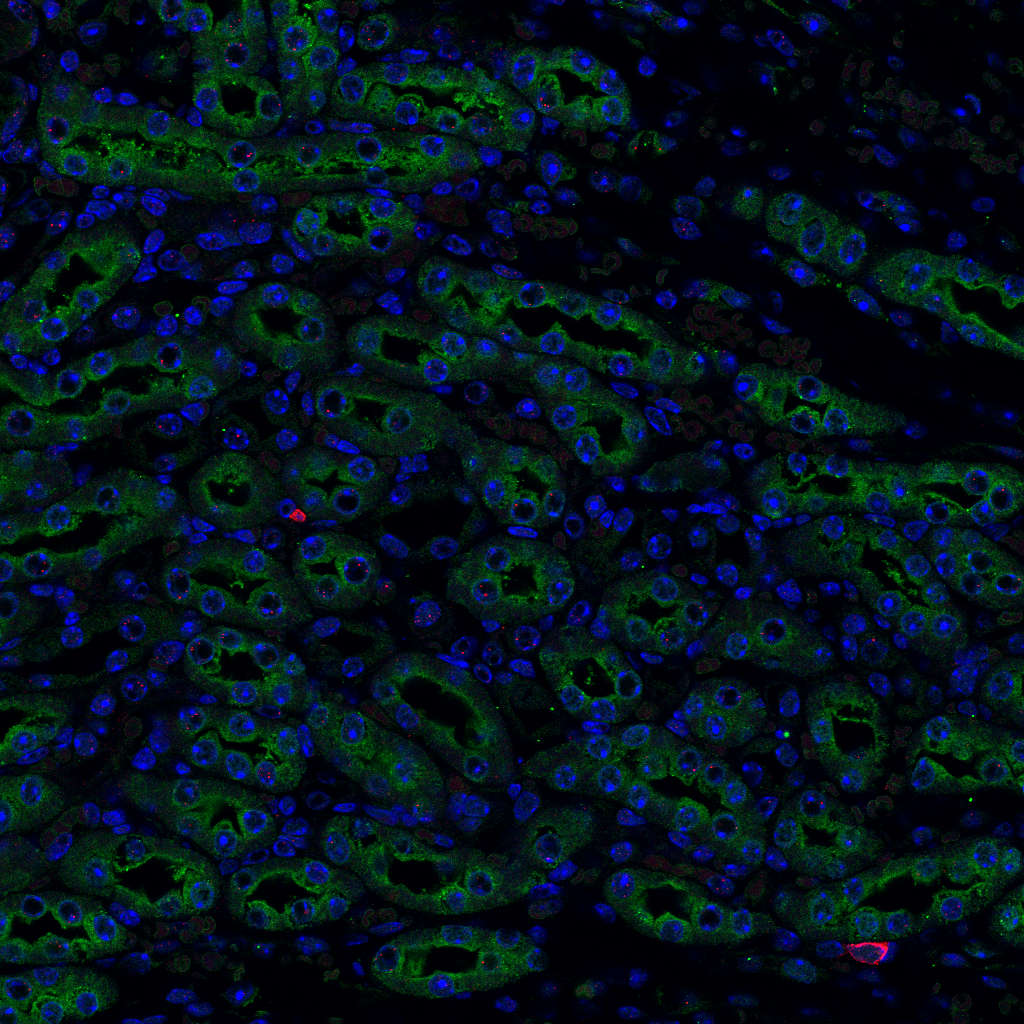

Supplement: Supplementary file 5 — Source Data for Figure 4 [file EMMM-15-e18242-s009.zip › Figure_4/4G/++_-_UMOD,_CD3.tif]

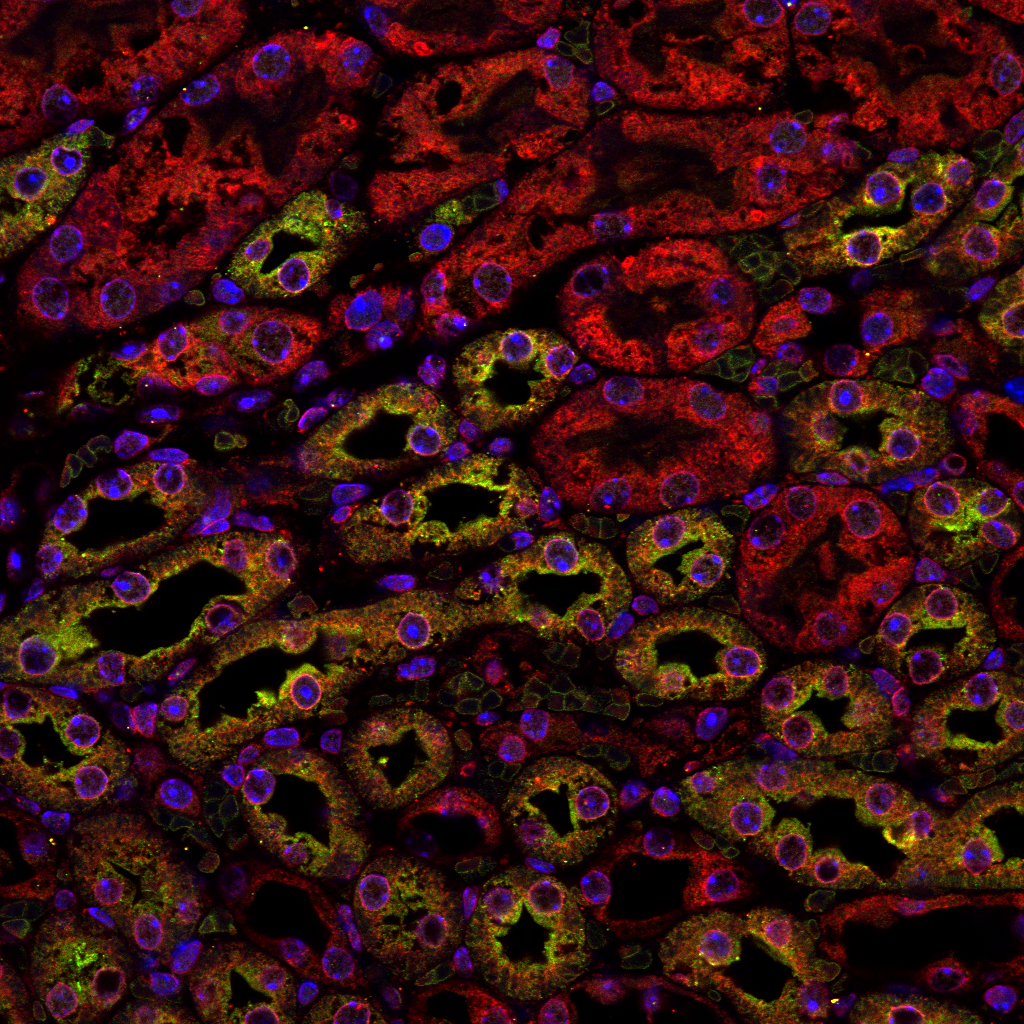

Supplement: Supplementary file 5 — Source Data for Figure 4 [file EMMM-15-e18242-s009.zip › Figure_4/4G/++_-_UMOD,_CNX.tif]

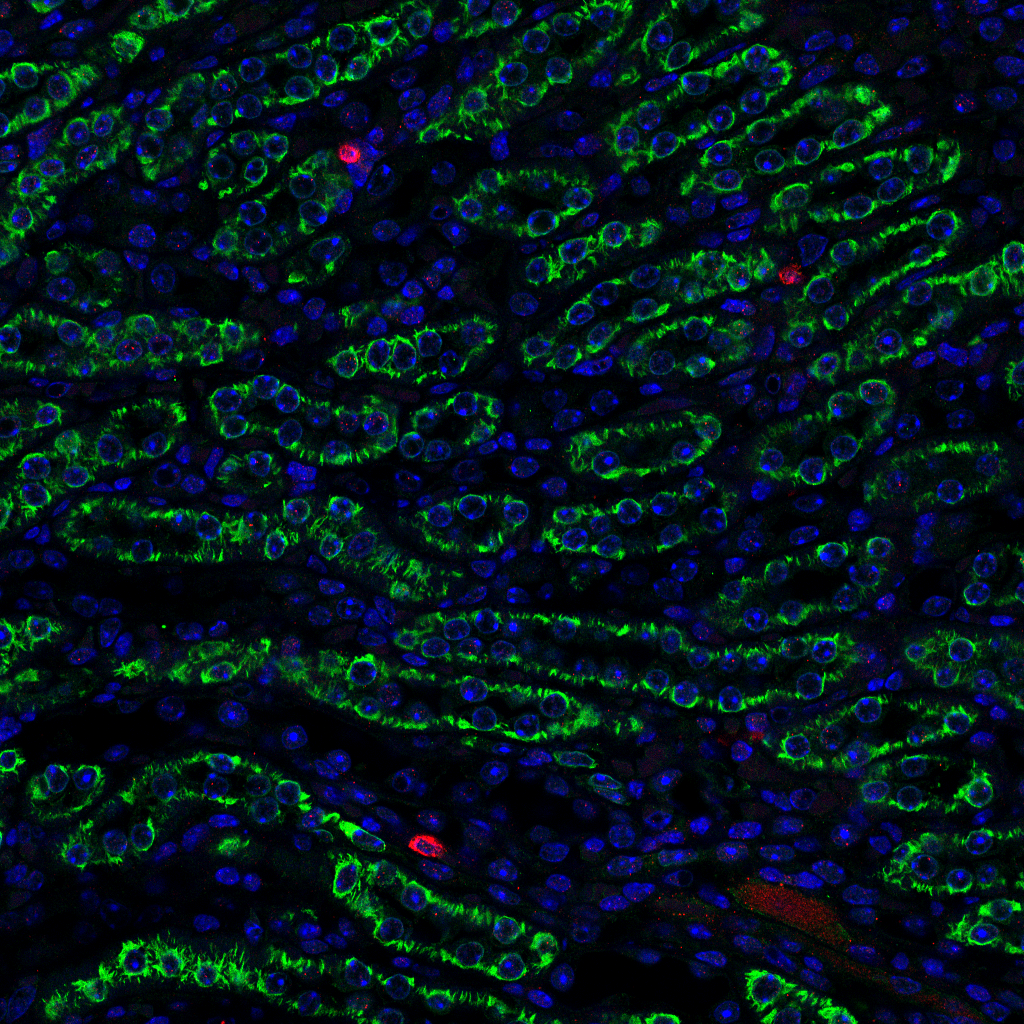

Supplement: Supplementary file 5 — Source Data for Figure 4 [file EMMM-15-e18242-s009.zip › Figure_4/4G/R186S+_-_UMOD,_CD3.tif]

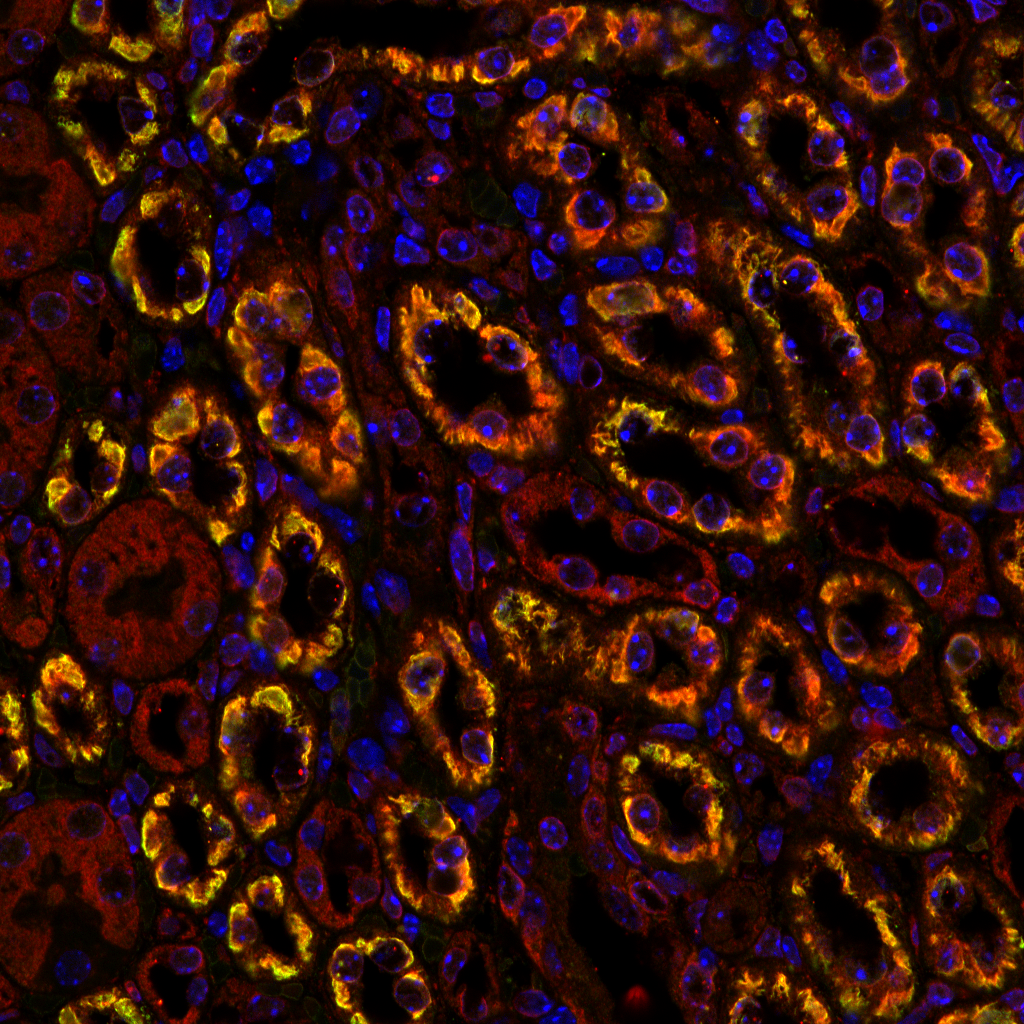

Supplement: Supplementary file 5 — Source Data for Figure 4 [file EMMM-15-e18242-s009.zip › Figure_4/4G/R186S+_-_UMOD,_CNX.tif]

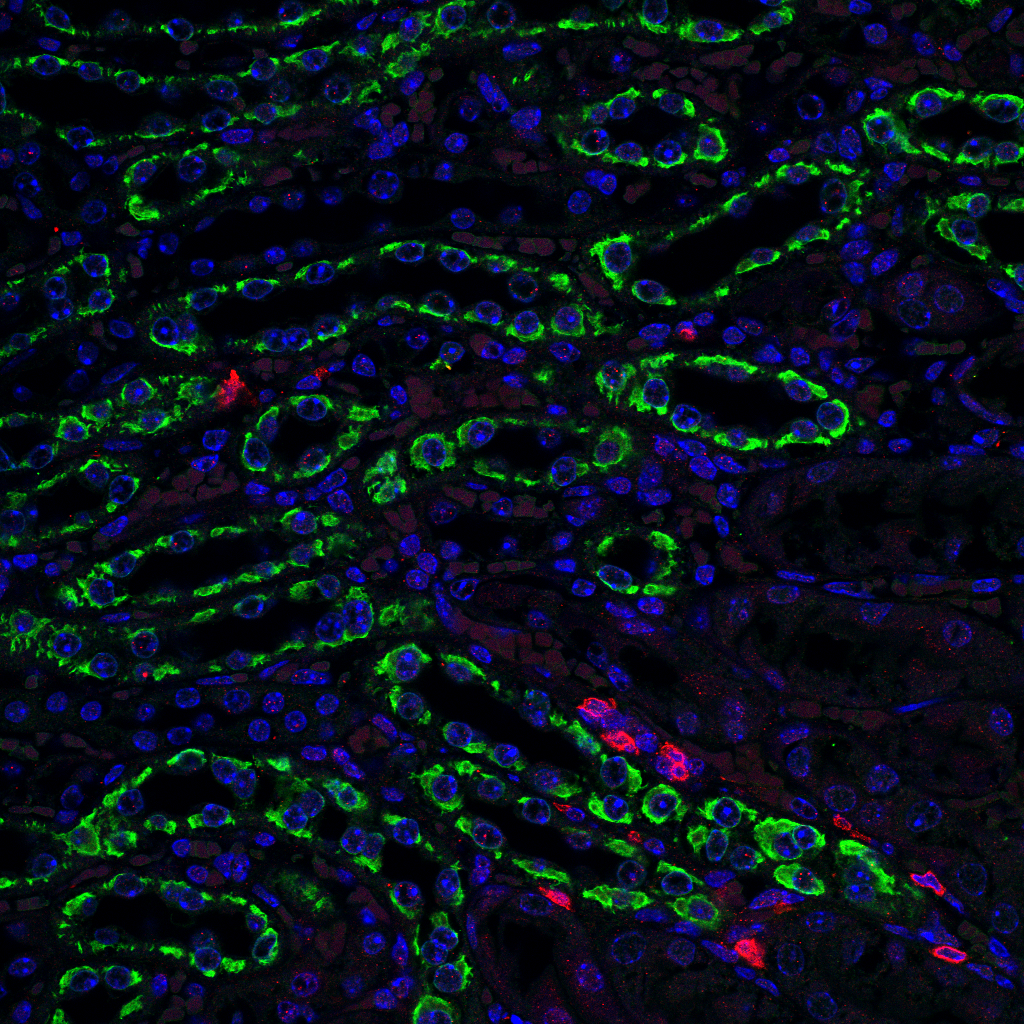

Supplement: Supplementary file 5 — Source Data for Figure 4 [file EMMM-15-e18242-s009.zip › Figure_4/4G/R186S-_-_UMOD,_CD3.tif]

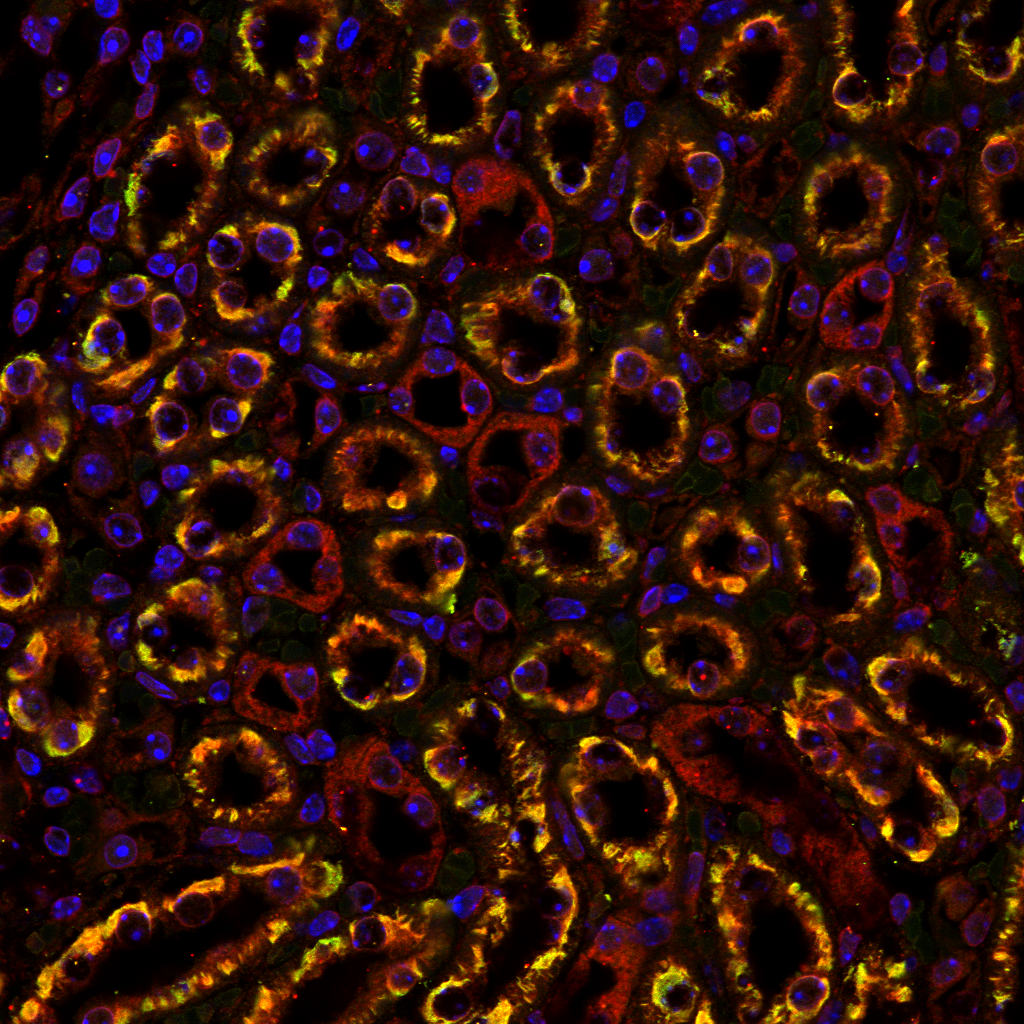

Supplement: Supplementary file 5 — Source Data for Figure 4 [file EMMM-15-e18242-s009.zip › Figure_4/4G/R186S-_-_UMOD,_CNX.tif]

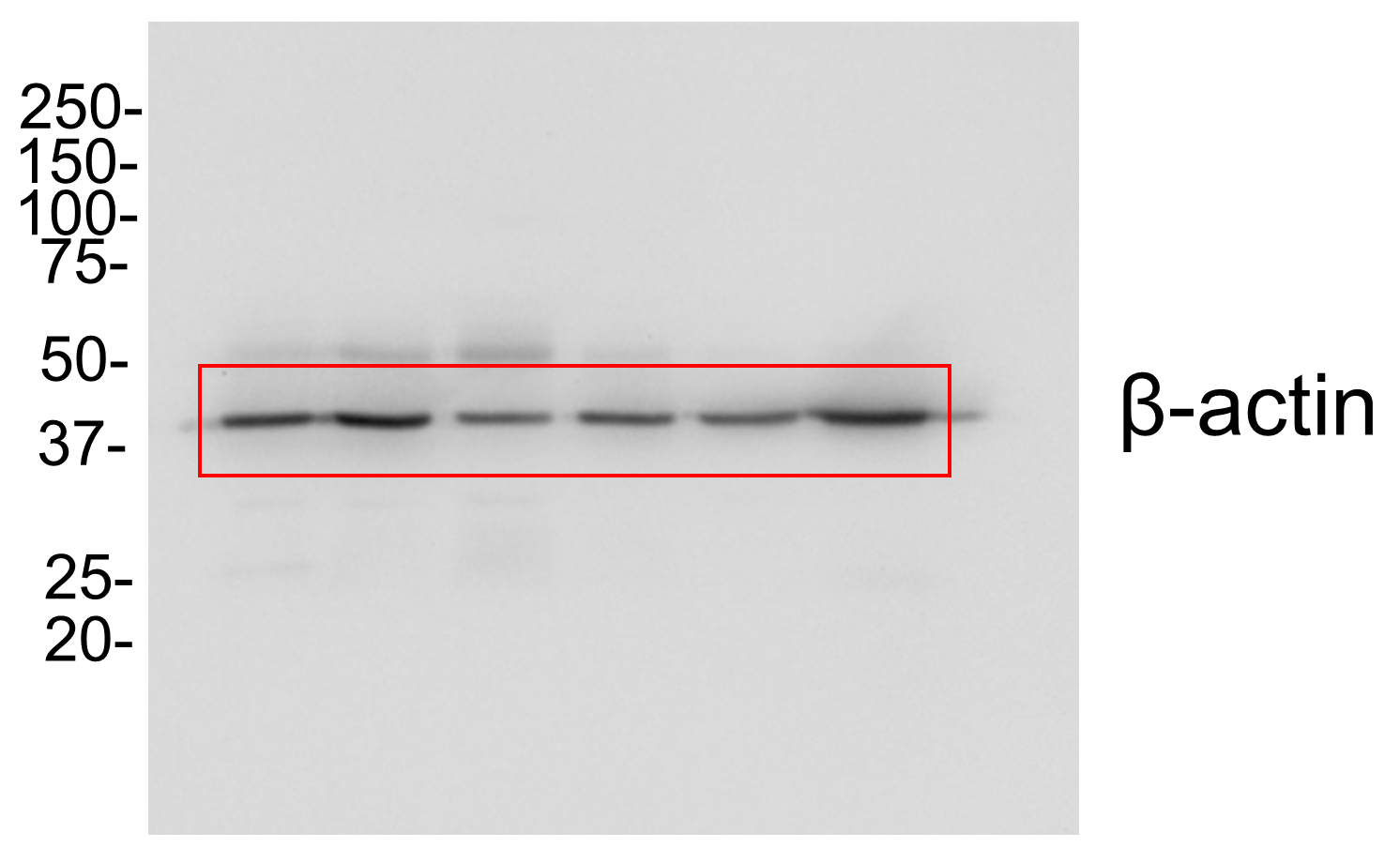

Supplement: Supplementary file 6 — Source Data for Figure 5 [file EMMM-15-e18242-s004.zip › Figure_5/5A/WB_beta-actin.tif]

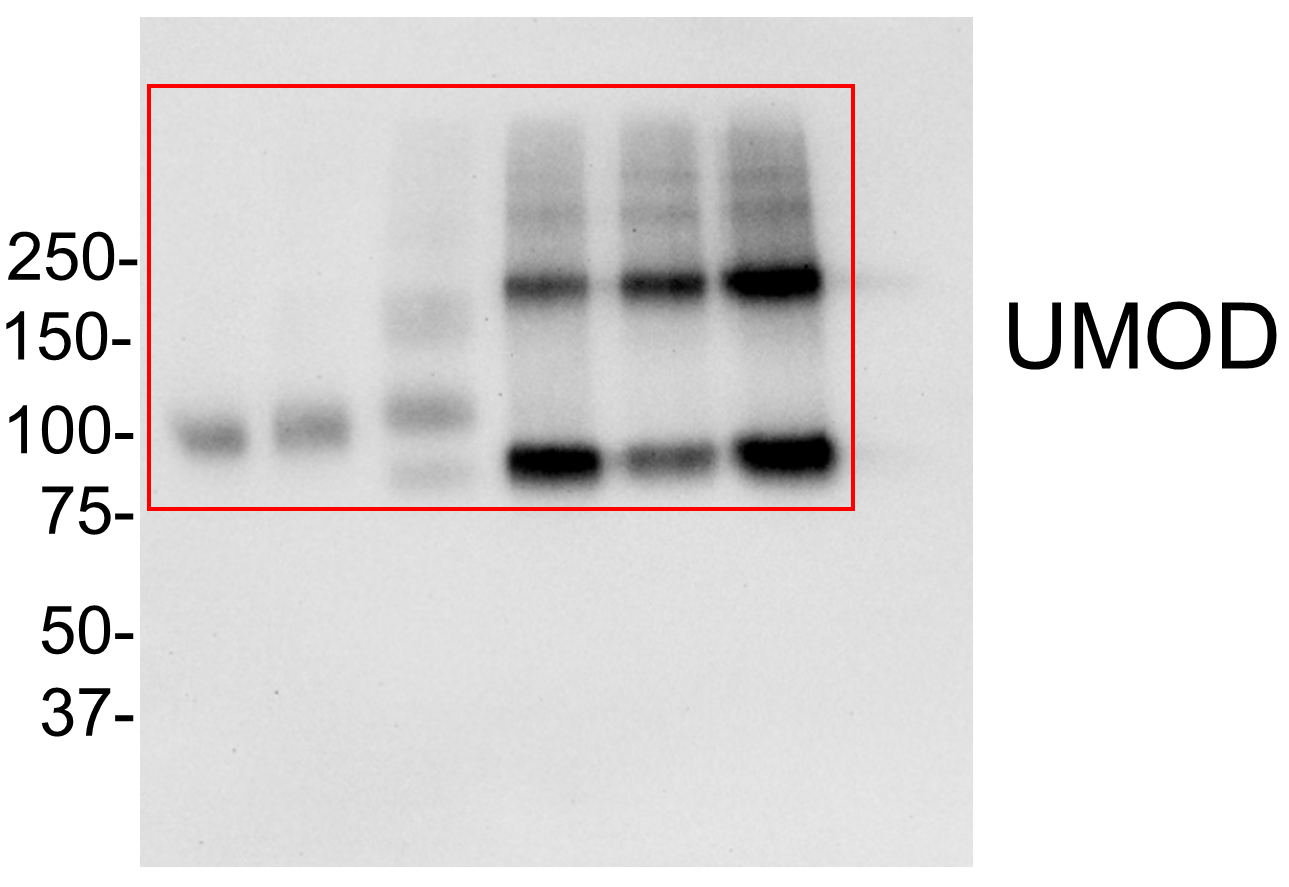

Supplement: Supplementary file 6 — Source Data for Figure 5 [file EMMM-15-e18242-s004.zip › Figure_5/5A/WB_UMOD.tif]

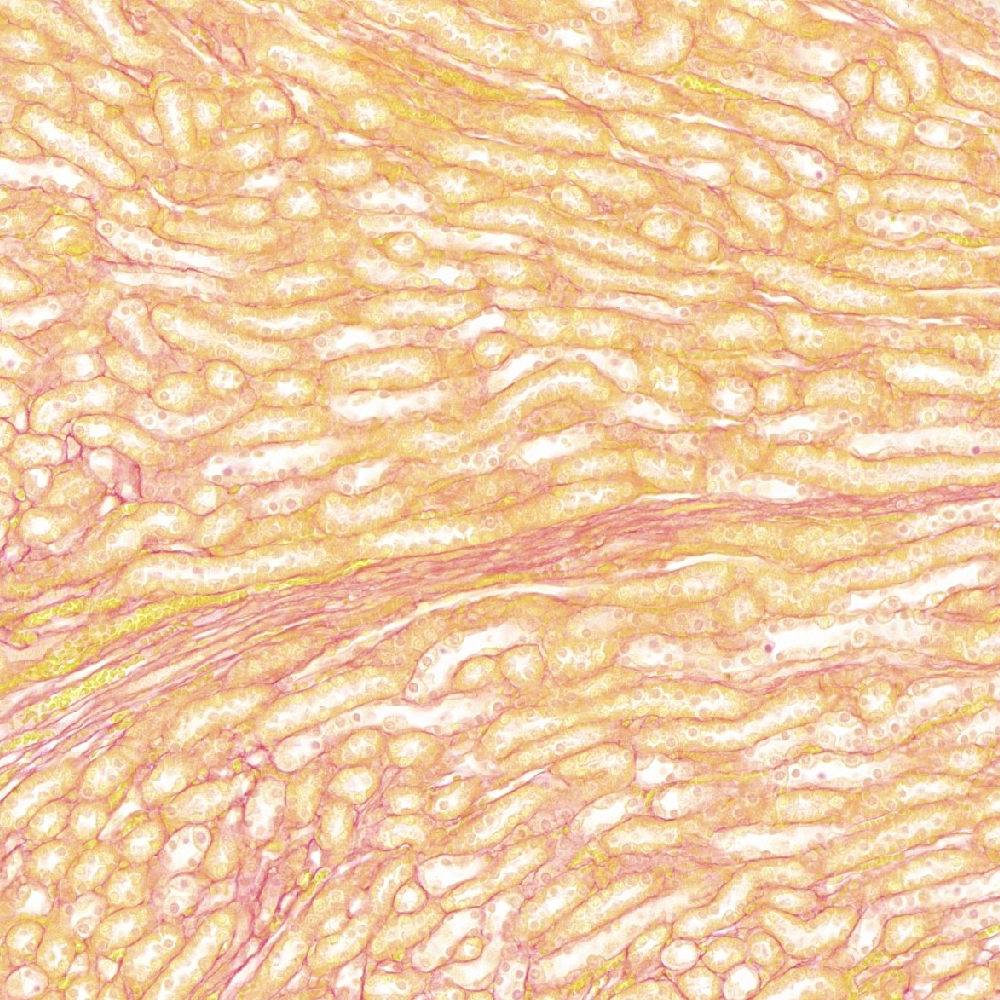

Supplement: Supplementary file 6 — Source Data for Figure 5 [file EMMM-15-e18242-s004.zip › Figure_5/5B/++_-_Picrosirius_red.tif]

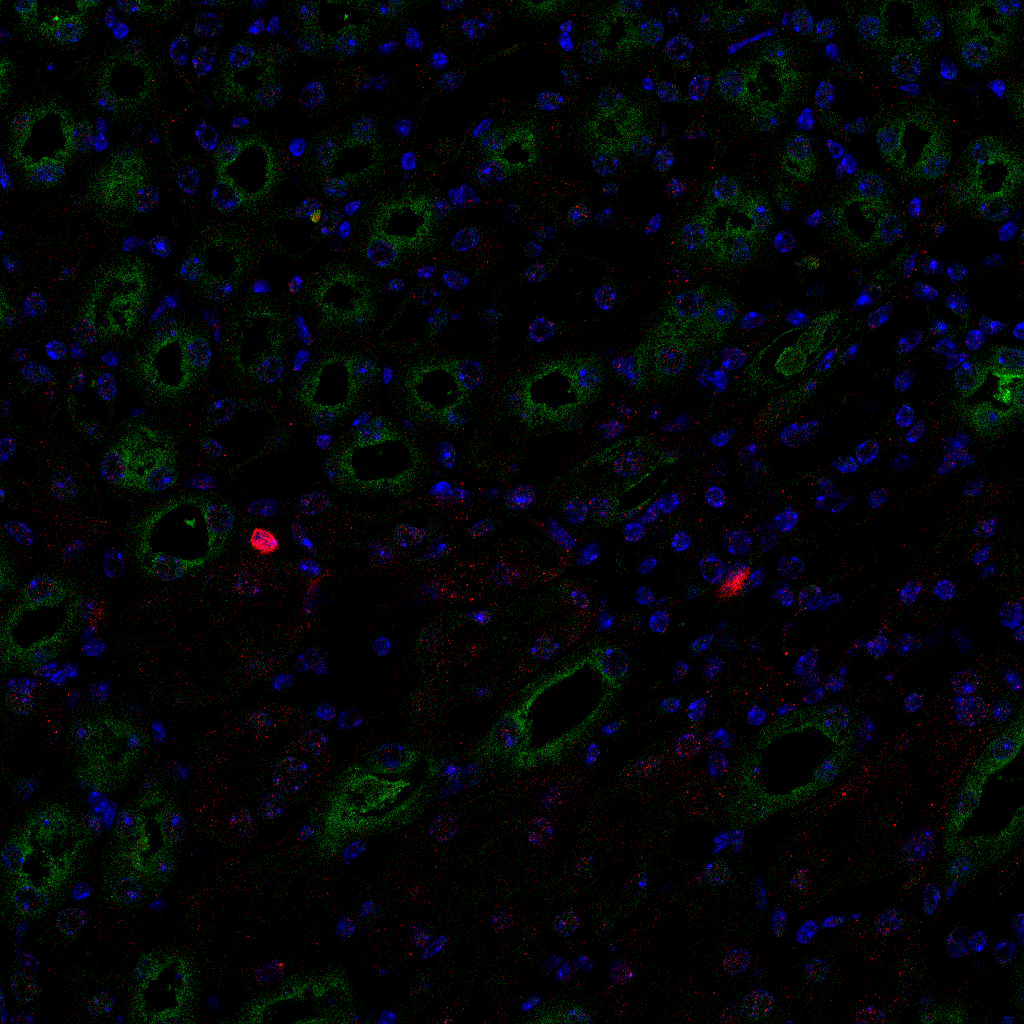

Supplement: Supplementary file 6 — Source Data for Figure 5 [file EMMM-15-e18242-s004.zip › Figure_5/5B/++_-_UMOD,_CD3.tif]

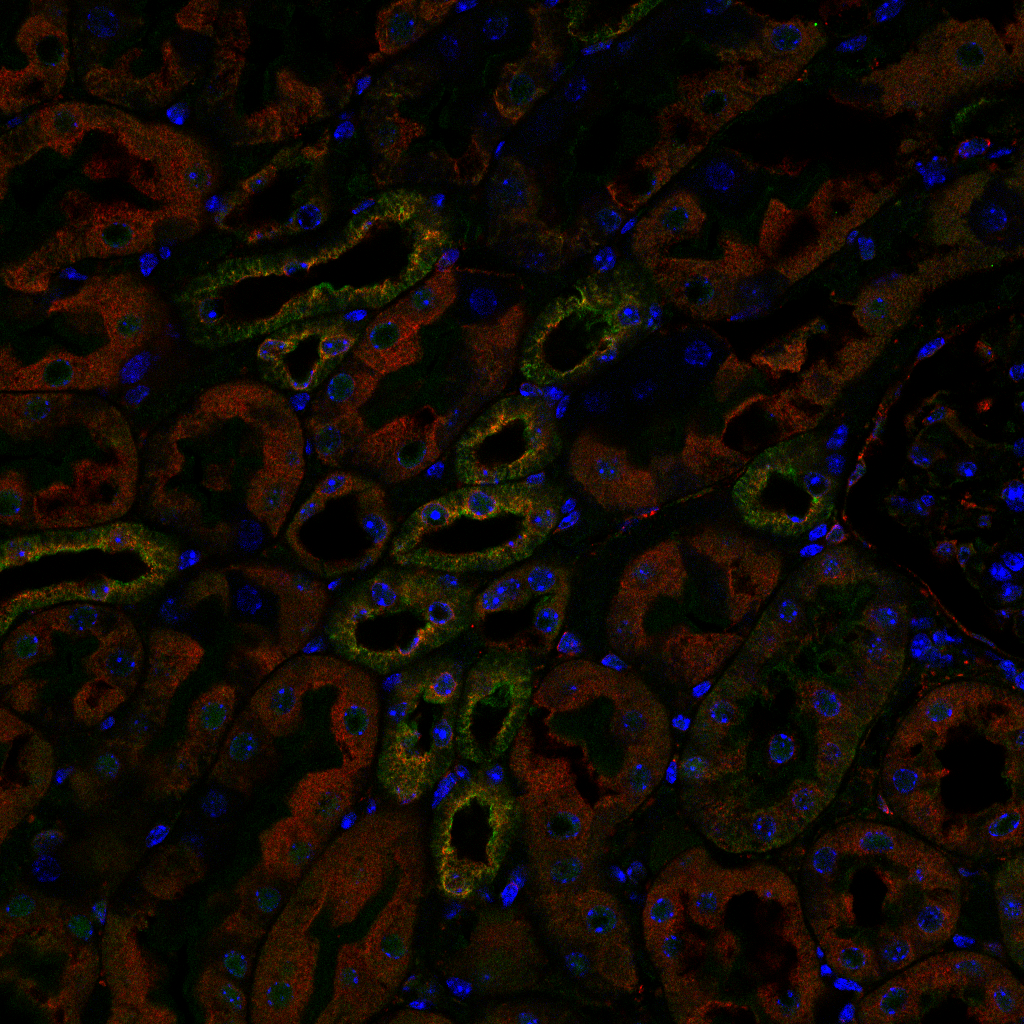

Supplement: Supplementary file 6 — Source Data for Figure 5 [file EMMM-15-e18242-s004.zip › Figure_5/5B/++_-_UMOD,_GRP78.tif]

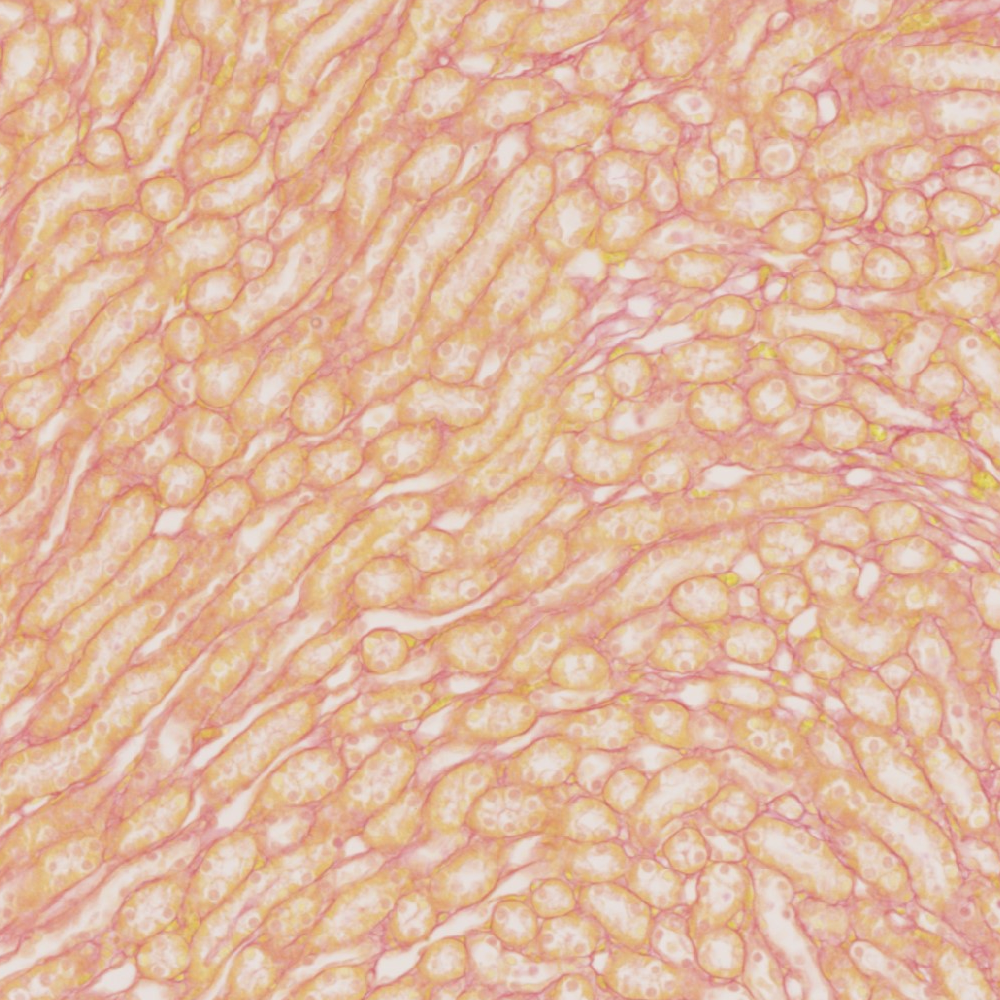

Supplement: Supplementary file 6 — Source Data for Figure 5 [file EMMM-15-e18242-s004.zip › Figure_5/5B/C171Y+_-_Picrosirius_red.tif]

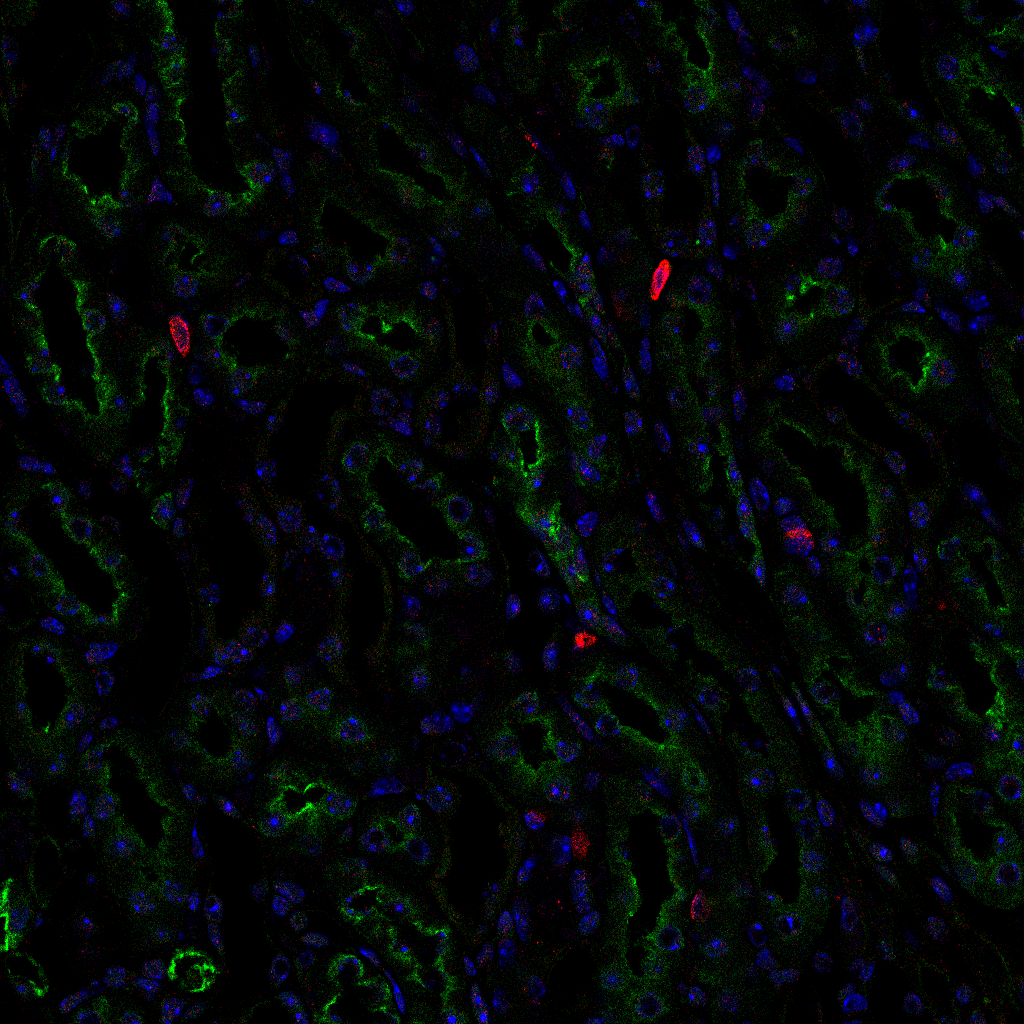

Supplement: Supplementary file 6 — Source Data for Figure 5 [file EMMM-15-e18242-s004.zip › Figure_5/5B/C171Y+_-_UMOD,_CD3.tif]

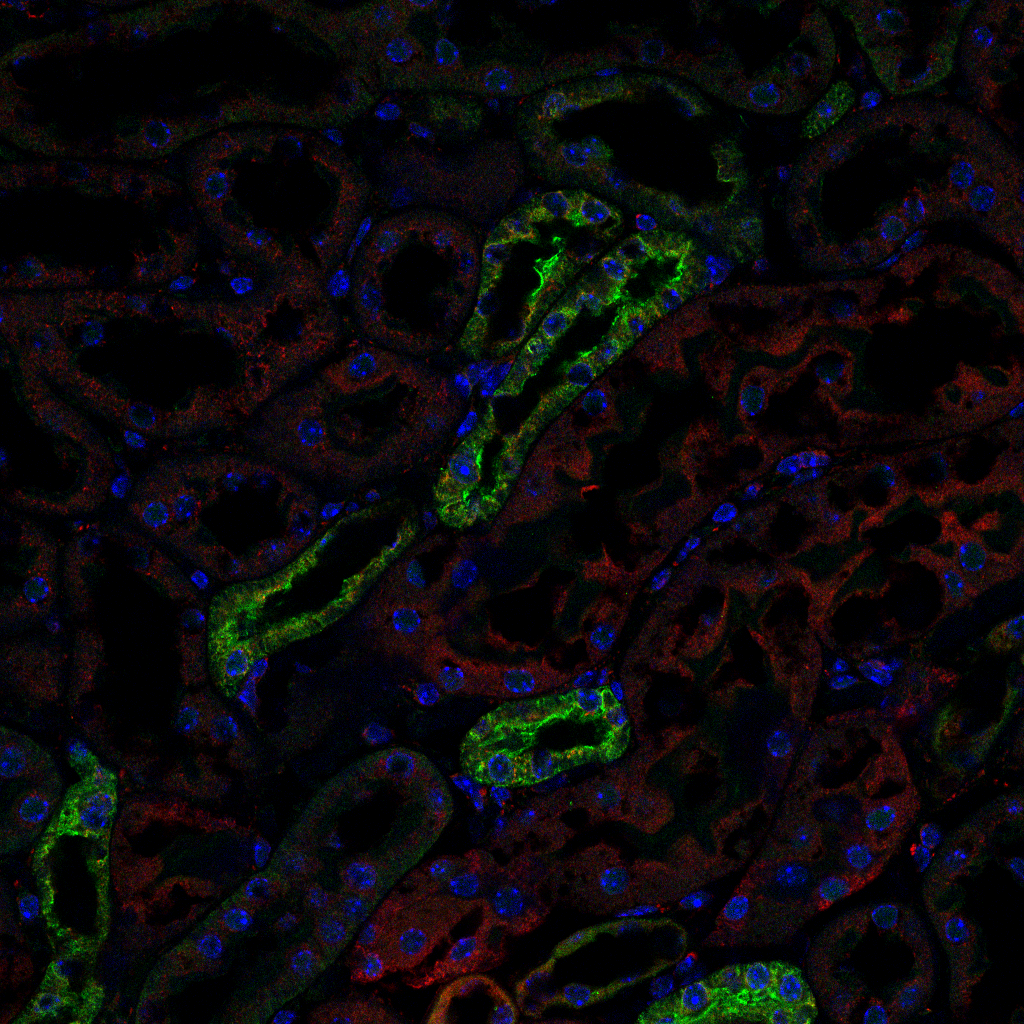

Supplement: Supplementary file 6 — Source Data for Figure 5 [file EMMM-15-e18242-s004.zip › Figure_5/5B/C171Y+_-_UMOD,_GRP78.tif]

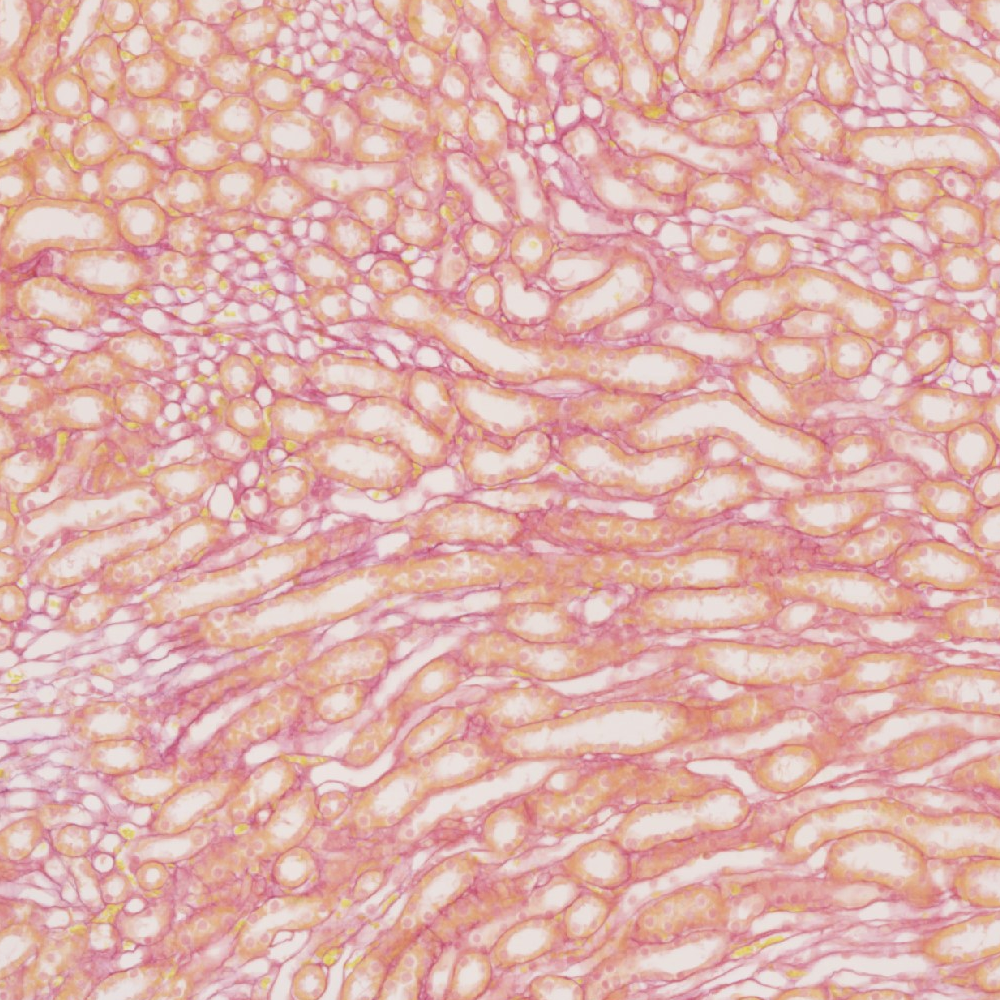

Supplement: Supplementary file 6 — Source Data for Figure 5 [file EMMM-15-e18242-s004.zip › Figure_5/5B/C171YC171Y_-_Picrosirius_red.tif]

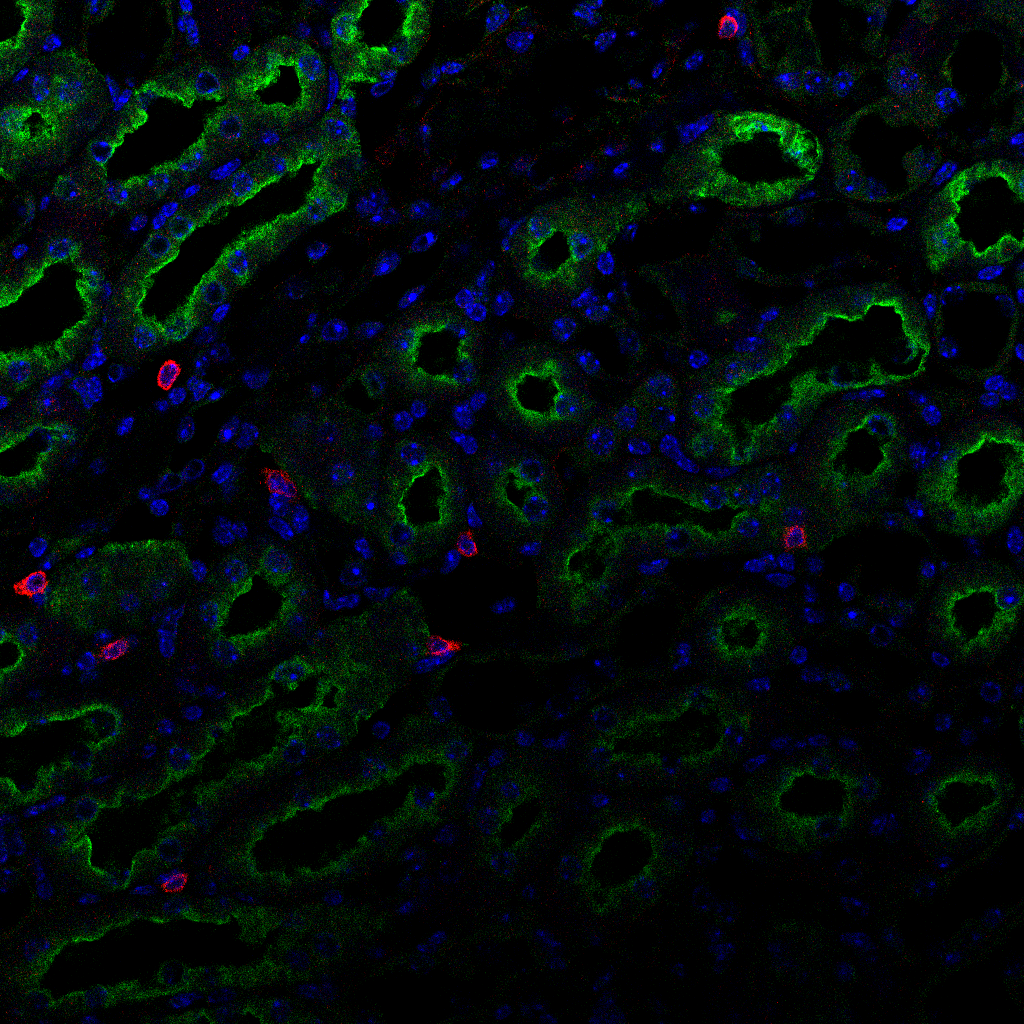

Supplement: Supplementary file 6 — Source Data for Figure 5 [file EMMM-15-e18242-s004.zip › Figure_5/5B/C171YC171Y_-_UMOD,_CD3.tif]

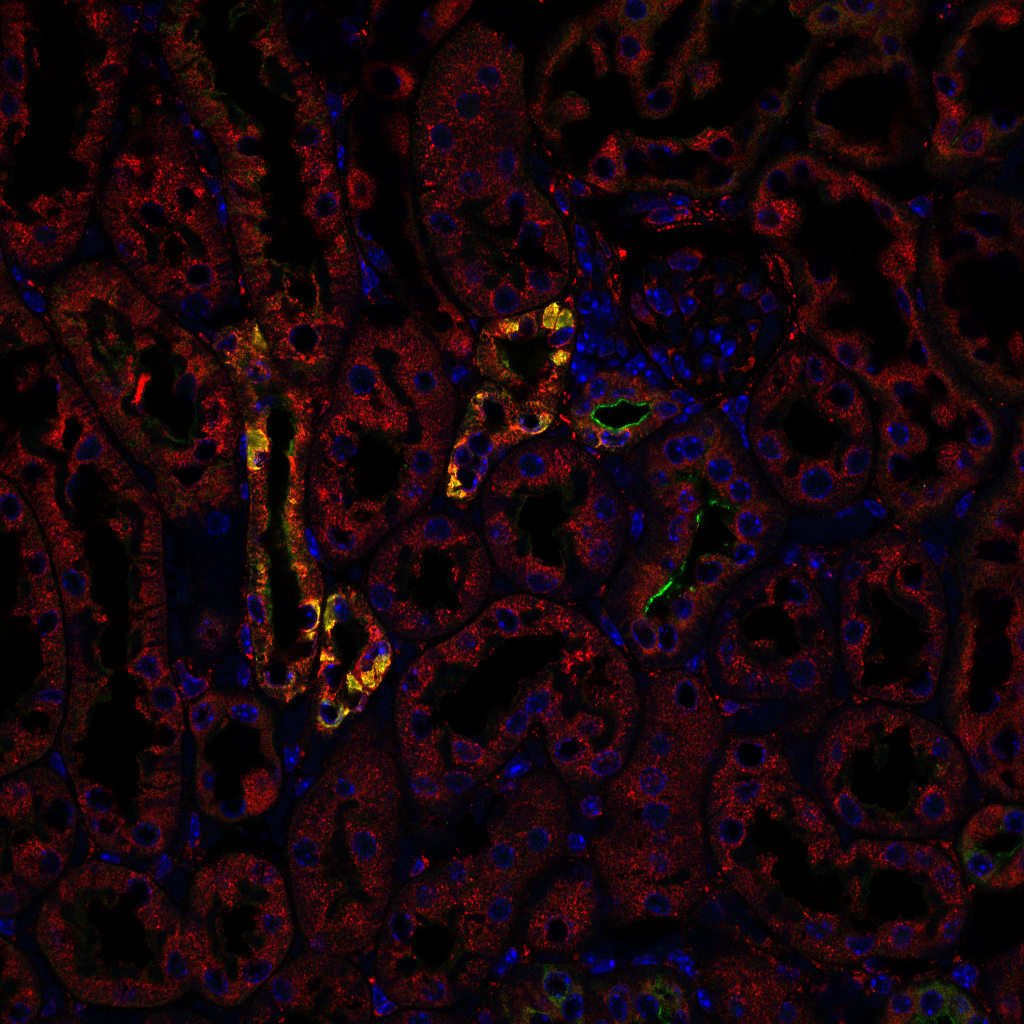

Supplement: Supplementary file 6 — Source Data for Figure 5 [file EMMM-15-e18242-s004.zip › Figure_5/5B/C171YC171Y_-_UMOD,_GRP78.tif]

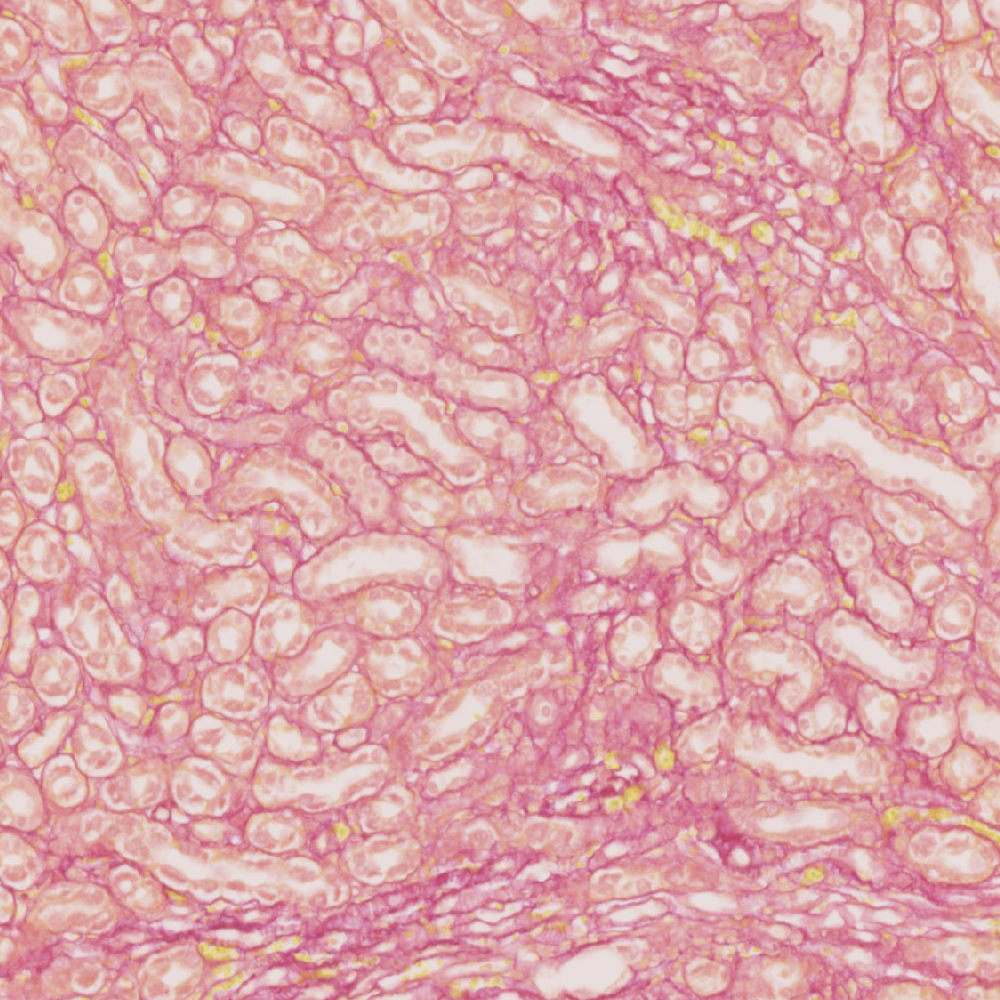

Supplement: Supplementary file 6 — Source Data for Figure 5 [file EMMM-15-e18242-s004.zip › Figure_5/5B/R186S+_-_Picrosirius_red.tif]

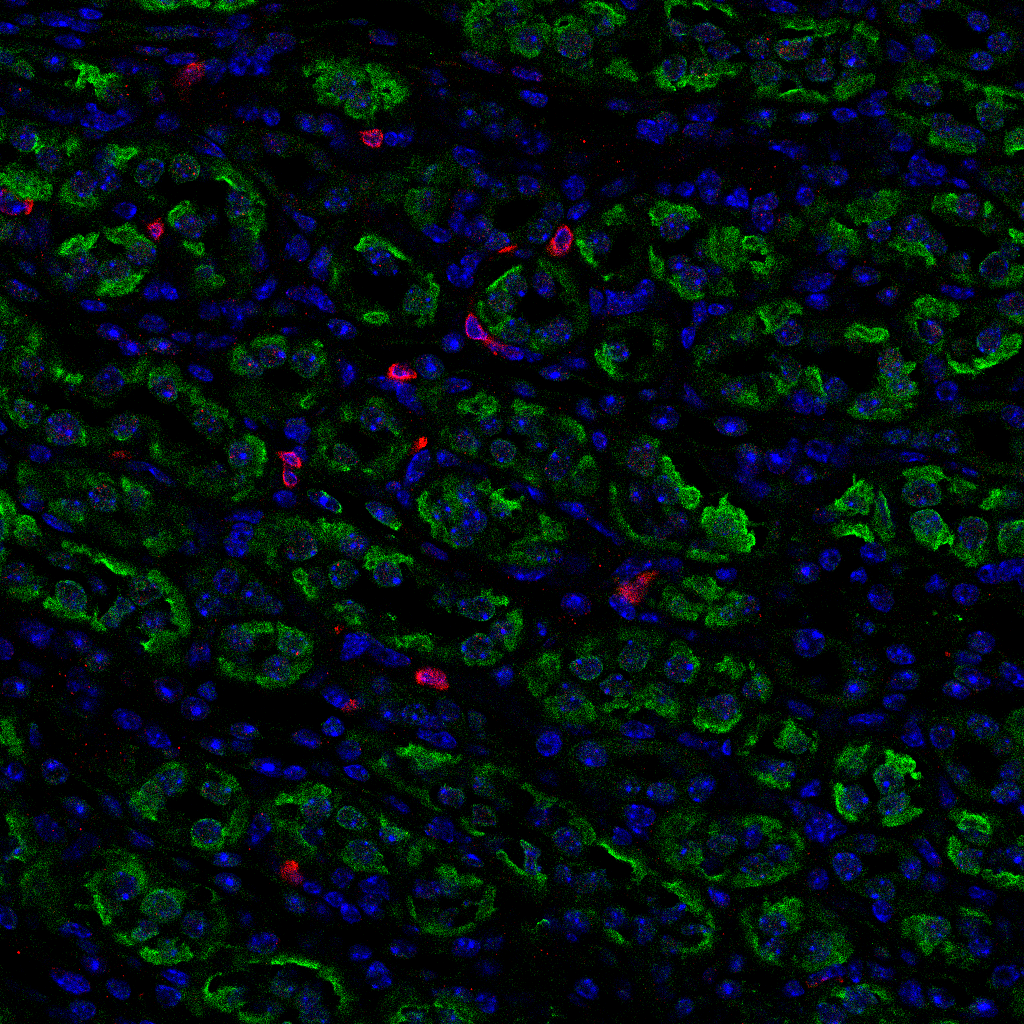

Supplement: Supplementary file 6 — Source Data for Figure 5 [file EMMM-15-e18242-s004.zip › Figure_5/5B/R186S+_-_UMOD,_CD3.tif]

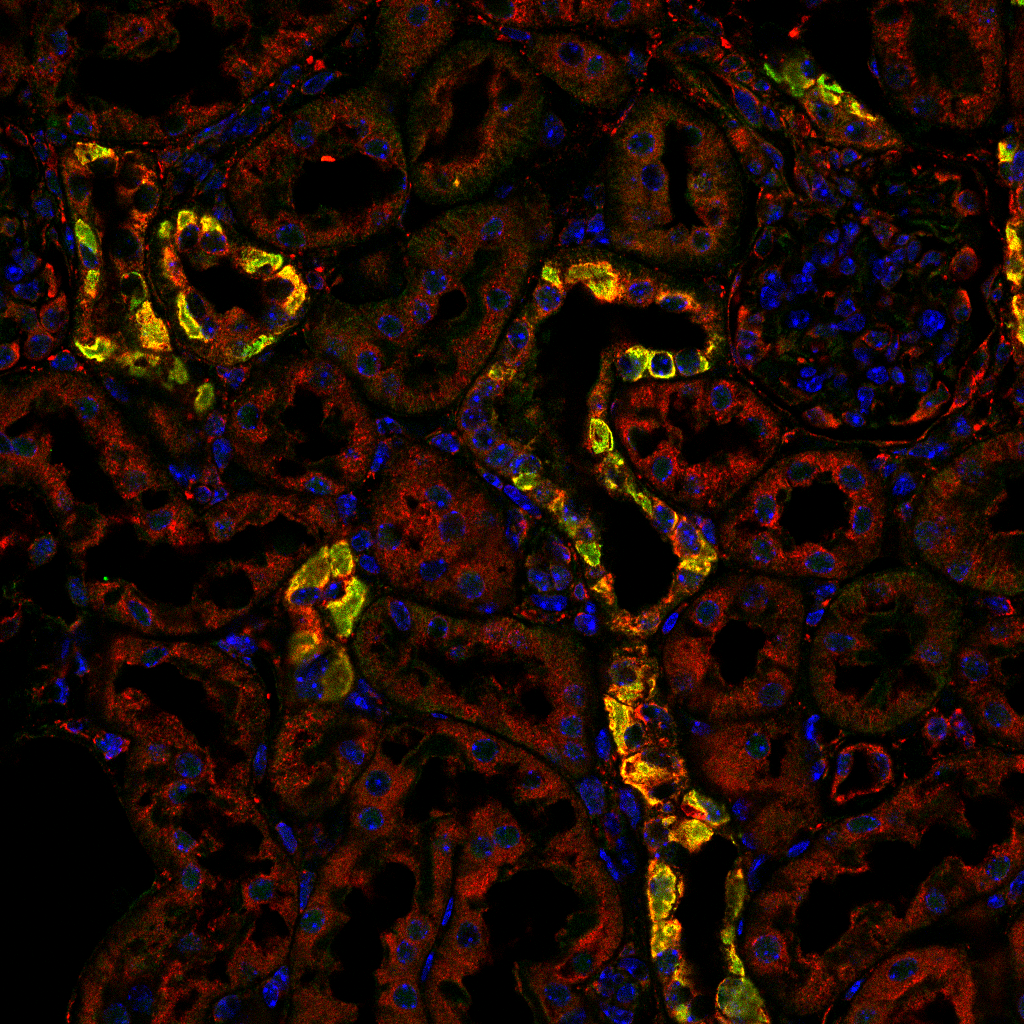

Supplement: Supplementary file 6 — Source Data for Figure 5 [file EMMM-15-e18242-s004.zip › Figure_5/5B/R186S+_-_UMOD,_GRP78.tif]

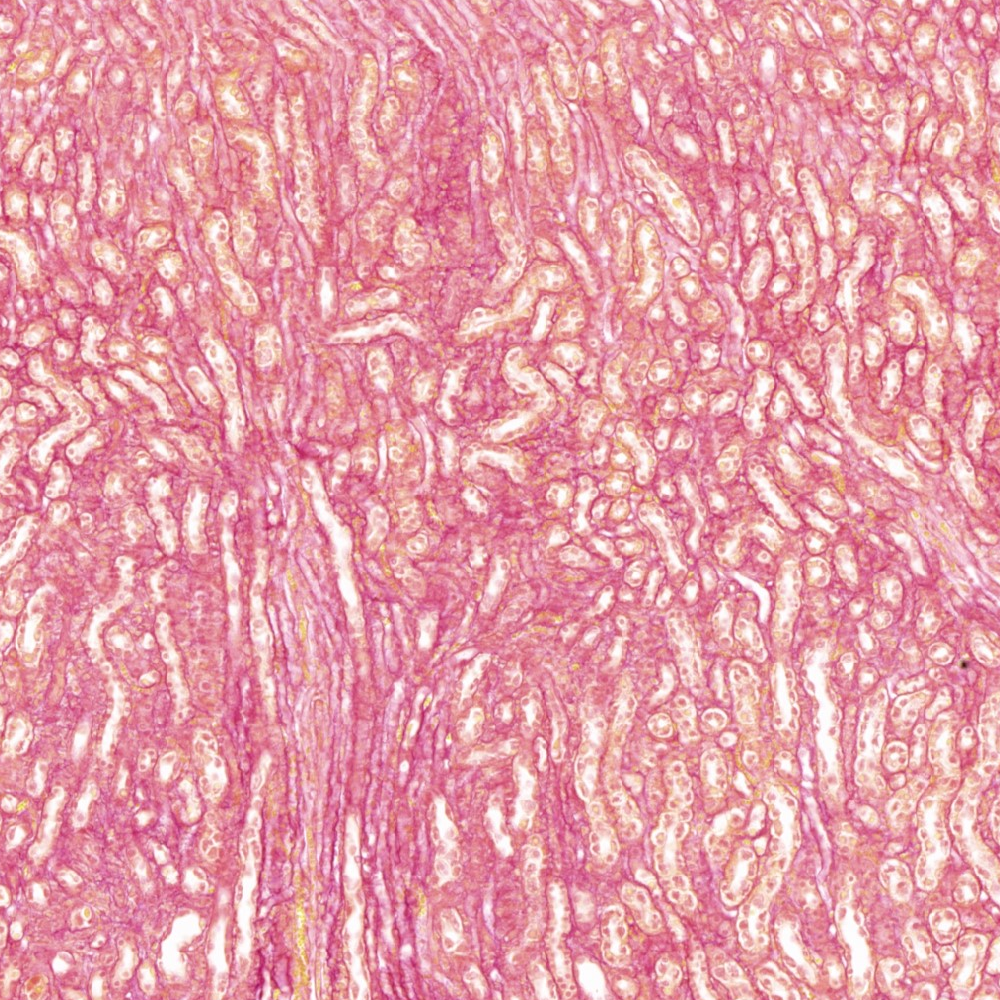

Supplement: Supplementary file 6 — Source Data for Figure 5 [file EMMM-15-e18242-s004.zip › Figure_5/5B/R186S-_-_Picrosirius_red.tif]

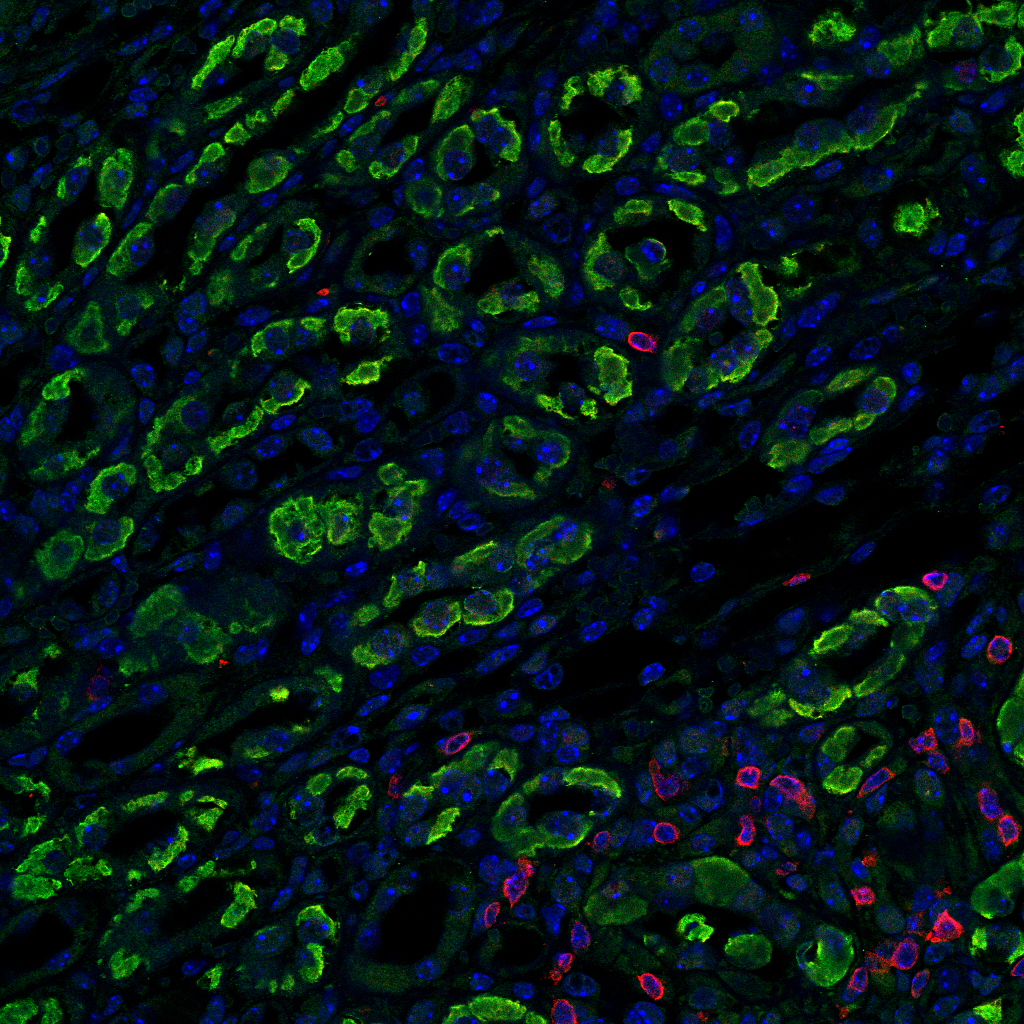

Supplement: Supplementary file 6 — Source Data for Figure 5 [file EMMM-15-e18242-s004.zip › Figure_5/5B/R186S-_-_UMOD,_CD3.tif]

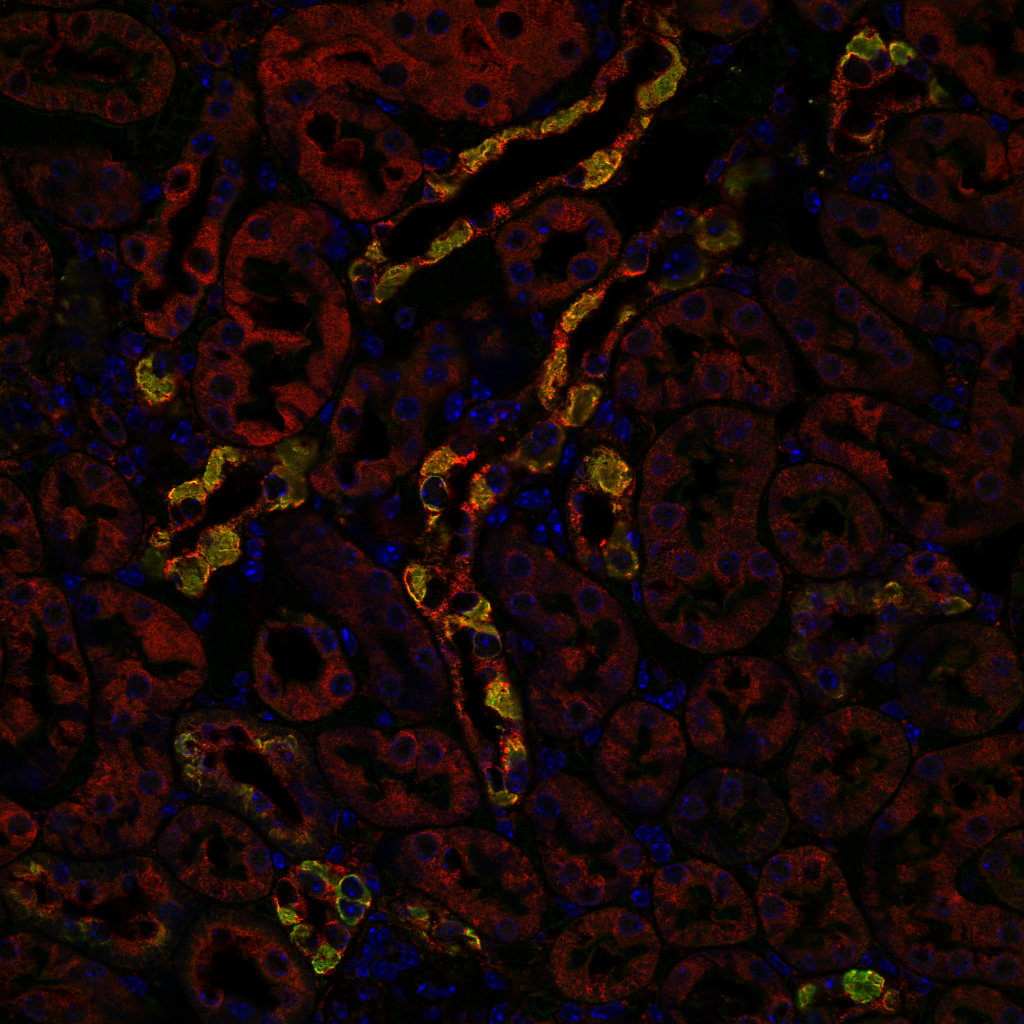

Supplement: Supplementary file 6 — Source Data for Figure 5 [file EMMM-15-e18242-s004.zip › Figure_5/5B/R186S-_-_UMOD,_GRP78.tif]

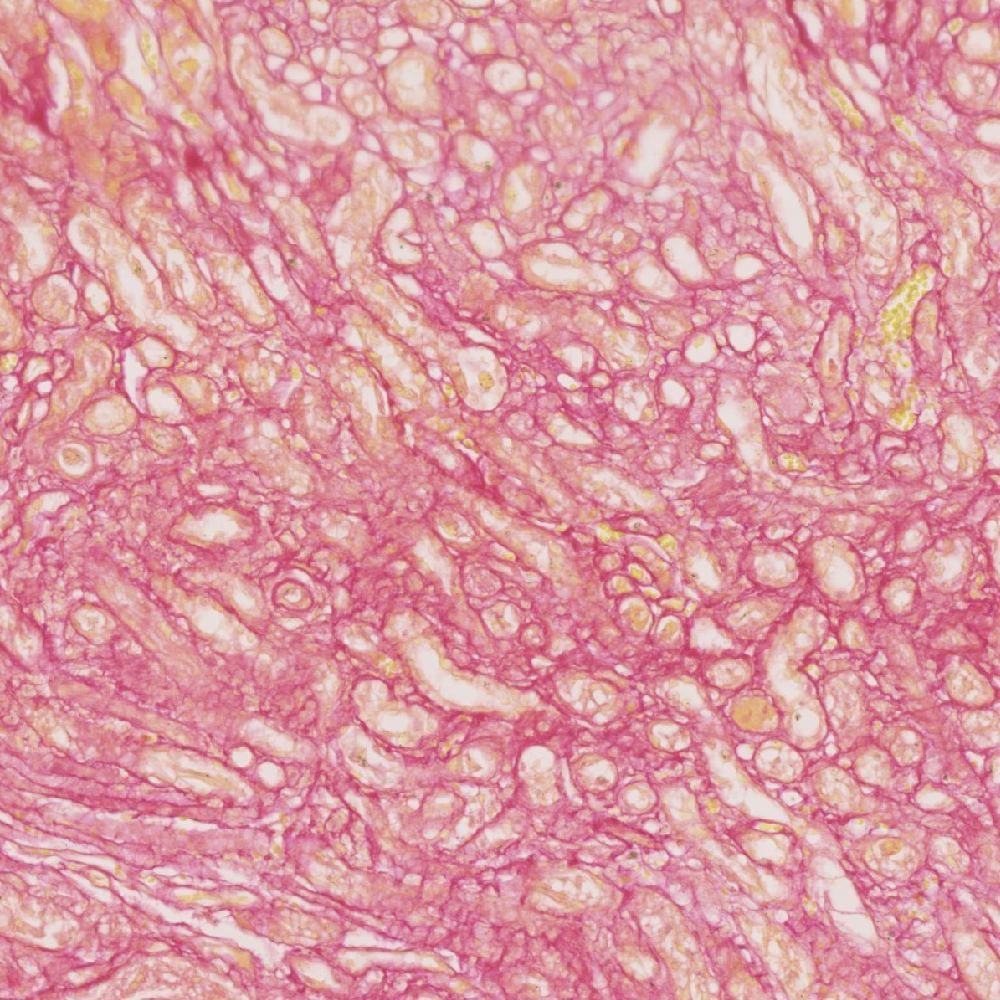

Supplement: Supplementary file 6 — Source Data for Figure 5 [file EMMM-15-e18242-s004.zip › Figure_5/5B/R186SR186S_-_Picrosirius_red.tif]

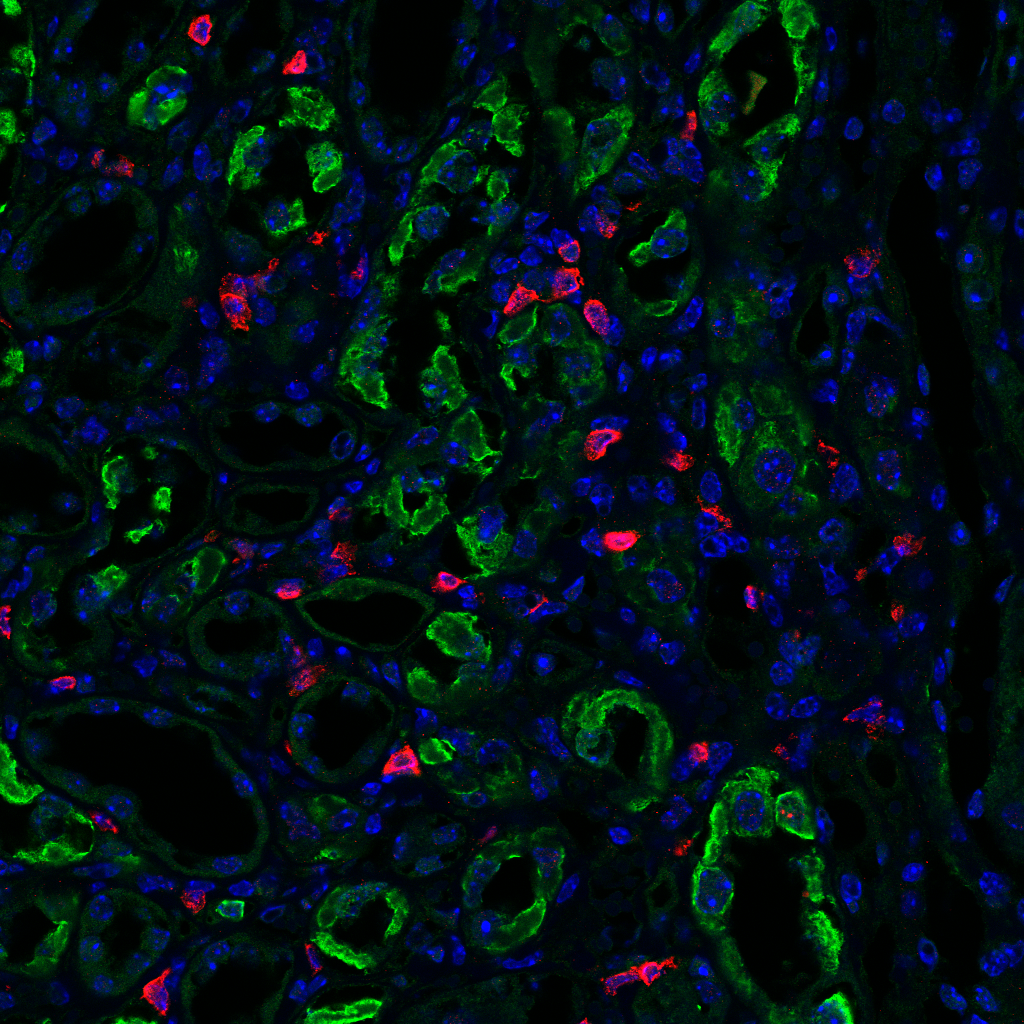

Supplement: Supplementary file 6 — Source Data for Figure 5 [file EMMM-15-e18242-s004.zip › Figure_5/5B/R186SR186S_-_UMOD,_CD3.tif]

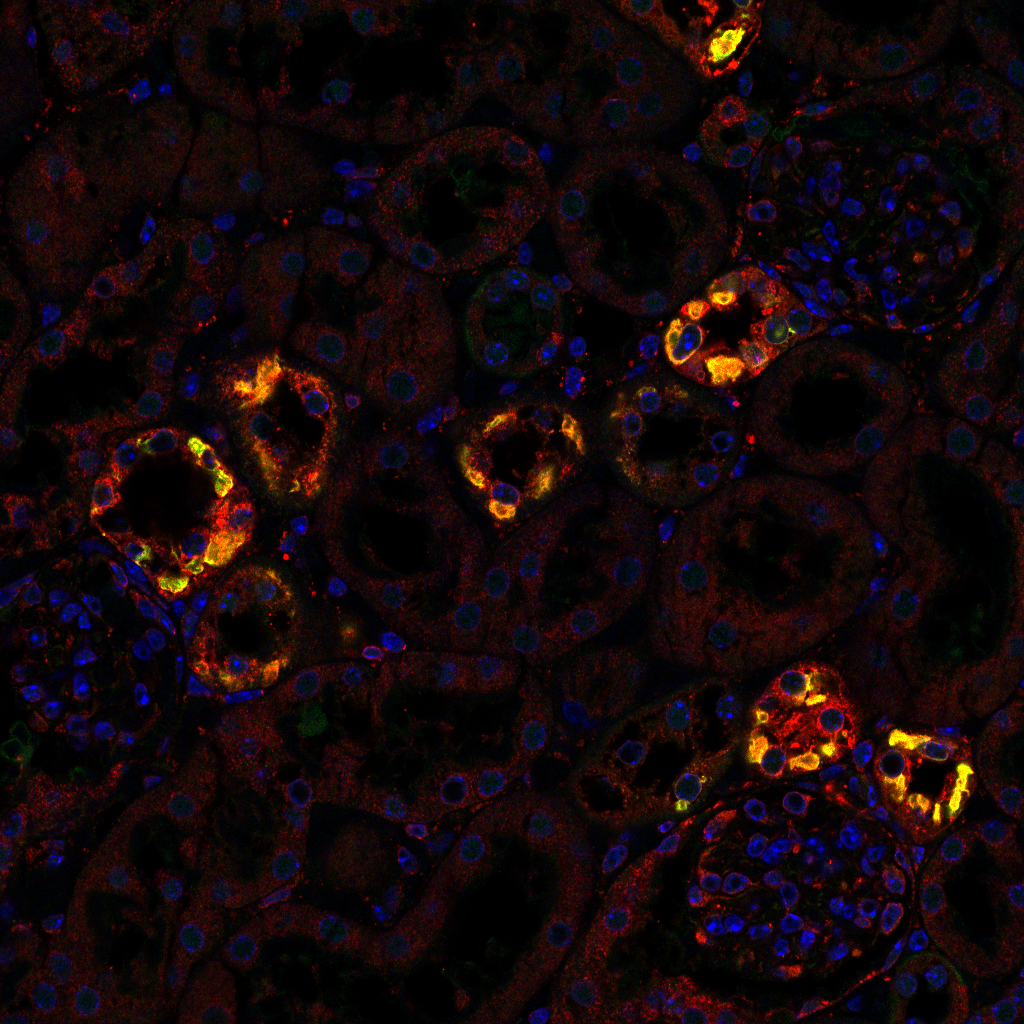

Supplement: Supplementary file 6 — Source Data for Figure 5 [file EMMM-15-e18242-s004.zip › Figure_5/5B/R186SR186S_-_UMOD,_GRP78.tif]

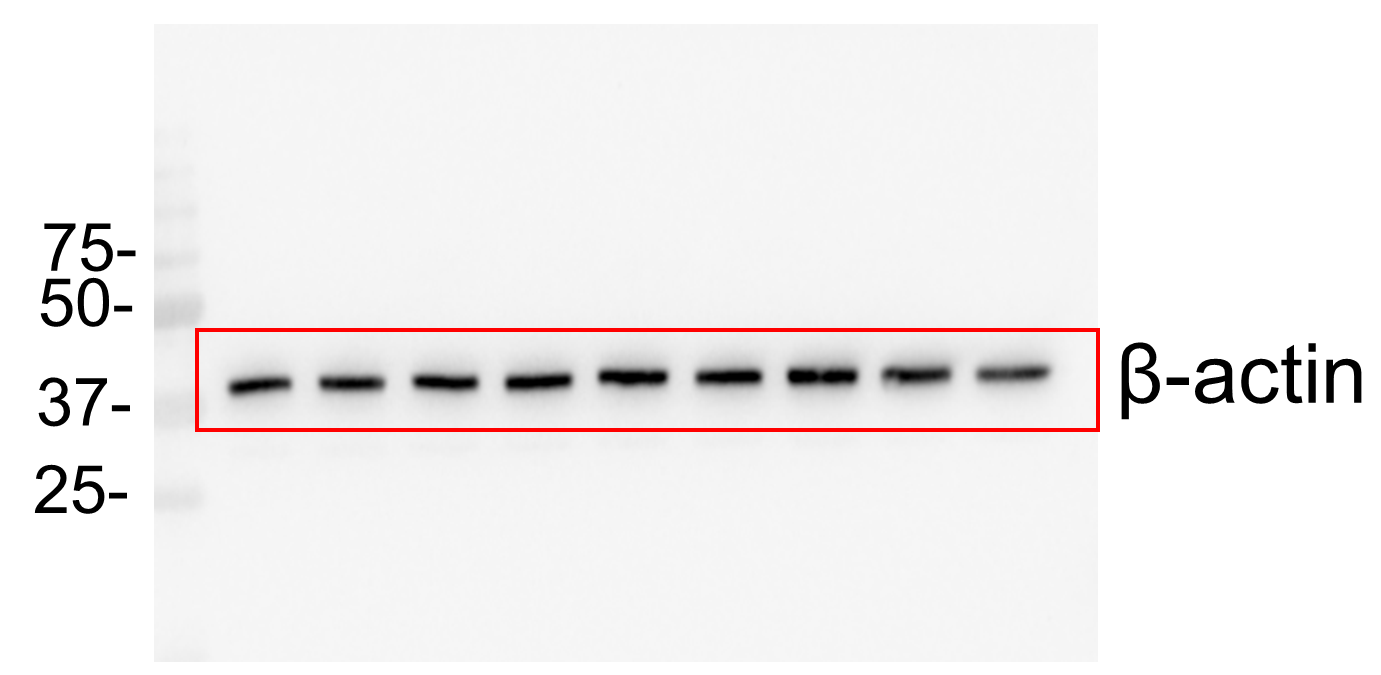

Supplement: Supplementary file 8 — Source Data for Figure 7 [file EMMM-15-e18242-s010.zip › Figure_7/7A/WB_beta-actin.tif]

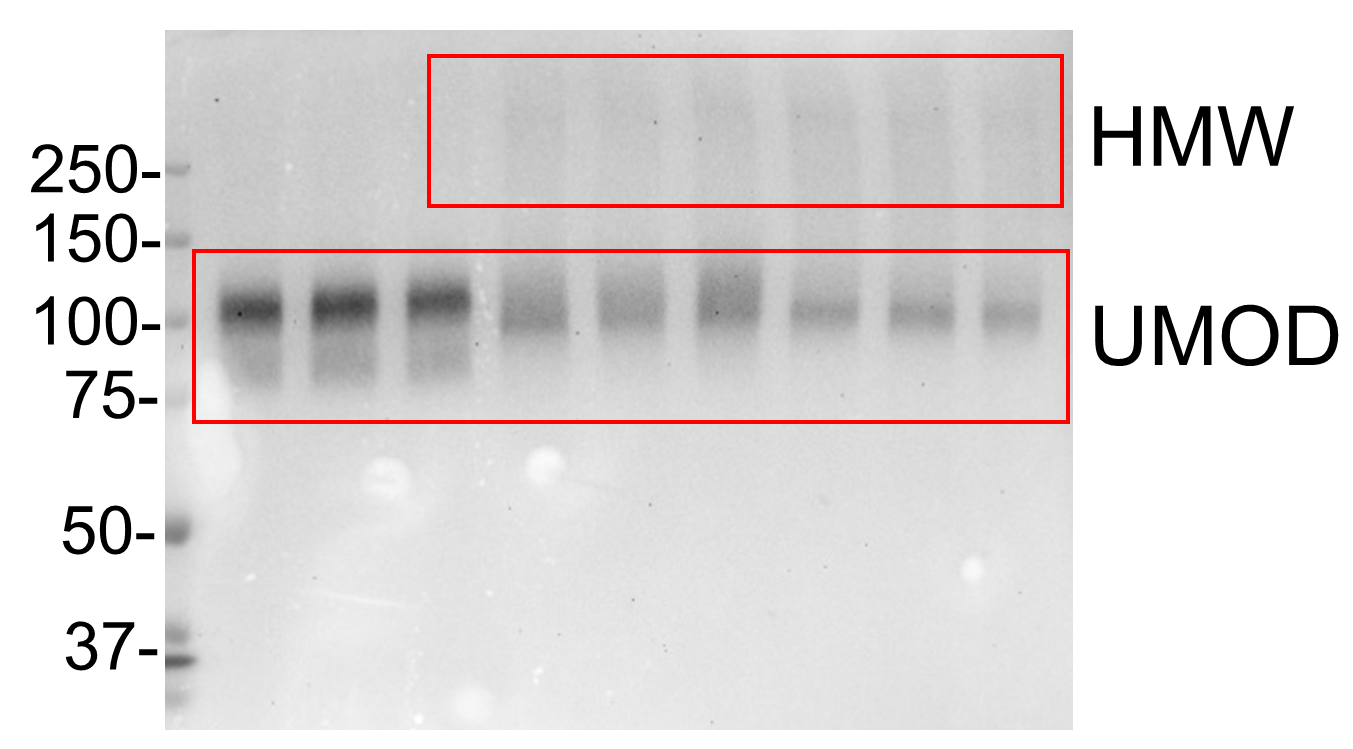

Supplement: Supplementary file 8 — Source Data for Figure 7 [file EMMM-15-e18242-s010.zip › Figure_7/7A/WB_UMOD.tif]

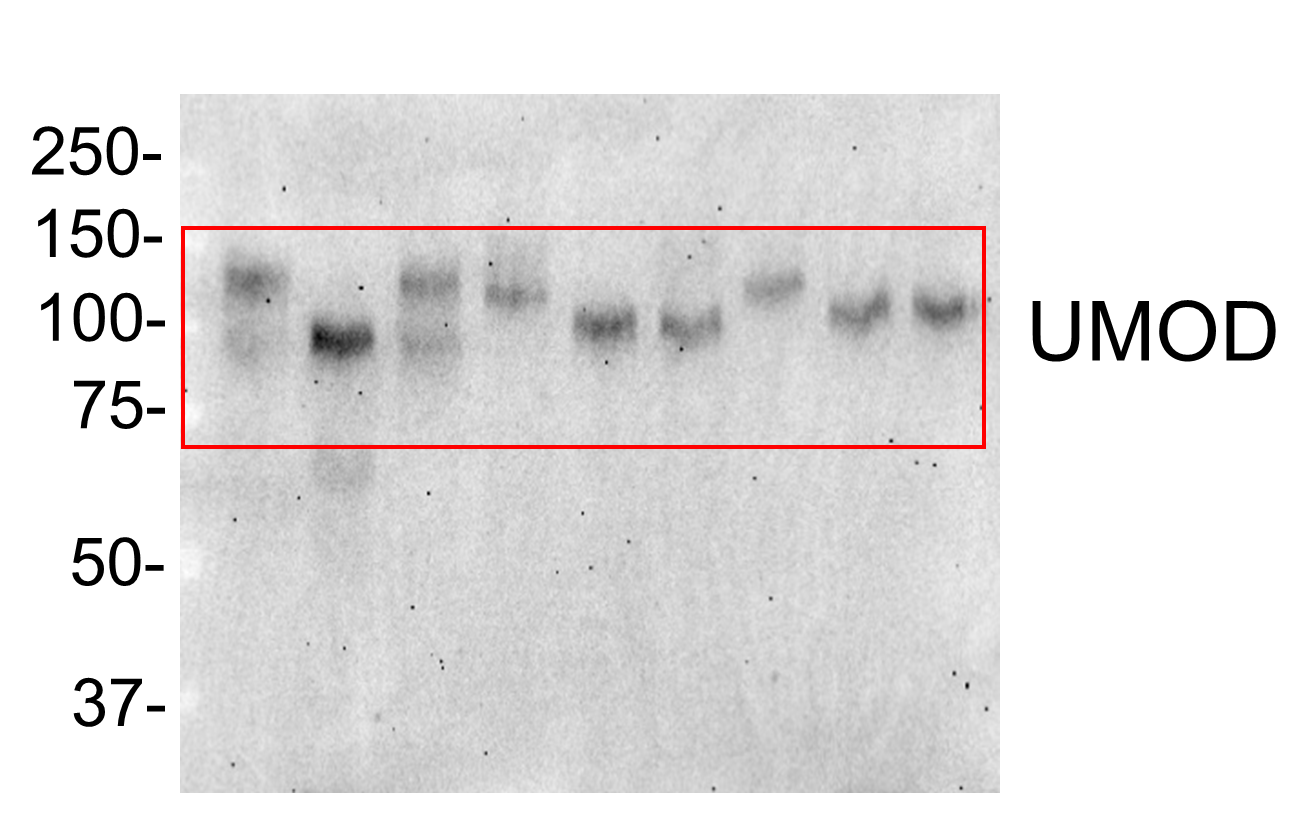

Supplement: Supplementary file 8 — Source Data for Figure 7 [file EMMM-15-e18242-s010.zip › Figure_7/7B/WB_UMOD_PNGaseF_Endo_H.tif]

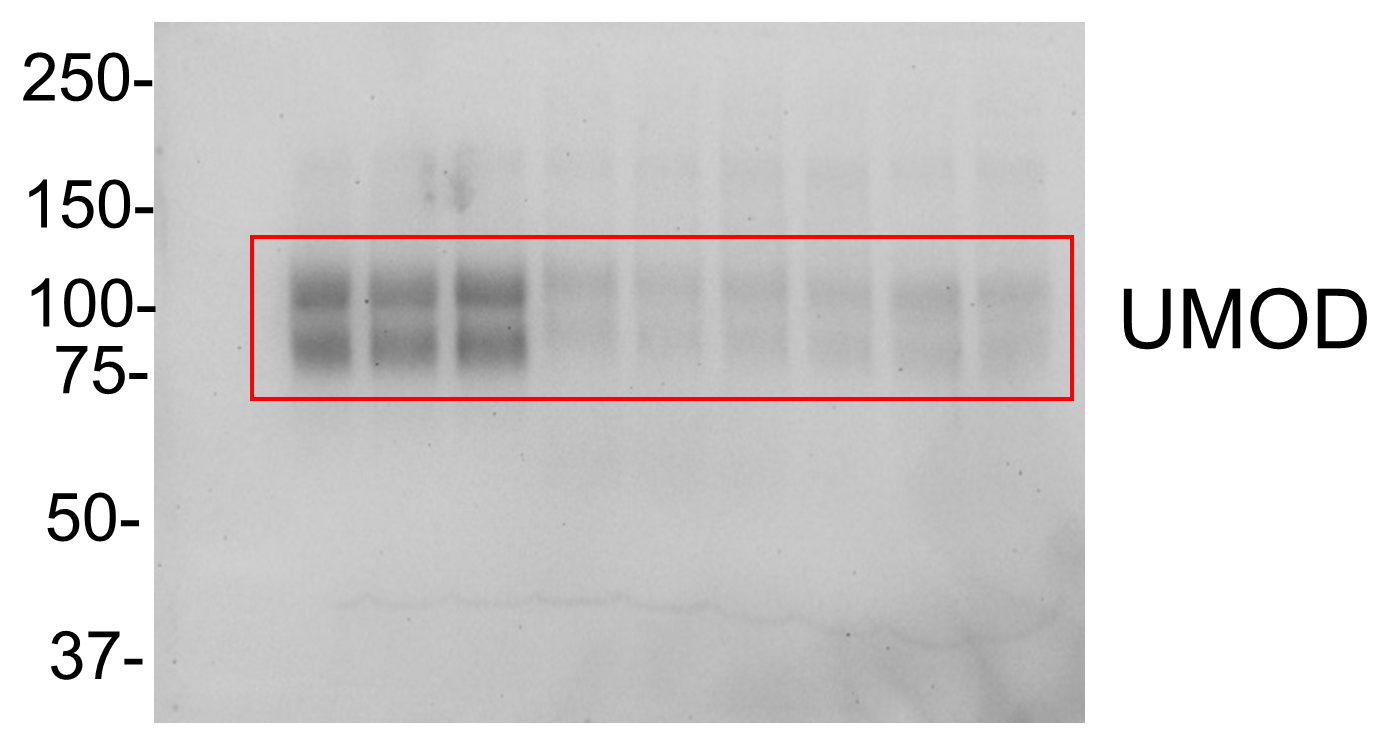

Supplement: Supplementary file 8 — Source Data for Figure 7 [file EMMM-15-e18242-s010.zip › Figure_7/7C/WB_secreted_UMOD.tif]

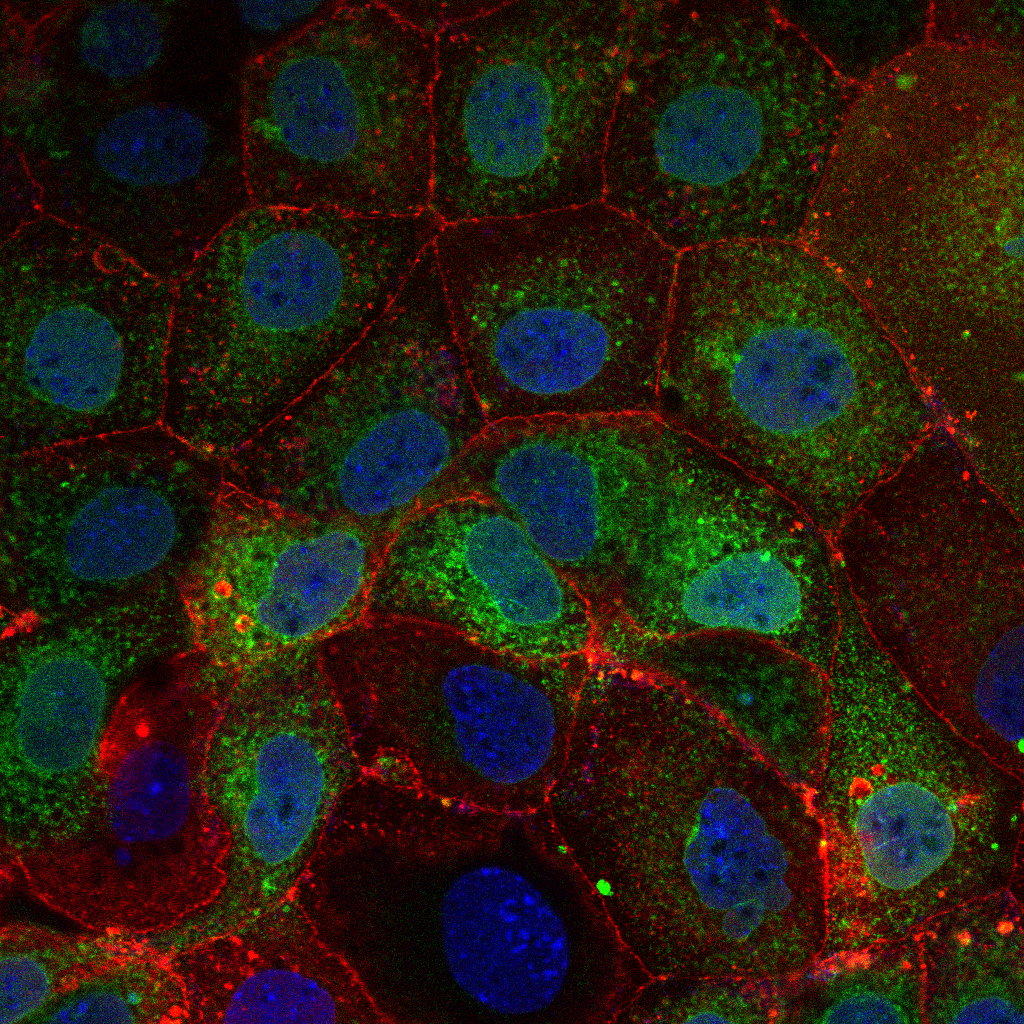

Supplement: Supplementary file 8 — Source Data for Figure 7 [file EMMM-15-e18242-s010.zip › Figure_7/7D/C170Y_-_UMOD,_WGA.tif]

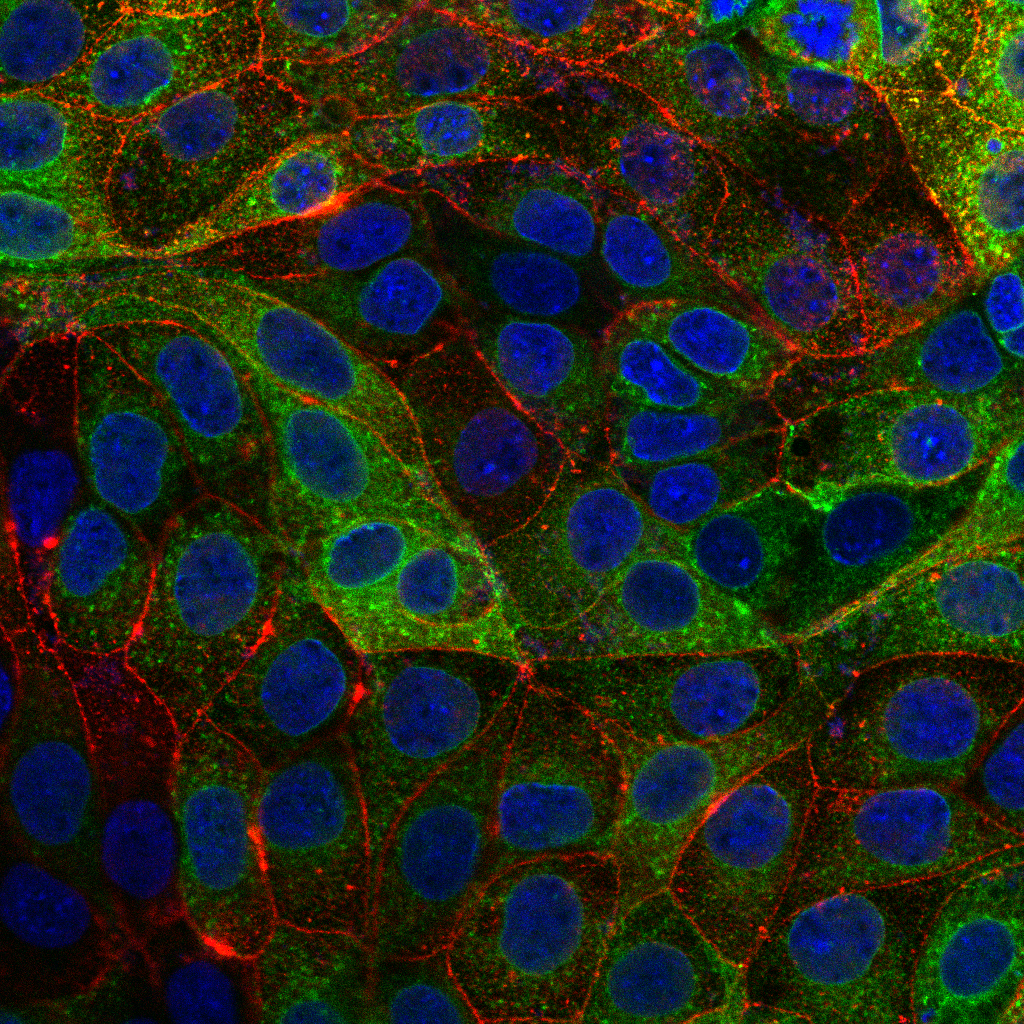

Supplement: Supplementary file 8 — Source Data for Figure 7 [file EMMM-15-e18242-s010.zip › Figure_7/7D/R185S_-_UMOD,_WGA.tif]

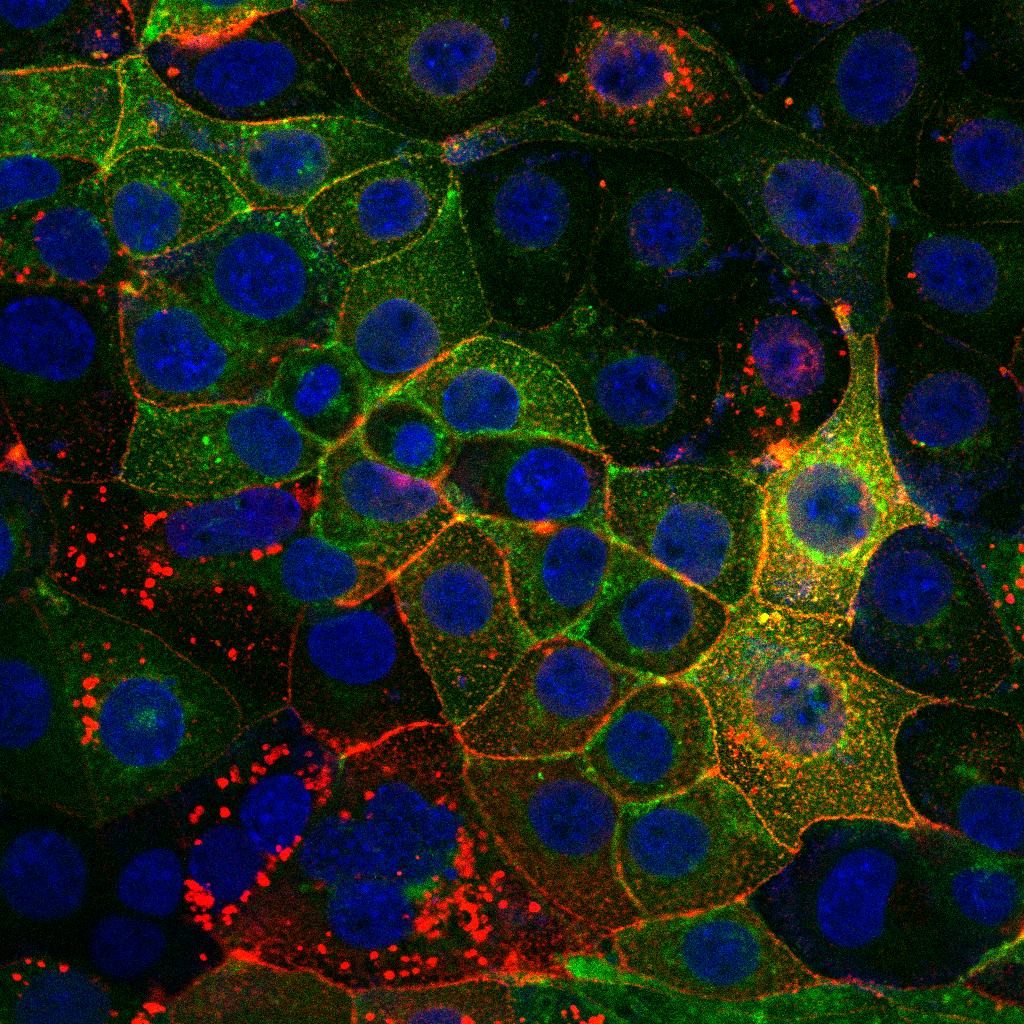

Supplement: Supplementary file 8 — Source Data for Figure 7 [file EMMM-15-e18242-s010.zip › Figure_7/7D/WT__-_UMOD,_WGA.tif]

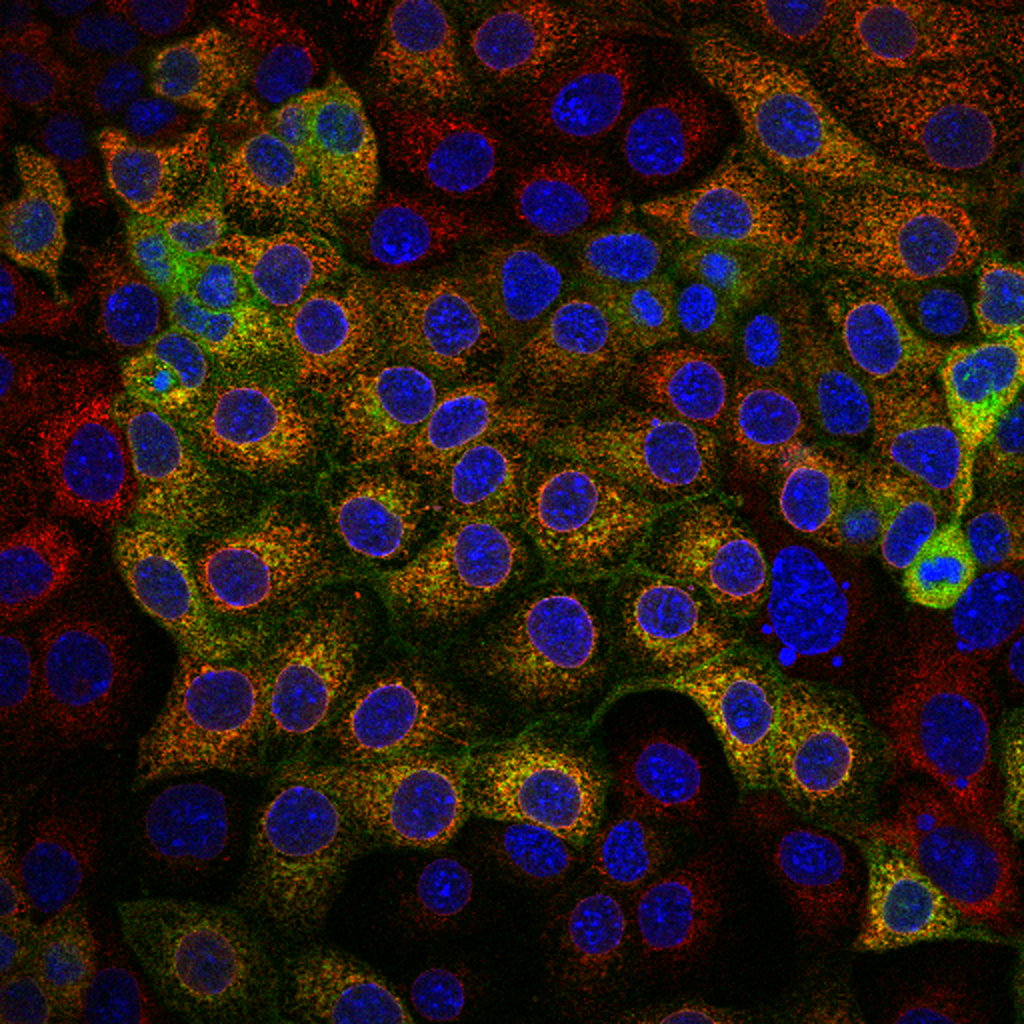

Supplement: Supplementary file 8 — Source Data for Figure 7 [file EMMM-15-e18242-s010.zip › Figure_7/7E/C170Y_-_UMOD,_CNX.tif]

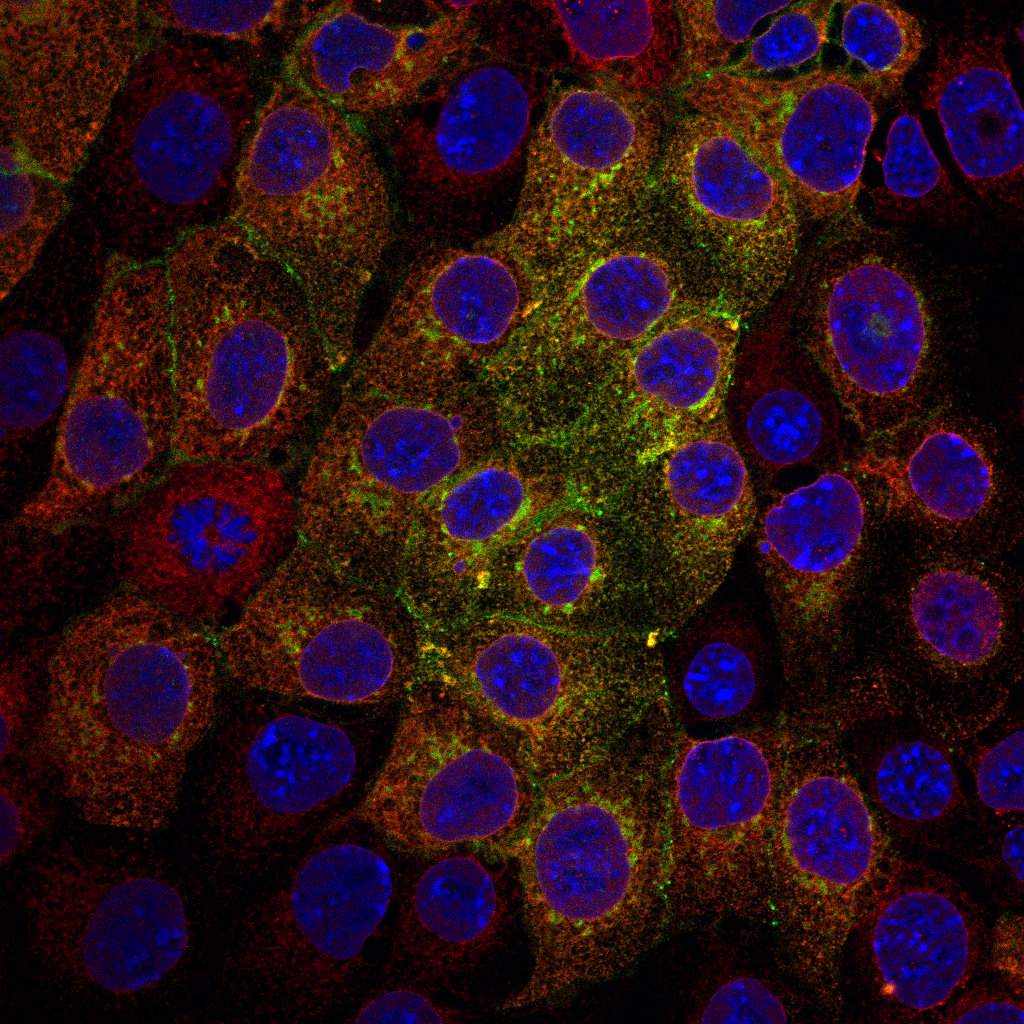

Supplement: Supplementary file 8 — Source Data for Figure 7 [file EMMM-15-e18242-s010.zip › Figure_7/7E/R185S_-_UMOD,_CNX.tif]

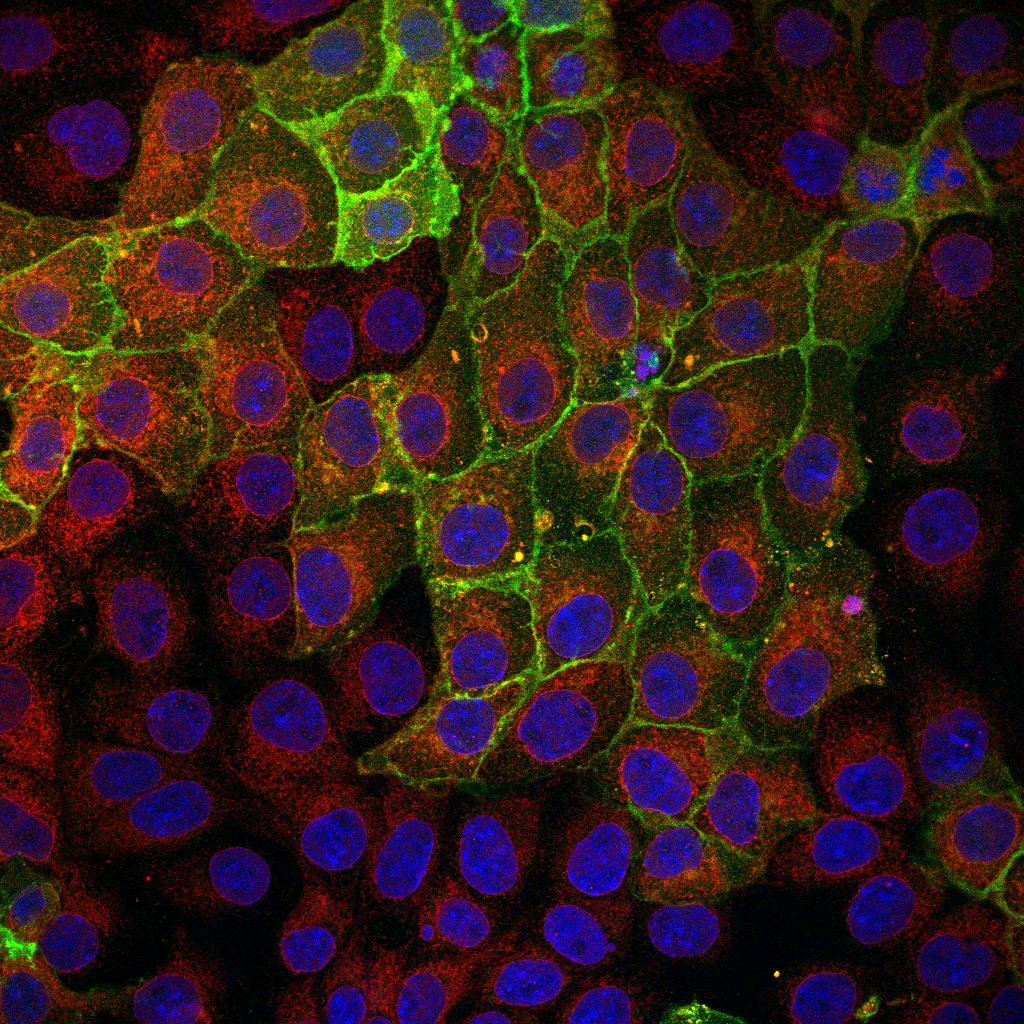

Supplement: Supplementary file 8 — Source Data for Figure 7 [file EMMM-15-e18242-s010.zip › Figure_7/7E/WT_-_UMOD,_CNX.tif]

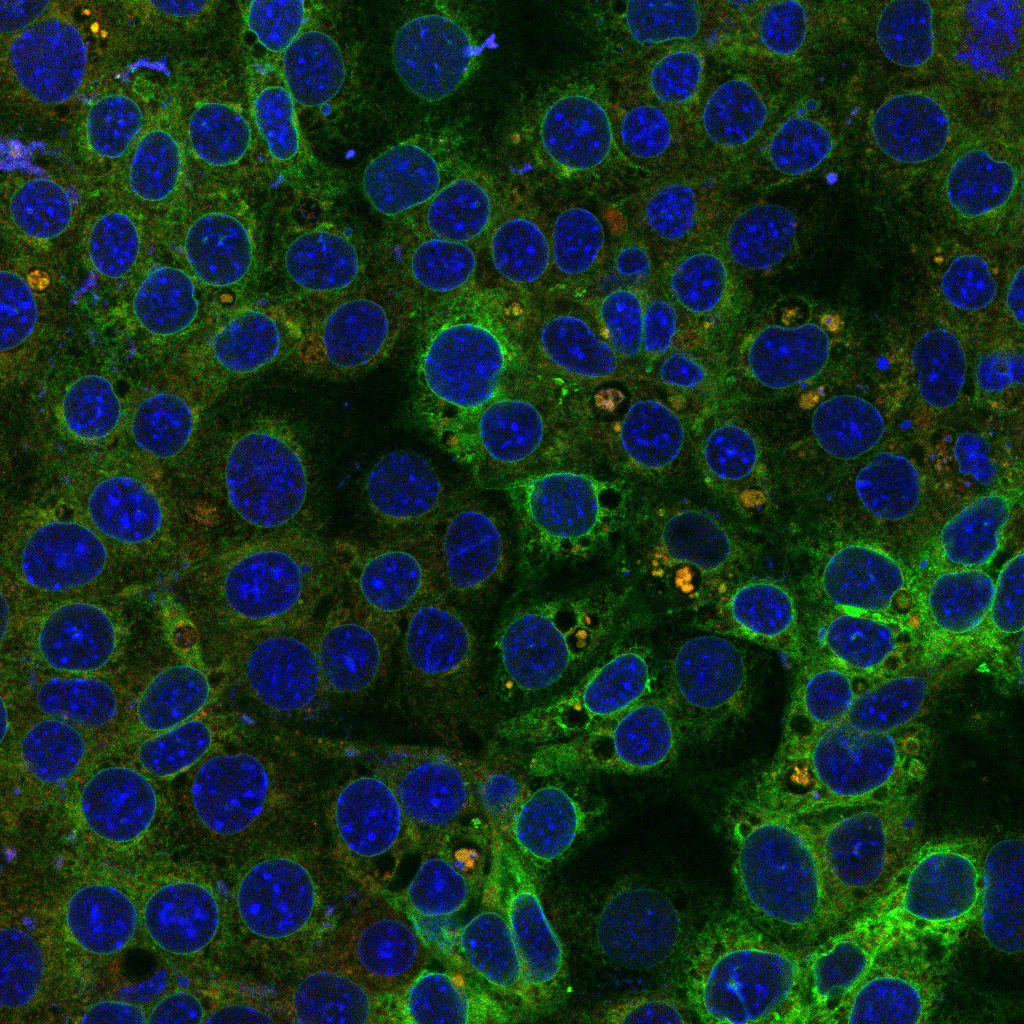

Supplement: Supplementary file 8 — Source Data for Figure 7 [file EMMM-15-e18242-s010.zip › Figure_7/7F/C170Y_-_UMOD,_Proteostat.tif]

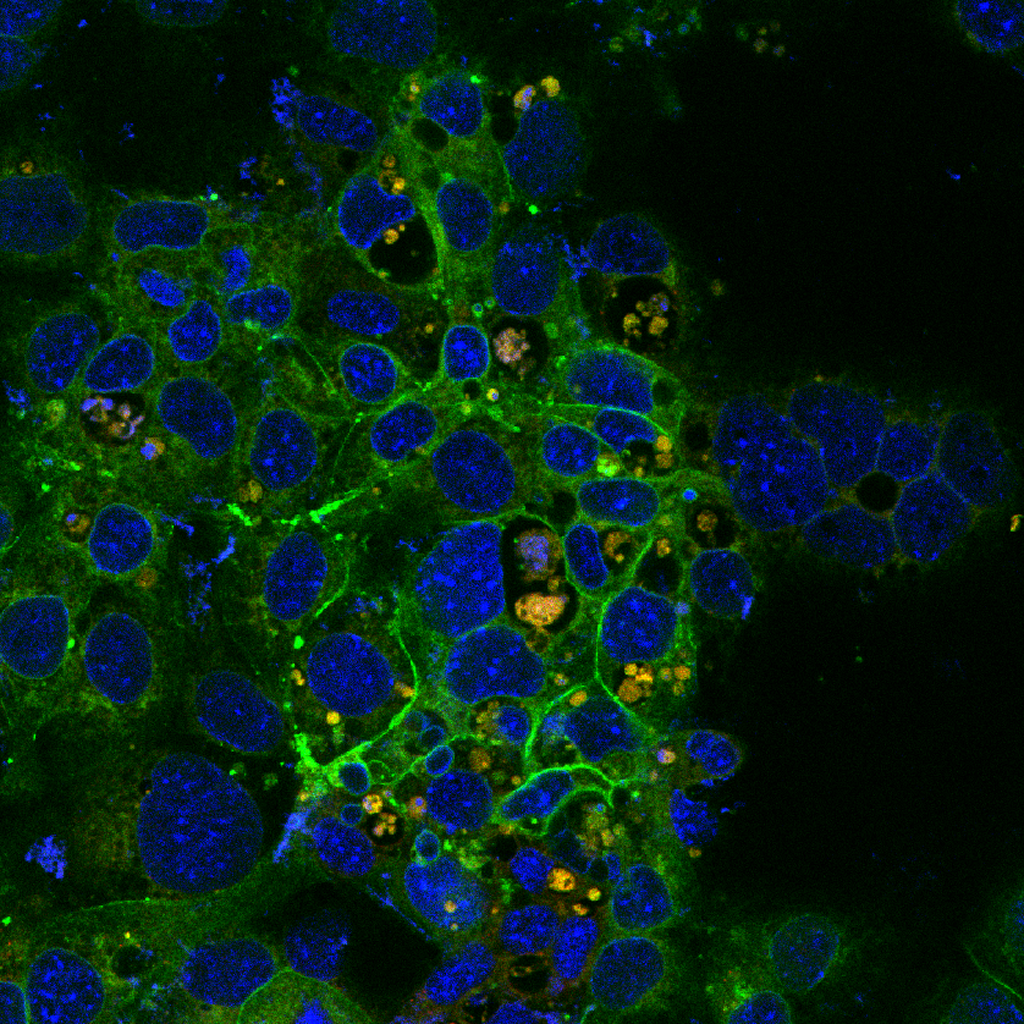

Supplement: Supplementary file 8 — Source Data for Figure 7 [file EMMM-15-e18242-s010.zip › Figure_7/7F/R185S_-_UMOD,_Proteostat.tif]

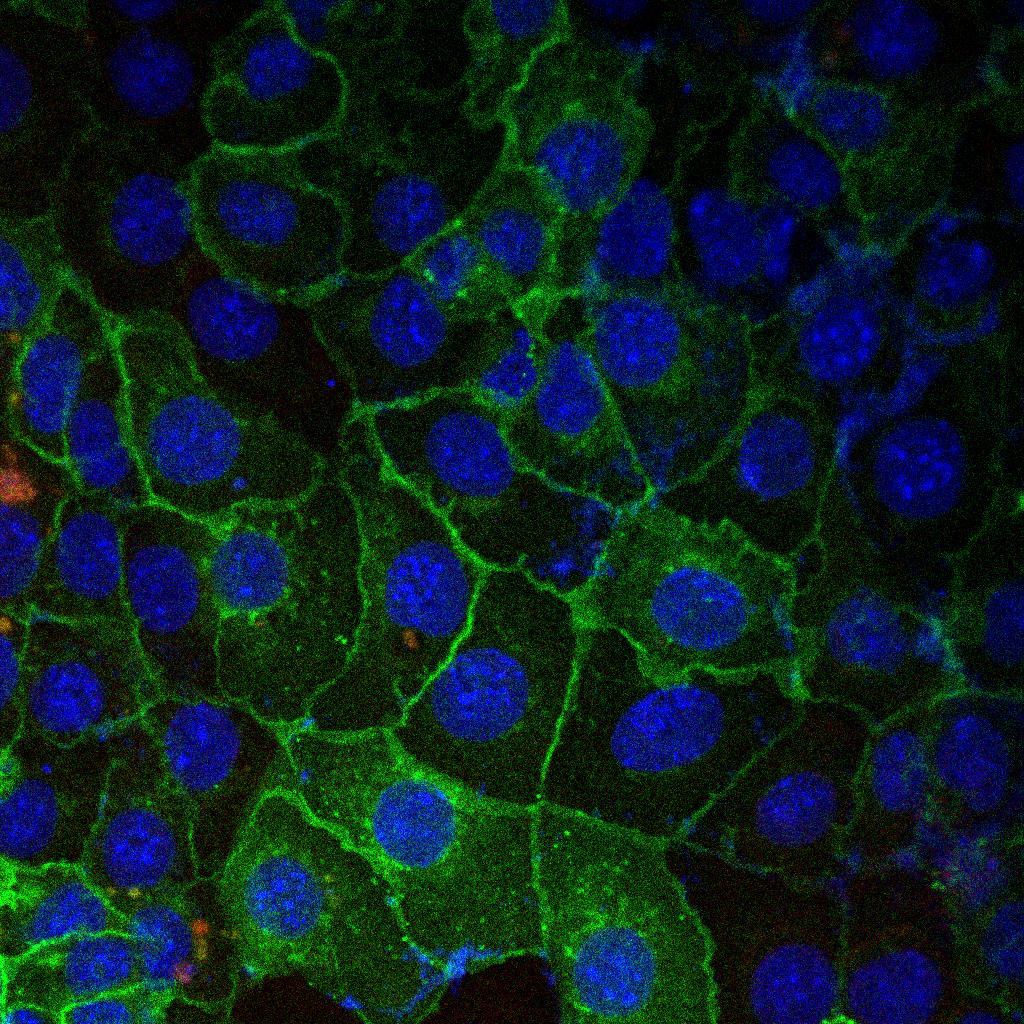

Supplement: Supplementary file 8 — Source Data for Figure 7 [file EMMM-15-e18242-s010.zip › Figure_7/7F/WT_-_UMOD,_Proteostat.tif]

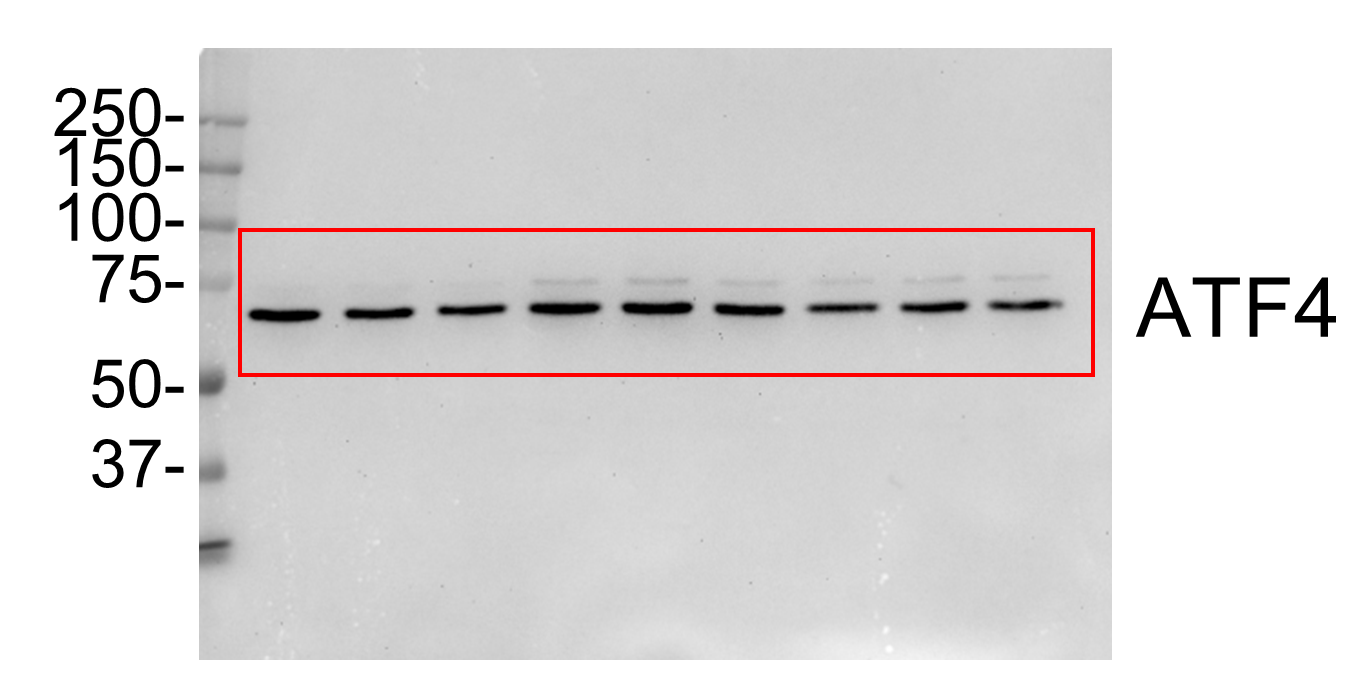

Supplement: Supplementary file 8 — Source Data for Figure 7 [file EMMM-15-e18242-s010.zip › Figure_7/7G/WB_ATF4.tif]

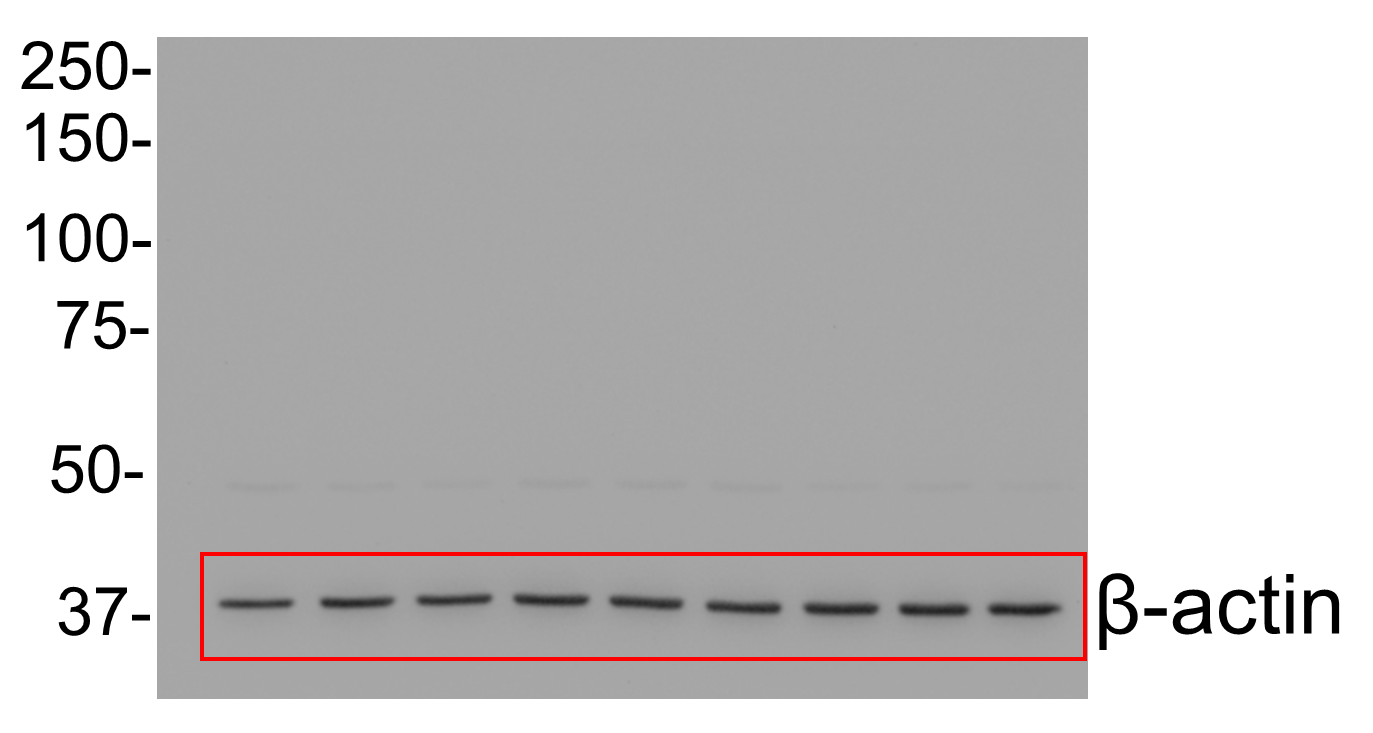

Supplement: Supplementary file 8 — Source Data for Figure 7 [file EMMM-15-e18242-s010.zip › Figure_7/7G/WB_beta-actin.tif]

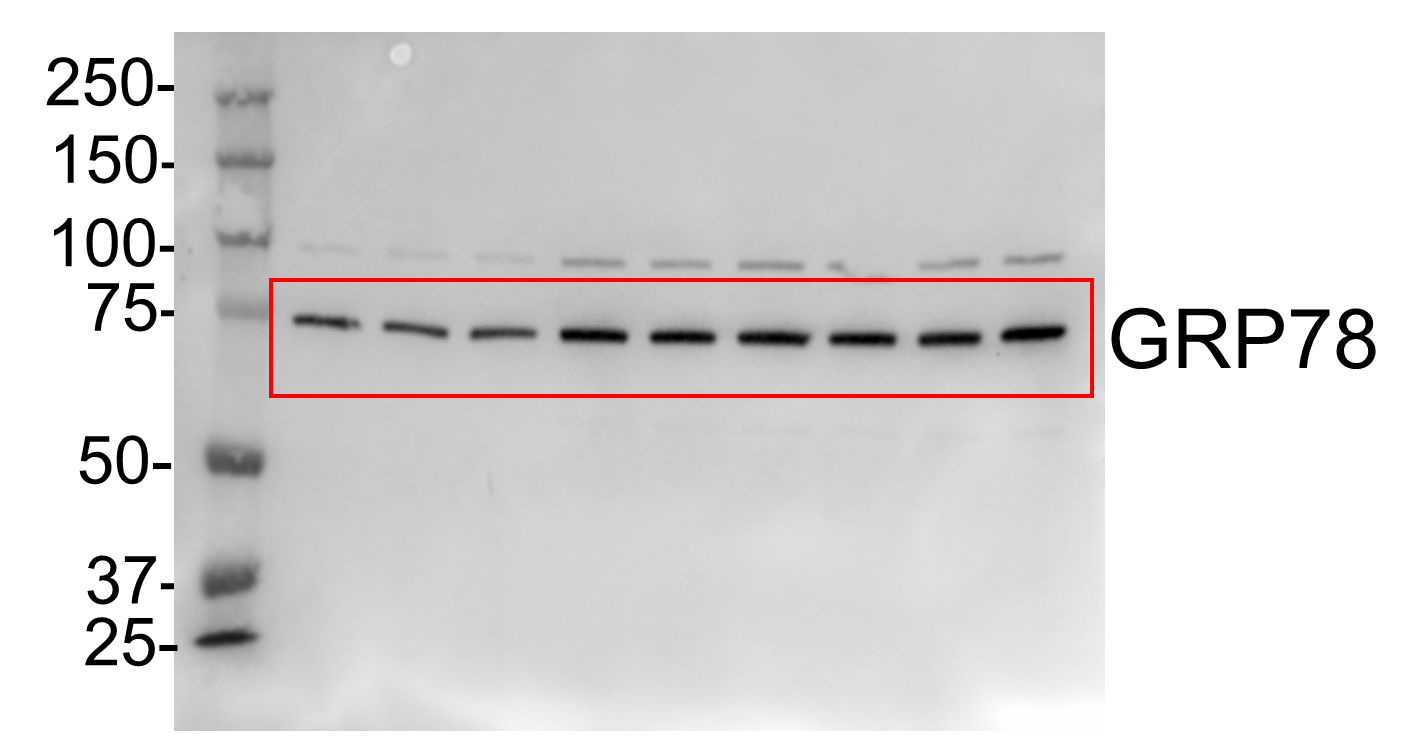

Supplement: Supplementary file 8 — Source Data for Figure 7 [file EMMM-15-e18242-s010.zip › Figure_7/7G/WB_GRP78.tif]

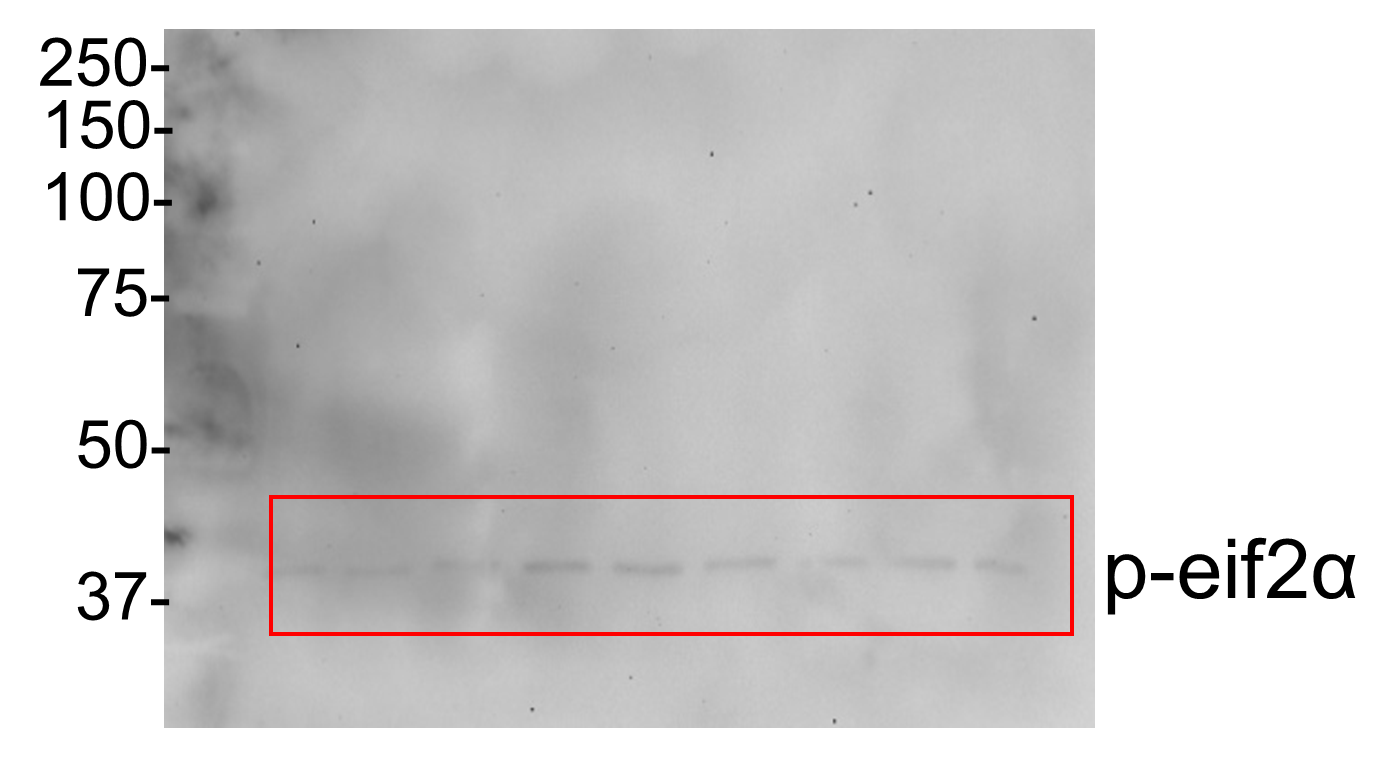

Supplement: Supplementary file 8 — Source Data for Figure 7 [file EMMM-15-e18242-s010.zip › Figure_7/7G/WB_p-eIF2_alpha.tif]

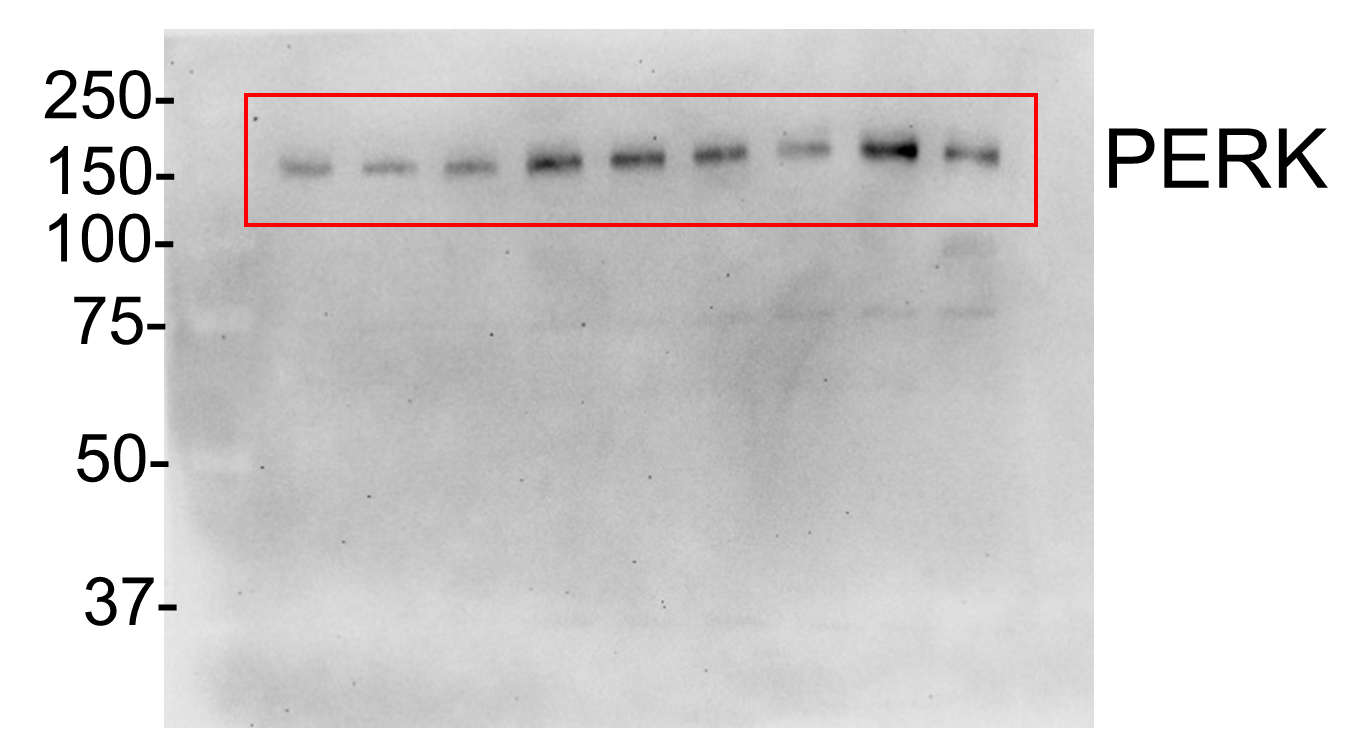

Supplement: Supplementary file 8 — Source Data for Figure 7 [file EMMM-15-e18242-s010.zip › Figure_7/7G/WB_PERK.tif]

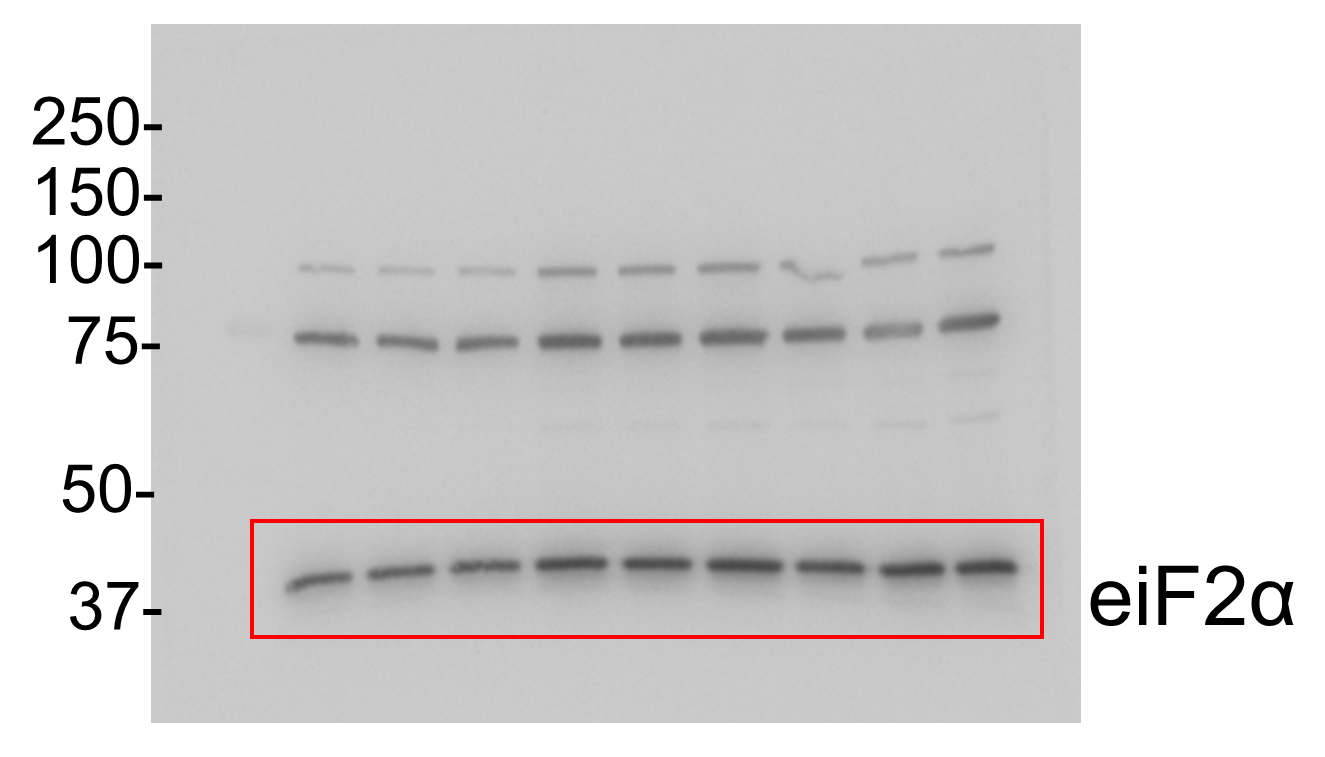

Supplement: Supplementary file 8 — Source Data for Figure 7 [file EMMM-15-e18242-s010.zip › Figure_7/7G/WB_t-eIF2_alpha.tif]

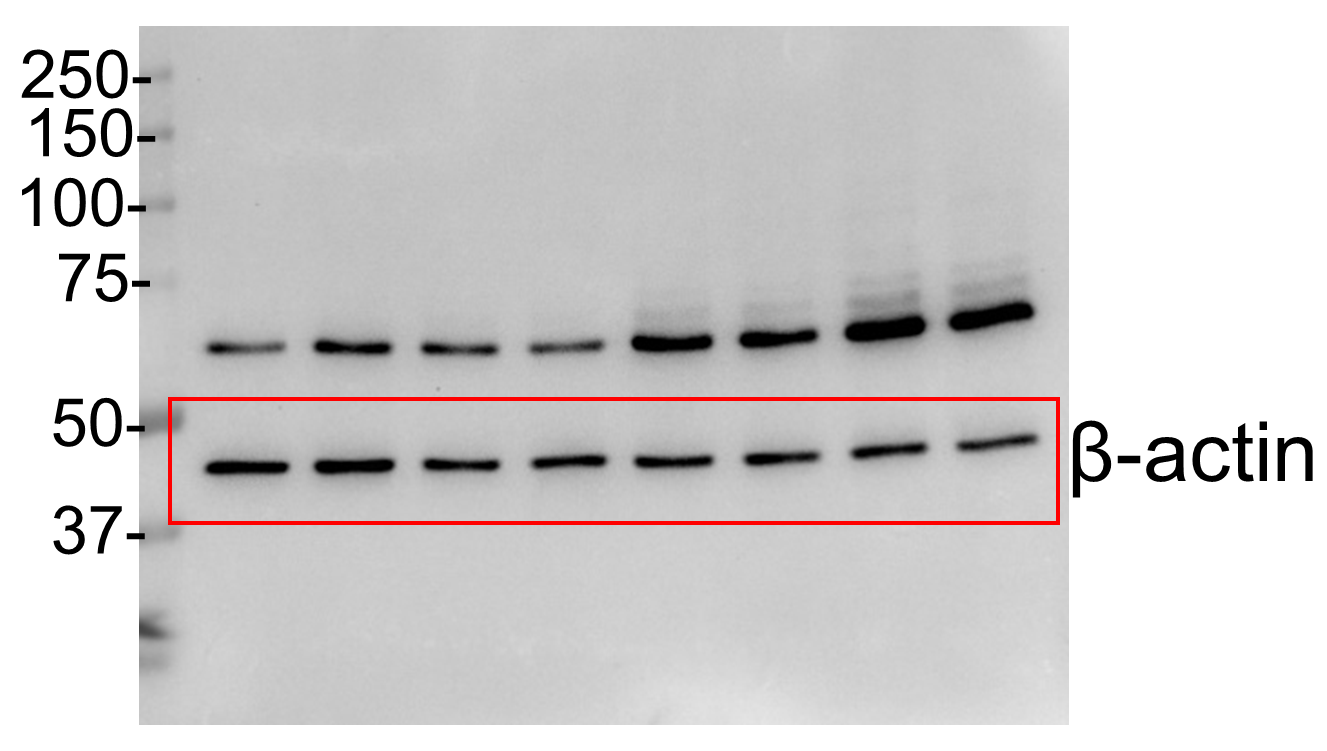

Supplement: Supplementary file 9 — Source Data for Figure 8 [file EMMM-15-e18242-s006.zip › Figure_8/8B/WB_beta-actin_C170Y.tif]

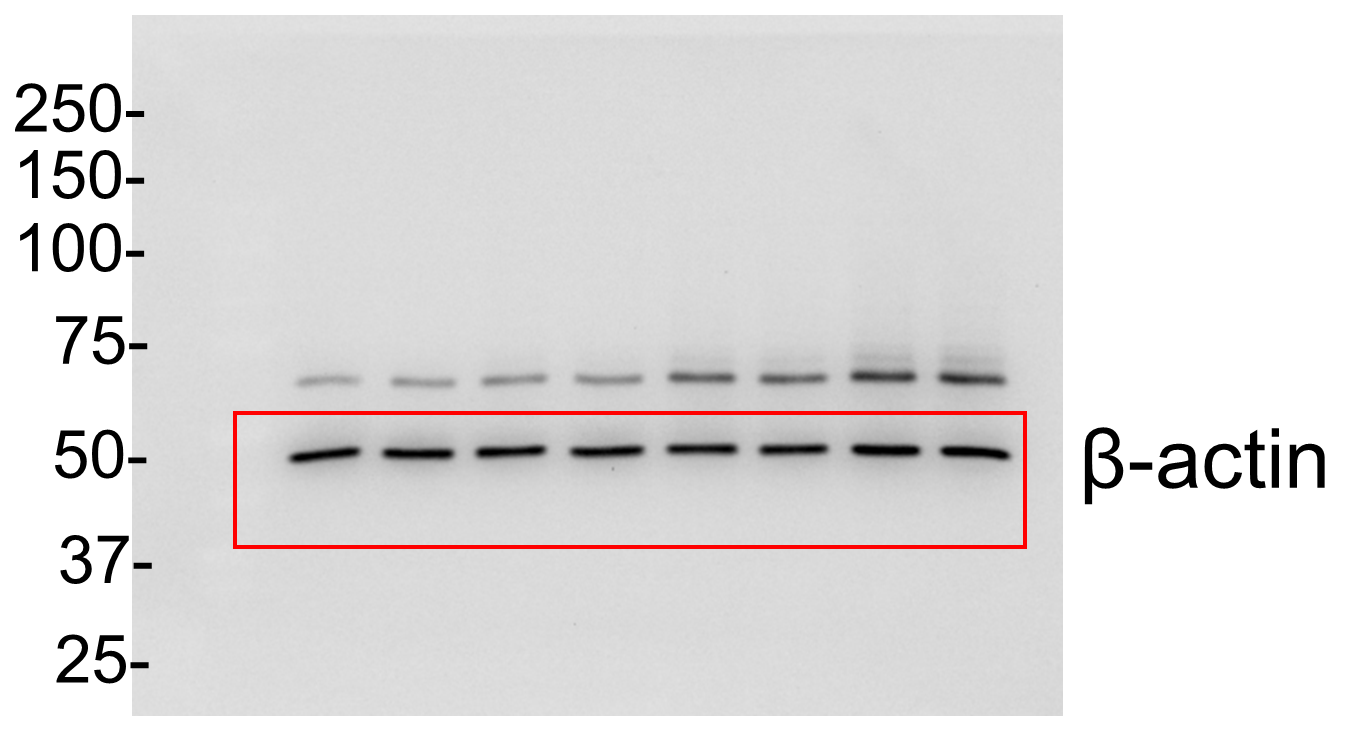

Supplement: Supplementary file 9 — Source Data for Figure 8 [file EMMM-15-e18242-s006.zip › Figure_8/8B/WB_beta-actin_R185S.tif]

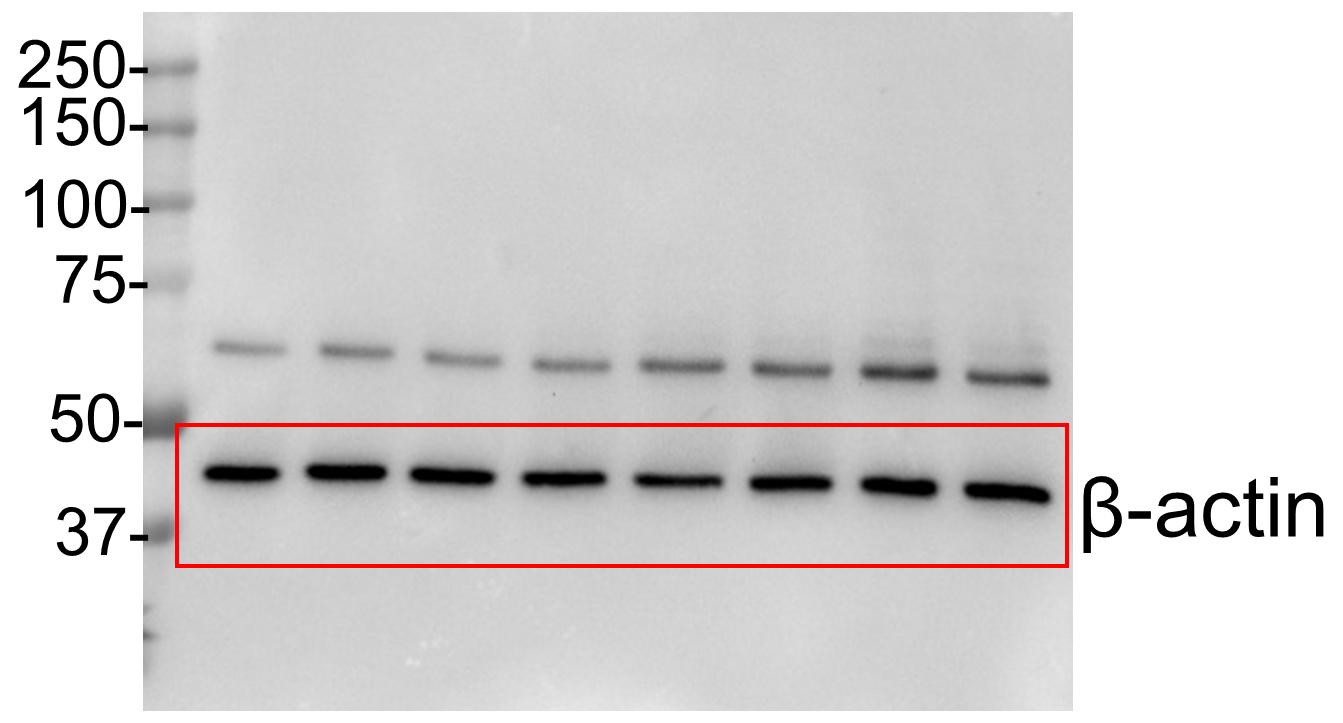

Supplement: Supplementary file 9 — Source Data for Figure 8 [file EMMM-15-e18242-s006.zip › Figure_8/8B/WB_beta-actin_WT.tif]

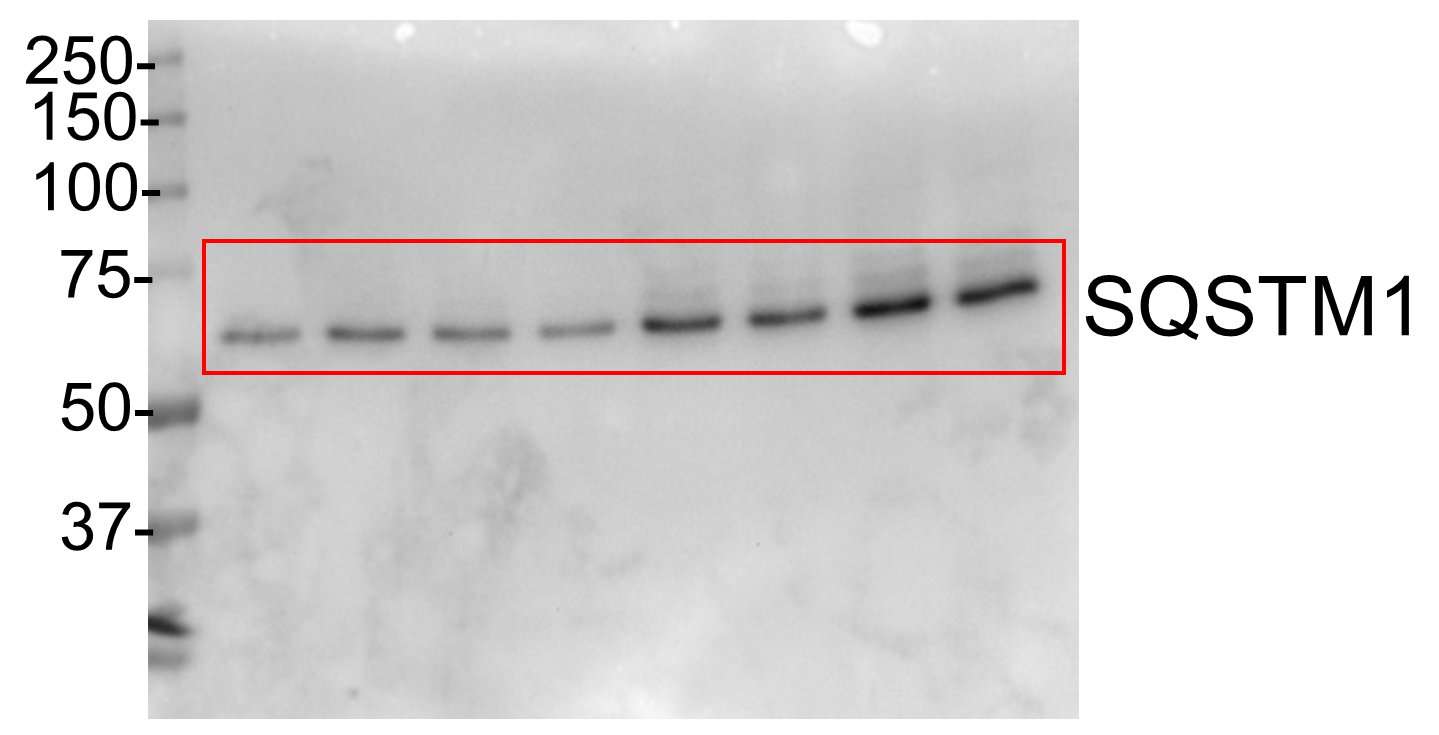

Supplement: Supplementary file 9 — Source Data for Figure 8 [file EMMM-15-e18242-s006.zip › Figure_8/8B/WB_SQSTM1_C170Y.tif]

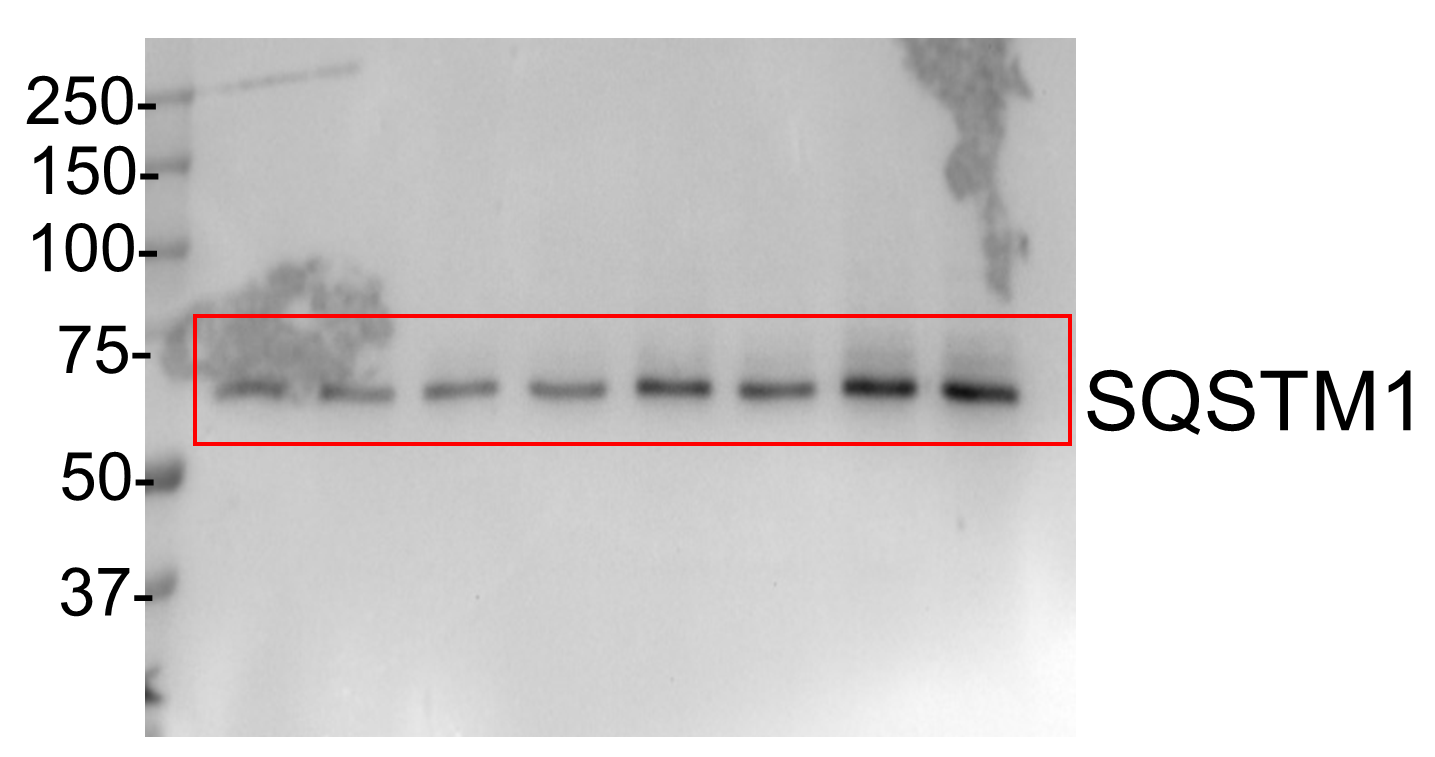

Supplement: Supplementary file 9 — Source Data for Figure 8 [file EMMM-15-e18242-s006.zip › Figure_8/8B/WB_SQSTM1_R185S.tif]

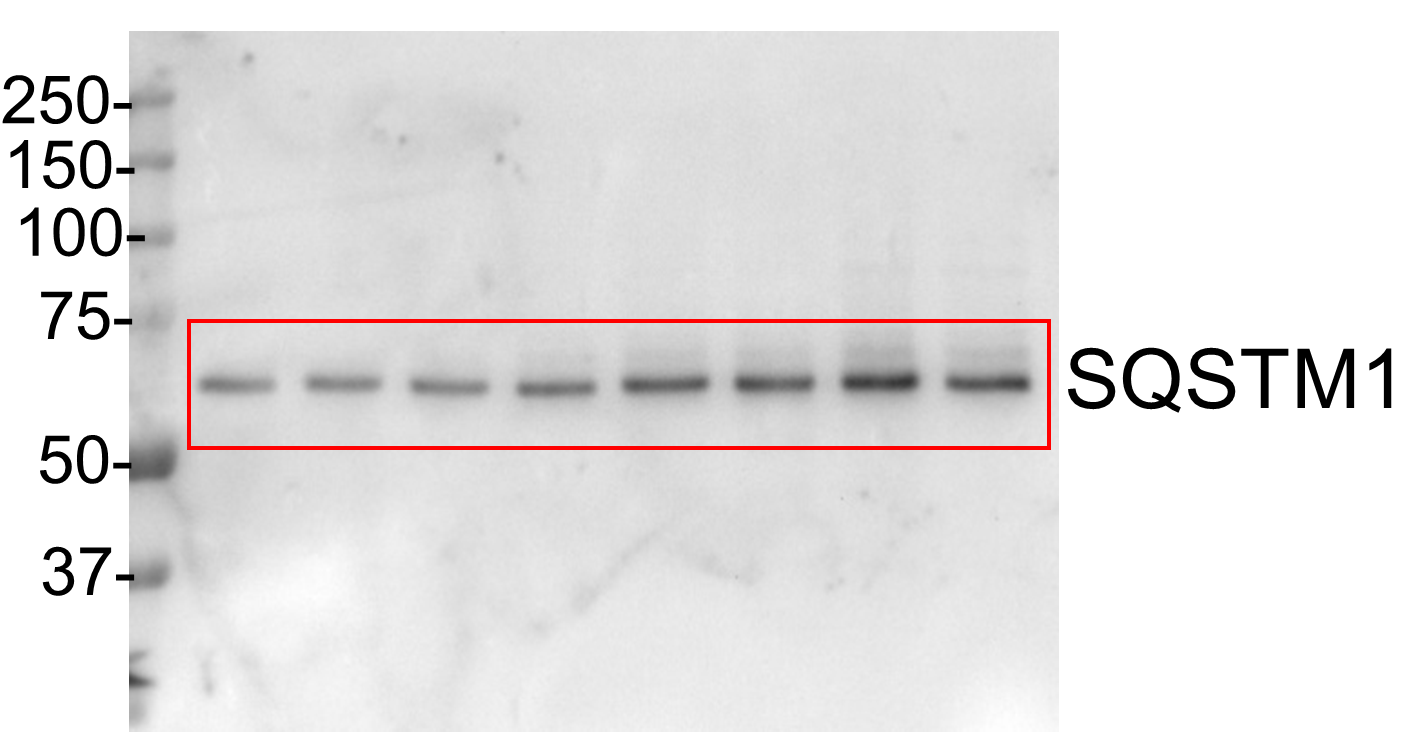

Supplement: Supplementary file 9 — Source Data for Figure 8 [file EMMM-15-e18242-s006.zip › Figure_8/8B/WB_SQSTM1_WT.tif]
