# Supplementary figures and images for: Allelic effects on uromodulin aggregates drive autosomal dominant tubulointerstitial kidney disease (part 2 of 2)
Source: EMBO Mol Med. 2023 Oct 26;15(12):e18242. doi: 10.15252/emmm.202318242 (PMC10701617; doi:10.15252/emmm.202318242)

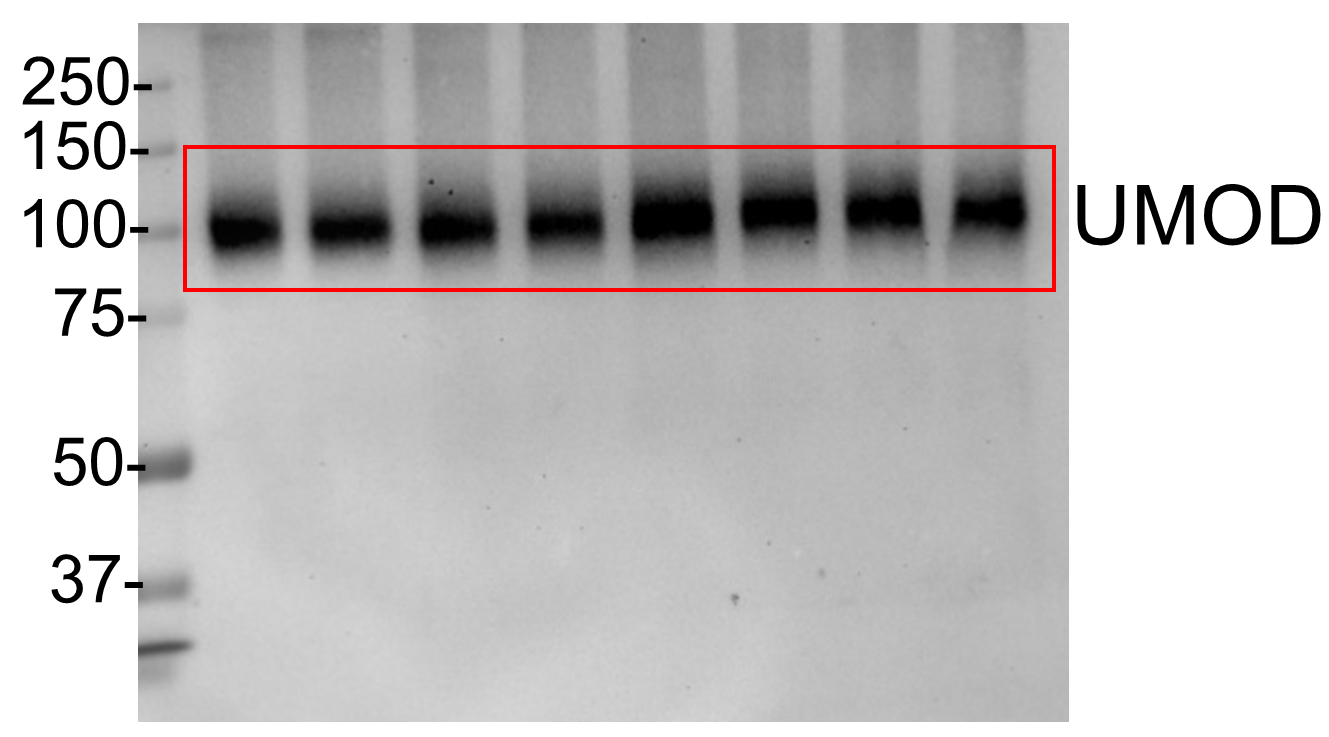

Supplement: Supplementary file 9 — Source Data for Figure 8 [file EMMM-15-e18242-s006.zip › Figure_8/8B/WB_UMOD_C170Y.tif]

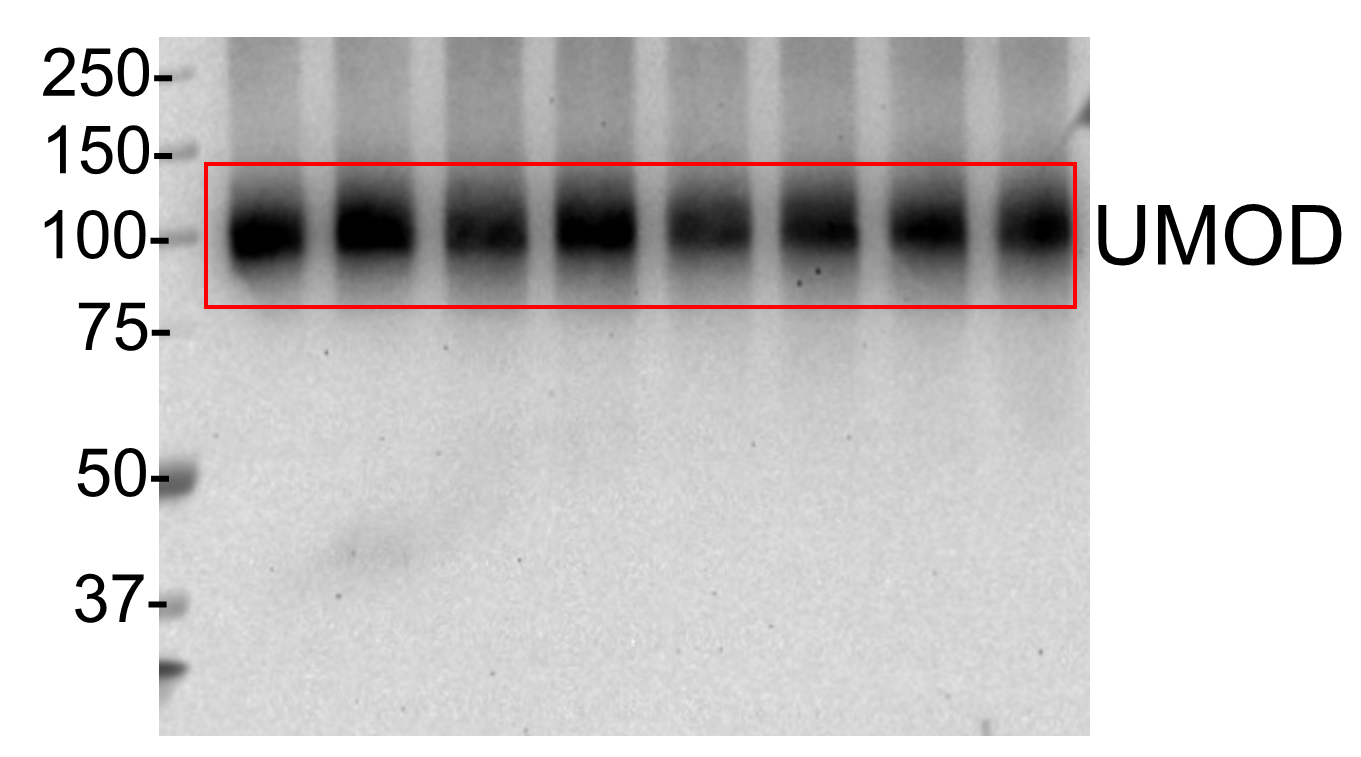

Supplement: Supplementary file 9 — Source Data for Figure 8 [file EMMM-15-e18242-s006.zip › Figure_8/8B/WB_UMOD_R185S.tif]

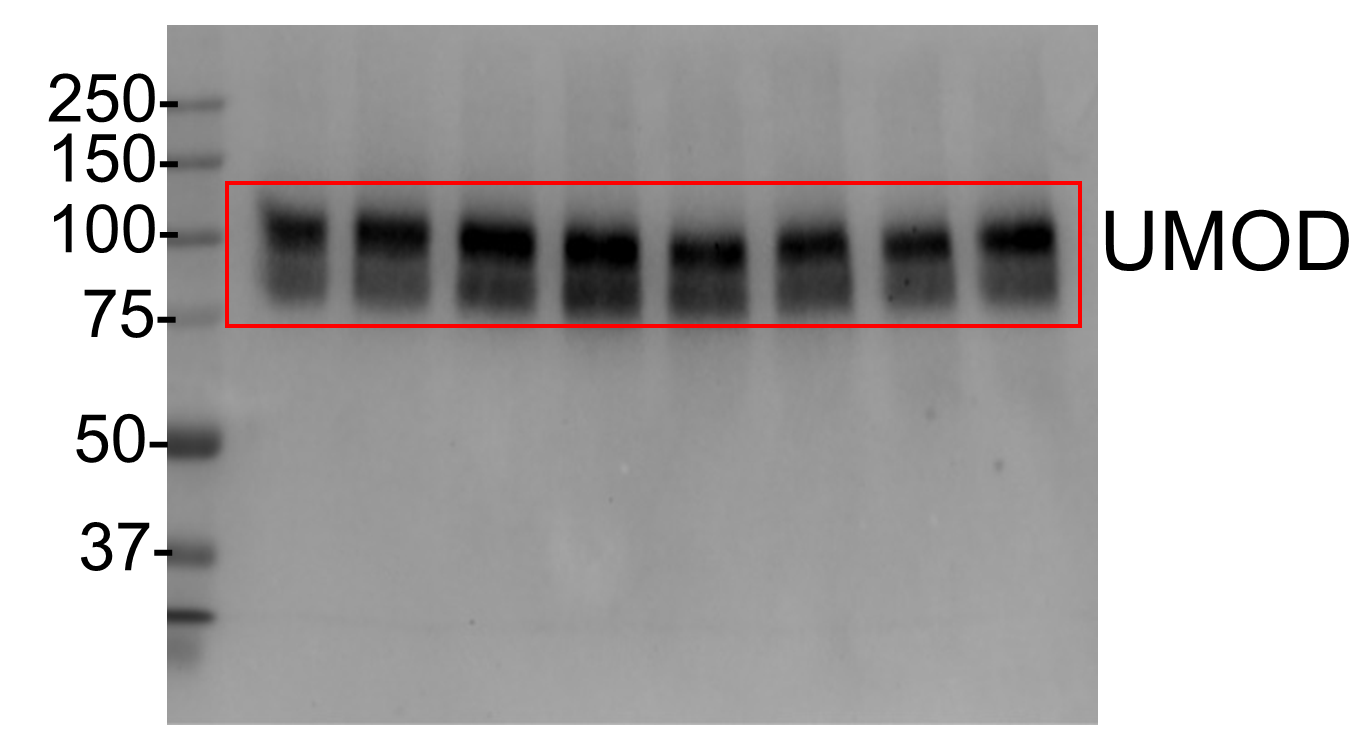

Supplement: Supplementary file 9 — Source Data for Figure 8 [file EMMM-15-e18242-s006.zip › Figure_8/8B/WB_UMOD_WT.tif]

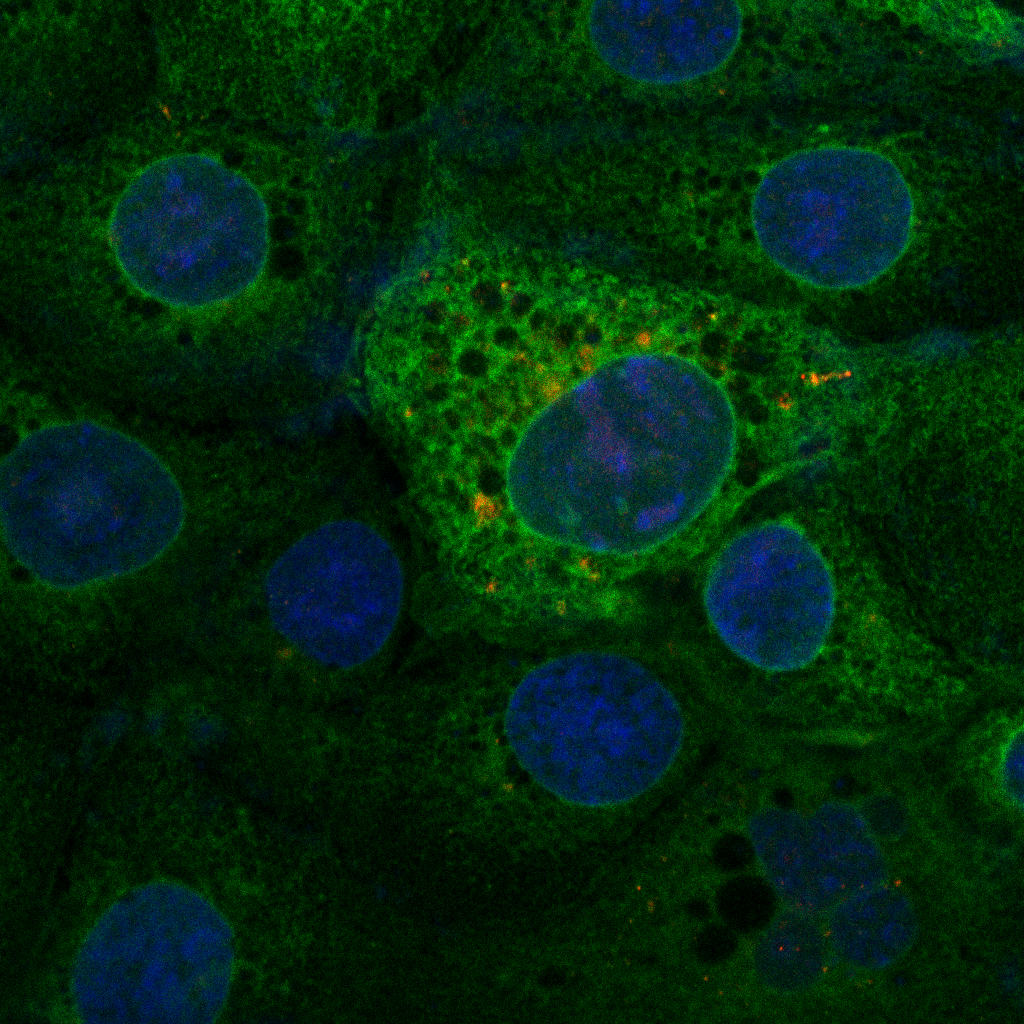

Supplement: Supplementary file 9 — Source Data for Figure 8 [file EMMM-15-e18242-s006.zip › Figure_8/8C/C170Y_FED_-_UMOD,_LC3.tif]

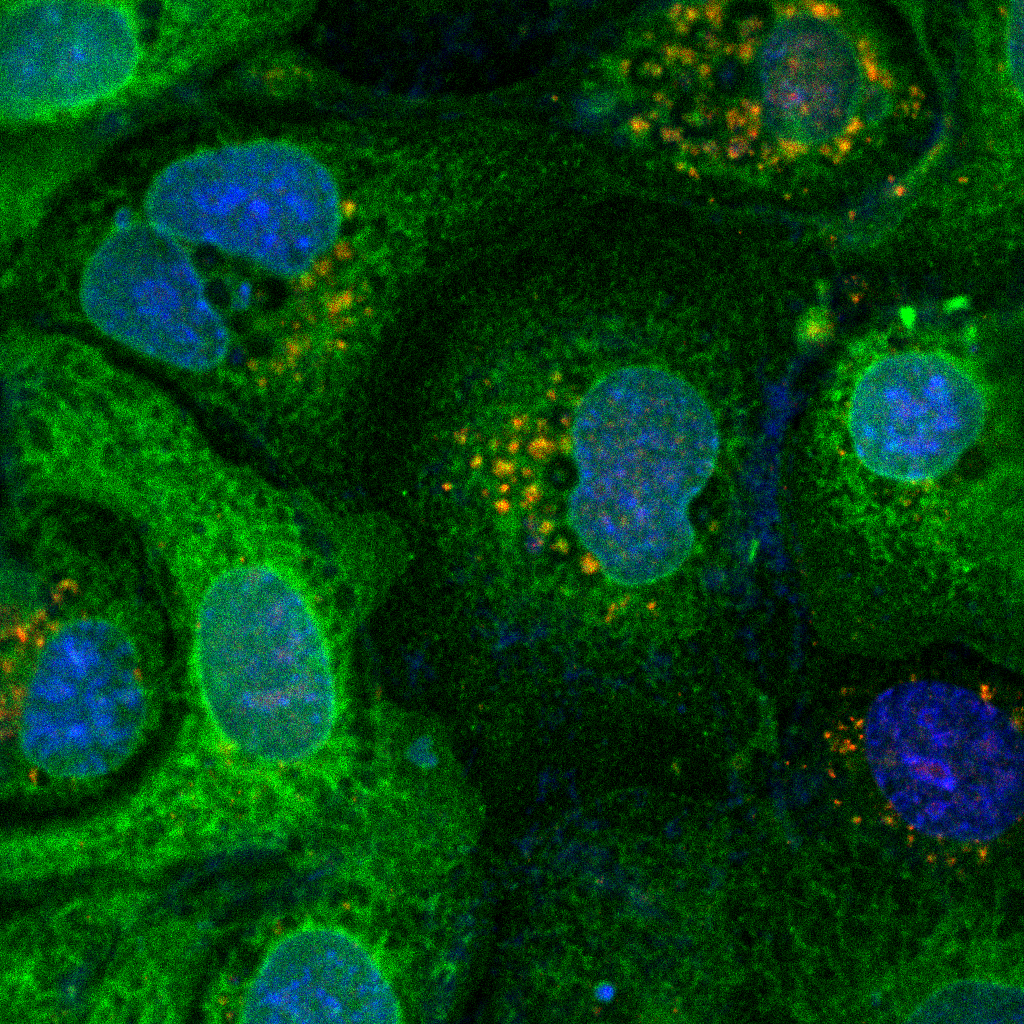

Supplement: Supplementary file 9 — Source Data for Figure 8 [file EMMM-15-e18242-s006.zip › Figure_8/8C/C170Y_STARV+BafA1_-_UMOD,_LC3.tif]

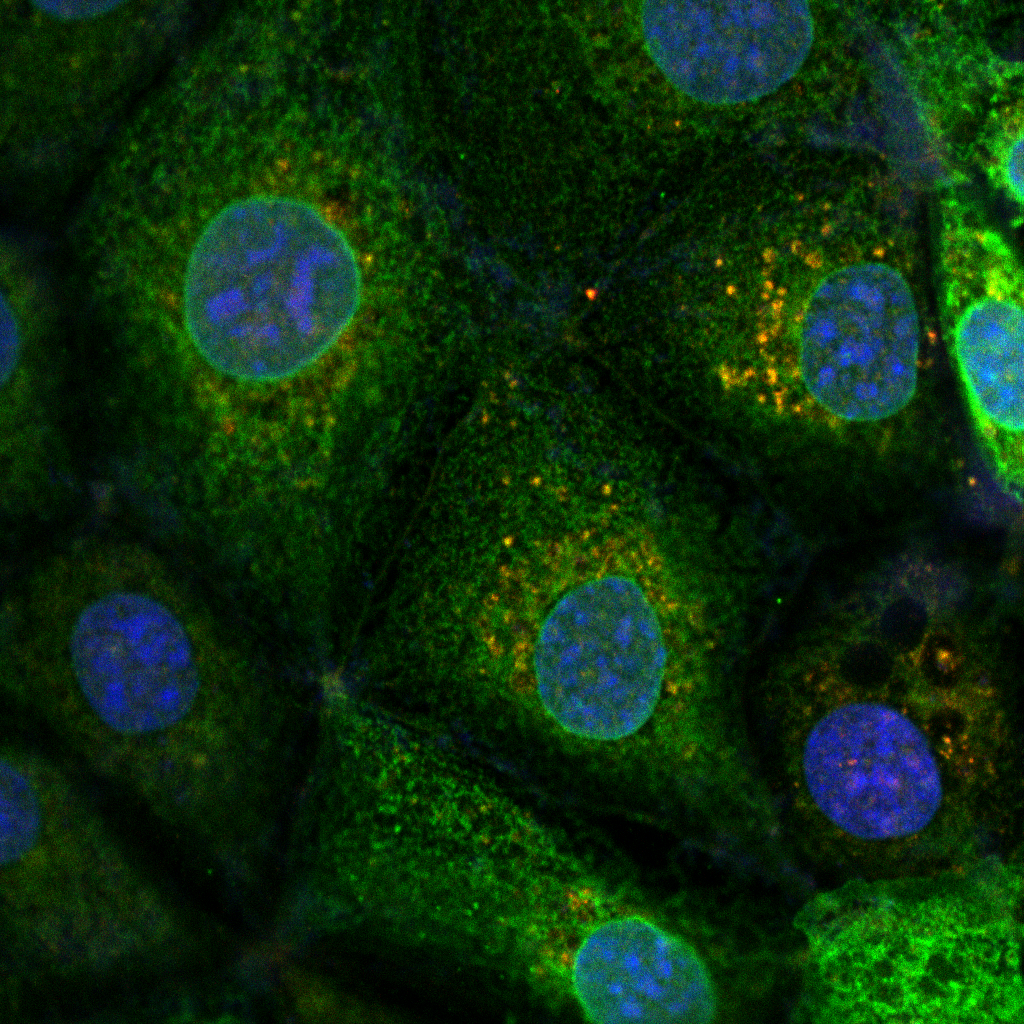

Supplement: Supplementary file 9 — Source Data for Figure 8 [file EMMM-15-e18242-s006.zip › Figure_8/8C/C170Y_STARV_-_UMOD,_LC3.tif]

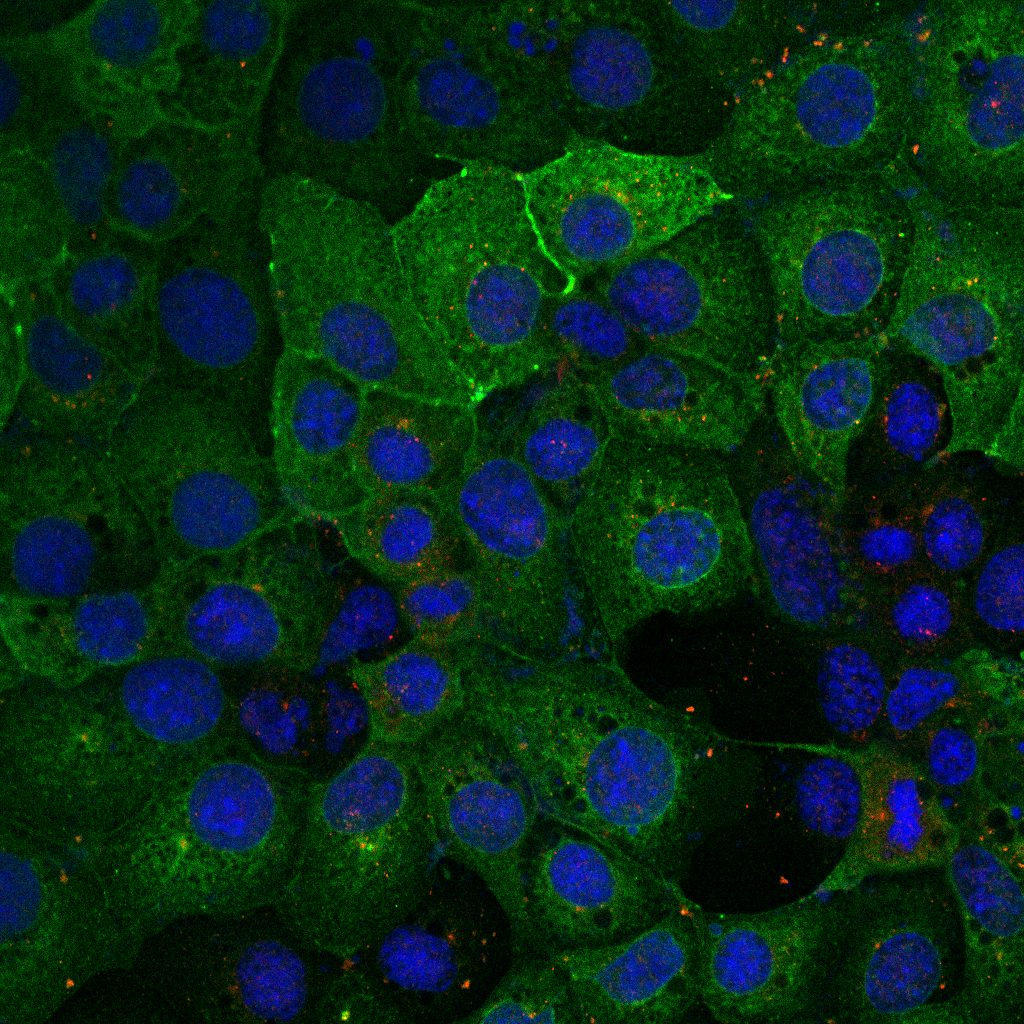

Supplement: Supplementary file 9 — Source Data for Figure 8 [file EMMM-15-e18242-s006.zip › Figure_8/8C/R185S_FED_-_UMOD,_LC3.tif]

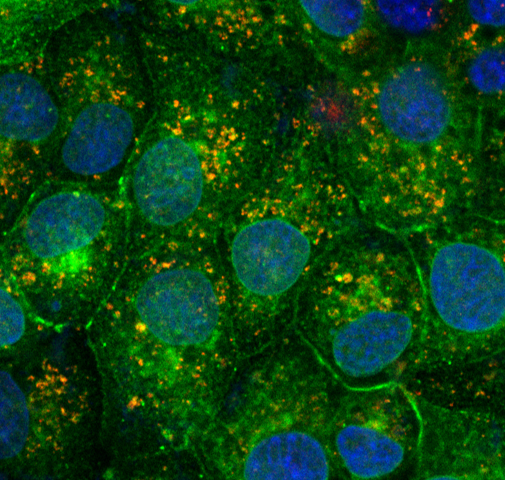

Supplement: Supplementary file 9 — Source Data for Figure 8 [file EMMM-15-e18242-s006.zip › Figure_8/8C/R185S_STARV_+_BafA1_-_UMOD,_LC3.tif]

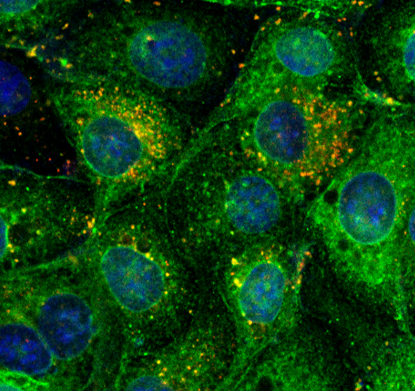

Supplement: Supplementary file 9 — Source Data for Figure 8 [file EMMM-15-e18242-s006.zip › Figure_8/8C/R185S_STARV_-_UMOD,_LC3.tif]

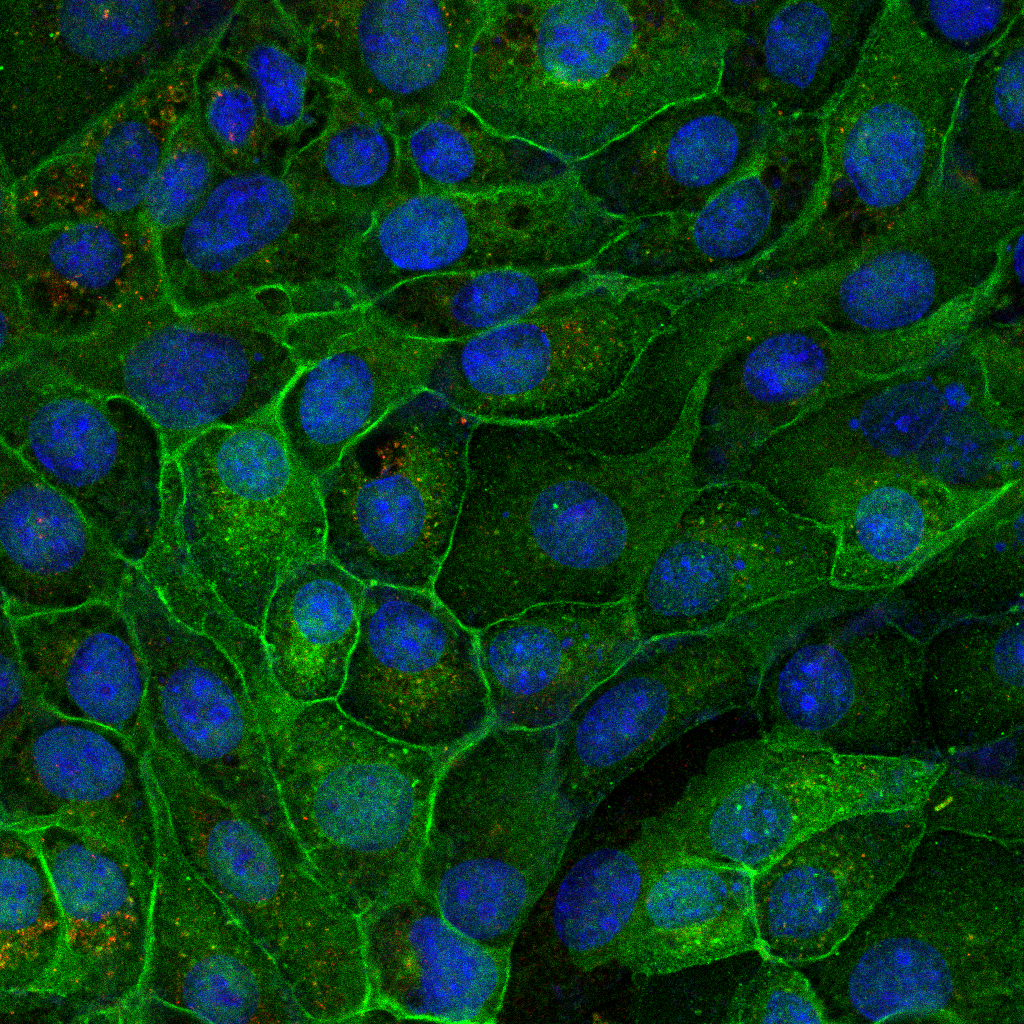

Supplement: Supplementary file 9 — Source Data for Figure 8 [file EMMM-15-e18242-s006.zip › Figure_8/8C/WT_FED_-_UMOD,_LC3.tif]

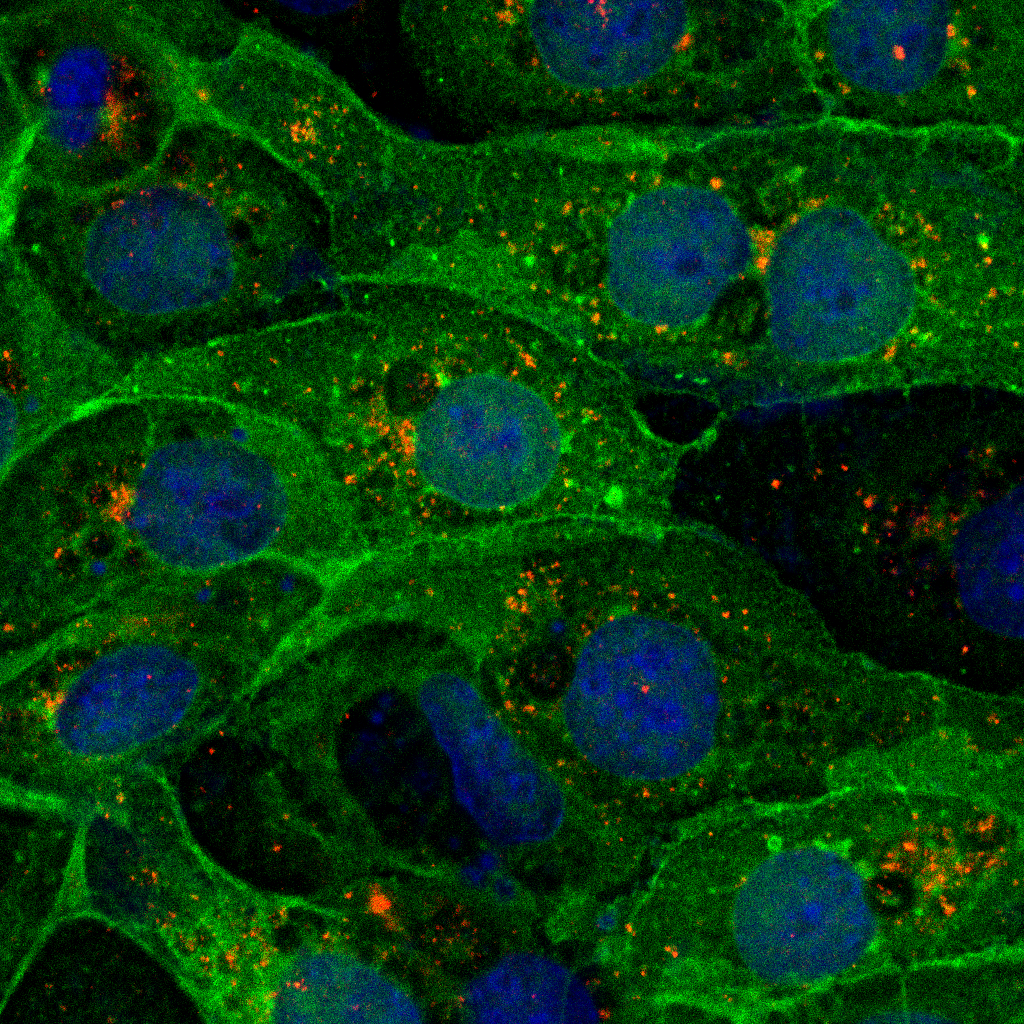

Supplement: Supplementary file 9 — Source Data for Figure 8 [file EMMM-15-e18242-s006.zip › Figure_8/8C/WT_STARV_+_BafA1_-_UMOD,_LC3.tif]

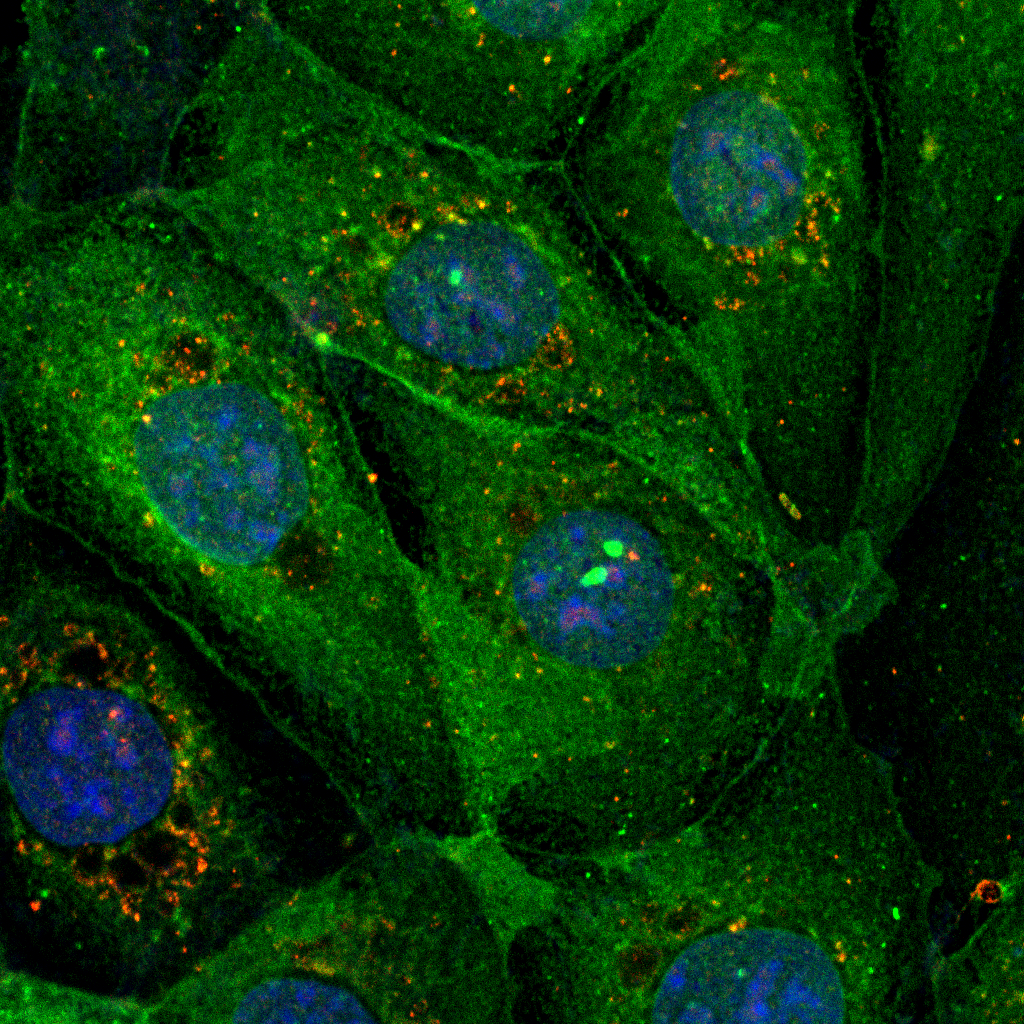

Supplement: Supplementary file 9 — Source Data for Figure 8 [file EMMM-15-e18242-s006.zip › Figure_8/8C/WT_STARV_-_UMOD,_LC3.tif]

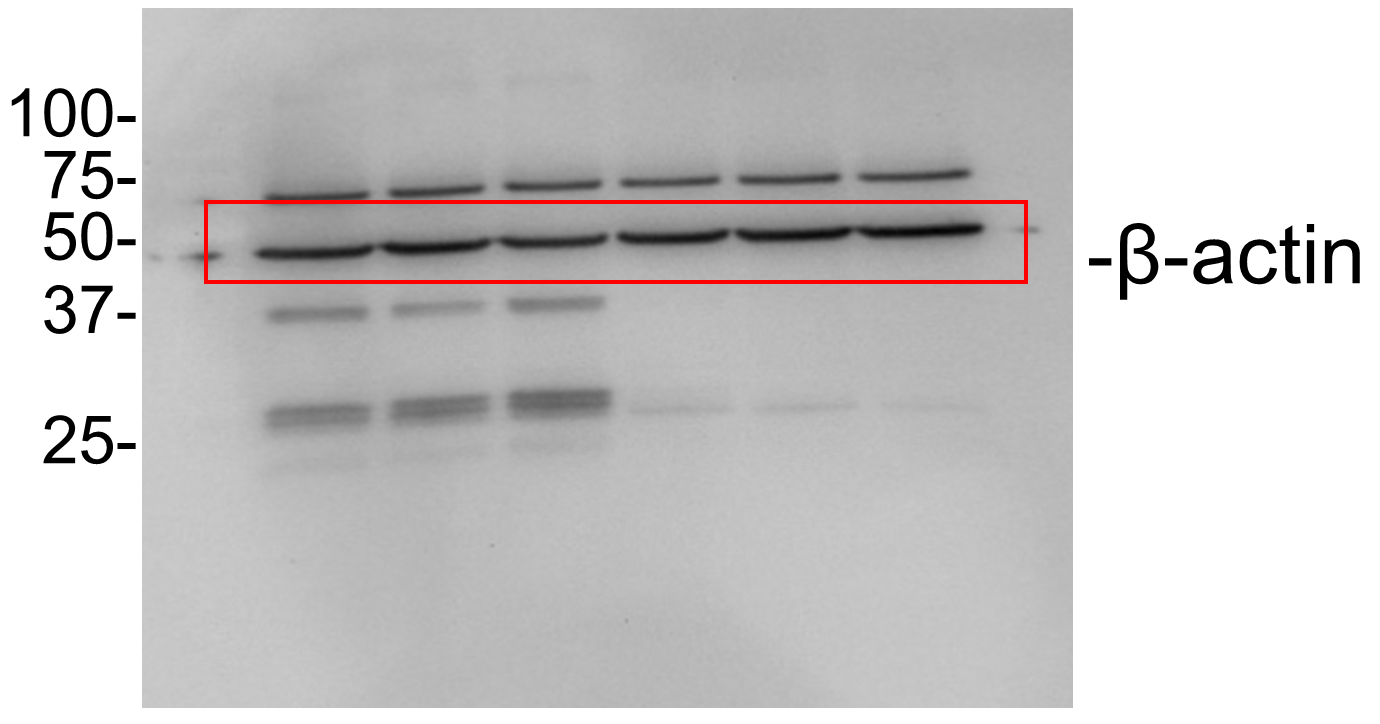

Supplement: Supplementary file 9 — Source Data for Figure 8 [file EMMM-15-e18242-s006.zip › Figure_8/8D/WB_beta-actin_C170Y.tif]

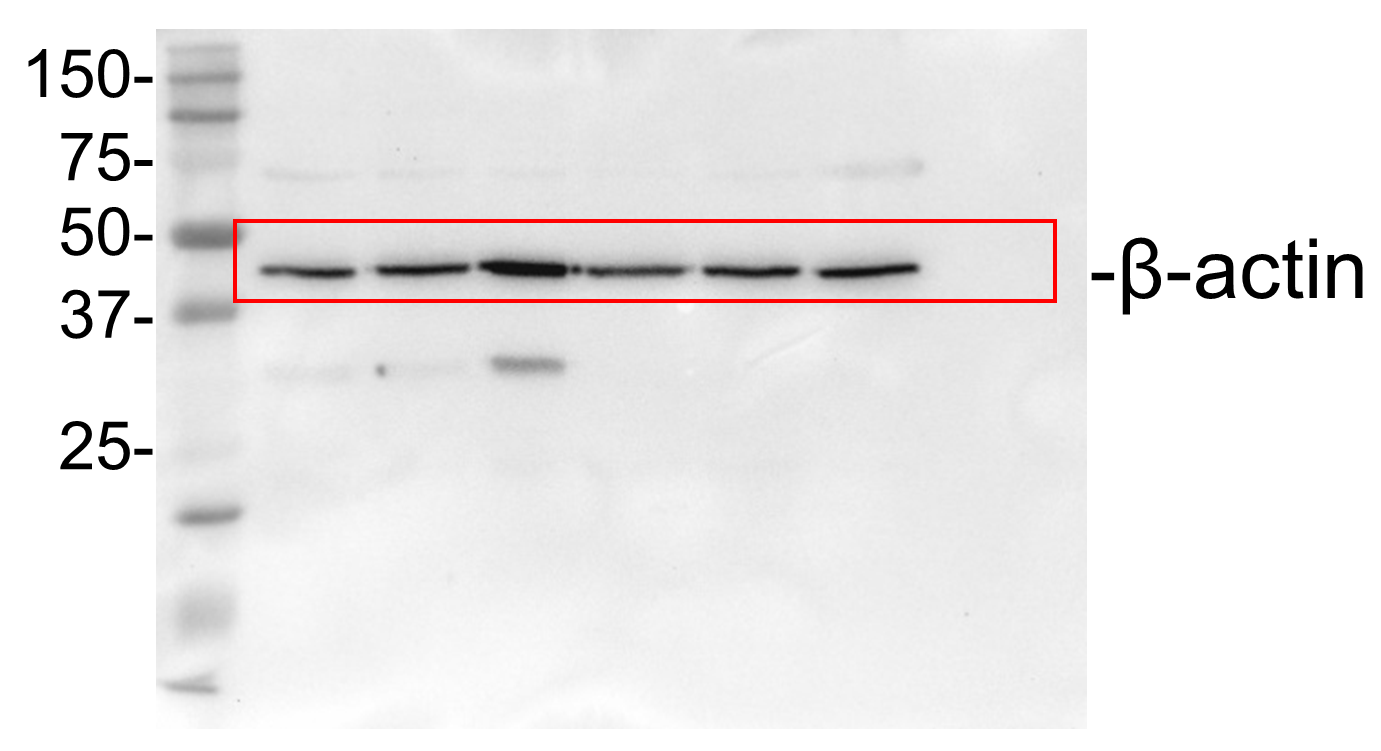

Supplement: Supplementary file 9 — Source Data for Figure 8 [file EMMM-15-e18242-s006.zip › Figure_8/8D/WB_beta-actin_R185S.tif]

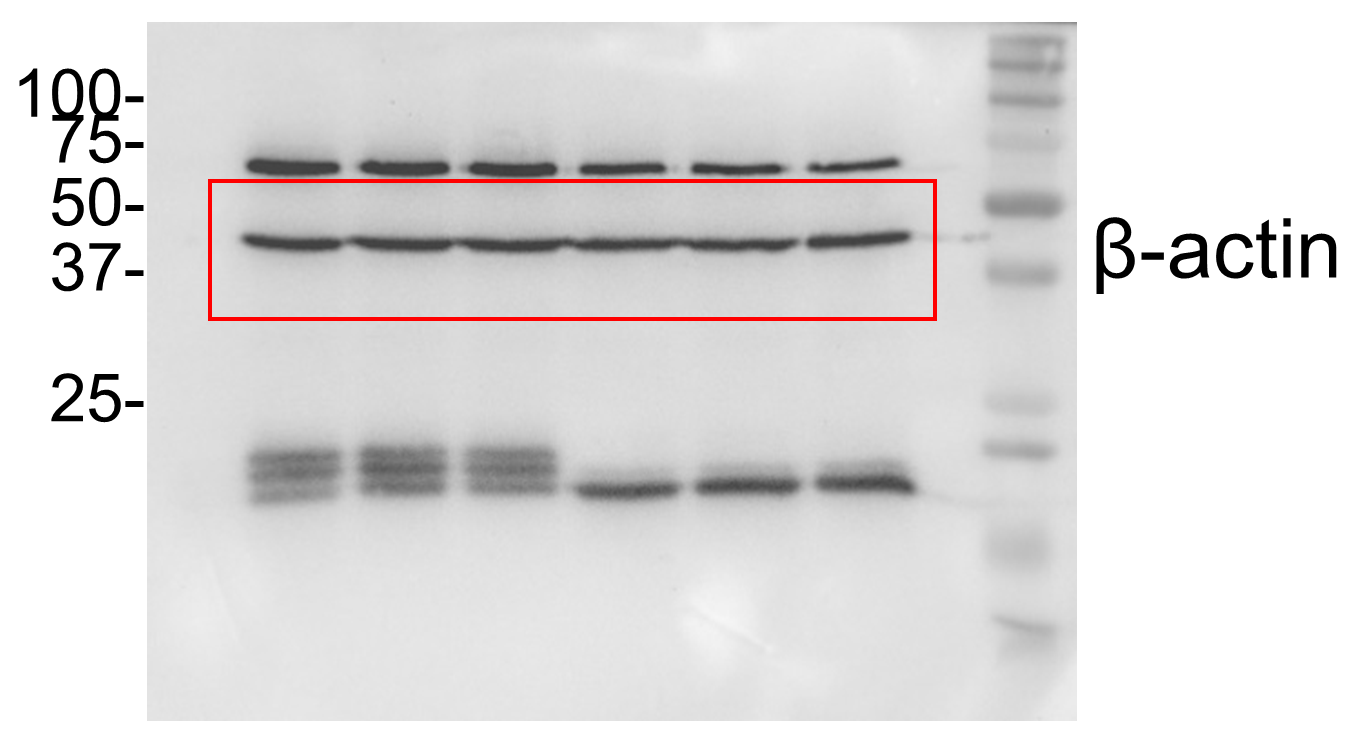

Supplement: Supplementary file 9 — Source Data for Figure 8 [file EMMM-15-e18242-s006.zip › Figure_8/8D/WB_beta-actin_WT.tif]

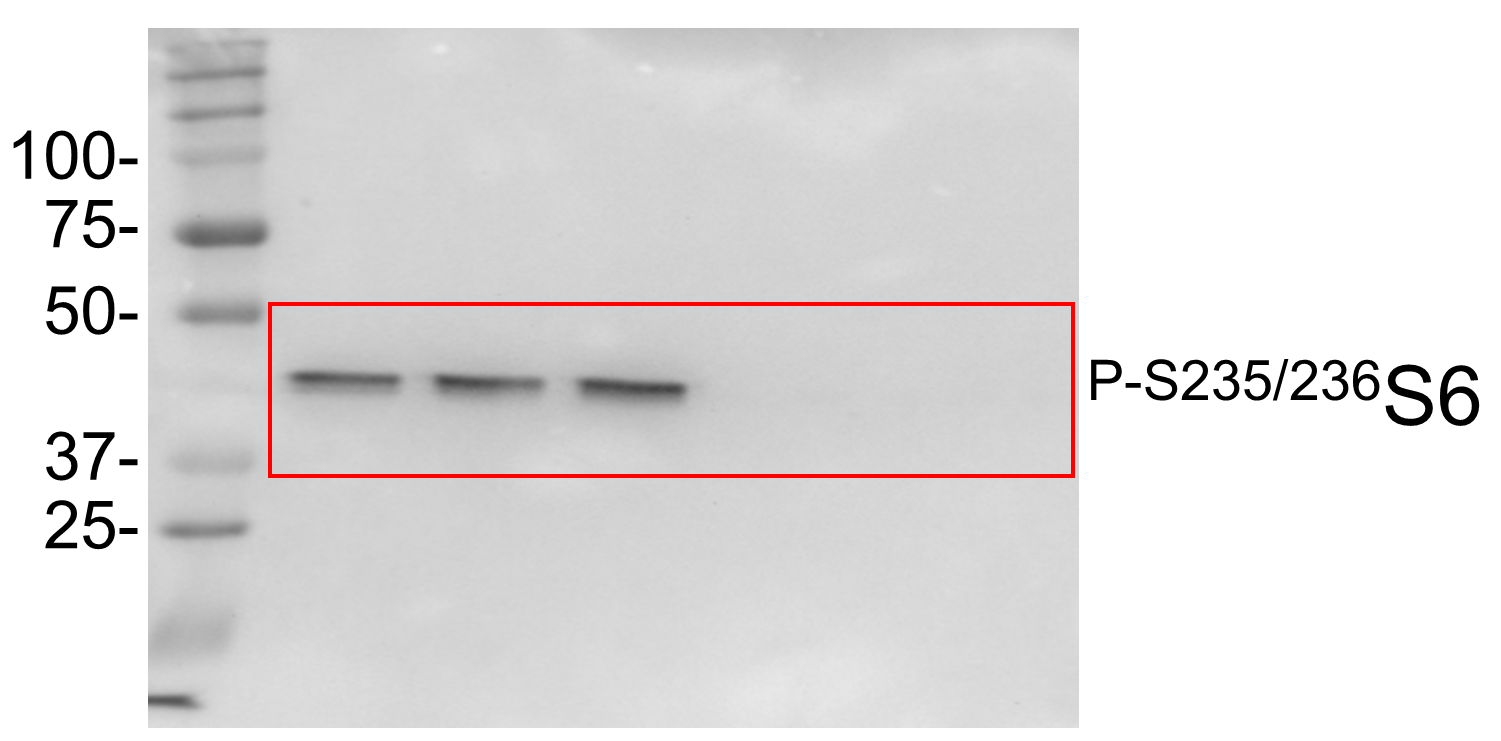

Supplement: Supplementary file 9 — Source Data for Figure 8 [file EMMM-15-e18242-s006.zip › Figure_8/8D/WB_P-S6_C170Y.tif]

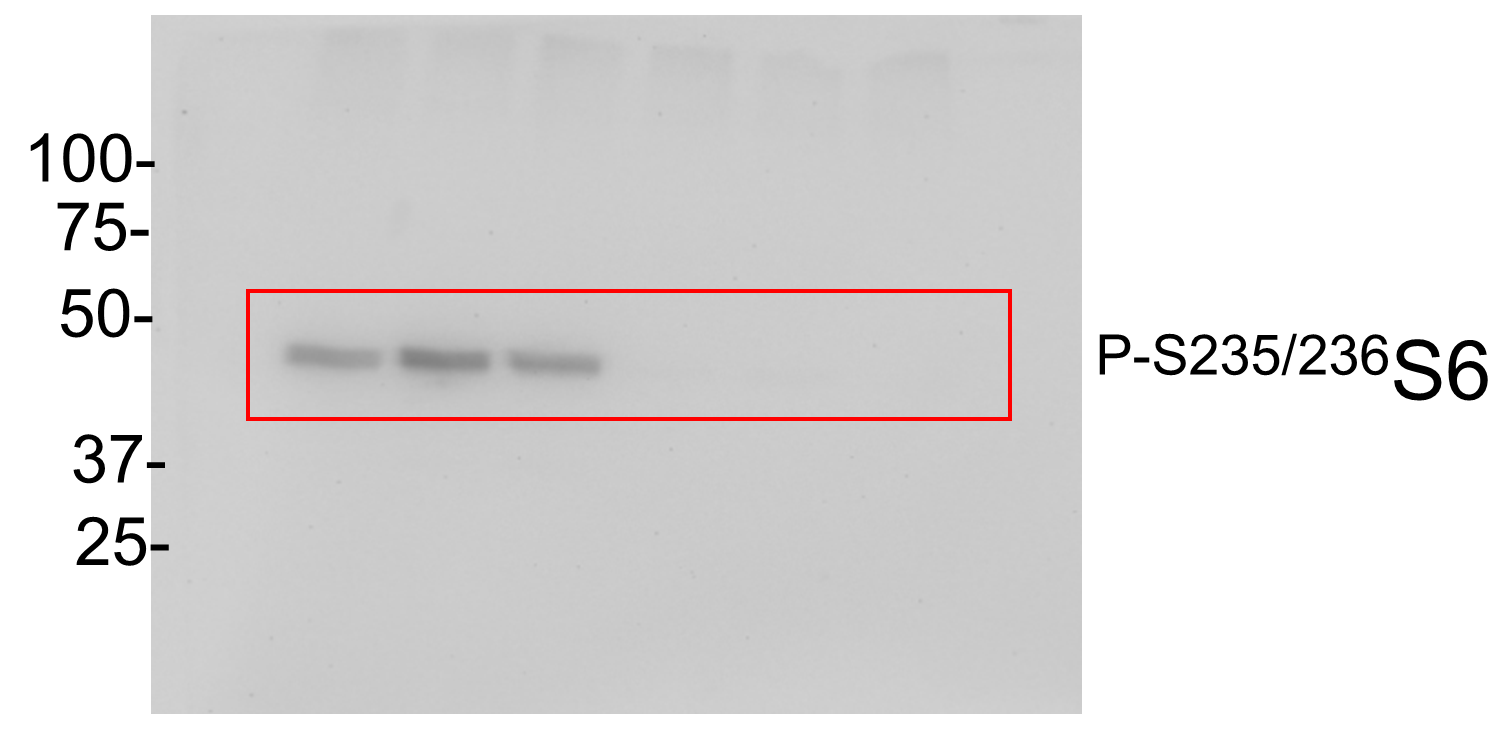

Supplement: Supplementary file 9 — Source Data for Figure 8 [file EMMM-15-e18242-s006.zip › Figure_8/8D/WB_P-S6_WT.tif]

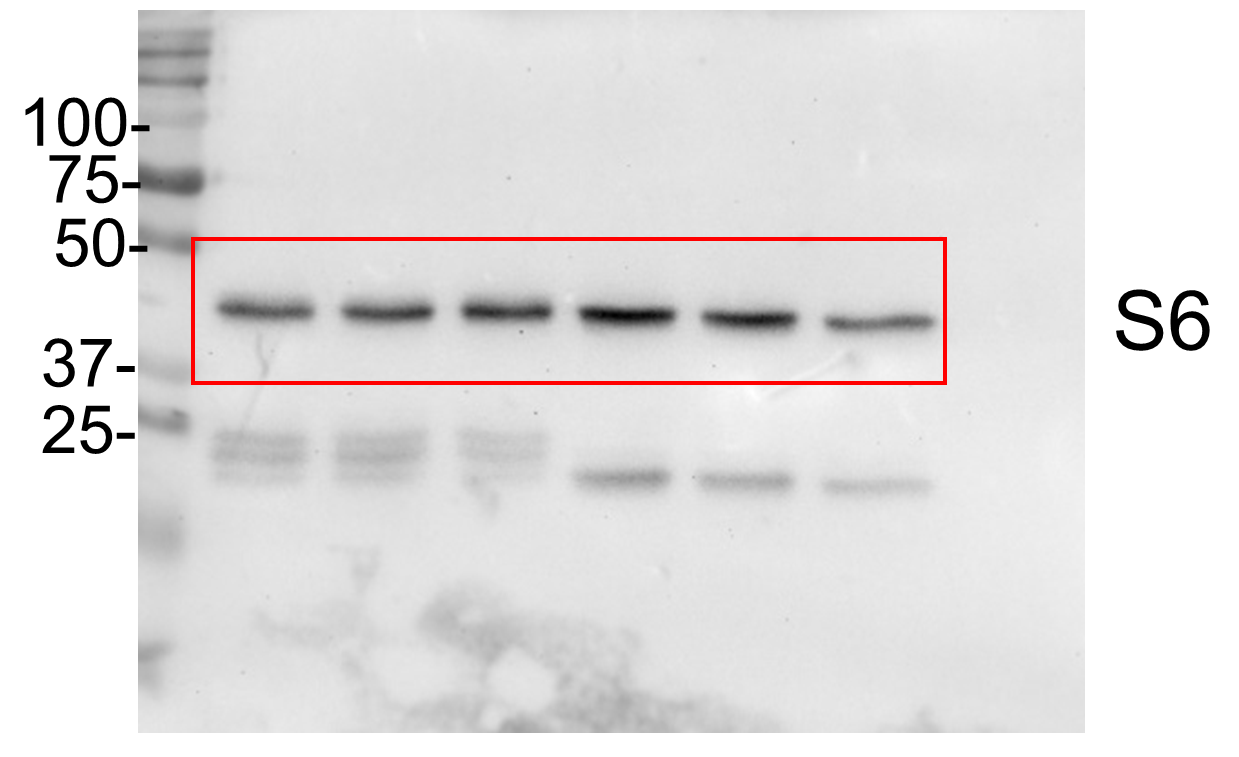

Supplement: Supplementary file 9 — Source Data for Figure 8 [file EMMM-15-e18242-s006.zip › Figure_8/8D/WB_S6_C170Y.tif]

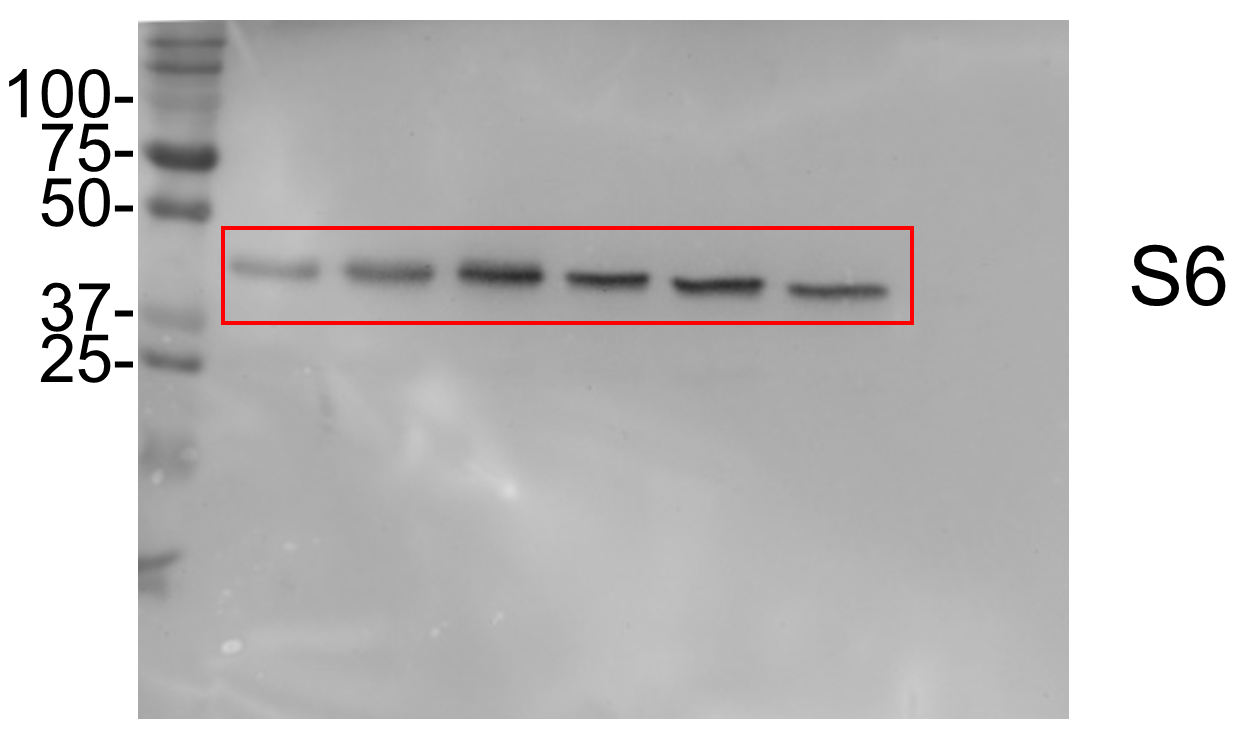

Supplement: Supplementary file 9 — Source Data for Figure 8 [file EMMM-15-e18242-s006.zip › Figure_8/8D/WB_S6_R185S.tif]

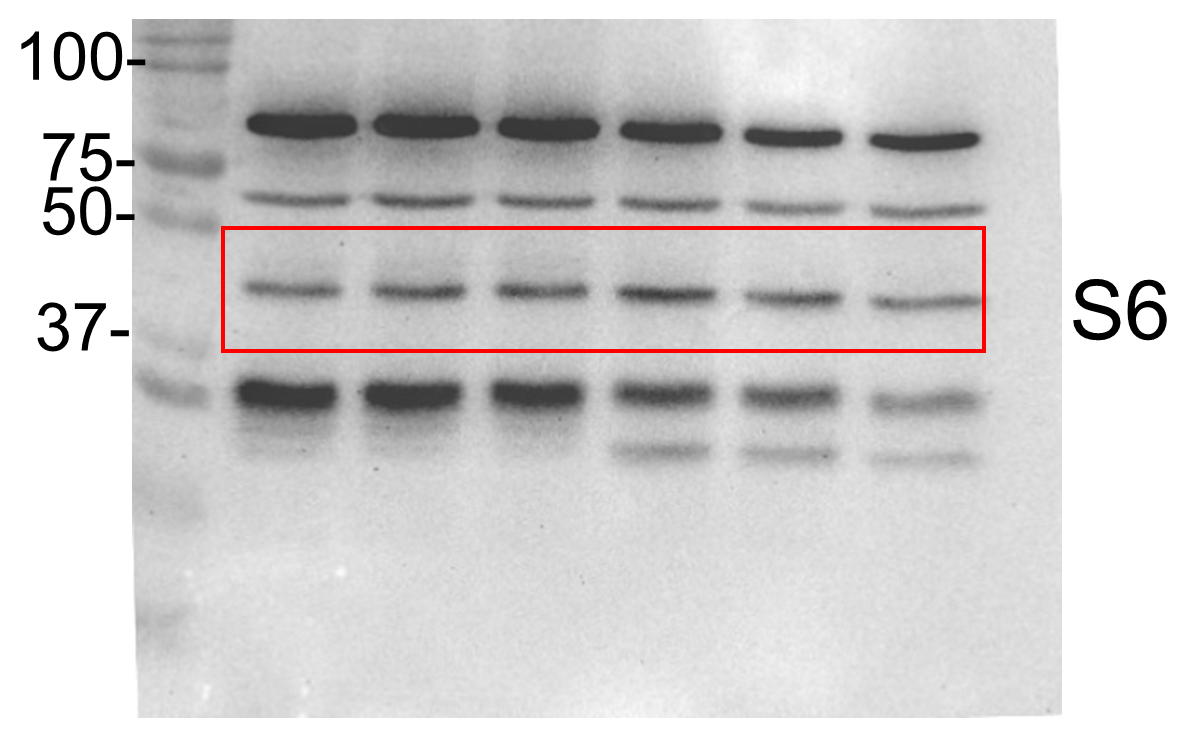

Supplement: Supplementary file 9 — Source Data for Figure 8 [file EMMM-15-e18242-s006.zip › Figure_8/8D/WB_S6_WT.tif]

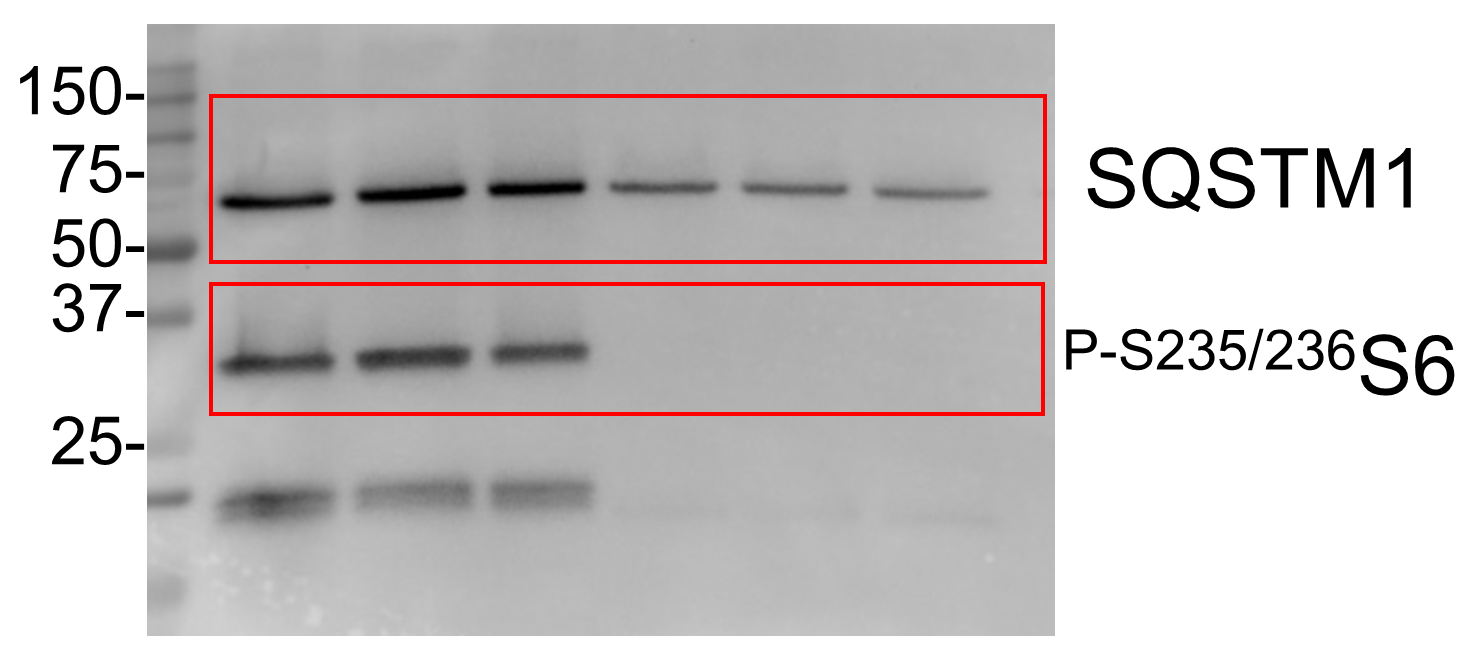

Supplement: Supplementary file 9 — Source Data for Figure 8 [file EMMM-15-e18242-s006.zip › Figure_8/8D/WB_SQSTM1,_P-S6_R185S.tif]

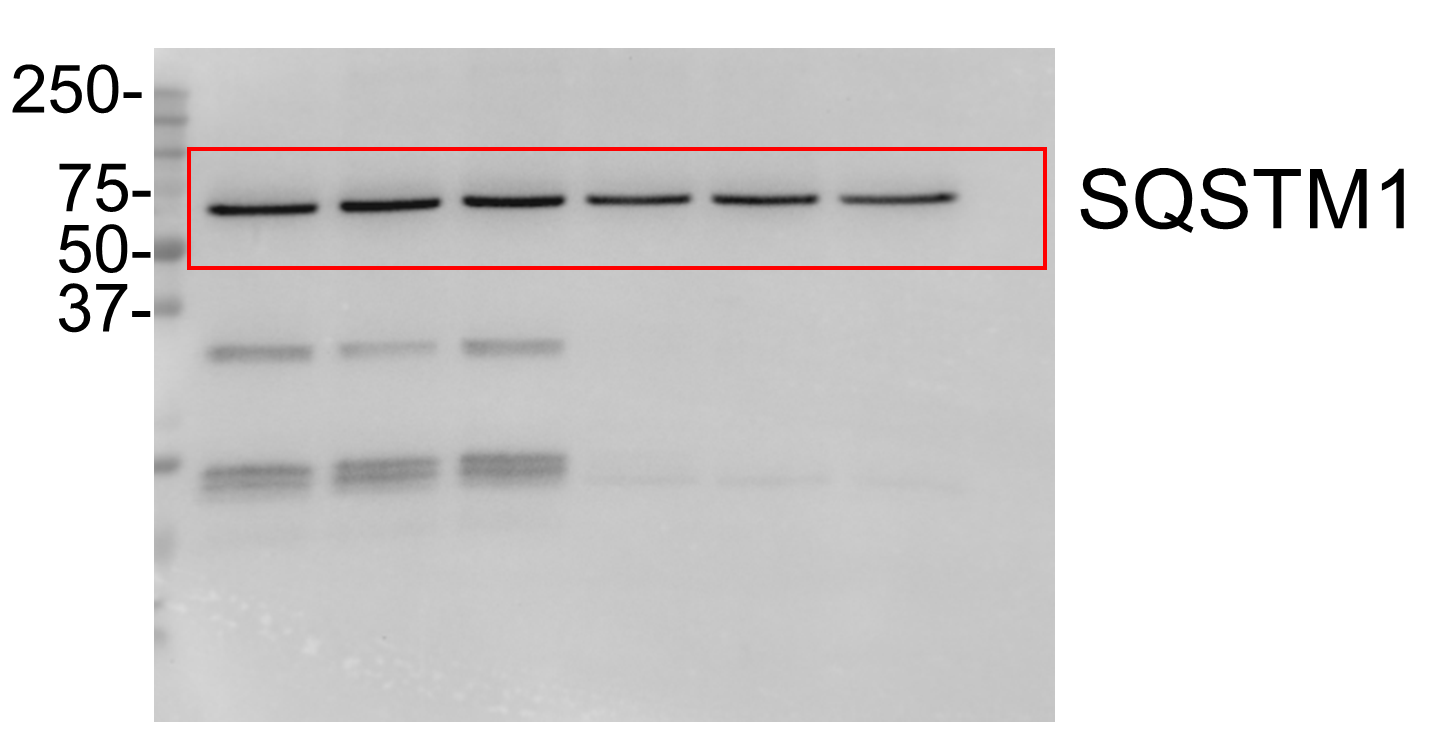

Supplement: Supplementary file 9 — Source Data for Figure 8 [file EMMM-15-e18242-s006.zip › Figure_8/8D/WB_SQSTM1_C170Y.tif]

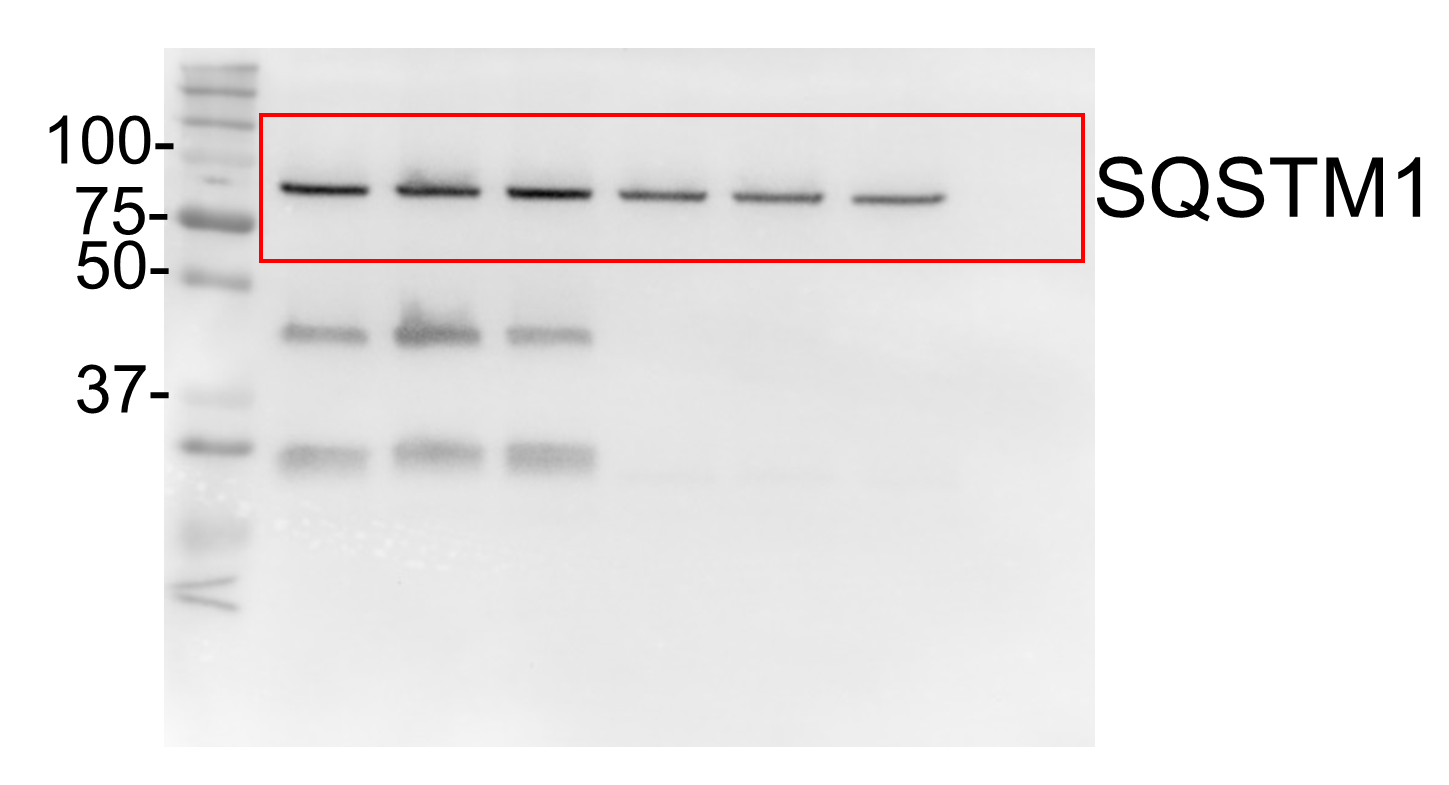

Supplement: Supplementary file 9 — Source Data for Figure 8 [file EMMM-15-e18242-s006.zip › Figure_8/8D/WB_SQSTM1_WT.tif]

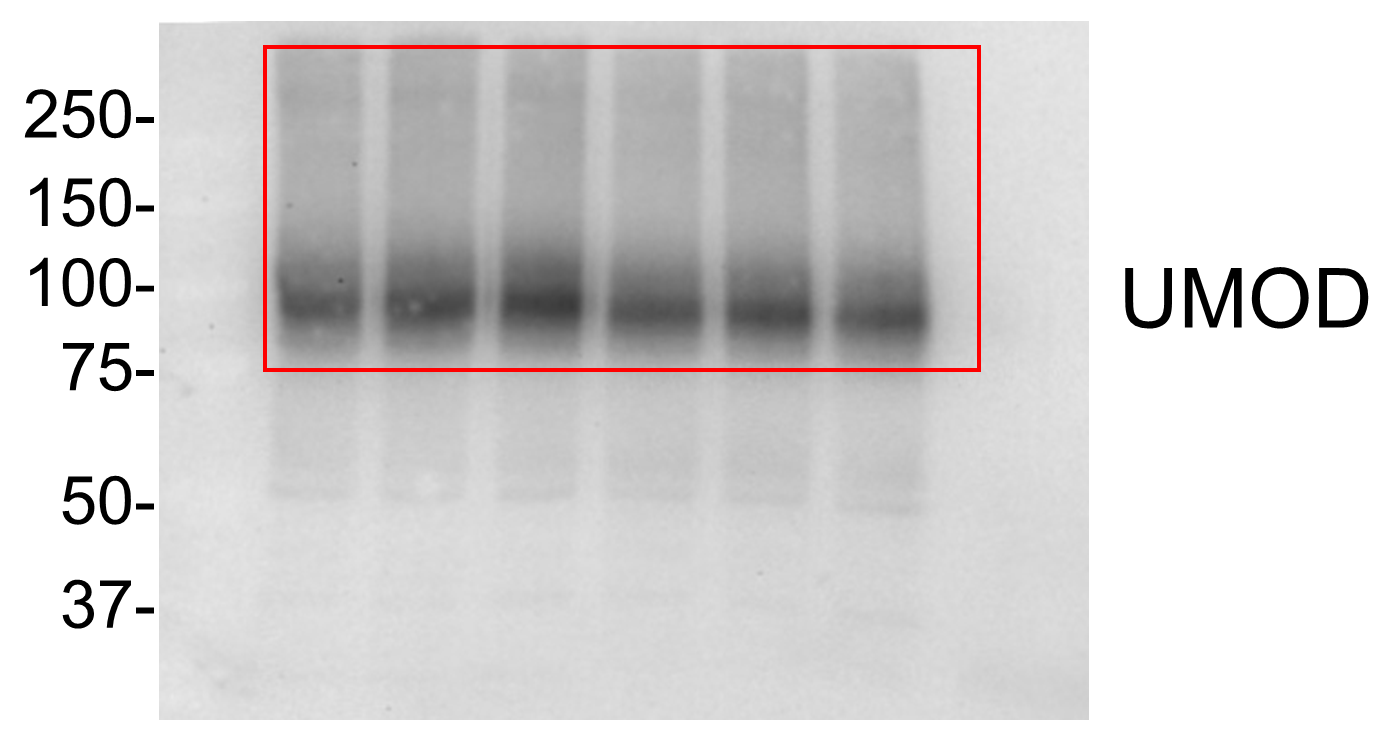

Supplement: Supplementary file 9 — Source Data for Figure 8 [file EMMM-15-e18242-s006.zip › Figure_8/8D/WB_UMOD_C170Y.tif]

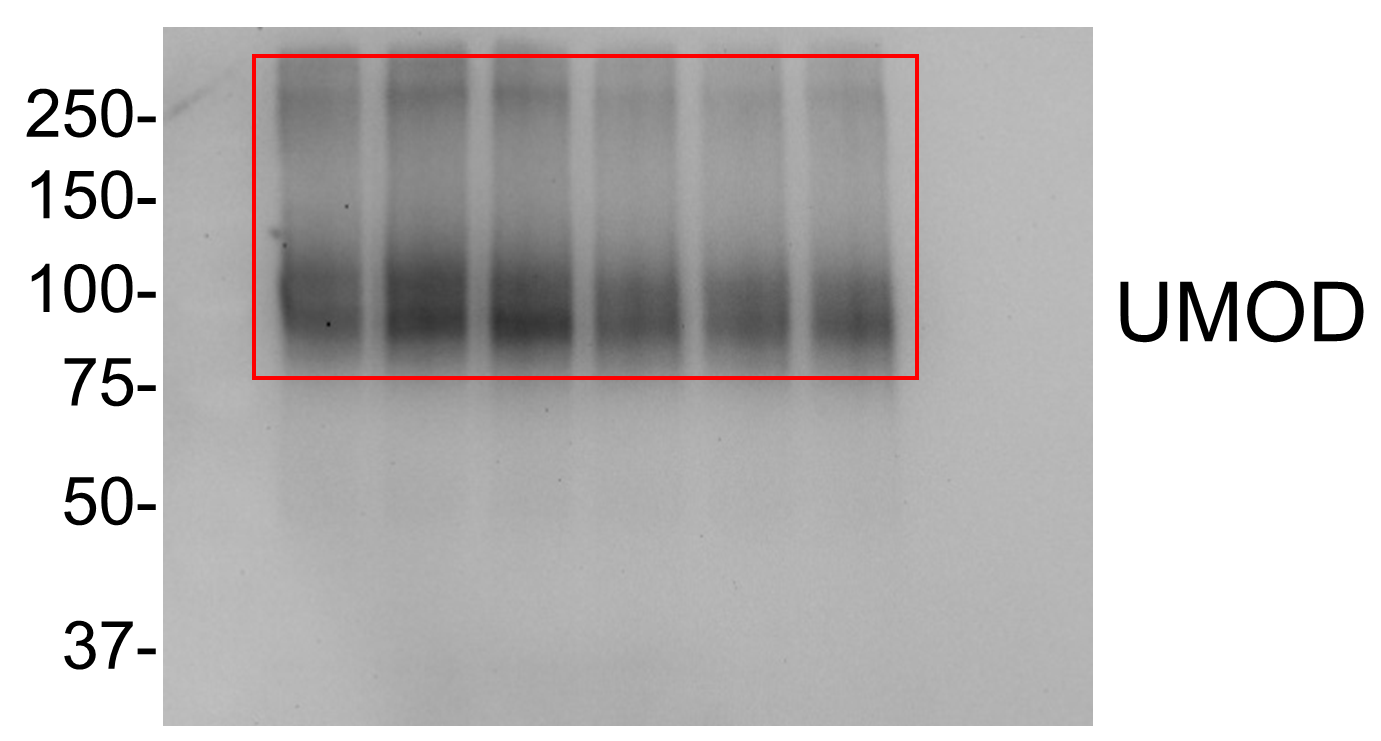

Supplement: Supplementary file 9 — Source Data for Figure 8 [file EMMM-15-e18242-s006.zip › Figure_8/8D/WB_UMOD_R185S.tif]

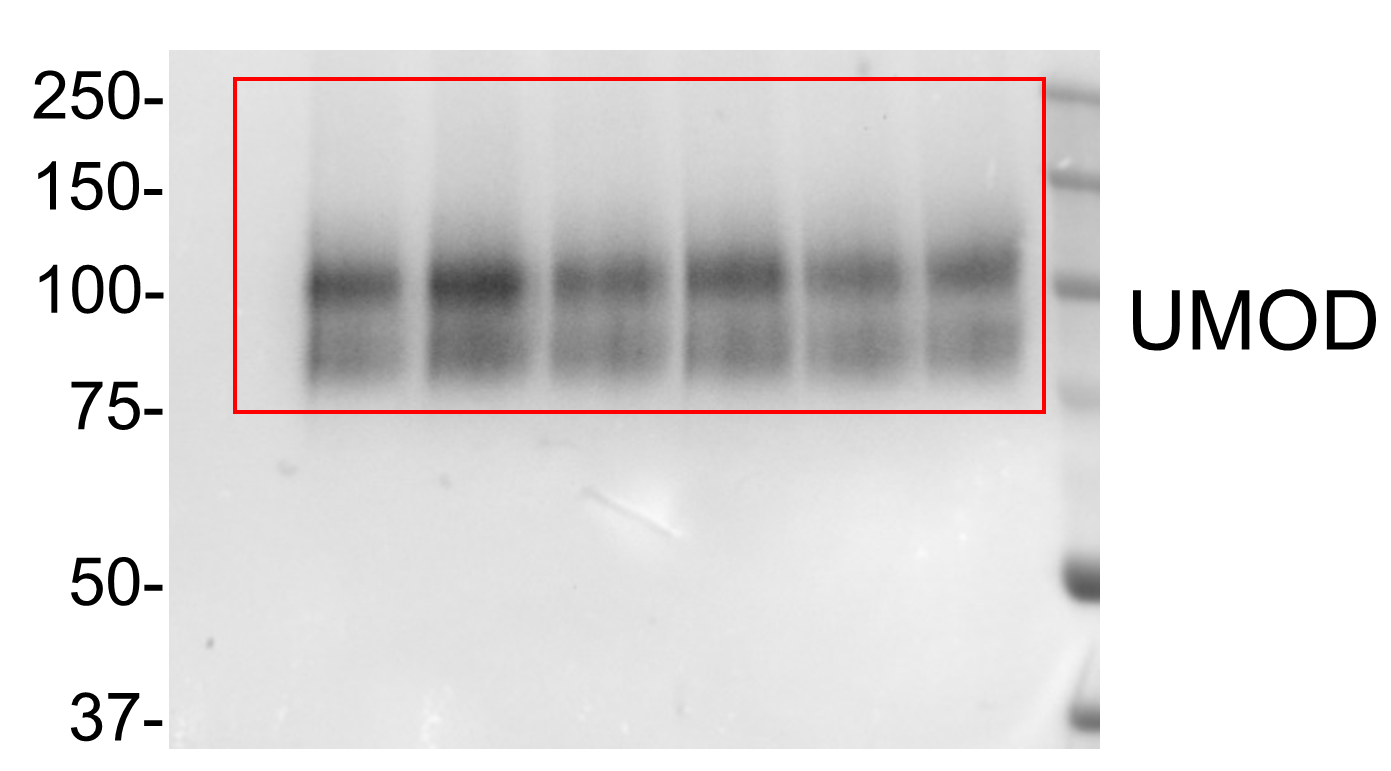

Supplement: Supplementary file 9 — Source Data for Figure 8 [file EMMM-15-e18242-s006.zip › Figure_8/8D/WB_UMOD_WT.tif]
