# Supplementary material for: Allelic effects on uromodulin aggregates drive autosomal dominant tubulointerstitial kidney disease
Source: EMBO Mol Med. 2023 Oct 26;15(12):e18242. doi: 10.15252/emmm.202318242 (PMC10701617; doi:10.15252/emmm.202318242)
Supplement: Supplementary file 10 [file EMMM-15-e18242-s002.pdf]

EMBO Molecular Medicine

Allelic Effects on Uromodulin Aggregates Drive Autosomal Dominant Tubulointerstitial Kidney Disease

Guglielmo Schiano, Jennifer Lake, Marta Mariniello, Céline Schaeffer, Marianne Harvent, Luca Rampoldi, Eric Olinger, and Oliver Devuyst
DOI: 10.15252/emmm.202318242

Corresponding author: Oliver Devuyst (olivier.devuyst@uzh.ch)

Review Timeline:

Transfer from Review Commons:	29th Jun 23
Editorial Decision:	7th Aug 23
Revision Received:	19th Sep 23
Editorial Decision:	22nd Sep 23
Revision Received:	29th Sep 23
Accepted:	5th Oct 23

Editor: Lise Roth

Transaction Report:

This manuscript was transferred to EMBO Molecular Medicine following peer review at Review Commons.

(Note: With the exception of the correction of typographical or spelling errors that could be a source of ambiguity, letters and reports are not edited. Depending on transfer agreements, referee reports obtained elsewhere may or may not be included in this compilation. Referee reports are anonymous unless the Referee chooses to sign their reports.)

Review #1

1. Evidence, reproducibility and clarity:

Evidence, reproducibility and clarity (Required)

Uromodulin (Tamm-Horsfall protein) is the most abundant protein excreted in human urine. It plays role in protection against urinary tract infections and renal stones. Mutations in UMOD gene encoding uromodulin cause Autosomal Dominant Tubulointerstitial Disease (ADTKD) that slowly progresses to chronic kidney disease.

In this manuscript, Schiano et al. isolate 12 missense UMOD mutations, which they classify into two groups by age occurrence. They then proceed to study two of these mutations: one from the earlier-onset - Arg185Ser - and the second from the later-onset - Cys170Tyr.

The authors generate UmodC171Y and UmodR186S knock-in mice with distinct dynamic pathways impacting on ADTKD progression. These mutations are equivalent with UMOD mutations (C170Y and R185S) in patients. UmodC171Y and UmodR186S knock-in mice show impaired uromodulin biogenesis, with strong allelic and gene-dosage effects. The trafficking problem of ADTKD-UMOD mutants, involving ER retention, ER stress, and activation of the UPR is recapitulated in mIMCD-3 cells, where the R185S mutant reveals more aggregates that are triggering PERK and IRE1 pathways and ER stress responses.

The manuscript is well written, experiments are in general well described and performed, results offer important insights on cellular events eventually leading to organ damage in ADTKD resulting from missense mutation in the UMOD gene.
The part of the work investigating the degradation mode of two different UMOD mutants, one relying on proteasomal and one relying on lysosomal clearance, is the most interesting for a general audience. Unfortunately, this last part of the work is too preliminary to be accepted as it is.

Comments/Suggestions:

- Selection of the UMOD variants, page 5: "R185S and C170Y are the most prevalent mutants in the clusters" please document/add reference.
- Fig. 1D: please show the position of the insets in the UMOD and BiP panels. Please separate the IF panels from the Picrosirius red panels (these are not the same samples that are shown),
Formally, the BiP panels in Fig. 1D reveal that there is more BiP in cells expressing R185S. That this correlates with UPR induction (as confirmed in Fig. x) should be written at the end of page 5 to make this issue clear for non-experts.
In Fig. 1D, the signal of BiP is not visible in WT and C170Y tissue/cells, which is odd because BiP is abundant protein. Moreover, the differences in BiP levels quantified in WB (semi-quantitative analyses) are not that dramatic in the mouse model (SFig. 3). Which panel in SFig. 3 (mouse) should be representative of the IF shown in Fig. 1D (patients)?
Fig. 1D: Magnification of these images is not sufficient to conclude that R185S accumulates in the ER, and that WT and C170Y are at the apical cell's membrane as written (page 5). Authors should refer to Suppl Fig 1C, where individual cells are visible.

Authors should briefly explain at the end of page 5 how the P. red staining in Fig. 1D informs on fibrosis.
- In the analyses of misfolded UMOD mutants (e.g., Fig. 2, 3, 4, ...) one would expect a test showing that BiP associates with R185S>C170Y>WT.
- Fig. 2F: in R186S there is a dramatic enlargement (at least 2x) of nuclei. Can the authors comment on that?
- Fig. 7E: Shouldn't one expects apical signal for C170Y?
- Fig. 7F: Why there is apical signal for R185S (and not for C170Y)?

- The part covering the degradation of the two UMOD variants would be of great interest for a wide audience of cell biologists. However, these data are too preliminary and, in this form, inconclusive.
Few examples: MG132 is a non-specific inhibitor of the proteasome, which may enhance endogenous and trans-gene expression (check in Pubmed "mg132 promoter" for relevant literature). Thus, an increase in the intracellular level of C170Y on MG132 treatment does not necessarily indicate inhibition of the protein's proteasomal turnover. It could also, at least in part, be caused by an increased synthesis of UMOD. The authors should show that MG132 does not increase synthesis of mutant UMOD (or use the more selective proteasome inhibitor PS-341 in their experiments); similarly, the data on R185S do not prove that this protein is client of autophagy. They rather show that autophagy removes the protein when cells are under nutrient restriction (note that starvation activates bulk autophagy, the non-selective lysosomal clearance of cellular components). To show that misfolded R185S is removed from cells by misfolded protein-induced ER-phagy (i.e., ER-to-lysosome-associated degradation), the authors should monitor in WB the accumulation of R185S in the presence of BafA1 and/or in IF the accumulation of R185S within lysosomes in the presence of BafA1.

Minor comments

- Figure 1B: dotted lines should be defined in the legend.
- Figure 1C: "phenotypes are denoted as indicated". The color-code used for the phenotype is unclear to me. For example, what is the phenotype of the V.2 (grey square)?
- The meaning of "Unlike in UMOD R185S cells, higher SQSTM1 puncta colocalizing with uromodulin were initially present in C170Y mutant cells and further accumulated in MG132-treated cells (Supplementary Figures 10A, B). These data suggest that mutant cells respond differently to UPS inhibition, with C170Y mutant uromodulin being mainly targeted to this pathway." (page 14) and the interpretation of the results shown in 10A and 10B is unclear to me.
- Page 7: "The UmodC171Y mice showed a progressive increase in BUN at 4 months" please define BUN.
- Please, provide a complete list of primary antibodies used for immunoblotting, immunohistochemistry, and immunofluorescence staining.

2. Significance:

Significance (Required)

The manuscript is well written, experiments are in general well described and performed, results offer important insights on cellular events eventually leading to organ damage in ADTKD resulting from missense mutation in the UMOD gene.
The part of the work investigating the degradation mode of two different UMOD mutants, one relying on proteasomal and one relying on lysosomal clearance, is the most interesting for a general audience. Unfortunately, this last part of the work is too preliminary to be accepted as it is.

My expertise: protein quality control, ER-phagy

3. How much time do you estimate the authors will need to complete the suggested revisions:

Estimated time to Complete Revisions (Required)

(Decision Recommendation)

Between 1 and 3 months

4. *Review Commons* values the work of reviewers and encourages them to get credit for their work. Select 'Yes' below to register your reviewing activity at Web of Science Reviewer Recognition Service (formerly Publons); note that the content of your review will not be visible on Web of Science.

Web of Science Reviewer Recognition

Yes

Review #2

1. Evidence, reproducibility and clarity:

Evidence, reproducibility and clarity (Required)

UAKD, a subtype of ADTKD, is extensively studied, although it is an rare inherit kidney disease. Using a knock-in strategy, the authors raised a novel concept that the differences in allelic and gene dosage of Umod mutation triggered distinct protein catabolic pathways, yielding distinct phenotypes and prognosis. The functional mechanisms include that UmodR186S mutation caused insoluble uromodulin aggregates resulting in activation of autophagy, and UmodC171Y mutation led to uromodulin misfolding and touched off ubiquitin-dependent ERAD pathway. Accordingly, the authors tested whether enhancing

autophagy attenuates the accumulation of UmodR186S protein in cell cultures. Based on these observations, the authors suggested a strategy to improve clearance of mutant uromodulin. This study was carried out by a team with strong reputation in this area. However, the story appears to be incomplete and in vivo testing of their therapeutic strategy is needed to improve this research.

Specific comments

1. Figure 1D: Images at low magnification do not show DAPI, therefore there is no information on the total number of cells in the selected field. Nephron loss (represented by glomeruli) did not appear to differ between UMOD p C170Y and UMOD p R185S, which is inconsistent with the overall conclusions. In addition, PAS staining should be added in Figure 1D.
2. Figure 2E: in image of C171Y/+, this is no corresponding tubules which is represented by the insert. Figure 2F lower panel, the bars in EM fields are same, indicating a hypertrophy of nuclei in R186S? Figure 2G: how about serum creatinine in these mice? In addition, signs of catabolism (e.g., loss of body weight) are associated with these KI mice?
3. Figure 3C: what is rationale of using two high speed centrifuges. Please state briefly in method.
4. Figure 4: histologic assessment of progression is missing here, please add images of PAS, Masson staining at low magnification
5. Figure 5: Can the authors provide low magnification images (40X) for each condition? A histological evaluation of kidney damage is critical to support the conclusion.
6. Figure 6: Why are no ubiquitin-related catabolic processes or pathways enriched in C171Y? The authors should perform GSEA analysis to determine whether defined gene sets have significant differences between C171Y and R186S.
7. Following the experiments in Figures 7 and 8, the authors should assess whether administration of autophagy agonists could improve kidney injury and function in R186S mice.

2. Significance:

Significance (Required)

Although ADTKD is an rare inherit kidney disease, the authors provide new insight into its pathogenesis. As nephrologist, I agreed with the observations and conclusions provided by the study. However, sufficient histological assessment and in vivo validation of the proposed therapeutic strategy would significantly improve this study.

3. How much time do you estimate the authors will need to complete the suggested revisions:

Estimated time to Complete Revisions (Required)

(Decision Recommendation)

Between 1 and 3 months

4. *Review Commons* values the work of reviewers and encourages them to get credit for their work. Select 'Yes' below to register your reviewing activity at Web of Science Reviewer Recognition Service (formerly Publons); note that the content of your review will not be visible on Web of Science.

Web of Science Reviewer Recognition

Yes

Review #3

1. Evidence, reproducibility and clarity:

Evidence, reproducibility and clarity (Required)

Schiano and colleagues present data on two mouse knock-in models with a missense mutation in uromodulin (C171Y and R186S). A strength of the paper is that the mutations are found in patients with autosomal dominant tubulointerstitial kidney disease (ADTKD) but lead to divergent disease progression. The mouse models are characterized in detail examining changes in uromodulin processing, plasma and urine biochemistry and transcript levels by RNA-sequencing. These findings combined with studies in collecting duct lines provide evidence that the extent of uromodulin aggregate formation is related to the severity of the disease and mechanisms are provided to explain these findings including clearance pathway which might be targeted in the future. Overall, there is a large quantity of good data in the manuscript which moves our understanding of uromodulin mutations forward. However, there are some issues that need to be addressed as outlined below.

Major Comments

1. In the Introduction, the authors state that the current mouse models have only provided limited information warranting this new study. More information is required here to provide a stronger rationale. What are the specific weaknesses of the prior approaches and what precise questions remain unanswered and how is this hindering therapeutic development. Subsequently, how does this study fill these gaps in our knowledge? This narrative of highlighting the new aspects of this study should also run through the Abstract of the paper more prominently.
2. The authors have selected two missense mutations from the Belgo-Swiss ADTKD Registry to subsequently model in mice. Are these mutations also present at a high prevalence in other genetic studies of ADTKD? The authors indicate that the patients with a Arg185Ser mutation have a faster progression than Cys170Tyr. One caveat here is that in Supplementary Table 1-2, the patients with Arg185Ser are predominately male and those with Cys170Tyr predominately female. Therefore, is gender playing a role here with males more susceptible

to renal disease. Taking this concept forward, if the generated mice are separated by gender are comparable results seen in pathology and renal function parameters than if the animals are grouped together as presented in the paper.

3. In Figure 1D, an examination of kidney biopsies is undertaken. Can the authors provide any quantification across multiple samples/sections/cells to strengthen this data? The authors measure CD3+ cells in their mouse models - any evidence of these cells in the human biopsies.

4. In Figure 2C, the quantification presented does not seem to fully reflect the pattern of the blot shown, for example, increase in total signal seen in homozygous mice versus heterozygous C171Y mice. As one of the focuses of the paper is the formation of uromodulin aggregates, perhaps there is a rationale for the core and HMW proteins to be quantified separately, rather than the ratio between them.

5. The authors use electron microscopy (Figure 2F) to conclude that expansion and hyperplasia of the ER occurs in their mutant mice. A representative snapshot is shown, but can quantification be provided to strengthen this data.

6. A detailed assessment of plasma and urine biochemistry has been made. As highlighted above, separating this data by sex could be helpful. It is stated that the C171Y mice have a progressive increase in BUN at 4 months, but this statement requires clarification. Are the authors referring to a progressive change over time or with respect to gene dosage? An additional measurement of creatinine clearance might also be useful here. Are there any changes in glomerular function? Significant changes are also found in the urine of C171 heterozygous mice (in sodium and creatinine) but not in the homozygous animals. Any explanation for these findings which are not mentioned in the text? Some of the data is not reported corrected, for example it is stated that uric acid excretion is reduced at 1 month, but this has not been measured then. The conclusion that there are strong gene-dosage effects in both models seems strong. The reviewer agrees this holds for BUN but is not so clear cut for other parameters such as diuresis and osmolarity in C171Y mice. This should be refined.

7. An interesting analysis is presented on the effect of partial and total denaturation treatments of uromodulin. The reproducibility of these experiments is unclear. Please clarify. Do the authors have any information on how the protein structure of uromodulin might change due to these mutations, for example by structural modelling?

8. Next, the authors delete a wild-type allele in the R186S mice and examine the severity of disease. In Figure 4D and E it would be more informative to also present the specific changes in HMW and core proteins separately. Is there really a pronounced reduction in premature uromodulin in Figure 4E? Why have the authors focused on CD3+ cells as a marker of inflammation, how about other cell types such as macrophages? The rationale needs to be provided here. Are there changes in fibrosis by histology? Importantly, there appears to be no changes in clinical parameters when the wild-type allele is deleted, so is the main conclusion of this part that the deletion of the wild-type allele has no effect on disease severity, despite some of the gene changes observed.

9. In Figure 5, the relationship between the amount of uromodulin aggregates and the UPR pathway, fibrosis and inflammation is examined. As highlighted above, the methodology to determine the number of uromodulin aggregates needs to be considered. It is unclear in Figure 5C how this parameter has been generated. Can the authors present the data in this panel as individual mice of all six groups rather than the grouped analysis currently done. This would distinguish if the individual mice with greatest uromodulin aggregates also had the most fibrosis and inflammation and strengthen the presentation of this data.

10. In your RNA-sequencing data, please clarify if the mice were of the same sex. Interesting changes are found, but the final conclusion is that the transcription signals recapitulate severe ADTMD. This seems an overinterpretation and to strengthen this section the authors could go

back to their biopsy samples and examine some of the expression patterns of the novel genes they have identified. Similarly, can any of the novel transcripts identified in the RNA-seq be examined (and/or) altered in the cell lines they have generated with the same mutations in uromodulin.
11. Using their cells the authors show the autophagy may be involved in the clearance of uromodulin in R185S mutants. However, this pathway is not explored in vivo, an assessment of autophagy in these mice would strengthen this connection.

Minor

1. The authors should present full Western blots in their Supplementary data
2. Figure 2C (and others). Please clarify and label clearly the blots from 1 month and 4-month-old mice.

2. Significance:

Significance (Required)

Schiano and colleagues present data on two mouse knock-in models with a missense mutation in uromodulin (C171Y and R186S). A strength of the paper is that the mutations are found in patients with autosomal dominant tubulointerstitial kidney disease (ADTKD) but lead to divergent disease progression. The mouse models are characterized in detail examining changes in uromodulin processing, plasma and urine biochemistry and transcript levels by RNA-sequencing. These findings combined with studies in collecting duct lines provide evidence that the extent of uromodulin aggregate formation is related to the severity of the disease and mechanisms are provided to explain these findings including clearance pathway which might be targeted in the future. Overall, there is a large quantity of good data in the manuscript which moves our understanding of uromodulin mutations forward. However, there are some issues that need to be addressed; in particular the authors should (i) precisely outline the novelty of their study compared with the prior literature; (ii) clarify the reproducibility of their experiments; (iii) refine areas of overinterpretation in the manuscript; (iv) consider the potential role of gender in their findings and (v) complete the circle in some of their findings, for example examining the novel genes identified in their RNA-sequencing in their human biopsy samples and examining autophagy in their mouse models. These changes will considerably strengthen their article.

3. How much time do you estimate the authors will need to complete the suggested revisions:

Estimated time to Complete Revisions (Required)

(Decision Recommendation)

Between 3 and 6 months

4. *Review Commons* values the work of reviewers and encourages them to get credit for their work. Select 'Yes'

below to register your reviewing activity at Web of Science Reviewer Recognition Service (formerly Publons); note that the content of your review will not be visible on Web of Science.

Web of Science Reviewer Recognition

Yes

Full Revision

Manuscript number: RC-2022-01754
Corresponding author(s): Olivier, Devuyst

[Please use this template only if the submitted manuscript should be considered by the affiliate journal as a full revision in response to the points raised by the reviewers.

If you wish to submit a preliminary revision with a revision plan, please use our "Revision Plan" template. ***It is important to use the appropriate template to clearly inform the editors of your intentions.****]*

1. General Statements [optional]

This section is optional. Insert here any general statements you wish to make about the goal of the study or about the reviews.

Missense mutations in the *UMOD* gene encoding uromodulin cause **autosomal dominant tubulointerstitial kidney disease** (ADTKD-*UMOD*), **one of the most common monogenic kidney diseases**. Affected individuals develop chronic kidney disease (CKD) and, ultimately, kidney failure. The ***UMOD*** **gene encodes uromodulin**, a kidney-specific protein that is abundantly excreted in the normal urine. Variants in *UMOD* generate a big interest, as they show **various effect sizes** involved in a spectrum of kidney disorders. The limitations of current models, the unknown impact of allelic and gene dosage effects, and the unrecognized fate of mutant uromodulin left open the **gap between postulated gain-of-function mutations in uromodulin, end-organ damage and disease progression in ADTKD**.

Based on two prevalent missense *UMOD* mutations associated with divergent disease progression, we generated *Umod* knock-in mice that showed strong allelic and gene dosage effects on uromodulin trafficking, formation of biochemically distinct intracellular aggregates, activation of ER stress and unfolded protein responses, kidney damage and progression to kidney failure. Deletion of the wild-type *Umod* allele in heterozygous *Umod*^{R186S} mice increased the formation of uromodulin aggregates and ER stress, indicating a protective role of wild-type uromodulin. Studies in kidney tubular cells confirmed differences between distinct uromodulin aggregates, with activation of mutation-specific quality control and clearance mechanisms. Enhancement of autophagy by starvation and mTORC1 inhibition decreased the uromodulin aggregates, suggesting a therapeutic strategy.

The revised version addresses all the comments provided by the Reviewers. In particular, we included additional data on the specific degradation mechanisms involved in the clearance of the mutant protein aggregates; differential pathways triggered by the mutations; phenotypical characterization of the models; choice of the mutations; gender effect; and various technical and

methodology details. Altogether, these additions support our initial conclusions and strengthen the study.

These studies substantiate a **model of allelic effects** and the **role of toxic aggregates** in ADTKD-*UMOD*, with relevance for **strategies to improve clearance** of mutant uromodulin and, more generally, for toxic **gain-of-function mechanisms in dominant diseases**.

2. Point-by-point description of the revisions

This section is mandatory. Please insert a point-by-point reply describing the revisions that were <u>*already carried out and included*</u> *in the transferred manuscript.*

Reviewer #1 (Evidence, reproducibility and clarity (Required)):

Uromodulin (Tamm-Horsfall protein) is the most abundant protein excreted in human urine.
It plays role in protection against urinary tract infections and renal stones. Mutations in UMOD gene encoding uromodulin cause Autosomal Dominant Tubulointerstitial Disease (ADTKD) that slowly progresses to chronic kidney disease.

In this manuscript, Schiano et al. isolate 12 missense UMOD mutations, which they classify into two groups by age occurrence. They then proceed to study two of these mutations: one from the earlier-onset - Arg185Ser - and the second from the later-onset - Cys170Tyr.

The authors generate UmodC171Y and UmodR186S knock-in mice with distinct dynamic pathways impacting on ADTKD progression. These mutations are equivalent with UMOD mutations (C170Y and R185S) in patients. UmodC171Y and UmodR186S knock-in mice show impaired uromodulin biogenesis, with strong allelic and gene-dosage effects. The trafficking problem of ADTKD-UMOD mutants, involving ER retention, ER stress, and activation of the UPR is recapitulated in mIMCD-3 cells, where the R185S mutant reveals more aggregates that are triggering PERK and IRE1 pathways and ER stress responses.

The manuscript is well written, experiments are in general well described and performed, results offer important insights on cellular events eventually leading to organ damage in ADTKD resulting from missense mutation in the UMOD gene. The part of the work investigating the degradation mode of two different UMOD mutants, one relying on proteasomal and one relying on lysosomal clearance, is the most interesting for a general audience. Unfortunately, this last part of the work is too preliminary to be accepted as it is.
***We thank the Reviewer for her/his positive comments and for pointing the important mechanistic insights in organ damage provided by our work. We agree on the specific interest of the specific degradation mechanisms of the two different UMOD mutants, as this represents a novel concept in the field of kidney diseases. We are glad to submit a revised version that substantiates this issue with additional evidence in vivo and in vitro, as suggested.

Review COMMONS

Comments/Suggestions:
- Selection of the UMOD variants, page 5: "R185S and C170Y are the most prevalent mutants in the clusters" please document/add reference.
***The statement refers to the specific cohort of patients that was used for the genotype-phenotype analysis. Among these patients, C170Y and R185S mutants are the most prevalent among slow and fast-progressing individuals respectively. The whole cohort has been described previously (Olinger et al. 2020). This point is now detailed in the Methods (P22, with reference #2).

- Fig. 1D: please show the position of the insets in the UMOD and BiP panels. Please separate the IF panels from the Picrosirius red panels (these are not the same samples that are shown).
***Thank you. We included the position of the insets on Fig. 1D and put the Picrosirius red staining in a new panel on Fig. 1E.

Formally, the BiP panels in Fig. 1D reveal that there is more BiP in cells expressing R185S. That this correlates with UPR induction (as confirmed in Fig. x) should be written at the end of page 5 to make this issue clear for non-experts.
***We agree and have clarified the role of BiP as a marker of UPR induction (P5). Further analysis of the p. Cys170Tyr biopsy revealed presence of intracellular UMOD accumulation and increased BiP signal, although to lesser extent compared to the R185S specimen. We have included these precisions in the Results (P5).

In Fig. 1D, the signal of BiP is not visible in WT and C170Y tissue/cells, which is odd because BiP is abundant protein. Moreover, the differences in BiP levels quantified in WB (semi-quantitative analyses) are not that dramatic in the mouse model (SFig. 3). Which panel in SFig. 3 (mouse) should be representative of the IF shown in Fig. 1D (patients)?
***As the original staining for BiP was performed a while ago, we repeated the immunostaining and observed a positive BiP signal in all samples. As stated by the Reviewer, the selective upregulation of BiP in the TAL (over the whole kidney) explains why immunofluorescence and immunoblots on isolated TALs are better at capturing such differences than analyses performed on whole kidneys. Accordingly, a head-to-head comparison between Fig. 1D (immunostaining, human kidney) and Suppl. Fig. 3 (immunoblot, whole mouse kidney) is not really informative.

Fig. 1D: Magnification of these images is not sufficient to conclude that R185S accumulates in the ER, and that WT and C170Y are at the apical cell's membrane as written (page 5). Authors should refer to Suppl Fig 1C, where individual cells are visible.
***The Reviewer rightly points that the CLEM images provided in Suppl. Fig. 1C represent a more precise characterization of UMOD localization. Therefore, we decided to refer to the UMOD accumulation observed in Fig. 1D simply as “intracellular accumulation” (P5).

Authors should briefly explain at the end of page 5 how the P. red staining in Fig. 1D informs on fibrosis.

***We have included a statement (P6) to explain the use of Picrosirius red, a well-established staining for collagen fibers found in interstitial fibrosis (Courtoy et al. 2020; Suppl. Methods P4). A strong interstitial fibrosis was present in the R185S biopsy, whereas the collagen-positive area was less pronounced in the C170Y specimen (Fig. 1E).

- In the analyses of misfolded UMOD mutants (e.g., Fig. 2, 3, 4, ...) one would expect a test showing that BiP associates with R185S>C170Y>WT.
***We thank the Reviewer for this suggestion. We complemented the immunofluorescence analysis for GRP78 (BiP) (Fig. 5B) with a statistical analysis for the comparison between WT and different mutants (One-way ANOVA with Tukey's post hoc test). These analyses clearly show the progressive, significant increase of GRP78 intensity following R186S>C171Y>WT. We included this analysis on Fig. 5B and in the Results (P11).

- Fig. 2F: in R186S there is a dramatic enlargement (at least 2x) of nuclei. Can the authors comment on that?
***The apparent difference in nucleus size observed in R186S samples does not result from nucleus enlargement but is an artifact due to sample sectioning. In fact, we measured the nucleus size in UMOD-positive tubules and did not observe any significant difference between R186S and WT tubules (26.9 vs 25.9 μm^2, $P = 0.44$, n ≥ 70 nuclei in 30 tubules). Nuclei of different sizes are present in all 3 tubules shown in Fig. 2F. We have replaced the R186S panel in Fig. 2F with a more representative tubule, where this artifact is less evident.

- Fig. 7E: Shouldn't one expects apical signal for C170Y?
- Fig. 7F: Why there is apical signal for R185S (and not for C170Y)?

*** The *UMOD*-GFP cells were generated by lentiviral transduction to express either the wild-type or mutant UMOD transgene, in order to characterize the intracellular processing of UMOD. The immunostaining panels (Figs. 7E and 7F) were acquired on non-polarized cells, distinct from native TAL cells as observed in mouse kidney samples. Despite the lack of polarization, the intracellular retention as well as the downstream effects (i.e. ER stress, UPR) of mutant UMOD are preserved between the in vitro and in vivo systems.

- The part covering the degradation of the two UMOD variants would be of great interest for a wide audience of cell biologists. However, these data are too preliminary and, in this form, inconclusive.
Few examples: MG132 is a non-specific inhibitor of the proteasome, which may enhance endogenous and trans-gene expression (check in Pubmed "mg132 promoter" for relevant literature). Thus, an increase in the intracellular level of C170Y on MG132 treatment does not necessarily indicate inhibition of the protein's proteasomal turnover. It could also, at least in part, be caused by an increased synthesis of UMOD. The authors should show that MG132 does not increase synthesis of mutant UMOD (or use the more selective proteasome inhibitor PS-341 in their experiments);

***We thank the Reviewer for these constructive suggestions. To exclude the possibility that the observed increase in C170Y UMOD levels following MG132 treatment resulted from increased *de novo* synthesis of uromodulin rather than proteasomal inhibition, we performed quantitative PCR to measure the expression levels of *UMOD* mRNA after MG132 treatment (Suppl. Fig. 13D). Our results demonstrate that there was no significant increase in *UMOD* mRNA expression following proteasomal blockage.
To further test the involvement of the ubiquitin-proteasome system in UMOD C170Y clearance, we conducted a time-course treatment of *UMOD*-GFP cells using the selective proteasome inhibitor PS-341 (Bortezomib) at the established concentration of 1 μM (Suppl. Fig. 13F). The effect of Bortezomib was demonstrated by a consistent, time-dependent increase on polyubiquitin levels in all cell types, confirming the effective inhibition of proteasomal proteolytic activity. Importantly, treatment with Bortezomib led to a significant increase in UMOD C170Y protein levels, observed 6h after drug exposure, whereas it had no effect on the wild-type or on the R186S UMOD.
These results provide compelling evidence that the ubiquitin-proteasome pathway plays a crucial role in the selective degradation of the C170Y mutant UMOD. The data are included in the new Suppl. Fig. 13 and Results (P14-15).

-Similarly, the data on R185S do not prove that this protein is client of autophagy. They rather show that autophagy removes the protein when cells are under nutrient restriction (note that starvation activates bulk autophagy, the non-selective lysosomal clearance of cellular components). To show that misfolded R185S is removed from cells by misfolded protein-induced ER-phagy (i.e., ER-to-lysosome-associated degradation), the authors should monitor in WB the accumulation of R185S in the presence of BafA1 and/or in IF the accumulation of R185S within lysosomes in the presence of BafA1.

***We agree. To investigate whether mutant UMOD is an autophagy substrate, UMOD-GFP cells were exposed to the autophagy inhibitor Bafilomycin A1 under normal nutrient conditions (Suppl. Fig. 14B). BafA1 treatment induced an increase of UMOD localization in LAMP1+ structures in both *UMOD* R185S (Mean delta +37%, $P < 0.0001$) and C170Y cells (Mean delta +69%, $P < 0.0001$) compared to untreated cells respectively. Comparative analysis confirms that the effect of BafA1 on R185S is significantly lower than that on C170Y mutant UMOD ($P < 0.0001$). Unlike the mutant protein, wild-type UMOD remained predominantly localized at the plasma membrane, with a lower level of UMOD signal detected in LAMP1^{+} structures after BAFA1 treatment.
To further support the role of autophagy on mutant UMOD, we used the selective VPS34 inhibitor SAR405 to prevent autophagosome biogenesis (Suppl. Fig. 14C). While no changes were observed in UMOD WT protein levels, a significant decrease in both UMOD R185S and C170Y levels was observed after starvation, followed by a rescue to baseline levels upon 5μM SAR405 treatment. The impairment of autophagosome formation was confirmed by the accumulation of LC3I and the decrease of p62/SQSTM1 in cells treated with SAR405 (Suppl. Fig. 14C).

Review COMMONS

These results indicate that both mutant UMOD proteins are clients of the autophagy-lysosomal system, emphasizing the potential therapeutic value of this pathway in ADTKD-*UMOD*. Thus, the main difference between the two mutants does not lie in their targeting to autophagy, but rather in the inability of the R185S mutant UMOD to undergo proteasomal degradation, as shown after the proteasomal blockage with either MG132 or Bortezomib.
We have included these novel data in the revised manuscript (P15-16).

Minor comments
- Figure 1B: dotted lines should be defined in the legend.
***We added the definition of dotted lines in the figure key.

- Figure 1C: "phenotypes are denoted as indicated". The color-code used for the phenotype is unclear to me. For example, what is the phenotype of the V.2 (grey square)?
***The grey square in V.2 results from the combination of the two half grey squares of CKD and gout. We added a separate icon for “Gout + CKD” in the panel key.

- The meaning of "Unlike in UMOD R185S cells, higher SQSTM1 puncta colocalizing with uromodulin were initially present in C170Y mutant cells and further accumulated in MG132-treated cells (Supplementary Figures 10A, B). These data suggest that mutant cells respond differently to UPS inhibition, with C170Y mutant uromodulin being mainly targeted to this pathway." (page 14) and the interpretation of the results shown in 10A and 10B is unclear to me.
***We thank the Reviewer for pointing this out. Indeed, the original Suppl. Fig. 10A does not show SQSTM1 increase, but rather ubiquitin levels during the MG132 time course, whereas Suppl. Fig. 10B shows SQSTM1 immunostaining following MG132. To clarify, we have included all the data regarding characterization and modulation of ubiquitin-proteasome system in the revised Suppl. Fig. 13 – also showing SQSTM1 expression levels in steady state condition. These modifications are detailed in the revised manuscript (P14-15).

- Page 7: "The UmodC171Y mice showed a progressive increase in BUN at 4 months" please define BUN.
*** BUN stands for “blood urea nitrogen”, an established marker of kidney function. This is now defined on P7 of the revised manuscript.

- Please, provide a complete list of primary antibodies used for immunoblotting, immunohistochemistry, and immunofluorescence staining.
*** We added a list of all the primary antibodies used in the Suppl. Table S13.

Reviewer #1 (Significance (Required)):

The manuscript is well written, experiments are in general well described and performed, results offer important insights on cellular events eventually leading to organ damage in ADTKD resulting from missense mutation in the UMOD gene.

The part of the work investigating the degradation mode of two different UMOD mutants, one relying on proteasomal and one relying on lysosomal clearance, is the most interesting for a general audience. Unfortunately, this last part of the work is too preliminary to be accepted as it is.

My expertise: protein quality control, ER-phagy

Reviewer #2 (Evidence, reproducibility and clarity (Required)):

UAKD, a subtype of ADTKD, is extensively studied, although it is a rare inherit kidney disease. Using a knock-in strategy, the authors raised a novel concept that the differences in allelic and gene dosage of Umod mutation triggered distinct protein catabolic pathways, yielding distinct phenotypes and prognosis. The functional mechanisms include that UmodR186S mutation caused insoluble uromodulin aggregates resulting in activation of autophagy, and UmodC171Y mutation led to uromodulin misfolding and touched off ubiquitin-dependent ERAD pathway. Accordingly, the authors tested whether enhancing autophagy attenuates the accumulation of UmodR186S protein in cell cultures. Based on these observations, the authors suggested a strategy to improve clearance of mutant uromodulin. This study was carried out by a team with strong reputation in this area. However, the story appears to be incomplete and in vivo testing of their therapeutic strategy is needed to improve this research.
***We thank the Reviewer for his positive comments, highlighting the novelty of our findings and the evidenced functional mechanisms based on in vivo and in vitro models, suggesting a novel therapeutic strategy. We are glad to submit a revised manuscript that addresses the comments provided.

SPECIFIC COMMENTS
1. Figure 1D: Images at low magnification do not show DAPI, therefore there is no information on the total number of cells in the selected field. Nephron loss (represented by glomeruli) did not appear to differ between UMOD C170Y and UMOD R185S, which is inconsistent with the overall conclusions. In addition, PAS staining should be added in Figure 1D.
***We have now added the DAPI channel to Figure 1D. Since ADTKD-*UMOD* is not a disease where kidney biopsies are routinely taken, only a few human samples are available for (qualitative) analyses. Due to the low numbers and heterogeneity in terms of sample and patient characteristics (age, gender), a reliable quantification of nephron loss is not possible on that material. We performed PAS staining on the human samples, showing thickening and splitting of the tubular basal membrane in the C170Y and R185S samples. These findings confirm previously observed features of ADTKD-*UMOD* – i.e. splitting of the tubular basement membranes (Dahan et al. 2001). These new data are included on Fig. 1E and P6 of the revised manuscript.

2. Figure 2E: in image of C171Y/+, this is no corresponding tubules which is represented by the insert.

***Thank you. Due to the orientation of the picture, the high-magnification inset ended up covering the corresponding tubule for C171/+. The orientation of the panel has been modified accordingly.

Figure 2F lower panel, the bars in EM fields are same, indicating a hypertrophy of nuclei in R186S?

***The apparent difference in nucleus size observed in R186S samples does not result from nuclear enlargement but is an artifact due to sample sectioning. In fact, we measured the nucleus size in UMOD-positive tubules and did not observe any significant difference between R186S and WT tubules (26.9 vs 25.9 μm^2, $P = 0.44$, $n \geq 70$ nuclei in 30 tubules). Nuclei of different sizes are present in all 3 tubules shown in Fig. 2F. We have replaced the R186S panel in Fig. 2F with a more representative tubule, where this artifact is less evident.

Figure 2G: how about serum creatinine in these mice? In addition, signs of catabolism (e.g., loss of body weight) are associated with these KI mice?

***Plasma creatinine values are shown in Suppl. Tables 4-6. Significantly increased levels were observed in 4 months R186S/+, R186S/- and R186S/R186S, compared to the controls. At the investigated time points, no significant differences in body weight between wild-type and *Umod* KI mice were observed.

3. Figure 3C: what is rationale of using two high speed centrifuges. Please state briefly in method.

***The rationale behind using these different high-speed centrifugations is rooted in the principle of differential centrifugation, a standard technique for separating molecules with varying sedimentation rates in cellular lysates. This approach has been used for isolating insoluble protein aggregates from human brain tissue (Arseni et al. 2022). In our study, the use of multiple ultracentrifugation steps was necessary to effectively separate the insoluble pellets containing aggregate and monomeric uromodulin from the soluble counterpart. The protocol is detailed and referenced in the Methods (P26).

4. Figure 4: histologic assessment of progression is missing here, please add images of PAS, Masson staining at low magnification

***To address this point, we performed additional staining in *Umod* KI kidneys, including Masson's trichrome and Picrosirius red at low and high magnifications (Suppl. Fig. 8). Picrosirius red staining is established as a reliable tool for interstitial fibrosis quantification in different organs, including the kidney (Courtoy et al. 2020). The quantification of interstitial fibrosis was performed using picrosirius red, as shown in Fig. 5B, pointing clearly to a strong allelic effect on ER stress, inflammatory infiltrate and fibrosis markers of progression.

5. Figure 5: Can the authors provide low magnification images (40X) for each condition? A histological evaluation of kidney damage is critical to support the conclusion.

***Low magnification images for the various stainings have been added (in Suppl. Fig. 8). The histological evaluation of kidney damage has been performed on whole section scans (200x

resolution, using a Zeiss Axio Scan.z1 slide scanner), for all mouse strains examined. This is indicated in the Supplementary Methods, P4.

6. Figure 6: Why are no ubiquitin-related catabolic processes or pathways enriched in C171Y? The authors should perform GSEA analysis to determine whether defined gene sets have significant differences between C171Y and R186S.
***The transcriptomic profiling of *Umod* KI mice was performed using over-representation analysis (ORA), which only takes into account statistically significant (FDR-adjusted p-value < 0.05) differentially expressed genes (DEGs). Accordingly, this method provides less redundant results compared to GSEA, but it can result in some enriched pathways not being properly detected. To address the point, we performed GSEA on *Umod* C171Y compared to both WT and R186S samples. At 1 month, gene sets upregulated in C171Y were almost exclusively associated with cell replication (i.e. spindle checkpoint signaling, chromatid separation and chromosome localization). At 4 months, we observed a significant upregulation of Skp1/Cul1/F-box protein (SCF)-dependent proteasomal ubiquitin-dependent protein catabolism in C171Y samples compared to WT. Of note, comparing C171Y and R186S confirmed the transcriptomic signatures observed with ORA, with enrichment of protein folding pathways in the C171Y and increased immune response in the R186S. We have added this novel information in the revised manuscript (Results, P12).

7. Following the experiments in Figures 7 and 8, the authors should assess whether administration of autophagy agonists could improve kidney injury and function in R186S mice.
***We agree with the Reviewer that the effect of an *in vivo* treatment to enhance autophagy using our mutant mice would be very interesting to study. However, we feel that such protocols, which would require an entirely new animal license, are beyond the scope of the current study.

Reviewer #2 (Significance (Required)):

Although ADTKD is an rare inherit kidney disease, the authors provide new insight into its pathogenesis. As nephrologist, I agreed with the observations and conclusions provided by the study. However, sufficient histological assessment and in vivo validation of the proposed therapeutic strategy would significantly improve this study.

Reviewer #3 (Evidence, reproducibility and clarity (Required)):

Schiano and colleagues present data on two mouse knock-in models with a missense mutation in uromodulin (C171Y and R186S). A strength of the paper is that the mutations are found in patients with autosomal dominant tubulointerstitial kidney disease (ADTKD) but lead to divergent disease progression. The mouse models are characterized in detail examining changes in uromodulin processing, plasma and urine biochemistry and transcript levels by RNA-sequencing. These findings combined with studies in collecting duct lines provide evidence that the extent of uromodulin aggregate formation is related to the severity of the disease and

mechanisms are provided to explain these findings including clearance pathway which might be targeted in the future. Overall, there is a large quantity of good data in the manuscript which moves our understanding of uromodulin mutations forward. However, there are some issues that need to be addressed as outlined below.
***We thank the Reviewer for his/her appreciation of our study, which moves forward the understanding of the disease due to uromodulin mutations. We are glad to submit a revised version that addresses all the issues as suggested.

Major Comments
1. In the Introduction, the authors state that the current mouse models have only provided limited information warranting this new study. More information is required here to provide a stronger rationale. What are the specific weaknesses of the prior approaches and what precise questions remain unanswered and how is this hindering therapeutic development. Subsequently, how does this study fill these gaps in our knowledge? This narrative of highlighting the new aspects of this study should also run through the Abstract of the paper more prominently.
***We thank the Reviewer for this suggestion. In brief, the limitations of the current models include technical issues (transgenesis resulting in overexpression of mutant, ENU random mutagenesis); lack of corresponding human mutations, in particular representative of distinct disease progression in an established registry; limited phenotype analysis, e.g. multi-level time-course analysis of disease progression; conflicting influence of apoptosis (CRISPR/Cas9 model), not verified in KI models or in vivo samples; lack of investigation of the fate of mutant uromodulin and in particular the generation and degradation of intracellular aggregates; influence of the wild-type allele; and how representative human mutations affect disease progression. Together, these gaps prevented to elucidate the link between postulated gain-of-toxic function mutations and clinically-relevant endpoints, slowing the development of new therapeutic targets. We have included more emphasis of limitations of existing models, the gaps of knowledge and the rationale of the study in the Abstract, Introduction (P4) and Discussion (P18).

2. The authors have selected two missense mutations from the Belgo-Swiss ADTKD Registry to subsequently model in mice. Are these mutations also present at a high prevalence in other genetic studies of ADTKD? The authors indicate that the patients with a Arg185Ser mutation have a faster progression than Cys170Tyr. One caveat here is that in Supplementary Table 1-2, the patients with Arg185Ser are predominately male and those with Cys170Tyr predominately female. Therefore, is gender playing a role here with males more susceptible to renal disease.
***We welcome the opportunity to address these issues. The selection of the R185S and C170Y mutations was based on the number of patients with these mutations that reached end stage kidney disease (ESKD) in the Belgo-Swiss cohort, and for which we had reliable clinical data. Among these patients, C170Y and R185S mutants are the most prevalent among slow and fast-progressing individuals respectively (Olinger et al. 2020). With only a few exceptions, missense mutations in *UMOD* are private and occur throughout the N-terminal half of the protein. For this reason, it is not surprising that, to the best of our knowledge, these two mutations have not been

described in other published cohorts (Kidd et al. 2020; Olinger et al. 2022). Furthermore, given the presumed importance of disulphide bridges for uromodulin folding, we were eager to study a mutation that is involving a cysteine and another that is not.
This rationale is detailed in the Results (P5) and Methods (P22).
*** Regarding a possible effect of gender on ADTKD-*UMOD* progression, Kidd et al. (2020) showed indeed that male gender was associated with an increased risk of reaching ESKD at an earlier age in an international cohort. ESKD was uncommon before age 30, with approximately 50% of the male cohort reaching ESKD between 30 and 50, and 50% of the female cohort reaching ESKD between 30 and 60. Yet, it is difficult to extrapolate from this international cohort, taken into account large differences in clinical practice and significant intrafamilial variability in disease progression in ADTKD-*UMOD* (Bollée et al. 2011; Devuyst et al. 2019). Furthermore, in general, males have an almost two-fold increased lifetime risk for ESKD compared to females (4% vs 2.8% respectively; Albertus et al. 2016). In the rapid progressing R185S family included in our study, the proportion of males and females reaching ESKD was similar (3 females and 4 males). It is thus difficult to state on a possible specific effect of gender in that case – as also indicated by the mouse studies (see below).
We have summarized this point in the Results (P4 and P9).

Taking this concept forward, if the generated mice are separated by gender are comparable results seen in pathology and renal function parameters than if the animals are grouped together as presented in the paper.
***Our mouse protocols included both genders in matched proportions, as recommended (ISN Consensus, Nangaku et al. 2023). Stratification of the statistical analyses by gender yielded trends that were similar to the global analyses. In particular, the increase of the BUN at 4 months was observed in both *Umod*$^{C171Y/C171Y}$ male and female mice compared to their respective *Umod*$^{+/+}$ controls. Similarly, the increased BUN levels, increased urine output, and decreased urine osmolality (with the gene dosage effect) were observed in the *Umod*$^{R186S/+}$, *Umod*$^{R186S/-}$ and *Umod*$^{R186S/R186S}$ female and male mice when stratified by gender and compared to their respective littermates (new Suppl. Table 7). Thus, the analysis on gender-split animals did not highlight major differences in disease progression. This data is included on Suppl. Fig. 7 and the Results (P9).

3. In Figure 1D, an examination of kidney biopsies is undertaken. Can the authors provide any quantification across multiple samples/sections/cells to strengthen this data? The authors measure CD3+ cells in their mouse models - any evidence of these cells in the human biopsies.
***We thank the Reviewer for this comment. Since kidney biopsies are not routinely performed in patients with ADTKD-*UMOD* (there are typically no glomerular manifestations), we lack sufficient human biopsy material to perform in-depth quantitative analyses. The data shown in Fig. 1D are meant to show qualitative differences between the two different mutations. We have performed new immunostaining for CD3 in these biopsies, evidencing a higher number of CD3^{+} cells in the R185S compared to the C170Y biopsy (Fig. 1D; Results P5).

4. In Figure 2C, the quantification presented does not seem to fully reflect the pattern of the blot

shown, for example, increase in total signal seen in homozygous mice versus heterozygous C171Y mice. As one of the focuses of the paper is the formation of uromodulin aggregates, perhaps there is a rationale for the core and HMW proteins to be quantified separately, rather than the ratio between them.

***Many thanks for that point. We have performed distinct quantifications for core and aggregate isoforms of uromodulin, depending on the emphasis.

In Fig. 2C-D, we separately quantify core (including mature and premature) uromodulin levels (in the kidney and urine, relative to wild-type) and the aggregates (in R186S homozygotes, relative to heterozygotes), over beta-actin. We did not include quantifications of aggregates in C171Y samples, as they are not detected in heterozygotes. These analyses show the progressive accumulation of core UMOD in the mutant kidneys, paralleled by a decrease in its urine excretion, and the appearance of HMW UMOD aggregates with allelic and gene dosage effects.

In Fig. 5A-C, we show, at 4 months of age, and for all genotypes, the level of HMW UMOD aggregates over the total UMOD signal. This relative quantification, in a large cumulative number of genotypes and samples, reflects the aggregation process that is then correlated with the tissular markers of ER stress (GRP78), inflammation (CD3) and fibrosis.

We have included these precisions in the Methods (P26), Results (P6 and P10) and legends.

5. The authors use electron microscopy (Figure 2F) to conclude that expansion and hyperplasia of the ER occurs in their mutant mice. A representative snapshot is shown, but can quantification be provided to strengthen this data.

***The ER enlargement was observed in several tubules coming from different mice and was exclusively observed in Umod$^{R186S/+}$ samples. Due to the vesicular structure of ER, particularly in *Umod*$^{+/+}$, a reliable quantification of the ER area by electron microscopy would be extremely difficult to perform in a robust fashion. Furthermore, ER hyperplasia associated with ADTKD-*UMOD* has already been described in human and mouse kidneys (Devuyst et al. 2019).

6. A detailed assessment of plasma and urine biochemistry has been made. As highlighted above, separating this data by sex could be helpful. It is stated that the C171Y mice have a progressive increase in BUN at 4 months, but this statement requires clarification. Are the authors referring to a progressive change over time or with respect to gene dosage? An additional measurement of creatinine clearance might also be useful here. Are there any changes in glomerular function?

***As shown in Suppl. Table 4, the increase in BUN refers to a change over time (between 1 month and 4 months of age) in a given genotype. Yet, at 4 months, we also observed higher levels of BUN in the homozygotes compared to the heterozygotes. We have now indicated that effect in Results (P7).

Our assessment of kidney function relies on plasma levels of creatinine (enzymatic) and BUN, as done in many studies (Nangaku et al. 2023). In fact, both plasma creatinine and BUN levels increase over time in the mutant models, with remarkable allelic and gene-dosage effects (Suppl. Tables 4-6), that parallel the structural damages in the kidneys. The gold standard for

measuring GFR in mouse, i.e. fluorescein-isothiocyanate-labelled sinistrin (FITC-S), is not included in our ethics license.

Significant changes are also found in the urine of C171 heterozygous mice (in sodium and creatinine) but not in the homozygous animals. Any explanation for these findings which are not mentioned in the text? Some of the data is not reported corrected, for example it is stated that uric acid excretion is reduced at 1 month, but this has not been measured then. The conclusion that there are strong gene-dosage effects in both models seems strong. The reviewer agrees this holds for BUN but is not so clear cut for other parameters such as diuresis and osmolarity in C171Y mice. This should be refined.
***We thank the Reviewer this comment. We indeed observed a slight increase in urinary sodium and a slight decrease in urinary calcium in C171Y/+ mice compared to the wild-type mice (Suppl. Table 4). In absence of any macroscopic indication of kidney damage (Fig. 5B), these changes may reflect the gender distribution in this specific genotype. We have to keep in mind that the changes in the C171Y mice are discrete compared to the other lines, with only mild increases in BUN observed at 4 months, with no impact on urinary concentrating ability for instance.

7. An interesting analysis is presented on the effect of partial and total denaturation treatments of uromodulin. The reproducibility of these experiments is unclear. Please clarify. Do the authors have any information on how the protein structure of uromodulin might change due to these mutations, for example by structural modelling?

***The characterization of UMOD aggregates following partial and total denaturation has been performed in technical triplicates, with 3 biological replicates per experiment. The loss of HMW bands was consistently observed following complete denaturation of the samples. This is now precised in the Methods (P26) and appropriate legends.
We explored the potential structural changes caused by the C170Y and R185S mutations by performing structural modeling based on the human UMOD structure (https://www.rcsb.org/structure/7PFP), as the currently available experimental models for mouse uromodulin do not cover the region of the two mutations. Our model of the C170Y mutation shows the disruption of a disulphide bridge with C155. Furthermore, as tyrosine is a bulkier residue compared to cysteine, several clashes with surrounding residues can be observed. Modeling of the R185S mutation results in the loss of a buried salt bridge with Asp196. It should be noted that several ADTKD-causing mutations affecting either Arg185 or Asp196 have been described (Devuyst et al, 2017; Olinger et al, 2020), suggesting the importance of this salt bridge for overall protein stability. The importance of this interaction for overall protein stability is supported by the relatively high number of ADTKD-causing missense mutations affecting Arg185 and Asp196. These new data are included in the Discussion (P20), Suppl. Fig. 15 and Suppl. Methods P4.

8. Next, the authors delete a wild-type allele in the R186S mice and examine the severity of

disease. In Figure 4D and E it would be more informative to also present the specific changes in HMW and core proteins separately.
*** We agree. Separate quantifications for core and HMW UMOD have now been provided in the revised Figures 4D,E.

Is there really a pronounced reduction in premature uromodulin in Figure 4E?
***We agree that the reduction in premature uromodulin in isolated TALs (Fig. 4E) is not visible as it is in whole kidney extracts (Fig. 4D). The isolation of the TAL allows to collect pure TAL fractions from the medulla, avoiding the diluting effect of the kidney. Due to the filtering and picking method, the cortex is discarded - excluding a certain proportion of TALs. Yet, the qualitative changes observed in whole kidney samples, including shift from mature to premature UMOD, apparition of HMW aggregates, and stimulation of GRP78 are observed. In particular, the TAL enrichment allowed us to better observe changes in the HMW UMOD and the induction of the GRP78 in *Umod*$^{R186S/-}$ samples. We have edited the text accordingly (Results, P9).

Why have the authors focused on CD3+ cells as a marker of inflammation, how about other cell types such as macrophages? The rationale needs to be provided here.
***We used CD3+ cells as a general marker of immune cell infiltration. As suggested, we performed a staining of the macrophage marker F4/80. Indeed, we observed that the F4/80-positive area follows the same allelic and gene-dosage effect observed for CD3-positive cells, suggesting a more general effect on the immune response, not limited to T-cells.
These new data are presented in the new Suppl. Fig. 7 and Results (P11).

Are there changes in fibrosis by histology? Importantly, there appears to be no changes in clinical parameters when the wild-type allele is deleted, so is the main conclusion of this part that the deletion of the wild-type allele has no effect on disease severity, despite some of the gene changes observed.
***Indeed, we did not observe significant differences in terms of interstitial fibrosis between *Umod* R186S/+ and R186S/- kidneys (Fig. 5). However, our results show that the deletion of the wild-type allele led to an early increase of UMOD aggregates compared to heterozygous mice (R186S/+) (Fig. 4). The increase of uromodulin towards aggregation is significant at 1 month old and is paralleled by an early kidney damage response (inflammation, damage markers, fibrosis markers), with no significant change in the clinical parameters. The latter observation may be due to the rapid disease progression in this genotype, with accumulation of mutant uromodulin, so that the effect of the deletion of the wild-type allele may be diluted. We have included this point in the Discussion (P19).

9. In Figure 5, the relationship between the amount of uromodulin aggregates and the UPR pathway, fibrosis and inflammation is examined. As highlighted above, the methodology to determine the number of uromodulin aggregates needs to be considered. It is unclear in Figure 5C how this parameter has been generated. Can the authors present the data in this panel as individual mice of all six groups rather than the grouped analysis currently done. This would distinguish if the individual mice with greatest uromodulin aggregates also had the most fibrosis and inflammation and strengthen the presentation of this data.
***Thank you for this suggestion. We now present Fig. 5C with the individual data in each group, which indeed strengthens the correlations. As discussed above (see point 4), we have now

quantified the aggregates as percentage over total uromodulin rather than ratio over core, confirming the allelic- and genotype-dependent increase of UMOD aggregates observed previously. The legend of Fig. 5C was adjusted accordingly.

10. In your RNA-sequencing data, please clarify if the mice were of the same sex. Interesting changes are found, but the final conclusion is that the transcription signals recapitulate severe ADTMD. This seems an overinterpretation and to strengthen this section the authors could go back to their biopsy samples and examine some of the expression patterns of the novel genes they have identified. Similarly, can any of the novel transcripts identified in the RNA-seq be examined (and/or) altered in the cell lines they have generated with the same mutations in uromodulin.
***We have clarified in the Methods that the RNA-sequencing was done on kidneys from male mice, matched for age (P28).
Overall, the transcriptomic signature observed in R186S kidneys is indeed compatible with severe ADTKD, as shown by upregulation of ER stress genes, inflammatory, and profibrotic signaling. However, this was not the case for C171Y samples, where not only a milder ER stress and inflammatory response were observed, together with an enhanced folding capacity that could contribute to the reduced severity of this mutation.
As discussed above, the limited availability of kidney biopsies in ADTKD prevents the validation of expression patterns obtained in mouse models. However, the characterization of ER stress (GRP78), inflammation (CD3) and tubular damage (picrosirius red, PAS) observed in the human kidney samples parallels the differences in severity observed in the mouse kidneys.
The transcription signals, including the differential upregulation of ER stress genes (*Hspa5, Lcn2*), was validated on both mouse kidneys (Figure 6E) and *UMOD*-GFP cells (Suppl. Figure 12). Similarly, validation of protein folding chaperones (*Dnaja4*, *Hsp90ab1*) and ER-phagy genes (*Rtn3*, *Ccpg1*, *Sec62*) was provided for both mouse models (Suppl. Figure 5A) and cell systems (Suppl. Figure 12). We have included these statements in the revised manuscript (Results, P14).

11. Using their cells the authors show the autophagy may be involved in the clearance of uromodulin in R185S mutants. However, this pathway is not explored in vivo, an assessment of autophagy in these mice would strengthen this connection.
***We thank the Reviewer for this suggestion. Our initial analysis of ER-phagy genes in the mutant kidneys (Suppl. Fig. 5) shows a distinct transcriptomic signature in the two mouse lines, with upregulation of ER-phagy in C171Y kidneys, in contrast with the other mutations. This suggests that mutant UMOD degradation relies on mutation-specific mechanisms.
As suggested, we assessed autophagy induction in the mutant kidneys, based on stainings for SQSTM1 (Suppl. Fig. 5B) and autophagy related 5 (ATG5), a key component of the phagophore extension process (Suppl. Fig. 6). These experiments showed a mutation-dependent effect on both proteins in UMOD-positive tubules, with a milder increase in C171Y kidneys contrasting with a stronger signal in R186S mutants. Overall, our data support the role of autophagy as a potential target to stimulate uromodulin clearance. As steady-state autophagy levels are insufficient to counteract the uromodulin accumulation and aggregation observed in R186S,

enhancement of this pathway represents a valuable therapeutical strategy. This conclusion is also supported by the in vitro evidence, clearly showing that both mutant UMOD proteins are client of the autophagy-lysosomal system, emphasizing the potential therapeutic value of this pathway in ADTKD-UMOD (Suppl. Fig. 14). These new data are reported in Results (P10, P14) and Discussion (P19-20).

Minor

1. The authors should present full Western blots in their Supplementary data

*** Done – Supplementary Figure 16.

2. Figure 2C (and others). Please clarify and label clearly the blots from 1 month and 4-month-old mice.

***The blots have been clarified.

Reviewer #3 (Significance (Required)):

Schiano and colleagues present data on two mouse knock-in models with a missense mutation in uromodulin (C171Y and R186S). A strength of the paper is that the mutations are found in patients with autosomal dominant tubulointerstitial kidney disease (ADTKD) but lead to divergent disease progression. The mouse models are characterized in detail examining changes in uromodulin processing, plasma and urine biochemistry and transcript levels by RNA-sequencing. These findings combined with studies in collecting duct lines provide evidence that the extent of uromodulin aggregate formation is related to the severity of the disease and mechanisms are provided to explain these findings including clearance pathway which might be targeted in the future. Overall, there is a large quantity of good data in the manuscript which moves our understanding of uromodulin mutations forward. However, there are some issues that need to be addressed; in particular the authors should (i) precisely outline the novelty of their study compared with the prior literature; (ii) clarify the reproducibility of their experiments; (iii) refine areas of overinterpretation in the manuscript; (iv) consider the potential role of gender in their findings and (v) complete the circle in some of their findings, for example examining the novel genes identified in their RNA-sequencing in their human biopsy samples and examining autophagy in their mouse models. These changes will considerably strengthen their article.

References:

Olinger E, Hofmann P, Kidd K, et al. Clinical and genetic spectra of autosomal dominant tubulointerstitial kidney disease due to mutations in UMOD and MUC1. Kidney Int. 2020 Sep;98(3):717-731.

Courtoy GE, Leclercq I, Froidure A, et al. Digital Image Analysis of Picrosirius Red Staining: A Robust Method for Multi-Organ Fibrosis Quantification and Characterization. Biomolecules. 2020 Nov 22;10(11):1585.

Dahan K, Fuchshuber A, Adamis S, et al. Familial juvenile hyperuricemic nephropathy and autosomal dominant medullary cystic kidney disease type 2: two facets of the same disease? J Am Soc Nephrol. 2001 Nov;12(11):2348-2357

Arseni D, Hasegawa M, Murzin AG, et al. Structure of pathological TDP-43 filaments from ALS with FTLD. Nature. 2022 Jan;601(7891):139-143.

Kidd K, Vylet'al P, Schaeffer C, et al. Genetic and Clinical Predictors of Age of ESKD in Individuals With Autosomal Dominant Tubulointerstitial Kidney Disease Due to UMOD Mutations. Kidney Int Rep. 2020 Jul 3;5(9):1472-1485.

Olinger E, Schaeffer C, Kidd K, et al. An intermediate-effect size variant in UMOD confers risk for chronic kidney disease. Proc Natl Acad Sci U S A. 2022 Aug 16;119(33):e2114734119.

Bollée G, Dahan K, Flamant M, et al. Phenotype and outcome in hereditary tubulointerstitial nephritis secondary to UMOD mutations. Clin J Am Soc Nephrol. 2011 Oct;6(10):2429-38

Devuyst O, Olinger E, Weber S, et al. Autosomal dominant tubulointerstitial kidney disease. Nat Rev Dis Primers. 2019 Sep 5;5(1):60.

Albertus P, Morgenstern H, Robinson B, Saran R. Risk of ESRD in the United States. Am J Kidney Dis. 2016 Dec;68(6):862-872.

Nangaku M, Kitching AR, Boor P, et al. International Society of Nephrology first consensus guidance for preclinical animal studies in translational nephrology. Kidney Int. 2023 Mar 28:S0085-2538(23)00174-6.

Devuyst O, Olinger E, Rampoldi L. Uromodulin: from physiology to rare and complex kidney disorders. *Nat Rev Nephrol*. 2017;13(9):525-544.

7th Aug 2023

Dear Prof. Devuyst,

Thank you for the submission of your revised manuscript to EMBO Molecular Medicine, and please accept my apologies for the delay in getting back to you in this busy time of the year. We have now received the reports from the referees who had originally reviewed your manuscript for Review Commons. As you will see below, they are supportive of publication, and I am therefore pleased to inform you that we will be able to accept your manuscript once the following points will be addressed:

1/ Referees comments:
- Please address the remaining minor concerns from referee #3.

2/ Manuscript text:
- Please provide a .docx formatted version of the manuscript text without figures (including legends for main figures, EV figures and tables). Please remove the red text and make sure that new changes are highlighted to be clearly visible.
- Please address the queries from our data editors in the Data edited MS file in track changes mode. This file will be sent to you in a couple of days.
- Please provide up to 5 keywords.
- Material and methods:
o Include a statement that informed consent was obtained from all subjects and that the experiments conformed to the principles set out in the WMA Declaration of Helsinki and the Department of Health and Human Services Belmont Report.
o Please indicate whether the cells were authenticated and tested for mycoplasma contamination.
- It is mandatory to include a 'Data Availability' section after the Materials and Methods. Before submitting your revision, primary datasets produced in this study need to be deposited in an appropriate public database, and the accession numbers and database listed under 'Data Availability'. Please remember to provide a reviewer password if the datasets are not yet public (see https://www.embopress.org/page/journal/17574684/authorguide#dataavailability). Note that the Data Availability Section is restricted to new primary data that are part of this study.
- Author contributions: CRediT has replaced the traditional author contributions section because it offers a systematic machine-readable author contributions format that allows for more effective research assessment. Please remove the Authors Contributions from the manuscript and use the free text boxes beneath each contributing author's name in our system to add specific details on the author's contribution. More information is available in our guide to authors.
- Acknowledgements: the complete funding information should be provided both in the acknowledgements and in the submission system (currently, Swiss National Science Foundation (P2ZHP3_195181 and P500PB_206851) and Kidney Research UK (Paed_RP_001_20180925), the European Reference Network for Rare Kidney Diseases (project N{degree sign} 739532), the Swiss National Science Foundation's National Center of Competence in Research Kidney Control of Homeostasis program and Italian Ministry of Health RF-2016- 02362623 are missing in the submission system).
- Disclosure statement and competing interests: We updated our journal's competing interests policy in January 2022 and request authors to consider both actual and perceived competing interests. Please review the policy https://www.embopress.org/competing-interests and update your competing interests if necessary.
- Please reformat the references in alphabetical order, and with 10 authors listed before et al.

3/ Figures:
- The figures should be removed from the manuscript text and uploaded as Please provide individual, high resolution figure files as .eps, .tif, .jpg (one file per figure). For guidance, download the 'Figure Guide PDF' (https://www.embopress.org/page/journal/17574684/authorguide#figureformat).
- Kindly provide exact p values (including for ns, non significant) in the figures or their legends (also in the Appendix).
- The Supplemental file should be renamed "Appendix", a table of content with page numbers should be added, and the figures should be renamed "Appendix Figure S1, etc". Alternatively, you could choose to make these figures Expanded View (EV) Figures that are collapsible/expandable online. EV Figures should be cited as "Figure EV1, Figure EV2" in the text and their respective legends should be included in the main text after the legends of regular figures. The supplementary methods should be removed from the files and merged with the main Materials and Methods

4/ At EMBO Press we ask authors to provide source data for the main figures. Our source data coordinator has contacted you and listed which figure panels we would need source data for and provided indications on how to upload and organize the files.

5/ Please provide a complete author checklist, which you can download from our author guidelines (https://www.embopress.org/page/journal/17574684/authorguide#submissionofrevisions). Please insert information in the checklist that is also reflected in the manuscript. The completed author checklist will also be part of the RPF.

6/ Please provide The paper explained: EMBO Molecular Medicine articles are accompanied by a summary of the articles to emphasize the major findings in the paper and their medical implications for the non-specialist reader. Please provide a draft summary of your article highlighting

- the medical issue you are addressing,
- the results obtained and
- their clinical impact.

This may be edited to ensure that readers understand the significance and context of the research. Please refer to any of our published articles for an example.

7/ For more information: There is space at the end of each article to list relevant web links for further consultation by our readers (Some examples are patient associations, relevant databases, OMIM/proteins/genes links, author's websites, etc...).

8/ Every published paper now includes a 'Synopsis' to further enhance discoverability. Synopses are displayed on the journal webpage and are freely accessible to all readers. They include a short stand first (maximum of 300 characters, including space) as well as 2-5 one-sentences bullet points that summarizes the paper. Please write the bullet points to summarize the key NEW findings. They should be designed to be complementary to the abstract - i.e. not repeat the same text. We encourage inclusion of key acronyms and quantitative information (maximum of 30 words / bullet point). Please use the passive voice. Please attach these in a separate file or send them by email, we will incorporate them accordingly.

Please also suggest a striking image or visual abstract to illustrate your article as a PNG file 550 px wide x 300-600 px high.

9/ As part of the EMBO Publications transparent editorial process initiative (see our Editorial at http://embomolmed.embopress.org/content/2/9/329), EMBO Molecular Medicine will publish online a Review Process File (RPF) to accompany accepted manuscripts.
This file will be published in conjunction with your paper and will include the anonymous referee reports, your point-by-point response and all pertinent correspondence relating to the manuscript. Let us know whether you agree with the publication of the RPF and as here, if you want to remove or not any figures from it prior to publication.
Please note that the Authors checklist will be published at the end of the RPF.

I look forward to receiving your revised manuscript at your earliest convenience.

Yours sincerely,

Lise Roth

Lise Roth, PhD
Senior Editor
EMBO Molecular Medicine

***** Reviewer's comments *****

Referee #1 (Remarks for Author):

The authors have fully addressed all my questions and concerns.
Thanks

Referee #2 (Comments on Novelty/Model System for Author):

Schiano and colleagues present data on two mouse knock-in models with a missense mutation in uromodulin (C171Y and R186S). A strength of the paper is that the mutations are found in patients with autosomal dominant tubulointerstitial kidney disease (ADTKD) but lead to divergent disease progression. The mouse models are characterized in detail examining changes in uromodulin processing, plasma and urine biochemistry and transcript levels by RNA-sequencing. These findings combined with studies in collecting duct lines provide evidence that the extent of uromodulin aggregate formation is related to the severity of the disease and mechanisms are provided to explain these findings including clearance pathway which might be targeted in the future. Overall, there is a large quantity of data in the manuscript which moves our understanding of uromodulin mutations forward.

Referee #2 (Remarks for Author):

In the revised version of the article the authors have responded comprehensively to the reviewers concerns. In particular they have highlighted the novelty, reproducibility of experiments, assessed the role of gender and refined areas of overinterpretation. A significant amount of additional analysis and data (including structural modelling and examining autophagy) has been included strengthening the manuscript. Therefore, I consider the revised paper to be a strong addition to the field of uromodulin mutation.

Referee #3 (Remarks for Author):

I congratulate the authors for this excellent work, they fully addressed my comments/suggestions.

The manuscript is in my opinion ready for publication in EMBO Mol Med, with minor changes.

In the sections reporting on proteasomal (ERAD) and lysosomal (ERLAD)-regulated clearance of aggregates (pages 10, 13-14 and Discussion, page 21) the authors should refer to Rudinskiy and Molinari FEBS Letts 2023 and use more appropriate nomenclature and wording. For example, at page 21, the sentence "However, UPS can only degrade proteins, whereas ER components and protein aggregates are mainly cleared by autophagy-lysosomal degradation (37)" is wrong or unclear. What is the meaning of ER components? If it is ER chaperones, these can also be degraded by the UPS.
To make the text clearer (pages 10, 13-14 and Discussion), I would use the words/acronyms ER-phagy (rather than autophagy); ERAD for ER-associated degradation by the UPS of misfolded proteins generated within the ER; ERLAD for lysosomal clearance of ER portions containing ERAD-resistant misfolded proteins.
The consensus in the field is that ERAD-resistant misfolded proteins (e.g., protein aggregates) are degraded via ER-to-lysosome-associated degradation (ERLAD), ERLAD describes ER-phagy pathways as induced by ER accumulation of misfolded proteins and consisting in misfolded protein segregation in ER subdomains displaying ER-phagy receptors at their limiting membrane (interesting the up-regulation of SEC62, CCPG1 and RTN3 reported in this study) that vesiculate and are eventually delivered to lysosomal compartments for clearance. I would also explicitly write, possibly in the Discussion, that UMOD mutants emerge as emblematic clients of the ER-associated degradation (ERAD, the C170Y variant) and of the ER-to-lysosome-associated degradation (ERLAD) pathways (the R185S and the C170Y variants).

Minor:

Page 10, line 4 from the bottom: ATG5 is a gene product regulating LC3 lipidation (not autophagosome biogenesis).

***
Rev_Com_number: RC-2022-01754
New_manu_number: EMM-2023-18242
Corr_author: Devuyst
Title: Allelic and Gene Dosage Effects Involving Uromodulin Aggregates Drive Autosomal Dominant Tubulointerstitial Kidney Disease

Zurich, September 20, 2023

Re: Decision on EMM-2023-18242

Dear Dr. Roth,

We thank you for your Decision letter (07/08/2023) stating that you will be able to accept our revised manuscript (EMM-2023-18242) in EMBO Molecular Medicine, once we address remaining minor concerns from a referee and format the manuscript as instructed.

We are glad to submit a revised manuscript addressing these minor contents and including all the format points listed. The changes are highlighted in the final version, and detailed in the itemized response below.

We hope that this revised version will meet your approval and wish to thank you for the constructive nature of the reviewing process. Your comments and those of the dedicated referees helped us to improve the manuscript and to widen its conclusions.

Yours sincerely,

Olivier Devuyst, on behalf of the authors

***** Reviewer's comments *****

Referee #1 (Remarks for Author):
The authors have fully addressed all my questions and concerns.
Thanks
*** P15We thank the Reviewer for his/her input, allowing us to improve the revised version of our manuscript.

Referee #2 (Remarks for Author):
In the revised version of the article the authors have responded comprehensively to the reviewers concerns. In particular they have highlighted the novelty, reproducibility of experiments, assessed the role of gender and refined areas of overinterpretation. A significant amount of additional analysis and data (including structural modelling and examining autophagy) has been included strengthening the manuscript. Therefore, I consider the revised paper to be a strong addition to the field of uromodulin mutation.
*** We appreciate the feedback provided by the Reviewer. We were glad to address his/her comments in the revised version of our work.

Referee #3 (Remarks for Author):
The manuscript is in my opinion ready for publication in EMBO Mol Med, with minor changes.
In the sections reporting on proteasomal (ERAD) and lysosomal (ERLAD)-regulated clearance of aggregates (pages 10, 13-14 and Discussion, page 21) the authors should refer to Rudinskiy and Molinari FEBS Letts 2023 and use more appropriate nomenclature and wording. For example, at page 21, the sentence "However, UPS can only degrade proteins, whereas ER components and protein aggregates are mainly cleared by autophagy-lysosomal

degradation (37)" is wrong or unclear. What is the meaning of ER components? If it is ER chaperones, these can also be degraded by the UPS.

To make the text clearer (pages 10, 13-14 and Discussion), I would use the words/acronyms ER-phagy (rather than autophagy); ERAD for ER-associated degradation by the UPS of misfolded proteins generated within the ER; ERLAD for lysosomal clearance of ER portions containing ERAD-resistant misfolded proteins.

The consensus in the field is that ERAD-resistant misfolded proteins (e.g., protein aggregates) are degraded via ER-to-lysosome-associated degradation (ERLAD), ERLAD describes ER-phagy pathways as induced by ER accumulation of misfolded proteins and consisting in misfolded protein segregation in ER subdomains displaying ER-phagy receptors at their limiting membrane (interesting the up-regulation of SEC62, CCPG1 and RTN3 reported in this study) that vesiculate and are eventually delivered to lysosomal compartments for clearance. I would also explicitly write, possibly in the Discussion, that UMOD mutants emerge as emblematic clients of the ER-associated degradation (ERAD, the C170Y variant) and of the ER-to-lysosome-associated degradation (ERLAD) pathways (the R185S and the C170Y variants).

*** We thank the Referee and now use the right acronyms as suggested on P11, P15-17 and Discussion P22, and of Fig. 8A.

Minor:

Page 10, line 4 from the bottom: ATG5 is a gene product regulating LC3 lipidation (not autophagosome biogenesis).

*** The sentence has been modified.

22nd Sep 2023

Dear Prof. Devuyst,

Thank you for the submission of your revised manuscript to EMBO Molecular Medicine.
Almost everything is fine now, and there are only a few minor editorial issues to address before I can proceed with acceptance:

1/ Manuscript text:
- Materials and Methods:
o Please provide the origin of the mice, as well as housing and husbandry conditions.
o Please indicate the cell culture conditions.
- Please rename "Relevant Web Links" to "For more information". Please also remove the link to "KDIGO Consensus Report on ADTKD" has it has a DOI and is a citable item.

2/ Figures:
- Kindly provide exact p values, not a range, in all figures or in their legends, including for ns, non-significant.

Once you have made these changes, please remove the yellow highlights, and only keep in track changes mode any new modification.

As part of the EMBO Publications transparent editorial process initiative (see our Editorial at http://embomolmed.embopress.org/content/2/9/329), EMBO Molecular Medicine will publish online a Review Process File (RPF) to accompany accepted manuscripts. This file will be published in conjunction with your paper and will include the anonymous referee reports, your point-by-point response and all pertinent correspondence relating to the manuscript.

Please let us know whether you agree with the publication of the RPF and as here, if you want to remove or not any figures from it prior to publication.
Please note that the Authors checklist will be published at the end of the RPF.

I look forward to receiving your revised manuscript.

Yours sincerely,

Lise Roth

Lise Roth, PhD
Senior Editor
EMBO Molecular Medicine

***
Rev_Com_number: RC-2022-01754
New_manu_number: EMM-2023-18242-V2
Corr_author: Devuyst
Title: Allelic Effects on Uromodulin Aggregates Drive Autosomal Dominant Tubulointerstitial Kidney Disease

The authors addressed the minor editorial issues.

5th Oct 2023

Dear Prof. Devuyst,

Thank you for submitting your revised files. I am pleased to inform you that your manuscript is accepted for publication and is now being sent to our publisher to be included in the next available issue of EMBO Molecular Medicine!

We would like to remind you that as part of the EMBO Publications transparent editorial process initiative, EMBO Molecular Medicine will publish a Review Process File online to accompany accepted manuscripts. If you do NOT want the file to be published or would like to exclude figures, please immediately inform the editorial office via e-mail.

Please read below for additional IMPORTANT information regarding your article, its publication and the production process.

Congratulations on your interesting work,

With kind regards,

Lise Roth

Lise Roth, Ph.D
Senior Editor
EMBO Molecular Medicine

Follow us on Twitter @EmboMolMed
Sign up for eTOCs at embopress.org/alertsfeeds

*** *** *** IMPORTANT INFORMATION *** *** ***

SPEED OF PUBLICATION
The journal aims for rapid publication of papers, using using the advance online publication "Early View" to expedite the process: A properly copy-edited and formatted version will be published as "Early View" after the proofs have been corrected. Please help the Editors and publisher avoid delays by providing e-mail address(es), telephone and fax numbers at which author(s) can be contacted.

Should you be planning a Press Release on your article, please get in contact with embomolmed@wiley.com as early as possible, in order to coordinate publication and release dates.

LICENSE AND PAYMENT:

All articles published in EMBO Molecular Medicine are fully open access: immediately and freely available to read, download and share.

EMBO Molecular Medicine charges an article processing charge (APC) to cover the publication costs. You, as the corresponding author for this manuscript, should have already received a quote with the article processing fee separately. Please let us know in case this quote has not been received.

Once your article is at Wiley for editorial production you will receive an email from Wiley's Author Services system, which will ask you to log in and will present you with the publication license form for completion. Within the same system the publication fee can be paid by credit card, an invoice, pro forma invoice or purchase order can be requested.

Payment of the publication charge and the signed Open Access Agreement form must be received before the article can be published online.

PROOFS

You will receive the proofs by e-mail approximately 2 weeks after all relevant files have been sent o our Production Office. Please return them within 48 hours and if there should be any problems, please contact the production office at embopressproduction@wiley.com.

Please inform us if there is likely to be any difficulty in reaching you at the above address at that time. Failure to meet our deadlines may result in a delay of publication.

All further communications concerning your paper proofs should quote reference number EMM-2023-18242-V3 and be directed to the production office at embopressproduction@wiley.com.

***

Rev_Com_number: RC-2022-01754
New_manu_number: EMM-2023-18242-V3
Corr_author: Devuyst
Title: Allelic Effects on Uromodulin Aggregates Drive Autosomal Dominant Tubulointerstitial Kidney Disease

EMBO Press Author Checklist

Corresponding Author Name: Olivier Devuyst
Journal Submitted to: EMBO Molecular Medicine
Manuscript Number: EMM-2023-18242

USEFUL LINKS FOR COMPLETING THIS FORM
The EMBO Journal - Author Guidelines
EMBO Reports - Author Guidelines
Molecular Systems Biology - Author Guidelines
EMBO Molecular Medicine - Author Guidelines

Reporting Checklist for Life Science Articles (updated January
This checklist is adapted from Materials Design Analysis Reporting (MDAR) Checklist for Authors. MDAR establishes a minimum set of requirements in transparent reporting in the life sciences (see Statement of Task: 10.31222/osf.io/9sm4x). Please follow the journal's guidelines in preparing your
Please note that a copy of this checklist will be published alongside your article.

Abridged guidelines for figures
1. Data
The data shown in figures should satisfy the following conditions:
- the data were obtained and processed according to the field's best practice and are presented to reflect the results of the experiments in an accurate and unbiased manner.
- ideally, figure panels should include only measurements that are directly comparable to each other and obtained with the same assay.
- plots include clearly labeled error bars for independent experiments and sample sizes. Unless justified, error bars should not be shown for technical
- if n<5, the individual data points from each experiment should be plotted. Any statistical test employed should be justified.
- Source Data should be included to report the data underlying figures according to the guidelines set out in the authorship guidelines on Data

2. Captions
Each figure caption should contain the following information, for each panel where they are relevant:
- a specification of the experimental system investigated (eg cell line, species name).
- the assay(s) and method(s) used to carry out the reported observations and measurements.
- an explicit mention of the biological and chemical entity(ies) that are being measured.
- an explicit mention of the biological and chemical entity(ies) that are altered/varied/perturbed in a controlled manner.
- the exact sample size (n) for each experimental group/condition, given as a number, not a range;
- a description of the sample collection allowing the reader to understand whether the samples represent technical or biological replicates (including how many animals, litters, cultures, etc.).
- a statement of how many times the experiment shown was independently replicated in the laboratory.
- definitions of statistical methods and measures:
  - common tests, such as t-test (please specify whether paired vs. unpaired), simple χ2 tests, Wilcoxon and Mann-Whitney tests, can be unambiguously identified by name only, but more complex techniques should be described in the methods section;
  - are tests one-sided or two-sided?
  - are there adjustments for multiple comparisons?
  - exact statistical test results, e.g., P values = x but not P values < x;
  - definition of 'center values' as median or average;
  - definition of error bars as s.d. or s.e.m.

Please complete ALL of the questions below.
Select "Not Applicable" only when the requested information is not relevant for your study.

Materials

Newly Created Materials	Information included in the manuscript?	In which section is the information available? (Reagents and Tools Table, Materials and Methods, Figures, Data Availability Section)
New materials and reagents need to be available; do any restrictions apply?	Not Applicable	

Antibodies	Information included in the manuscript?	In which section is the information available? (Reagents and Tools Table, Materials and Methods, Figures, Data Availability Section)
For **antibodies** provide the following information:<br>- Commercial antibodies: RRID (if possible) or supplier name, catalogue number and or/clone number<br>- Non-commercial: RRID or citation	Yes	Appendix Table S13

DNA and RNA sequences	Information included in the manuscript?	In which section is the information available? (Reagents and Tools Table, Materials and Methods, Figures, Data Availability Section)
**Short novel DNA or RNA including primers, probes**: provide the sequences.	Yes	Appendix Table S14

Cell materials	Information included in the manuscript?	In which section is the information available? (Reagents and Tools Table, Materials and Methods, Figures, Data Availability Section)
**Cell lines:** Provide species information, strain. Provide accession number in repository **OR** supplier name, catalog number, clone number, and/**OR** RRID.	Yes	Materials and Methods
**Primary cultures:** Provide species, strain, sex of origin, genetic modification status.	Not Applicable	
Report if the cell lines were recently **authenticated** (e.g., by STR profiling) and tested for mycoplasma contamination.	Yes	Materials and Methods

Experimental animals	Information included in the manuscript?	In which section is the information available? (Reagents and Tools Table, Materials and Methods, Figures, Data Availability Section)
**Laboratory animals or Model organisms:** Provide species, strain, sex, age, genetic modification status. Provide accession number in repository **OR** supplier name, catalog number, clone number, **OR** RRID.	Yes	Materials and Methods
**Animal observed in or captured from the field:** Provide species, sex, and age where possible**.**	Not Applicable	
Please detail **housing and husbandry conditions**.	Yes	Materials and Methods

Plants and microbes	Information included in the manuscript?	In which section is the information available? (Reagents and Tools Table, Materials and Methods, Figures, Data Availability Section)
**Plants:** provide species and strain, ecotype and cultivar where relevant, unique accession number if available, and source (including location for collected wild specimens).	Not Applicable	
**Microbes:** provide species and strain, unique accession number if available, and source.	Not Applicable	

Human research participants	Information included in the manuscript?	In which section is the information available? (Reagents and Tools Table, Materials and Methods, Figures, Data Availability Section)
If collected and within the bounds of privacy constraints report on age, sex and gender or ethnicity for all study participants.	Yes	Appendix

Core facilities	Information included in the manuscript?	In which section is the information available? (Reagents and Tools Table, Materials and Methods, Figures, Data Availability Section)
If your work benefited from core facilities, was their service mentioned in the acknowledgments section?	Not Applicable	

Design

Study protocol	Information included in the manuscript?	In which section is the information available? (Reagents and Tools Table, Materials and Methods, Figures, Data Availability Section)
If study protocol has been **pre-registered, provide DOI in the manuscript**. For clinical trials, provide the trial registration number **OR** cite DOI.	Not Applicable	
Report the **clinical trial registration number** (at ClinicalTrials.gov or equivalent), where applicable.	Not Applicable	

Laboratory protocol	Information included in the manuscript?	In which section is the information available? (Reagents and Tools Table, Materials and Methods, Figures, Data Availability Section)
Provide DOI OR other citation details if **external detailed step-by-step protocols** are available.	Not Applicable	

Experimental study design and statistics	Information included in the manuscript?	In which section is the information available? (Reagents and Tools Table, Materials and Methods, Figures, Data Availability Section)
Include a statement about **sample size** estimate even if no statistical methods were used.	Yes	Materials and Methods
Were any steps taken to minimize the effects of subjective bias when allocating animals/samples to treatment (e.g. **randomization procedure**)? If yes, have they been described?	Not Applicable	
Include a statement about **blinding** even if no blinding was done.	Yes	Materials and Methods
Describe **inclusion/exclusion criteria** if samples or animals were excluded from the analysis. Were the criteria pre-established?<br><br>If sample or data points were omitted from analysis, report if this was due to attrition or intentional exclusion and provide justification.	Yes	Materials and Methods
For every figure, are **statistical tests** justified as appropriate? Do the data meet the assumptions of the tests (e.g., normal distribution)? Describe any methods used to assess it. Is there an estimate of variation within each group of data? Is the variance similar between the groups that are being statistically compared?	Yes	Materials and Methods

Sample definition and in-laboratory replication	Information included in the manuscript?	In which section is the information available? (Reagents and Tools Table, Materials and Methods, Figures, Data Availability Section)
In the figure legends: state number of times the experiment was **replicated** in laboratory.	Yes	Figure Legends
In the figure legends: define whether data describe **technical or biological replicates**.	Yes	Figure Legends

Ethics

Ethics	Information included in the manuscript?	In which section is the information available? (Reagents and Tools Table, Materials and Methods, Figures, Data Availability Section)
Studies involving **human participants**: State details of **authority granting ethics approval** (IRB or equivalent committee(s), provide reference number for approval.	Yes	Materials and Methods
Studies involving **human participants**: Include a statement confirming that **informed consent** was obtained from all subjects and that the experiments conformed to the principles set out in the WMA Declaration of Helsinki and the Department of Health and Human Services Belmont Report.	Yes	Materials and Methods
Studies involving **human participants:** For publication of **patient photos**, include a statement confirming that consent to publish was obtained.	Not Applicable	
Studies involving experimental **animals**: State details of **authority granting ethics approval** (IRB or equivalent committee(s), provide reference number for approval. Include a statement of compliance with ethical regulations.	Yes	Materials and Methods
Studies involving **specimen and field samples:** State if relevant **permits** obtained, provide details of authority approving study; if none were required, explain why.	Not Applicable	

Dual Use Research of Concern (DURC)	Information included in the manuscript?	In which section is the information available? (Reagents and Tools Table, Materials and Methods, Figures, Data Availability Section)
Could your study fall under dual use research restrictions? Please check biosecurity documents and list of **select agents and toxins** (CDC): https://www.selectagents.gov/sat/list.htm	Not Applicable	
If you used a select agent, is the security level of the lab appropriate and reported in the manuscript?	Not Applicable	
If a study is subject to dual use research of concern regulations, is the name of the **authority granting approval and reference number** for the regulatory approval provided in the manuscript?	Not Applicable	

Reporting

The MDAR framework recommends adoption of discipline-specific guidelines, established and endorsed through community initiatives. Journals have their own policy about requiring specific guidelines and recommendations to complement MDAR.

Adherence to community standards	Information included in the manuscript?	In which section is the information available? (Reagents and Tools Table, Materials and Methods, Figures, Data Availability Section)
State if relevant guidelines or checklists (e.g., **ICMJE, MIBBI, ARRIVE, PRISMA**) have been followed or provided.	Yes	
For **tumor marker prognostic studies,** we recommend that you follow the **REMARK** reporting guidelines (see link list at top right). See author guidelines, under 'Reporting Guidelines'. Please confirm you have followed these guidelines.	Not Applicable	
For **phase II and III randomized controlled trials**, please refer to the **CONSORT** flow diagram (see link list at top right) and submit the CONSORT checklist (see link list at top right) with your submission. See author guidelines, under 'Reporting Guidelines'. Please confirm you have submitted this list.	Not Applicable	

Data Availability

Data availability	Information included in the manuscript?	In which section is the information available? (Reagents and Tools Table, Materials and Methods, Figures, Data Availability Section)
Have **primary datasets** been deposited according to the journal's guidelines (see 'Data Deposition' section) and the respective accession numbers provided in the Data Availability Section?	Yes	Data Availability
Were **human clinical and genomic datasets** deposited in a public access-controlled repository in accordance to ethical obligations to the patients and to the applicable consent agreement?	Not Applicable	
Are **computational models** that are central and integral to a study available without restrictions in a machine-readable form? Were the relevant accession numbers or links provided?	Not Applicable	
If publicly available data were reused, provide the respective **data citations in the reference list**.	Yes	Appendix
